# Global excess tuberculosis mortality during COVID-19: a country-level modeling study of policy and development correlates

**DOI:** 10.1186/s12889-025-24858-8

**Published:** 2025-10-31

**Authors:** Hamed Karami, Svenn-Erik Mamelund, Alexandra Smirnova, Gerardo Chowell

**Affiliations:** 1 Department of Mathematics & Statistics, Georgia State University, Atlanta, GA 30303 USA; 2 Centre for Research on Pandemics & Society (PANSOC), Oslo Metropolitan University, Oslo, 0130 Norway; 3 Department of Population Health Sciences, Georgia State University, Atlanta, GA 30303 USA

**Keywords:** Excess deaths, Tuberculosis, COVID-19, Stringency index

## Abstract

**Background:**

The COVID-19 pandemic disrupted global tuberculosis (TB) control efforts, leading to a surge in TB-related excess mortality, particularly in low- and middle-income countries. Pandemic mitigation measures, such as lockdowns, reallocation of healthcare resources, and reduced access to diagnosis and treatment, contributed to delayed TB care and disease progression. Quantifying this collateral damage is crucial to bolstering health system resilience.

**Methods:**

We estimated country-level excess TB mortality between 2020 and 2023 using annual TB mortality data reported by the World Health Organization (WHO). Our approach leverages the SubEpiPredict toolbox of the ensemble n-sub-epidemic modeling framework, calibrated to pre-pandemic trends (2010–2019) to forecast expected TB deaths in the absence of COVID-19 disruptions. We selected the best-fitting model based on AICc and compared projected and reported deaths to quantify excess mortality, incorporating both normal and Poisson error structures. We further examined associations between excess TB mortality and country-level indicators, including the COVID-19 Stringency Index, Global Health Security (GHS) Index, and Socio-demographic Index (SDI). It is also noteworthy that for each estimate of excess TB deaths, we provide an associated uncertainty.

**Results:**

Our global estimate of 755,876 excess TB deaths (95%CI: 591,099 to 965,015) aligns closely with the WHO estimate of approximately 700,000 excess deaths. Therefore, it can be estimated a global relative excess mortality of 12.8% (95%CI: 11.6% to 14.2%), compared to the WHO estimate of 14.6% (95% CI: 5.9% to 26.7%). We found substantial geographic heterogeneity, with the highest TB excess mortality rates observed in southern Africa, South Asia, and parts of South America. Countries with high GHS or SDI scores did not necessarily exhibit lower excess TB mortality, suggesting that pandemic-specific disruptions and competing priorities may have overridden structural advantages. Weak-to-moderate correlations were observed between excess mortality and pandemic stringency, peaking in 2021 and waning by 2022, possibly reflecting health system adaptation.

**Conclusion:**

This study presents a systematic, model-based analysis of global excess TB mortality during the COVID-19 pandemic, emphasizing disparities in pandemic response impacts across countries. The findings highlight the need for integrated and resilient public health systems capable of maintaining essential services amid global crises. Our methodology can support real-time monitoring of collateral effects of pandemics on endemic diseases and guide strategic investments in TB surveillance and care continuity.

**Supplementary Information:**

The online version contains supplementary material available at 10.1186/s12889-025-24858-8.

## Introduction

Tuberculosis (TB), caused by *Mycobacterium tuberculosis* (MTB), remains one of the most persistent and deadly infectious diseases worldwide [1, 2]. It is transmitted primarily through airborne droplets [3] expelled when an infected person coughs, sneezes, shouts, sings, or talks [3–5]. Although less common, transmission can also occur through contaminated surfaces or utensils [6], and vertical transmission from mother to child has been documented [7]. While TB primarily affects the lungs [8, 9], it can also impact other organs including the brain [10], heart [11], liver [12], spine [13], kidneys [14], and lymphatic system [15]. It is estimated that approximately one-quarter of the global population carries latent TB infection [16–18], which is asymptomatic and non-infectious. However, under conditions of immune suppression, latent TB can progress to active disease. Notably, 95% of active TB cases are concentrated in developing countries, where social determinants and limited healthcare access amplify transmission [19].

TB outcomes are often worsened by comorbidities such as HIV and, more recently, COVID-19, both of which impair immune responses and may facilitate reactivation of latent TB [20–22]. Paradoxically, while stringent lockdowns may have reduced transmission of active TB, they also disrupted services and may have indirectly increased TB-related mortality by delaying diagnosis and treatment. Sub-Saharan Africa continues to carry the highest burden of TB deaths, particularly among children under 15 [23, 24].

Controlling TB has remained a central public health objective for over a century [25–27]. Key strategies have included BCG vaccination [28], isolation of infectious individuals [29], and preventive therapy [30]. However, TB control efforts have faced persistent and evolving challenges [31, 32]. The HIV epidemic in the 1980 s severely impacted TB control by increasing host susceptibility [33, 34]. The rise of multidrug-resistant TB strains has further complicated treatment [35–37], while chronic underfunding has left TB programs without essential diagnostic tools, treatments, and trained personnel [38–40]. Armed conflicts and humanitarian crises have further disrupted TB control by displacing populations and damaging health infrastructure [41–44].

The COVID-19 pandemic, which began in late 2019, triggered unprecedented global public health responses [45]. Governments imposed lockdowns, closed institutions, and restricted travel [46–49], which succeeded in mitigating SARS-CoV-2 spread. However, these same interventions disrupted access to routine health services, including TB diagnosis and treatment [50, 51]. Many TB clinics were shuttered or repurposed for COVID-19 care [52, 53], leading to delayed case detection, treatment interruption, and potentially increased transmission and mortality. Compounding these effects, SARS-CoV-2 infection may contribute to immune dysregulation, increasing the risk of latent TB reactivation [54]. The WHO’s End TB Strategy, adopted in 2014, set a target to end the TB epidemic by 2035, with the Sustainable Development Goals (SDGs) aiming for 2030 [55–57]. The COVID-19 pandemic has placed both goals at risk, with some estimates suggesting a decade-long setback in progress [58].

To mitigate future disruptions, it is essential to rigorously assess how COVID-19 has impacted TB trends and to evaluate health systems’ resilience in maintaining TB control during pandemics [59, 60].

The WHO estimated that approximately 700,000 excess TB deaths occurred globally between 2020 and 2023 [56]. Other estimates include 7,000 excess TB deaths in Europe [61, 62], and roughly 400,000 deaths in four high-burden countries (Pakistan, India, Indonesia, and Kenya) [63]. Excess TB mortality in China during the COVID-19 pandemic has also been explored, though estimates remain limited [64]. There has also been several studies on modeling the potential impact of COVID-19 pandemic on the TB epidemic [65, 66]

Building on these insights, we present a global assessment of excess TB mortality from 2020 to 2023 using WHO-reported data and a calibrated sub-epidemic forecasting model [67]. We further explore the role of pandemic policy responses and structural health determinants in shaping these outcomes [68].

This manuscript is organized as follows: Data section describes the data sources; Methodology section outlines the modeling framework; Results section presents the results for eight high TB burden countries; Bangladesh, China, the Democratic Republic of Congo, India, Indonesia, Nigeria, Pakistan, and the Philippines, as well as global estimates. Discussion section concludes with a discussion of key findings and implications.

## Data

Global Tuberculosis Report 2024, published by WHO on 29 October 2024, provides a comprehensive assessment of the TB epidemic and progress in prevention, diagnosis, and treatment at a global, regional, and national level. This report is based on data from 193 countries and areas, covering over 99% of the world’s population and TB cases[56].

In 2023, the global number of TB-related deaths continued the downward trend first observed in 2022, following increases during the early years of the COVID-19 pandemic (2020–2021). Despite this progress, TB has likely resumed its position as the leading cause of death from a single infectious agent, according to Dr. Tereza Kasaeva, Director of the WHO Global TB Programme [56].

While TB incidence continued to rise between 2020 and 2023, the growth rate stabilized by 2023. The estimated $2\%$ increase in incidence between 2022 and 2023 is largely attributed to population growth. Thirty high-burden countries account for $87\%$ of global TB incidence, with five countries—India, Indonesia, China, the Philippines, and Pakistan—alone contributing $56\%$ of all cases. The observed decline in TB mortality beginning in 2022 is believed to reflect a partial recovery in diagnostic and treatment services following pandemic-related disruptions.

Our analysis includes all countries for which WHO data are available. However, for clarity, we focus the main results on eight high-burden countries: India, Indonesia, China, the Philippines, Pakistan, Nigeria, Bangladesh, and the Democratic Republic of the Congo (DRC) [69], while results for other countries are presented in the supplementary materials.

**Fig. 1:**
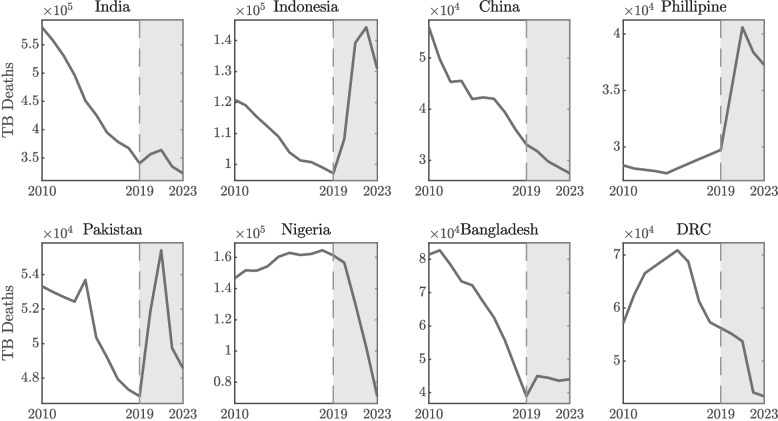
Annual reported tuberculosis (TB) deaths from 2010 to 2023 in eight high-burden countries—India, Indonesia, China, the Philippines, Pakistan, Nigeria, Bangladesh, and the Democratic Republic of the Congo (DRC)—based on World Health Organization (WHO) data. These countries collectively account for a substantial proportion of the global TB burden. The figure illustrates temporal trends and highlights pandemic-era shifts in TB mortality

Figure 1 shows TB deaths in the eight high-burden countries from 2010 to 2023. A significant increase in TB deaths was observed in India, Indonesia, the Philippines, Pakistan, and Bangladesh, during the COVID-19 pandemic. In contrast, China, Nigeria, and the DRC experienced a decline in reported TB deaths during the pandemic, likely due to underreporting or misclassification as COVID-19 deaths.

To assess how COVID-19 policies may have affected TB deaths, we used the Oxford COVID-19 Government Response Tracker (OxCGRT) Stringency Index. This index measures how strict a country’s COVID-19 rules were, based on things like school and workplace closures, travel restrictions, stay-at-home orders, and other key policies. Each of these nine measures is scored from 0 to 100, and the average gives an overall stringency score, with 100 meaning the strictest possible response. Because guidelines sometimes differed for vaccinated and unvaccinated people, the index includes separate scores for each group. It also provides a national average, which is weighted based on how many people in the country were vaccinated. This gives a more balanced picture of how strict the measures were for the population as a whole [68, 70]. The dataset in 2020, 2021, and 2022, along with a detailed explanation of the methodology, is available here.

We also compared TB excess deaths with the Global Health Security (GHS) Index, which evaluates countries’ capacity to prevent, detect, and respond to biological threats across six categories: prevention, detection, rapid response, health system, compliance with international norms, and risk environment. The most recent GHS Index data (2021) and full methodological details are publicly available GHS Index Report.

In addition, we used the Socio-demographic Index (SDI), developed by the Global Burden of Disease Study 2021 (GBD 2021), as a proxy for development level. SDI is a composite of income per capita, average educational attainment among individuals aged 15 and older, and total fertility rate among women under age 25. SDI values range from 0 (least developed) to 1 (most developed). Data include annual SDI estimates from 1950 to 2021 for 204 countries and territories [71].

To estimate TB excess deaths per 100,000 population, we used 2021 population data from the GBD results tool (https://vizhub.healthdata.org/gbd-results/). The global population growth rate was about 1% in 2020 and dropped to 0.87% in 2021 (MacroTrends), and we consider this a valid approximation for the 2020–2023 period.

## Methodology

In this section, we detail the modeling framework used to estimate TB excess deaths attributable to the COVID-19 pandemic between 2020 and 2023.

We utilized annual TB mortality data from the WHO for all available countries and regions. To estimate the counterfactual number of TB deaths that would have occurred in the absence of the pandemic, we calibrated our model using data from the pre-pandemic period (2010–2019) and generated forecasts for 2020–2023.

To capture the multi-phase nature of TB mortality trends, we modeled each country’s trajectory as a sum of up to n sub-epidemics, each governed by a generalized logistic growth curve. The dynamics are described by the following system of differential equations [72]:

1 $$\begin{aligned} \dfrac{dC_i(t)}{dt} = A_i(t) r_i C_i^{p_i}(t) \left( 1- \dfrac{C_i(t)}{K_{0_i}}\right), \quad i = 1,\cdots,n, \end{aligned}$$

where $C_i$ represents the cumulative number of deaths, $r_i$ denotes the growth rate, $p_i$ is called the scaling of growth, and $K_{0_i}$ is the final outbreak size, for the $i_\text {th}$ sub-epidemic, $i=\lbrace 1,\cdots,n\rbrace$. The onset timing of the $i_\text {th}$ sub-epidemic is employed by an indicator function $A_i$ which is given by

2 $$\begin{aligned} A_i(t) &= \left\{ \begin{array}{ll} 1 & C_{i-1}(t)>C_{\text {thr}}\\ 0 & \text {Otherwise} \end{array}\right., \quad i = 2,\cdots, n, \end{aligned}$$

where $A_1(t) =1$ for the first sub-epidemic and $C_{\text {thr}}$ is the threshold. Therefore, the sub-epidemic *i* triggers as soon as $C_{i-1}>C_{\text {thr}}$. The initial number of deaths is given by $C_1(0)$ which is the initial number of deaths in the reported data. The values of $C_{\text {thr}}$ are obtained by equally discretizing the cumulative sum of the smoothed signal into as many levels as there are data points. This approach treats $C_{\text {thr}}$ as a range of candidate thresholds, enabling the model to be evaluated at each fixed level rather than estimating the threshold directly from the data. We use the corrected Akaike Information Criterion ($AIC_c$) to select the top-ranked sub-epidemic models. This is done by calculating the $AIC_c$ values for the best-fitting models using different values of $C_{\text {thr}}$. The $AIC_c$ is calculated as follows [73, 74]:

3 $$\begin{aligned} AIC_c = -2 \log (\text {likelihood}) + 2m + \frac{2m(m + 1)}{n_d - m - 1}, \end{aligned}$$

where *m* is the total number of model parameters, and $n_d$ is the number of data points.

In this study, we utilize the SubEpiPredict toolbox for fitting and forecasting growth trajectories using the ensemble n-sub-epidemic modeling framework [67]. Each sub-epidemic follows a three-parameter generalized logistic growth model as mentioned earlier. For each value of the threshold parameter $C_{\text {thr}}$, we consider two model structures: one with $n=1$ sub-epidemic (i.e., $m=3$ parameters) and another with $n=2$ sub-epidemics (i.e., $m=6$ parameters), resulting in two models with different complexities for each choice of $C_{\text {thr}}$. Therefore, we estimate the parameters $r_i$, $p_i$, and $K_{0i}$ from the data, while $C_{\textrm{thr}}$ obtained by systematically scanning a discretized grid of values and choosing the one that yields the lowest AICc, ensuring the model best captures the underlying temporal dynamics. Model parameters are estimated using the nonlinear least squares (NLS) method. During the modeling process, we selected the top-ranked model within the *n*-sub-epidemic framework based on the $AIC_c$ values.

For parameter estimation, we employed the MultiStart optimization method with 30 different initial guesses to ensure robustness. We did not assume that the onset timing of sub-epidemics is fixed at zero, allowing for greater flexibility in capturing epidemic dynamics. To assess the accuracy of the upper bound of the 95% prediction interval (PI) and provide a qualitative measure of uncertainty, we performed 300 bootstrap resampling iterations.

The Poisson error structure usually results in a narrower uncertainty range, which enhances the accuracy of death count estimations. Consequently, we generate forecasts using both normal and Poisson error structures. The final selection is based on the model that yields the 95%PI that best captures the data during the calibration phase.

Let ${\textbf {Y}} = \lbrace y_{2020}, y_{2021}, y_{2022}, y_{2023}\rbrace$ denotes the number of deaths from which we calculate excess mortality. The number of TB deaths forecast for each year is denoted by $\hat{{\textbf {Y}}} = \lbrace \hat{y}_{2020}, \hat{y}_{2021}, \hat{y}_{2022}, \hat{y}_{2023}\rbrace$. Also for simplicity, let us denote the lower and upper bound of this forecast by $L_j$ and $U_j$, where $j = \lbrace 2020, 2021, 2022, 2023\rbrace$. We estimate the number of excess TB deaths, *ETB*, coming with a lower $ETB_{LB_j}$, and an upper bound $ETB_{UB_j}$, as following

4 $$\begin{aligned}ETB_j &= \max\{0, y_j - \hat{y}_j\}, \\ETB_{LB,j} &= \max\{0, y_j - U_j\}, \\ETB_{UB,j} &= \max\{0, y_j - L_j\},\end{aligned}$$

where $$j = \lbrace 2020, 2021, 2022, 2023\rbrace$$.

Finally, we calculate the total number of excess deaths, $ETB_{\text {total}}$ coming with a lower and an upper bound as below

5 $$\begin{aligned}ETB_{\text{total}} &= \sum_{j=2020}^{2023} ETB_{j}, \\ETB_{LB_\text{total}} &= \sum_{j=2020}^{2023} ETB_{LB,j}, \\ETB_{UB_\text{total}} &= \sum_{j=2020}^{2023} ETB_{UB,j}.\end{aligned}$$

Note that while prediction intervals ideally should not be simply summed across years due to potential correlations, in this analysis we sum the yearly lower and upper bounds to approximate the total bounds. This approach is justified by the distinct nature of each pandemic year, with differing public health dynamics and disruptions affecting TB mortality independently. Therefore, summing the bounds provides a reasonable and practical estimate of the total uncertainty over the multi-year period.

We also compute the excess TB death rate per 100,000 population, denoted by *ETBR*, for each year $j \in \{2020, 2021, 2022, 2023\}$ and in total, using the 2021 population as:

6 $$\begin{aligned}& ETBR_{j} = \frac{ETB_{j}}{\text {Population}_{2021}} \times 100{,}000, \\& ETBR_{\text {total}} = \frac{ETB_{\text {total}}}{\text {Population}_{2021}} \times 100{,}000. \end{aligned}$$

In addition, we calculate the Standardized Mortality Ratio (SMR), which compares observed TB deaths to expected deaths for each year and in total:

7 $$\begin{aligned}& SMR_{j} = \frac{y_{j}}{\hat{y}_{j}}, \\&\quad SMR_{\text {total}} = \frac{\sum _{j=2020}^{2023} y_{j}}{\sum _{j=2020}^{2023} \hat{y}_{j}}. \end{aligned}$$

An $SMR> 1$ indicates excess mortality relative to the expected baseline, while an $SMR < 1$ implies fewer deaths than expected.

## Results

In this section, we present results for the eight high-burden countries described earlier. We also provide estimates for nearly all countries globally, as well as aggregated results by region, with detailed data available in the supplementary file. To enhance clarity and usability for public health officials and policymakers, we include a set of comprehensive figures and tables. In addition, we present two global maps: one displaying the estimated excess TB mortality rate per 100,000 population and another illustrating the SMR, enabling cross-country comparisons of pandemic-related disruptions.

### National-level trends in high-burden countries

**Fig. 2:**
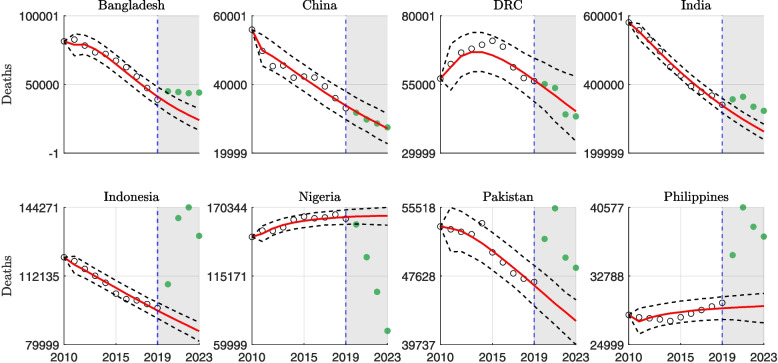
Model calibration and forecasting of annual tuberculosis (TB) deaths from 2010 to 2023 for eight high-burden countries—India, Indonesia, China, the Philippines, Pakistan, Nigeria, Bangladesh, and the Democratic Republic of the Congo (DRC)—using WHO-reported mortality data and an ensemble n-sub-epidemic modeling framework. The model is calibrated to the pre-pandemic period (2010–2019) and used to generate forecasts for the pandemic period (2020–2023). Red curves represent the median model predictions, while black dashed lines denote the 95% prediction intervals (PIs). Hollow black circles indicate observed TB deaths for the calibration period. Green filled circles represent observed TB deaths for the forecast period (2020–2023)

Figure 2 presents TB mortality trends from 2010 to 2023 for the eight high-burden countries discussed earlier. Among them, Nigeria is the only country that shows no evidence of excess TB mortality during the COVID-19 pandemic (2020–2023). In contrast, countries such as Indonesia, Pakistan, and the Philippines exhibited notably high levels of excess TB mortality, while Bangladesh, China, the DRC, and India showed comparatively lower excess death levels.

### Excess mortality rates per 100,000 population

**Fig. 3:**
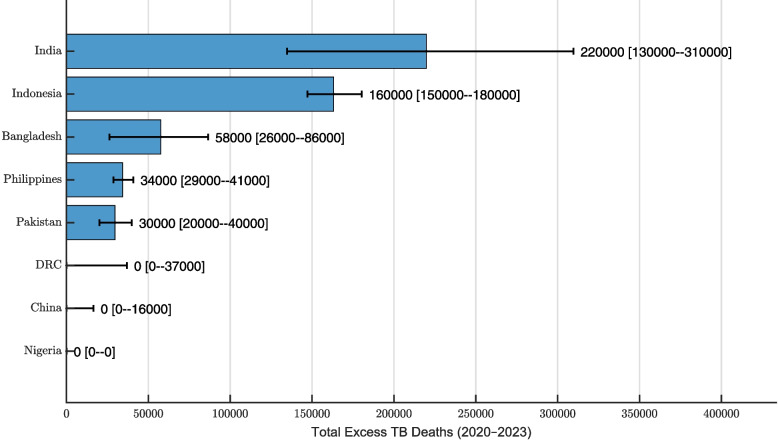
Estimated total excess tuberculosis (TB) deaths from 2020 to 2023 in eight high-burden countries—Bangladesh, India, Indonesia, Pakistan, the Philippines, China, Nigeria, and the Democratic Republic of the Congo (DRC)—based on an ensemble n-sub-epidemic modeling framework calibrated to pre-pandemic mortality trends. The horizontal bars represent the median estimates of excess TB mortality, while the accompanying lines denote the 95% uncertainty bounds derived from bootstrap simulations. This visualization highlights the heterogeneity in excess mortality burden across countries, with Indonesia and India contributing disproportionately to global excess TB deaths, while China and Nigeria show minimal or no excess, possibly reflecting stronger TB control programs or limitations in mortality reporting

Figure 3 illustrates total excess TB deaths across the eight high-burden countries from 2020 to 2023. India, Indonesia, and Bangladesh contributed the largest absolute numbers of excess TB deaths over this period. Notably, Indonesia’s relatively narrow uncertainty bounds suggest a high degree of confidence in the estimate, reinforcing the magnitude of its excess mortality burden. The Philippines and Pakistan follow, with comparable excess TB death totals. The DRC shows a wide upper bound for excess mortality, indicating substantial uncertainty and the possibility that actual deaths may exceed the median estimate. In contrast, China and Nigeria—despite their large populations—exhibited minimal or no excess TB deaths during the pandemic.

These patterns are further clarified in Table 1, which provides annual estimates of excess TB deaths and highlights temporal trends across the COVID-19 period. Consistent with earlier findings, Nigeria did not report any excess TB deaths during the COVID-19 period. The table also indicates that China began to experience excess TB mortality only in 2023, while the DRC reported excess deaths in 2020 and 2021 but none thereafter, suggesting improved management of TB services as the pandemic progressed. In contrast, countries such as Indonesia, India, Bangladesh, Pakistan, and the Philippines experienced persistent excess TB mortality from 2020 to 2023, reflecting sustained disruption of TB services throughout the pandemic. The particularly high excess mortality in Indonesia stands out and likely warrants further exploration into potential health system vulnerabilities or other contributing factors. Although Bangladesh and India reported substantial excess TB deaths, this may be partially attributable to their large population sizes.

**Fig. 4:**
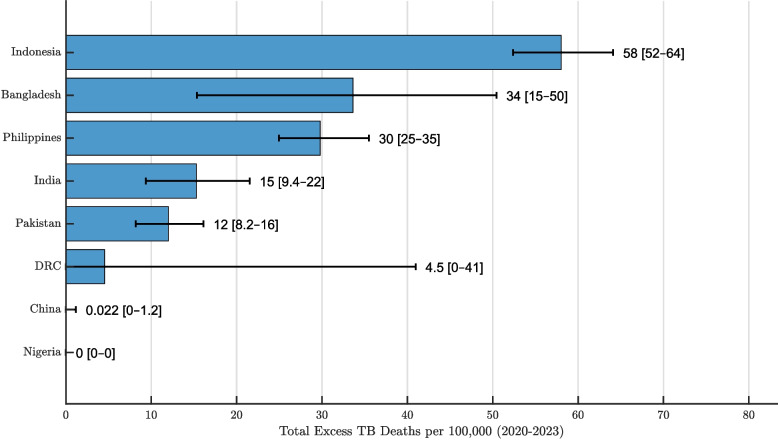
Estimated total excess tuberculosis (TB) mortality rates (per 100,000 population) from 2020 to 2023 in eight high-burden countries—Bangladesh, India, Indonesia, Pakistan, the Philippines, China, Nigeria, and the Democratic Republic of the Congo (DRC)—based on an ensemble n-sub-epidemic modeling framework with two overlapping epidemic waves. Horizontal bars indicate the median estimated excess mortality rate for each country, while horizontal lines denote the corresponding 95% uncertainty intervals. Exact rates are labeled adjacent to each bar for ease of comparison. This figure reveals substantial variation in the magnitude of excess TB mortality across countries, with the highest rates observed in Indonesia, Bangladesh, and the Philippines, and minimal excess in China and Nigeria

While excess TB deaths counts provide insight into absolute burden, comparing mortality rates allows for population-adjusted interpretations. Figure 4 displays the estimated excess TB mortality rates (per 100,000 population) across high-burden countries during the COVID-19 period. The DRC shows a relatively wide upper uncertainty bound, suggesting some imprecision in the estimate. This may reflect data limitations during the pandemic, such as disruptions in reporting or reduced availability of timely mortality information. These factors could have contributed to greater variability in the model estimates. In contrast, most other countries display narrower uncertainty intervals, suggesting more stable results. Indonesia ranks highest in terms of excess mortality rate, followed by Bangladesh and the Philippines, which show similar levels. India and Pakistan fall just below them. As noted earlier, China and Nigeria appear to have effectively controlled TB mortality during the pandemic, with no observed excess deaths.

Adding a temporal lens to the rate-based comparison, Table 2 gives annual excess TB mortality rates (per 100,000 population) for each high-burden country during the COVID-19 period. China and Nigeria stand out as top performers in TB control, consistently reporting negligible excess mortality across all years. In contrast, Indonesia, Bangladesh, and the Philippines exceeded 20 excess TB deaths per 100,000 population, placing them among the countries with the highest mortality burden and weakest pandemic-era TB control. India and Pakistan show similar mortality rates, possibly reflecting shared challenges and strategies given their geographic proximity and health system characteristics. The DRC, despite earlier uncertainty in estimates, shows a relatively low excess mortality rate, indicating a possible recovery in TB program effectiveness during the pandemic.

**Table 1:** Estimated annual and cumulative excess tuberculosis (TB) deaths from 2020 to 2023 in eight high-burden countries—Bangladesh, India, Indonesia, Pakistan, the Philippines, China, Nigeria, and the Democratic Republic of the Congo (DRC)—using an ensemble n-sub-epidemic modeling framework with two overlapping waves. Each panel presents the median excess deaths per year alongside lower and upper 95% uncertainty bounds, as well as the total excess deaths over the four-year period. This visualization highlights the temporal evolution of TB mortality disruptions during the COVID-19 pandemic and underscores the heterogeneity in timing and magnitude of excess deaths across countries

Country	Excess TB mortality (LB,UB)				
	2020	2021	2022	2023	Total
**Bangladesh**	8867 (1368, 15795)	12861 (5239, 19957)	15922 (8021, 23323)	20016 (11665, 27397)	57666 (26294, 86472)
**China**	0 (0, 3905)	0 (0, 3526)	0 (0, 4308)	308 (0, 4726)	308 (0, 16465)
**DRC**	1375 (0, 9498)	2705 (0, 12251)	0 (0, 6106)	0 (0, 9012)	4080 (0, 36869)
**India**	38907 (17120, 60529)	65918 (45172, 87927)	54905 (33748, 77545)	60106 (38709, 83635)	219837 (134748, 309635)
**Indonesia**	14808 (10938, 18675)	48201 (44309, 52402)	55568 (51556, 59874)	44590 (40468, 49307)	163167 (147271, 180258)
**Nigeria**	0 (0, 0)	0 (0, 0)	0 (0, 0)	0 (0, 0)	0 (0, 0)
**Pakistan**	6373 (4000, 8787)	10957 (8543, 13391)	6259 (4005, 8869)	6102 (3678, 8838)	29691 (20225, 39885)
**Philippines**	5963 (4629, 7338)	11316 (9939, 12859)	9069 (7654, 10804)	7881 (6456, 9786)	34229 (28678, 40786)

**Table 2:** Estimated annual and cumulative excess tuberculosis (TB) mortality rates (per 100,000 population) from 2020 to 2023 in eight high-burden countries—Bangladesh, India, Indonesia, Pakistan, the Philippines, China, Nigeria, and the Democratic Republic of the Congo (DRC)—using an ensemble n-sub-epidemic modeling framework with two overlapping waves. Each bar displays the median estimated excess mortality rate for each year, along with lower and upper 95% uncertainty bounds, as well as the total rate for the full pandemic period (2020–2023). This figure highlights the magnitude and variation of TB mortality disruptions across time and space, with countries such as Indonesia, Bangladesh, and the Philippines showing persistently elevated rates, and others like China and Nigeria displaying minimal excess burden

Country	Excess TB mortality rate per 100,000 (LB, UB)				
	2020	2021	2022	2023	Total
**Bangladesh**	5.2 (0.80, 9.2)	7.5 (3.1, 12)	9.3 (4.7, 14)	12 (6.8, 16)	34 (15, 50)
**China**	0.00 (0.00, 0.28)	0.00 (0.00, 0.25)	0.00 (0.00, 0.31)	0.022 (0.00, 0.34)	0.022 (0.00, 1.2)
**DRC**	1.5 (0.00, 11)	3.0 (0.00, 14)	0.00 (0.00, 6.8)	0.00 (0.00, 10)	4.5 (0.00, 41)
**India**	2.7 (1.2, 4.2)	4.6 (3.1, 6.1)	3.8 (2.3, 5.4)	4.2 (2.7, 5.8)	15 (9.4, 22)
**Indonesia**	5.3 (3.9, 6.6)	17 (16, 19)	20 (18, 21)	16 (14, 18)	58 (52, 64)
**Nigeria**	0.00 (0.00, 0.00)	0.00 (0.00, 0.00)	0.00 (0.00, 0.00)	0.00 (0.00, 0.00)	0.00 (0.00, 0.00)
**Pakistan**	2.6 (1.6, 3.6)	4.4 (3.5, 5.4)	2.5 (1.6, 3.6)	2.5 (1.5, 3.6)	12 (8.2, 16)
**Philippines**	5.2 (4.0, 6.4)	9.8 (8.7, 11)	7.9 (6.7, 9.4)	6.9 (5.6, 8.5)	30 (25, 35)

### Global burden visualization

**Fig. 5:**
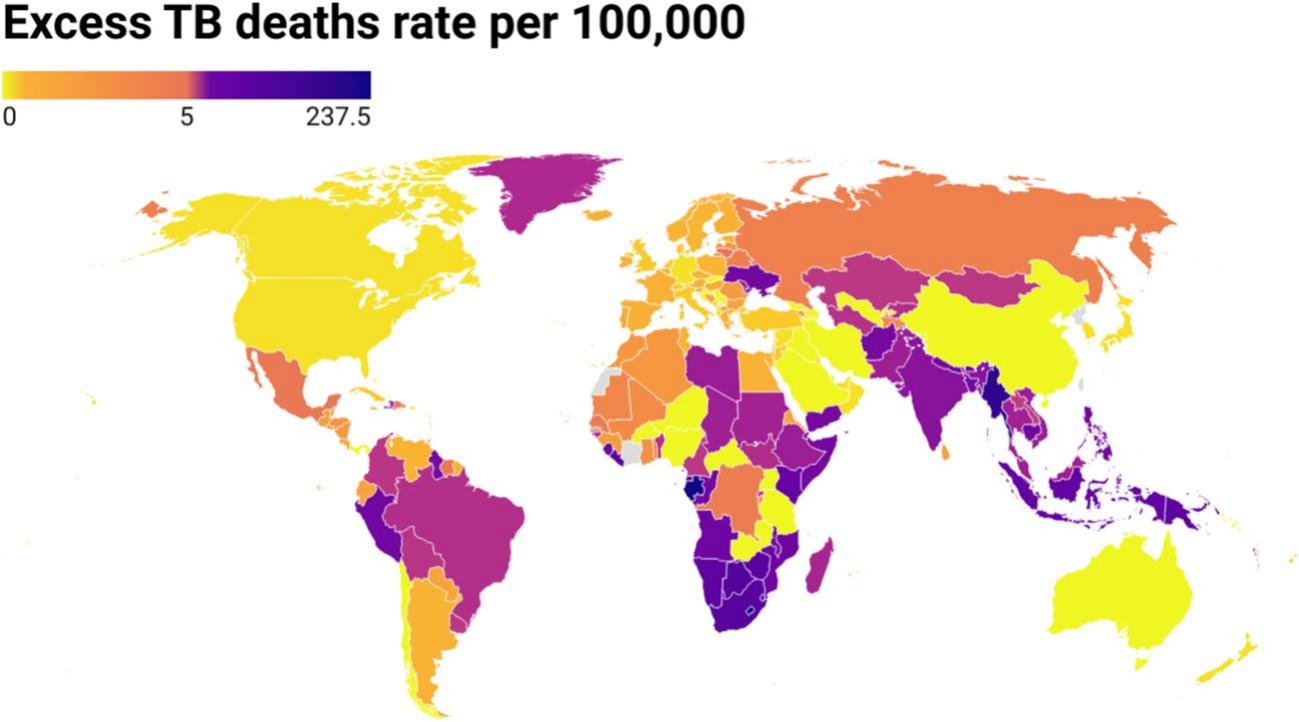
Global map of estimated excess tuberculosis (TB) mortality rates (per 100,000 population) from 2020 to 2023. The map displays country-level estimates derived using an ensemble n-sub-epidemic modeling framework (Ranked 1 model), calibrated to TB mortality data from 2010 to 2019 and forecasted through the COVID-19 pandemic period (2020–2023). Darker shades represent higher excess mortality rates, highlighting geographic variation in TB impact during the pandemic. Countries with no available or reliable data are shown in gray. This visualization emphasizes regions with disproportionately elevated TB mortality burden and supports cross-country comparisons of pandemic-associated disruptions. The map is generated using app.datawrapper.de

Figure 5 offers a global visualization of excess TB mortality rates (per 100,000 population) from 2020 to 2023. The map reveals that Africa contains the highest concentration of countries with elevated TB mortality rates, particularly in Southern Africa, which appears disproportionately affected. Parts of South America—notably Peru—also exhibit high excess deaths rates. In contrast, most high-income countries in North America, Europe, and Australia are shaded yellow, indicating minimal excess TB mortality during the pandemic. The figure underscores the concentration of elevated TB mortality in low- and middle-income countries, reinforcing the role of structural health inequities. Geographical proximity also appears to influence outcomes, as neighboring countries tend to display similar color gradients, probably reflecting shared socioeconomic conditions and regional health system capacities. Overall, the map highlights stark global disparities in the impact of the COVID-19 pandemic on TB mortality.

**Fig. 6:**
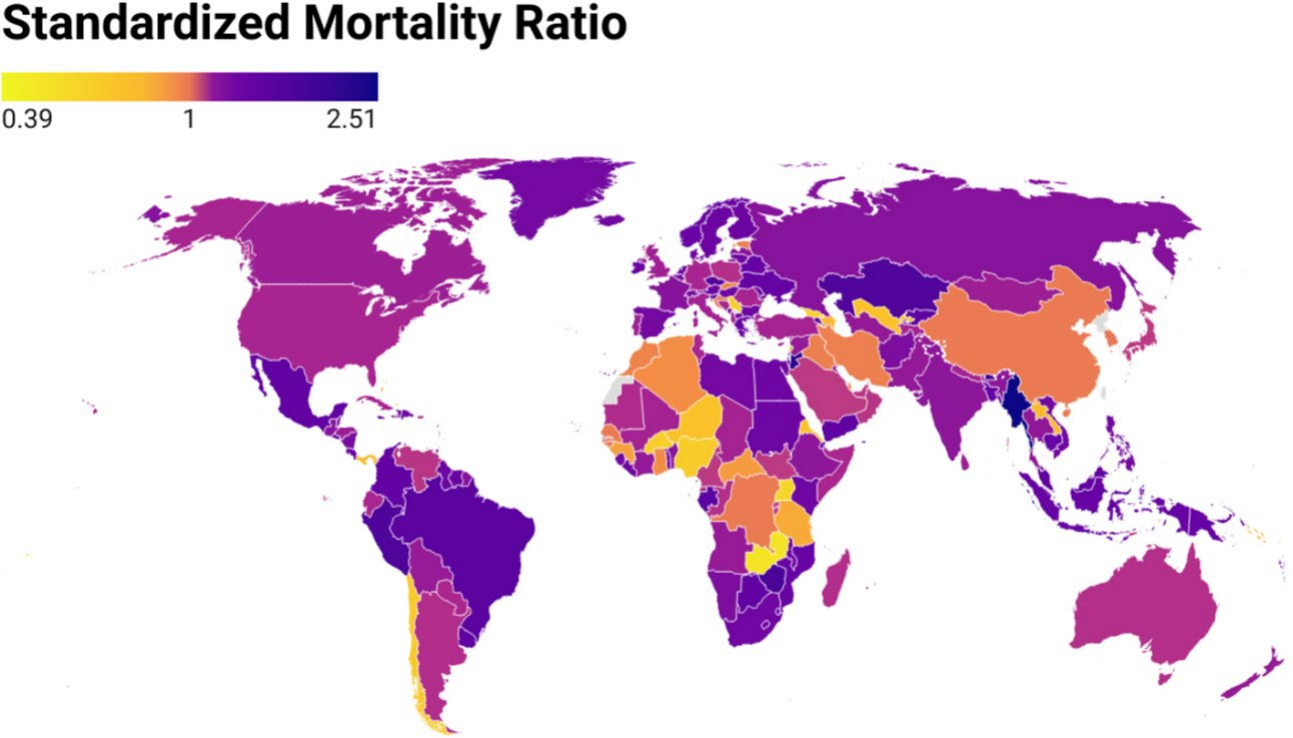
Global map of total standardized mortality ratios (SMR) for tuberculosis (TB) from 2020 to 2023. SMR is defined as the ratio of observed to expected TB deaths, with values greater than 1 indicating excess mortality. Estimates were obtained using the Ranked 1 model in ensemble n-sub-epidemic modeling framework, calibrated to pre-pandemic TB mortality data from 2010 to 2019 and forecasted through the pandemic period (2020–2023). Darker shades represent higher SMR values, indicating greater relative mortality burden, while countries with missing or insufficient data are shown in gray. This figure illustrates cross-national disparities in TB mortality during the COVID-19 pandemic relative to expected baseline trends. The map is generated by app.datawrapper.de

To provide a more standardized basis for international comparison, the SMR is mapped in Fig. 6. Unlike the raw excess mortality rates shown in Fig. 5, the SMR accounts for baseline expectations, offering a more nuanced view of the relative impact of the pandemic on TB mortality across countries. Regions such as Brazil, Argentina, and parts of Africa and Asia exhibit SMR values below 1, indicating that observed TB mortality was lower than expected, likely due to sustained TB care or the presence of mitigating factors. In contrast, many countries in North America, Europe, Russia, and portions of Africa and Asia display SMR values above 1, reflecting excess TB mortality relative to baseline trends. Australia and neighboring regions also fall into this category. As in the previous map, a clear geographical clustering effect is evident, with adjacent countries often showing similar SMR patterns. Taken together, these maps highlight profound global disparities in pandemic-related TB outcomes and emphasize the complex interactions between regional health system capacity, policy responses, and socioeconomic vulnerabilities.

### Correlations with COVID-19 stringency index

**Fig. 7:**
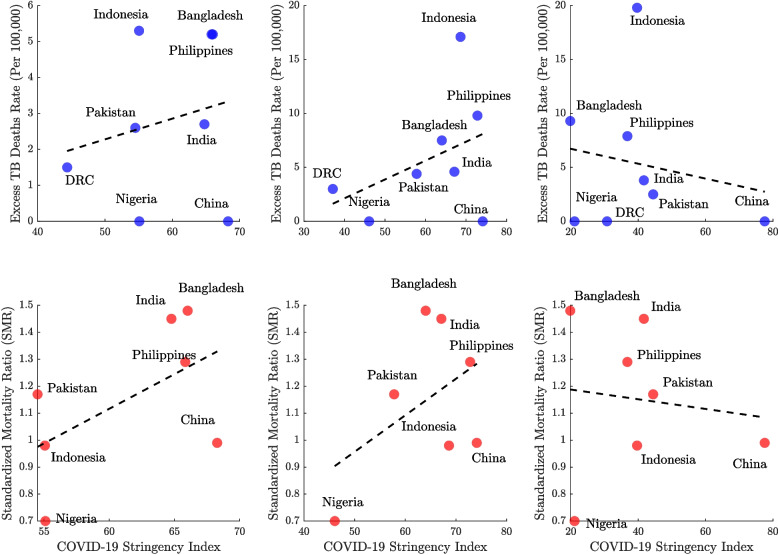
Association between COVID-19 Stringency Index and tuberculosis (TB) mortality outcomes across countries for the years 2020, 2021, and 2022. The top panels display the relationship between the excess TB mortality rate (per 100,000 population) and the Stringency Index, with each country represented by a blue dot. The bottom panels show the association between the Standardized Mortality Ratio (SMR) and the Stringency Index, with countries represented by red dots. Each column corresponds to a single year, allowing for year-over-year comparison of trends. This visualization explores whether stricter COVID-19 control policies were associated with elevated TB mortality and how this relationship evolved over time

Figure 7 illustrates the association between COVID-19 policy stringency and TB mortality outcomes across high-burden countries over three years. In 2020 and 2021 (the first two columns), both the TB mortality rate and the SMR exhibit positive correlations with stringency measures. Indonesia and Bangladesh consistently show high excess TB death rates across all years, whereas China maintains low excess mortality despite moderate-to-high stringency levels (index range: 68–79). Notably, the correlation trends shift in 2022, with a negative association emerging between stringency and TB outcomes. Countries like Nigeria and the DRC maintain low TB mortality throughout the period, while India and Pakistan typically fall in the mid-range for both metrics. The SMR data further show that Bangladesh and India consistently report elevated values above 1.4, indicating substantial excess mortality, whereas Nigeria exhibits the lowest SMR values (0.7), suggesting fewer excess TB deaths than expected. Interestingly, although Indonesia has a high absolute excess TB death rate, its relatively low SMR suggests a pre-existing high baseline burden rather than a dramatic pandemic-era surge.

**Fig. 8:**
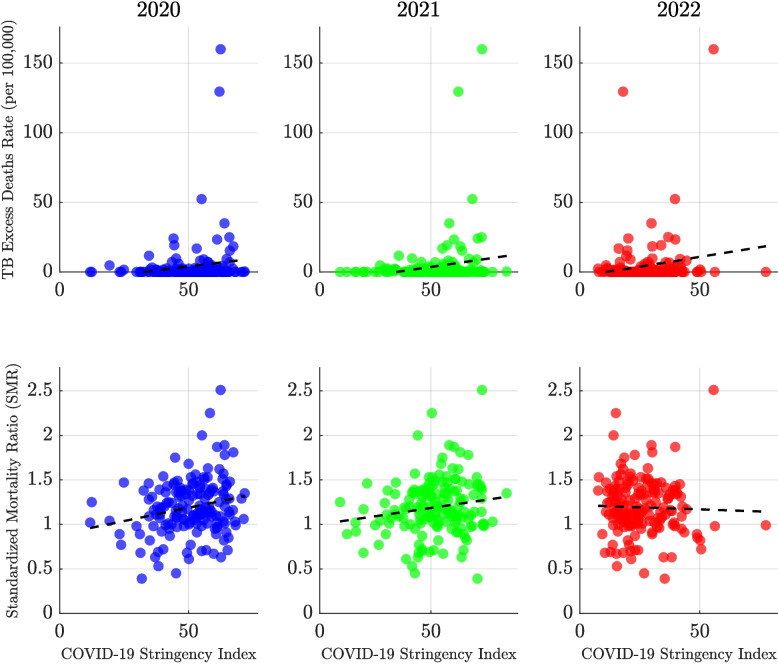
Association between excess tuberculosis (TB) mortality rates and the COVID-19 Stringency Index across all countries globally from 2020 to 2022. Each panel corresponds to a specific year—2020 (blue points), 2021 (green points), and 2022 (red points)—and displays the excess TB death rate per 100,000 population plotted against the country’s average Stringency Index for that year. Correlation coefficients (*r*) for excess mortality rates were 0.15 (95% CI: 0.001 to 0.30, $p = 0.048$) in 2020, 0.19 (95% CI: 0.04 to 0.33, $p = 0.014$) in 2021, and 0.18 (95% CI: 0.03 to 0.32, $p = 0.021$) in 2022. For standardized mortality ratios (SMRs), the corresponding *r* values were 0.22 (95% CI: 0.07 to 0.36, $p = 0.004$) in 2020, 0.17 (95% CI: 0.01 to 0.31, $p = 0.034$) in 2021, and $-0.03$ (95% CI: $-0.19$ to 0.12, $p = 0.672$) in 2022. Each analysis included $n = 165$ countries. These modest correlations suggest that stricter COVID-19 policy responses may have been weakly associated with higher TB mortality, especially in earlier years, while no such association was evident in 2022. The findings highlight changing dynamics as countries adapted their health systems during the pandemic

Expanding the analysis beyond the eight high-burden countries, Fig. 8 explores the global association between COVID-19 policy stringency and TB mortality outcomes over a three-year period. Most countries cluster at relatively low TB excess mortality rates (below 50 per 100,000 population), but several notable outliers exhibit persistently high rates exceeding 20 per 100,000 across all three years. The trend lines indicate how the relationship between policy stringency and TB outcomes evolved over time. In 2020, little correlation is observed, but by 2021 a modest positive association emerges, and by 2022 it becomes more pronounced. The SMR data reveal a more complex and dispersed pattern. In 2020 and 2021, a positive correlation between stringency and SMR is evident, whereas in 2022, the relationship reverses. The wide vertical spread of SMR values in all three years underscores the influence of additional factors beyond policy stringency—with some countries maintaining low SMRs despite strict measures, and others showing high SMRs even under moderate policy regimes.

### Associations with health system strength and socioeconomic development

**Fig. 9:**
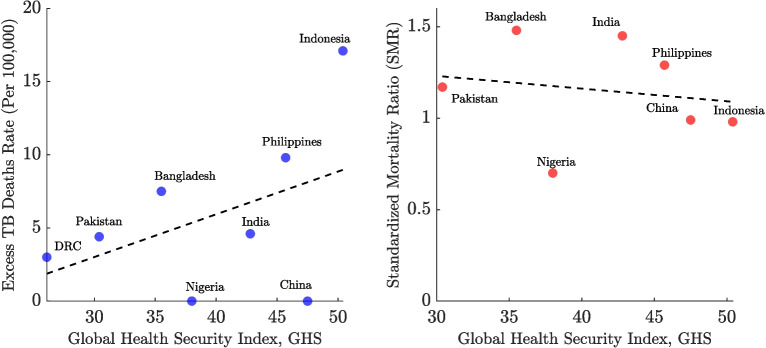
Relationship between tuberculosis (TB) mortality outcomes and the Global Health Security (GHS) Index in 2021 among eight high-burden countries—Bangladesh, India, Indonesia, Pakistan, the Philippines, China, Nigeria, and the Democratic Republic of the Congo (DRC). The left panel plots the excess TB mortality rate (per 100,000 population) against the GHS Index, with each country represented by a blue dot. The right panel shows the Standardized Mortality Ratio (SMR) plotted against the GHS Index, with countries represented by red dots. In both panels, the black dashed line represents the linear trend across these high-burden countries. This figure explores whether higher national health security preparedness was associated with lower TB mortality during the pandemic

Figure 9 explores the relationship between the GHS Index and TB mortality outcomes among high-burden countries. In the left panel, a positive correlation is observed between GHS scores and excess TB mortality rates. Countries such as Indonesia (GHS: 50) experienced higher excess TB mortality (17 per 100,000) compared to countries with lower GHS scores, like the DRC (GHS: 30), which had lower excess mortality (3 per 100,000). The Philippines also shows relatively high TB death rates despite a moderate GHS score, while China and Nigeria appear as outliers—both maintaining low excess mortality despite high GHS scores. In the right panel, which plots SMR against GHS scores, a negative correlation is observed. Countries with higher GHS scores generally experienced lower SMRs. Bangladesh and India exhibit the highest SMRs despite mid-range GHS scores, while Indonesia and China, which have higher GHS scores, show comparatively lower SMRs. Nigeria again stands out with a notably low SMR despite having a relatively modest GHS score.

**Fig. 10:**
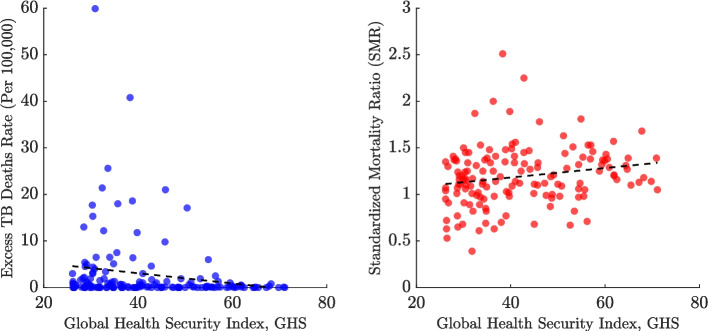
Association between tuberculosis (TB) mortality outcomes and the Global Health Security (GHS) Index across all countries in 2021. The left panel shows the excess TB mortality rate (per 100,000 population) plotted against the GHS Index, with each country represented by a blue dot. The right panel shows the Standardized Mortality Ratio (SMR) versus the GHS Index, with countries represented by red dots. In both panels, the black dashed line represents the linear trend across all countries. Correlation coefficients and statistics are as follows: GHS vs Excess TB Deaths Rate: $r = -0.184$ (95% CI: $-0.334$ to $-0.026$), $p = 0.023$; GHS vs SMR: $r = 0.200$ (95% CI: 0.040 to 0.349), $p = 0.015$

Figure 10 illustrates the global relationship between the GHS Index and TB mortality outcomes, revealing patterns distinct from those observed in the high-burden country subset. In the left panel, the highest excess TB mortality rates are concentrated among countries with low to mid-range GHS scores (approximately 20–40). Several outliers report extremely high excess death rates (40–60 per 100,000) despite modest GHS scores around 30–40. As GHS scores rise above 40, a clear downward trend emerges, with most countries clustering near zero excess deaths as GHS scores approach 60–80. In the right panel, the relationship between SMR and GHS Index is weakly positive, as indicated by the slight upward slope of the dashed trend line. The wide dispersion of data points reflects considerable variation in mortality outcomes not explained by GHS scores alone. Several countries with GHS scores between 30–50 exhibit SMRs above 1.5, while others with similar scores report SMRs below 1. This global analysis highlights more complex and heterogeneous relationships than those observed in the high-burden subset.

**Fig. 11:**
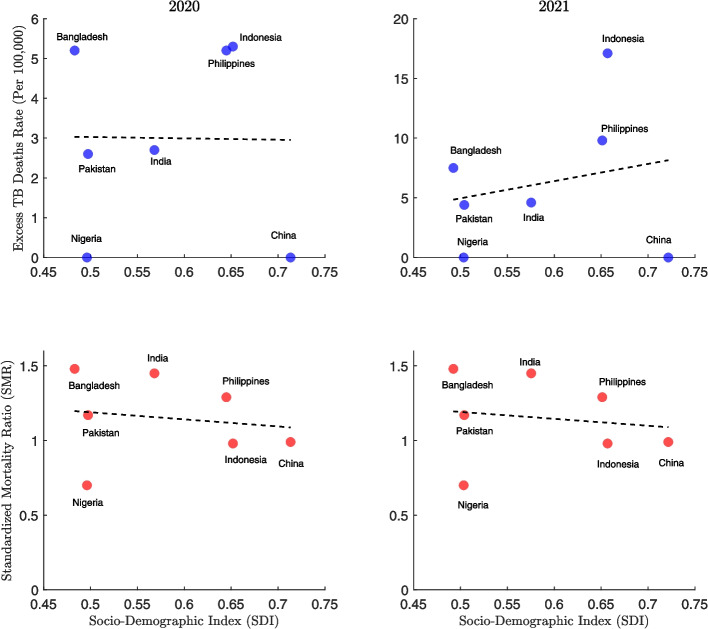
Association between tuberculosis (TB) mortality outcomes and the Socio-demographic Index (SDI) in 2020 and 2021 for eight high-burden countries—Bangladesh, India, Indonesia, Pakistan, the Philippines, China, Nigeria, and the Democratic Republic of the Congo (DRC). The top panels display the relationship between the excess TB mortality rate (per 100,000 population) and SDI, with countries represented by blue dots. The bottom panels show the corresponding relationship between the Standardized Mortality Ratio (SMR) and SDI, with countries depicted by red dots. Each column represents a different year, allowing for comparison of year-over-year shifts. This figure explores how varying levels of socioeconomic development may have influenced the TB mortality burden during the early pandemic years

Figure 11 examines the relationship between the SDI and TB mortality outcomes in high-burden countries for the years 2020 and 2021. In the top panels, Bangladesh and Indonesia stand out with high excess TB mortality rates despite having different SDI levels. By 2021, a positive correlation emerges between SDI and excess TB deaths. For example, Indonesia’s excess TB death rate rises to approximately 17 per 100,000, with an SDI of 0.65. In contrast, China shows consistently low excess TB mortality across both years, with a high SDI value.

The bottom panels plot SMR against SDI and show a consistent negative correlation between these variables over time. Countries with higher SDI generally experienced lower SMRs. India has the highest SMRs (around 1.5), while Nigeria has the lowest SMRs despite its low SDI. The overall pattern remains stable between 2020 and 2021.

**Fig. 12:**
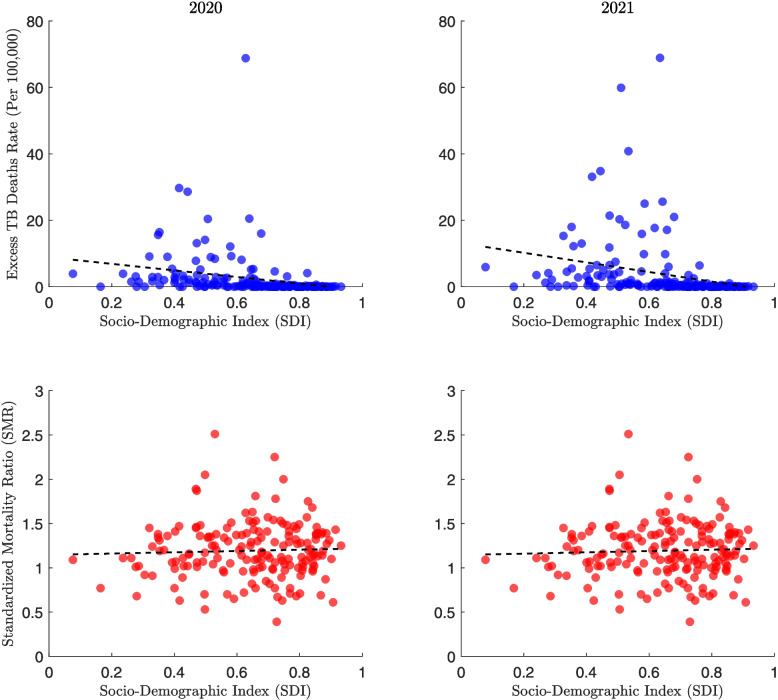
Global association between tuberculosis (TB) mortality outcomes and the Socio-demographic Index (SDI) for the years 2020 and 2021. The top panels show the relationship between the excess TB mortality rate (per 100,000 population) and SDI, with countries represented by blue dots. The bottom panels display the association between the Standardized Mortality Ratio (SMR) and SDI, with countries shown as red dots. Each column corresponds to a specific year, allowing for comparison of trends between 2020 and 2021. Correlation coefficients and statistics are as follows: 2020 (SDI vs Excess Deaths): $r = -0.256$ (95% CI: $-0.389$ to $-0.113$), $p = 0.001$; 2021 (SDI vs Excess Deaths): $r = -0.274$ (95% CI: $-0.405$ to $-0.131$), $p = 0$; 2020 (SDI vs SMR): $r = 0.041$ (95% CI: $-0.108$ to 0.188), $p = 0.591$; 2021 (SDI vs SMR): $r = 0.041$ (95% CI: $-0.108$ to 0.189), $p = 0.587$

Figure 12 presents the global relationship between the SDI and TB mortality outcomes for 2020 and 2021. In the top panels, most countries cluster below 10 excess TB deaths per 100,000 population. A number of outliers, particularly those with SDI values between 0.4 and 0.7, report substantially higher excess TB mortality, with the highest reaching nearly 70 deaths per 100,000 in both years. The trend lines in both years show a slight negative slope.

In the bottom panels, the SMR values span a wide range across SDI levels. Countries with SDI between 0.4 and 0.8 exhibit the broadest spread in SMR, ranging from below 0.5 to above 2.5. The trend lines for both years are similar and show a modest decline in SMR as SDI increases.

## Discussion

Our study provides a comprehensive, country-level assessment of excess TB mortality during the COVID-19 pandemic (2020–2023), revealing substantial geographic heterogeneity driven by differences in health system capacity, pandemic response strategies, and underlying socioeconomic conditions. Using a sub-epidemic modeling framework calibrated to pre-pandemic mortality trends, we estimate approximately 755,876 (95%CI: 591,099 to 965,015) excess TB deaths globally, closely aligning with WHO’s estimate of 700,000 [56]. Our approach enhances previous global estimates by incorporating uncertainty intervals and enabling disaggregated, country-level comparisons. The highest excess TB death rates were observed in southern Africa, South Asia (notably India), and parts of South America, while high-income countries reported minimal impact. These findings underscore the pandemic’s differential impact on TB mortality and emphasize the need to protect TB services during health emergencies.

While the WHO and regional agencies provide estimates for selected areas, our global modeling approach extends beyond these efforts. For example, WHO and European Centre for Disease Prevention and Control estimate approximately 7,000 excess TB deaths in Europe during 2020–2022 [61, 62], whereas we estimate approximately 13,551 (95%CI: 9,277 to 17,904) deaths for the same period. A large share of this excess was concentrated in Russia and Ukraine, where TB control efforts may have been further disrupted by armed conflict and the 2022 invasion.

In a separate study [63], about 400,000 excess TB deaths were estimated in four high-burden countries (Pakistan, India, Indonesia, and Kenya). Our broader estimation framework yields an estimate of 338,026 (95%CI: 219,663 to 460,551) for these countries, which includes the earlier estimate within its confidence interval.

Our global map of excess TB mortality per 100,000 population reveals stark regional disparities. Countries in southern Africa, South Asia, and South America (e.g., Peru) experienced the highest rates, while regions like North America, Europe, and Australia had minimal excess mortality. Spatial patterns such as the presence of similar mortality rates in neighboring countries such as India and Pakistan suggest shared vulnerabilities and constraints of the healthcare infrastructure.

One striking finding is the near-zero upper bound for excess TB deaths in Nigeria, which may reflect substantial underreporting of both TB and COVID-19 deaths. Nigeria’s relatively low number of reported COVID-19 cases, combined with known weaknesses in disease surveillance systems [75], suggests major gaps in mortality reporting during the pandemic.

In settings such as Indonesia and the DRC, wide uncertainty intervals point to the challenges of estimating excess mortality where data completeness is limited. In the DRC, the large upper bound suggests substantial underestimation of TB deaths is possible.

Analysis of policy stringency during the pandemic revealed no consistent immediate relationship with TB mortality. While 2020 showed limited correlation, a modest positive association emerged in 2021, which may partly reflect disruptions to TB services. However, it is important to note that countries facing higher risks from COVID-19 or TB might have implemented stricter policies as protective measures, complicating causal interpretations. By 2022, the relationship weakened, likely reflecting both the adaptation of health systems over time and the increased availability of COVID-19 vaccines. These patterns suggest that stringent COVID-19 measures could have unintended effects on non-COVID conditions like TB, particularly where mitigation strategies are lacking.

When evaluating health system capacity, no consistent global relationship was observed between the GHS Index and excess TB mortality. Among high-burden countries, a surprising negative correlation emerged, with higher GHS scores associated with increased excess deaths. This unexpected pattern may reflect complex dynamics such as the diversion of resources from TB programs to COVID-19 response efforts. Notably, countries like China and Nigeria appeared as outliers, maintaining low excess mortality despite high GHS scores, which suggests that programmatic resilience, pre-existing health infrastructure, and demographic factors likely play significant protective roles [66, 76]. At the global level, stronger health security systems were generally linked to lower excess TB mortality, particularly as GHS scores rose above 40. However, this association did not extend clearly to the SMR, where the relationship was weakly positive and highly dispersed. This heterogeneity indicates that factors beyond health system capacity, such as data quality, co-infection dynamics, or resource allocation strategies, also shaped TB outcomes during the pandemic. These findings highlight the complex and context-dependent nature of pandemic impacts on TB mortality, underscoring the need for integrated approaches that strengthen routine TB services alongside emergency preparedness.

The relationship between excess TB mortality and the SDI also proved complex. Globally, higher SDI is associated with lower TB mortality, as reflected by a consistent negative correlation between SDI and SMR in high-burden countries across 2020 and 2021. However, in 2021, a positive correlation emerged between SDI and excess TB deaths among these countries. This pattern suggests that higher socioeconomic development did not necessarily confer protection against excess TB mortality during the pandemic. One possible explanation is that countries with higher SDI may have redirected resources toward COVID-19 response efforts, inadvertently disrupting TB services. In contrast, some lower-SDI countries may have maintained continuity in TB care despite facing broader structural challenges. At the global level, a modest decline in excess TB deaths and SMR with increasing SDI was observed, indicating that socioeconomic development was broadly associated with lower TB mortality, though with considerable variation across countries.

The indirect effects of the pandemic on TB mortality may persist in the coming years. Our results highlight the need for balanced public health strategies that preserve essential services for endemic diseases during crises. Regional disparities in excess TB mortality reflect systemic inequalities in healthcare delivery, surveillance, and preparedness, which must be addressed through targeted investments.

One limitation of this study is the model selection approach. We used AICc to compare in-sample fits, which helps avoid overly simple or complex models but does not assess out-of-sample predictive accuracy. Ideally, validation with a separate dataset would allow evaluation of absolute error and prediction interval coverage. Due to data limitations, we could not perform such validation, which may reduce generalizability. Future studies with larger datasets should apply cross-validation or holdout testing to better assess predictive performance.

Static 2021 population data were used, which may not capture population declines caused by COVID-19. As a result, excess TB death rate estimates could potentially be inflated. Future work should incorporate updated demographic data and age-standardized analyses to improve accuracy and comparability.

An important limitation of our analysis concerns the treatment of negative excess TB deaths. While such values can arise from the modeling process, interpreting them as true reductions in mortality due to the pandemic is challenging and requires strong contextual justification. Many of these negative estimates may reflect surveillance disruptions or underreporting, rather than genuine declines in TB mortality. To avoid misinterpretation, we chose to truncate negative values; however, future work could explore alternative approaches that retain these values and explicitly compare the results against relevant indices such as the SDI, GHS, and stringency scores. Such comparisons may offer new insights but would require careful interpretation to avoid drawing spurious conclusions. Additionally, we assumed temporal independence across years when summarizing excess mortality. While this may appear simplistic, it reflects the distinct dynamics of each pandemic year—such as the emergency response phase in 2020, vaccine rollout in 2021, transition in 2022, and early recovery in 2023, which likely introduced year-specific patterns in TB service disruptions. Nonetheless, this assumption introduces some uncertainty, and future studies may consider more sophisticated time-series approaches to account for potential inter-year dependencies. To address limitations related to truncation and temporal independence, we also report SMR alongside excess deaths rate. SMRs, which do not rely on truncation and are less sensitive to certain modeling assumptions, provide an important complementary measure to enhance the robustness of our findings.

In our study, we examined how often reported data fell within the forecast intervals across calibration and forecast years, which provided some empirical reassurance of model adequacy. A limitation, however, was relying on visual assessment of uncertainty intervals rather than systematic coverage probability evaluation. While this pragmatic approach produced results consistent with prior studies, it does not formally assess how well prediction intervals capture the true data. A more rigorous alternative would be to evaluate coverage probability at multiple confidence levels (e.g., $50\%$, $80\%$, $95\%$) during the calibration phase, which would allow for more robust error structure selection across countries. Although implementing this systematically at the global scale is computationally demanding, we acknowledge it as an important methodological refinement.

Our estimates avoid the major pitfalls highlighted in recent reviews of ad hoc demographic approaches such as the growth rate discontinuity method (GRDM) [77]. Specifically, we do not depend on intercensal growth projections or assumptions about census completeness. Instead, our reliance on continuous mortality registration data and validated excess mortality modeling techniques reduces susceptibility to biases from fertility or migration shocks and allows for transparent uncertainty quantification. This positions our study as a more robust and reproducible alternative to GRDM-based analyses.

Data quality and completeness are important considerations when interpreting model results. In particular, countries such as Nigeria have been identified as outliers due to questionable data reliability. Factors contributing to potential bias include disruptions caused by COVID-19 lockdowns, which limited the capacity of TB programs to diagnose and report cases accurately, likely leading to underestimation of TB mortality. Additionally, challenges such as underreporting, misclassification of causes of death, variations in health system capacity, and delays in data reporting may further affect the accuracy of estimates. These limitations highlight the need for cautious interpretation and underscore the importance of improving surveillance and data collection systems.

In summary, this study highlights the uneven global impact of COVID-19 on TB mortality, demonstrating how pre-existing health system vulnerabilities and policy trade-offs shaped pandemic outcomes. Despite past progress, many countries experienced setbacks in TB control. Our findings reinforce the need for adaptive, integrated pandemic responses that safeguard continuity in essential health services. Future research should monitor post-pandemic TB trends and evaluate sustainable strategies for strengthening health system resilience without compromising endemic disease control.

## Supplementary Information

Additional file 1. The supplementary material includes figures and tables presenting excess TB deaths for all countries individually. This additional content provides a detailed breakdown of the global data referenced in the main text

## Data Availability

All data used in this study are publicly available and can be accessed through the provided online sources.
