# Supplementary material for: Global excess tuberculosis mortality during COVID-19: a country-level modeling study of policy and development correlates
Source: BMC Public Health. 2025 Oct 31;25:3723. doi: 10.1186/s12889-025-24858-8 (PMC12577229; doi:10.1186/s12889-025-24858-8)
Supplement: Supplementary file 1 — Additional file 1. The supplementary material includes figures and tables presenting excess TB deaths for all countries individually. This additional content provides a detailed breakdown of the global data referenced in the main text [file 12889_2025_24858_MOESM1_ESM.pdf]

# Supplementary Materials

Hamed Karami*[1], Svenn-Erik Mamelund†[2], Alexandra Smirnova‡[1], and Gerardo Chowell§[3]

[1]Department of Mathematics & Statistics, Georgia State University, Atlanta, 30303, Georgia, USA
[2]Centre for Research on Pandemics & Society (PANSOC), Oslo Metropolitan University, Oslo, 0130, Norway
[3]Department of Population Health Sciences, Georgia State University, Atlanta, 30303, Georgia, USA

*hkarami1@student.gsu.edu
†masv@oslomet.no
‡asmirnova@gsu.edu
§gchowell@gsu.edu

# S1 Southeast Asia

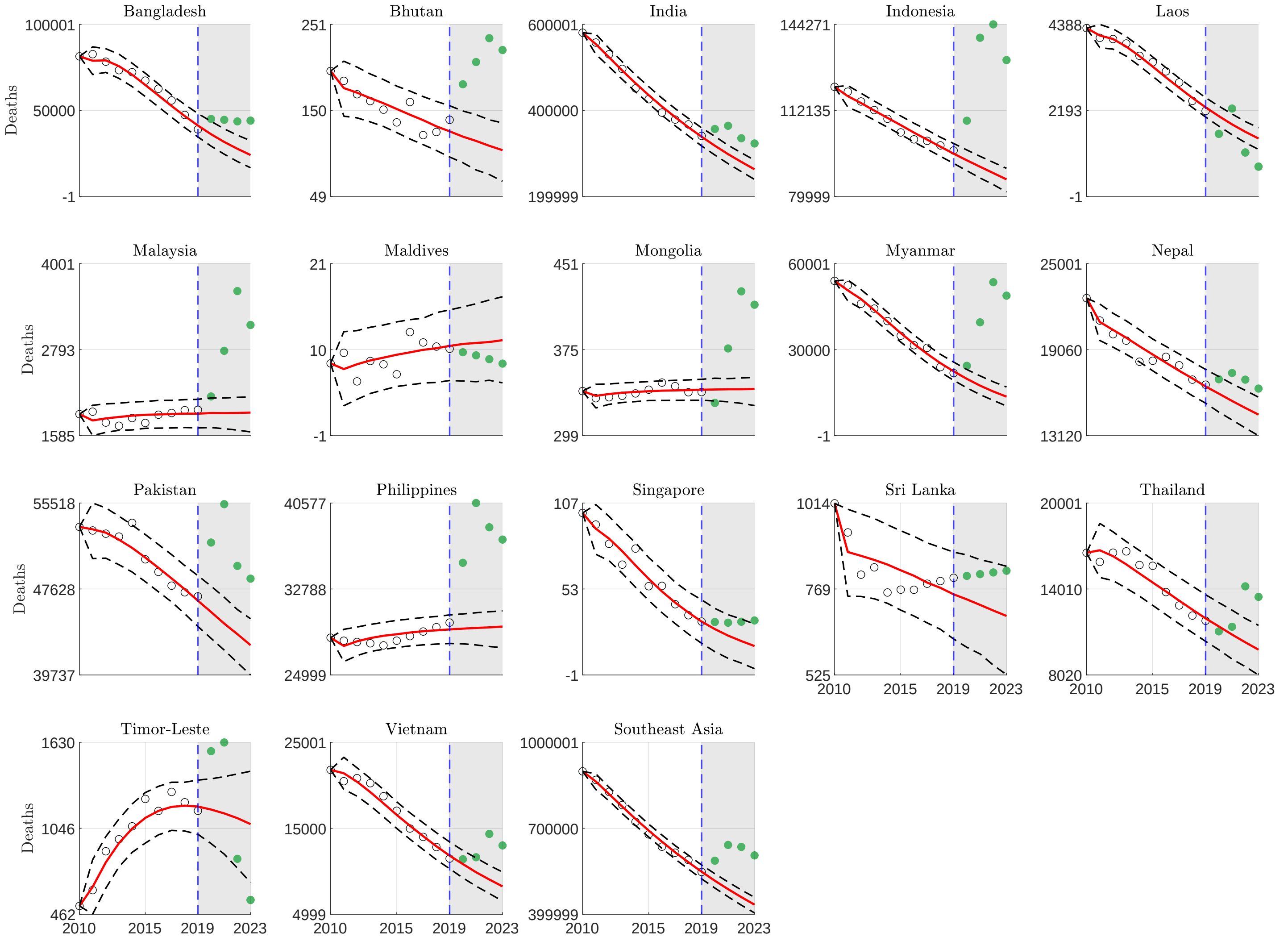


Figure S1: Forecasting of annual tuberculosis (TB) deaths in Southeast Asia from 2020 to 2023 using a subepidemic ensemble modeling framework. The model was calibrated to reported mortality data from 2010 to 2019 and used to generate forecasts for the pandemic period (2020–2023), with the Ranked 1 model selected based on best fit criteria. The red curve represents the median forecast, and black dashed lines indicate the 95% prediction interval (PI). Reported mortality data are shown as circles: black circles represent the calibration period, and green-filled circles represent actual data points during the forecast period.

## S1.1 Excess mortality

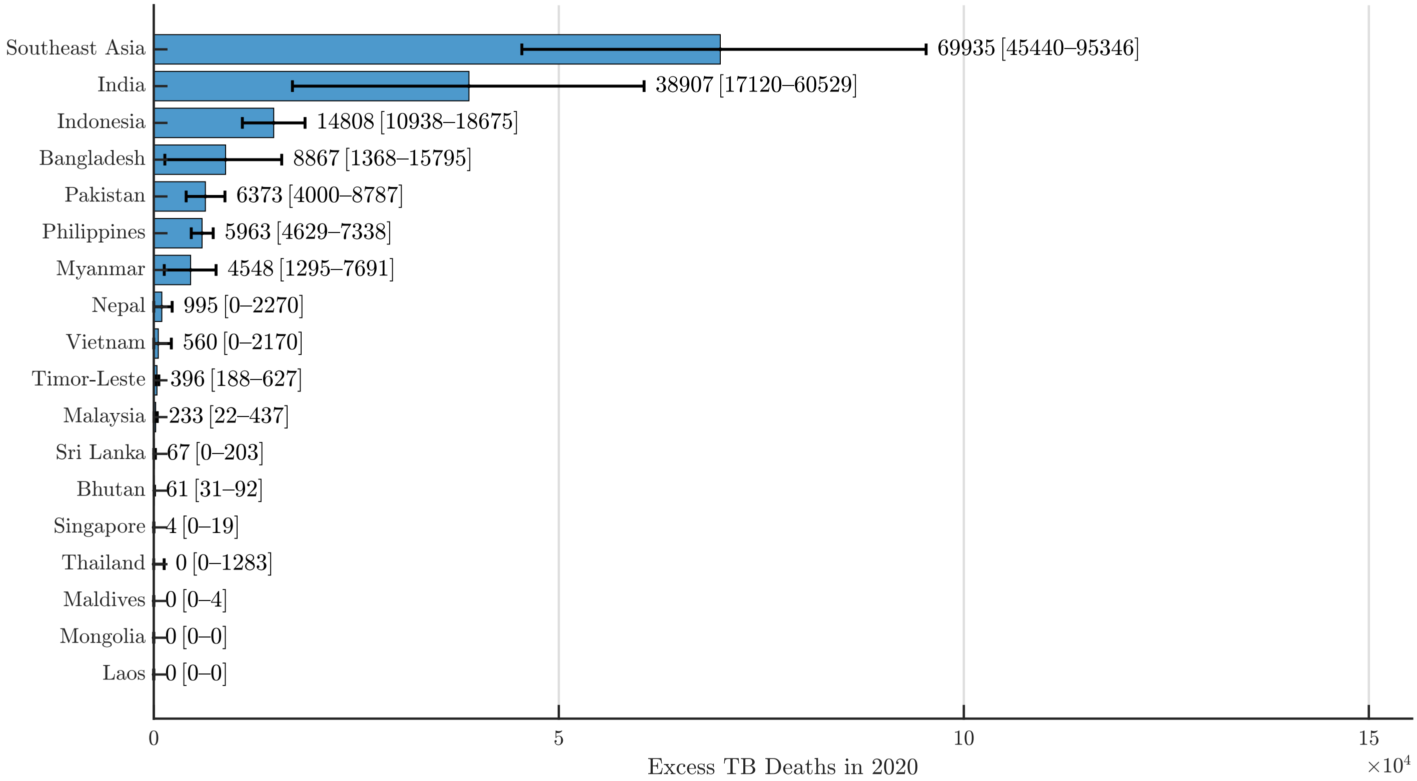


Figure S2: Estimated excess tuberculosis (TB) deaths in Southeast Asian countries in 2020 using an ensemble subepidemic modeling framework with two overlapping waves. The model was calibrated to WHO-reported TB mortality data from 2010 to 2019 and forecasted expected deaths for 2020. Horizontal bars represent the median excess deaths for each country, and horizontal lines denote the corresponding 95% uncertainty intervals. Exact numerical values are labeled adjacent to each bar for ease of interpretation. This figure highlights country-level variation in excess TB mortality during the first year of the COVID-19 pandemic across the Southeast Asia region.

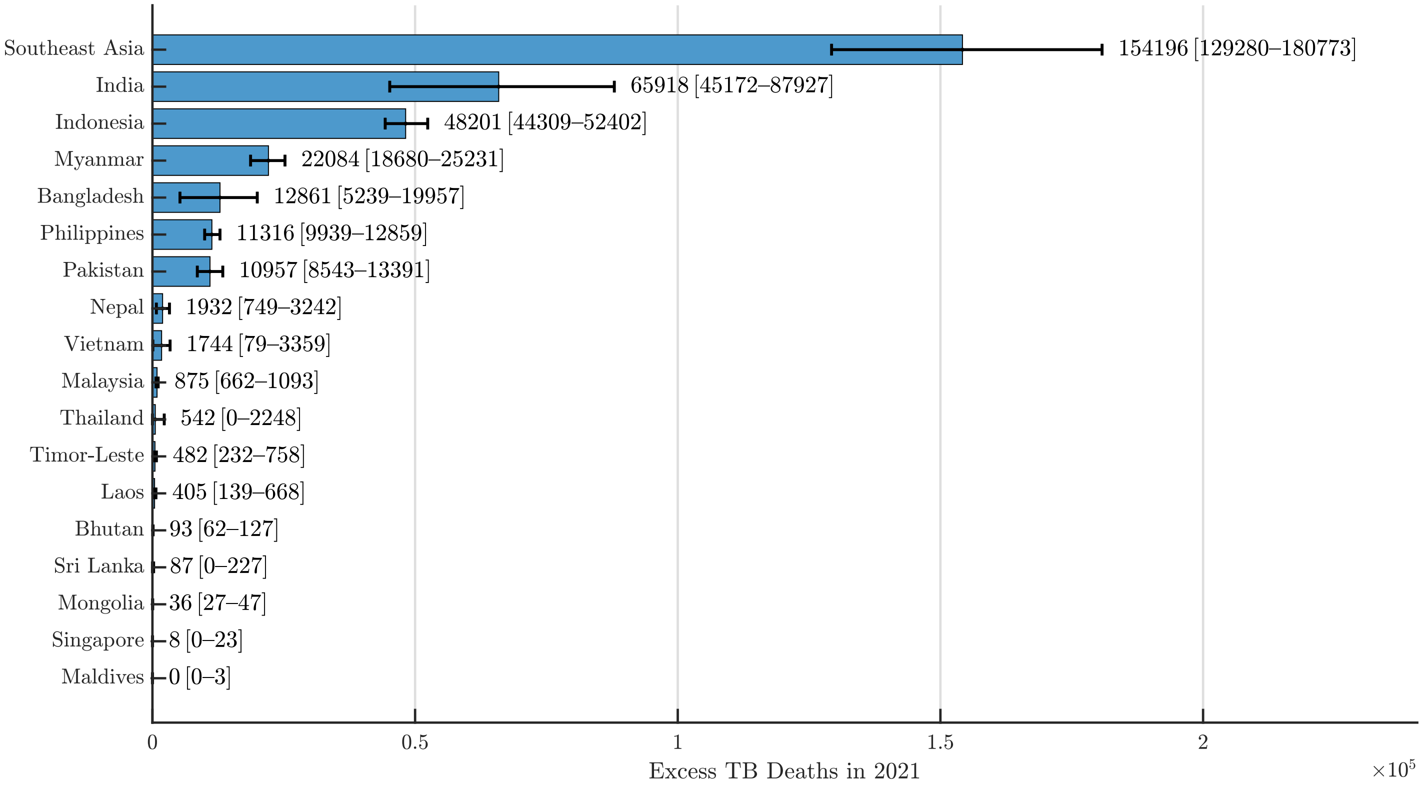


Figure S3: Estimated excess tuberculosis (TB) deaths in Southeast Asian countries in 2021 using an ensemble subepidemic modeling framework with two overlapping waves. The model was calibrated to pre-pandemic TB mortality data from 2010 to 2019 and forecasted expected mortality for 2021. Horizontal bars indicate the median estimated excess deaths for each country, with horizontal lines showing the 95% uncertainty intervals (lower and upper bounds). Exact mortality estimates are labeled next to each bar to support country-level comparisons. This figure illustrates the second-year impact of the COVID-19 pandemic on TB mortality in the region, highlighting variability in excess deaths across national contexts.

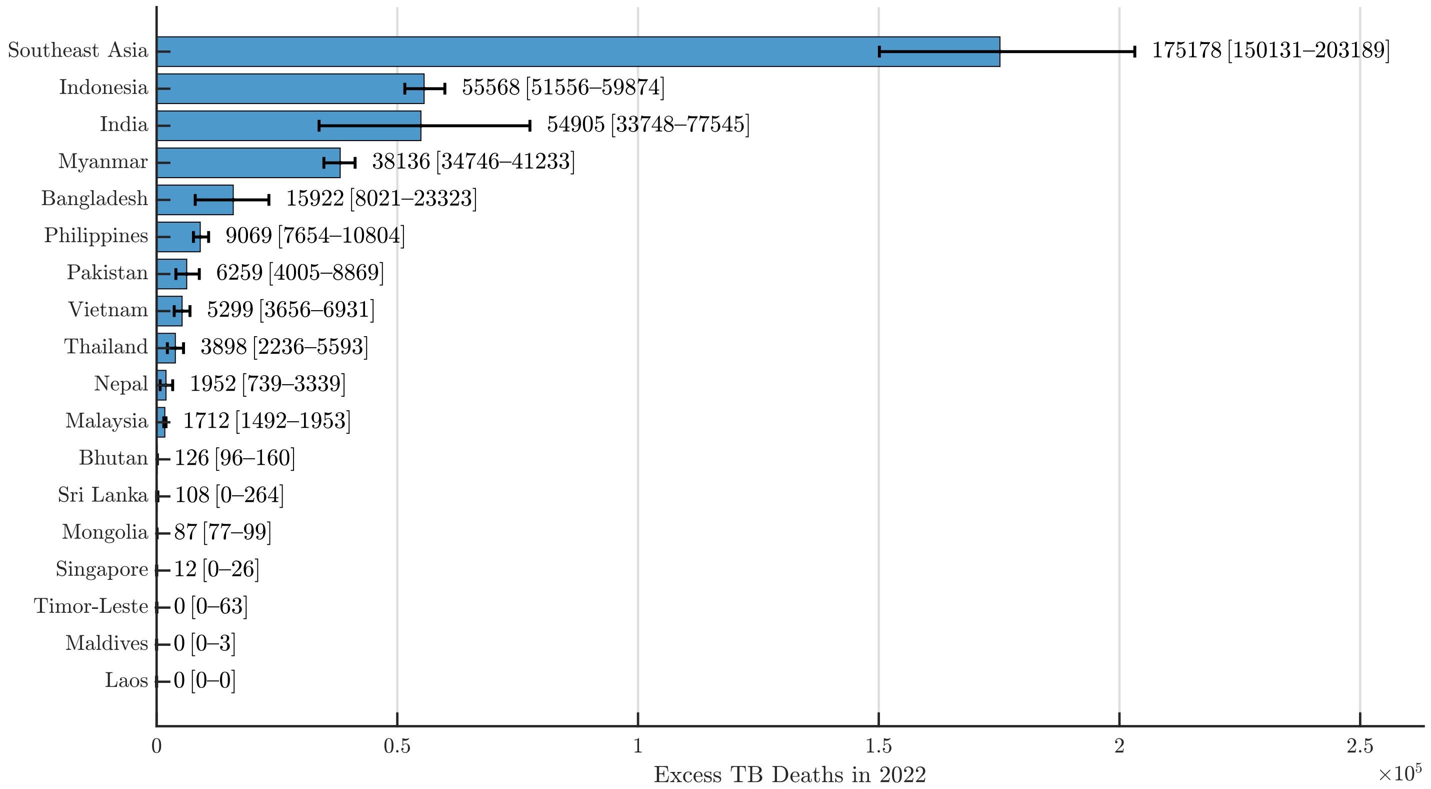


Figure S4: Estimated excess tuberculosis (TB) deaths in Southeast Asian countries in 2022 using an ensemble subepidemic modeling framework with two overlapping waves. The model was calibrated to TB mortality data from 2010 to 2019 and forecasted expected mortality for the pandemic period. Horizontal bars indicate the median estimated excess deaths per country, and horizontal lines represent the 95% uncertainty intervals (lower and upper bounds). Exact values are annotated next to each bar for direct comparison. This figure reflects the continued impact of the COVID-19 pandemic on TB mortality during its third year, revealing ongoing disparities in excess deaths across the region.

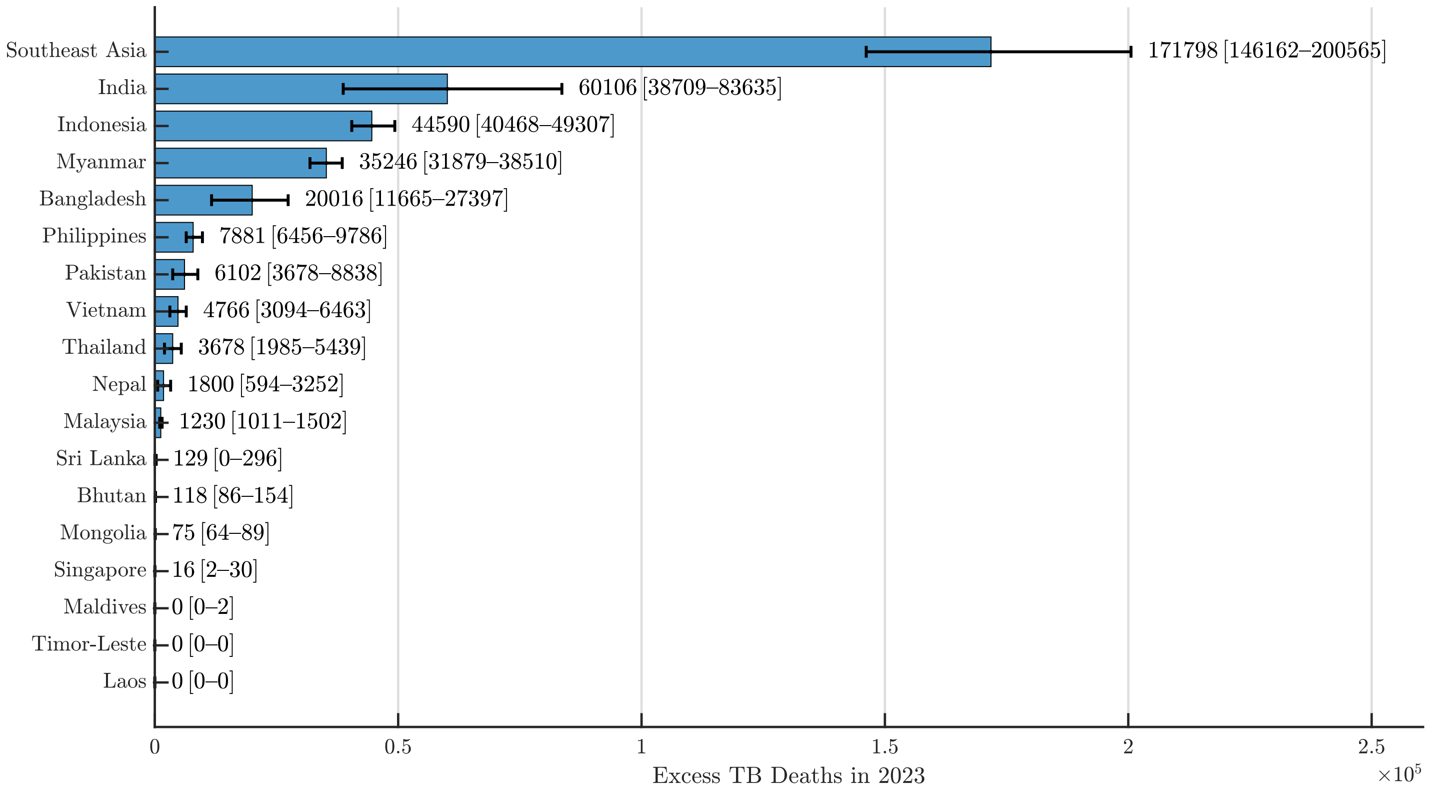


Figure S5: Estimated excess tuberculosis (TB) deaths in Southeast Asian countries in 2023 using an ensemble subepidemic modeling framework with two overlapping waves. The model was calibrated using TB mortality data from 2010 to 2019 and forecasted expected mortality through the end of the COVID-19 pandemic period. Horizontal bars indicate the median estimated excess deaths for each country, and horizontal lines represent the 95% uncertainty intervals. Exact excess mortality values are labeled adjacent to each bar. This figure captures the residual effects of the pandemic on TB mortality in 2023 and highlights persistent differences in excess mortality burden across the region.

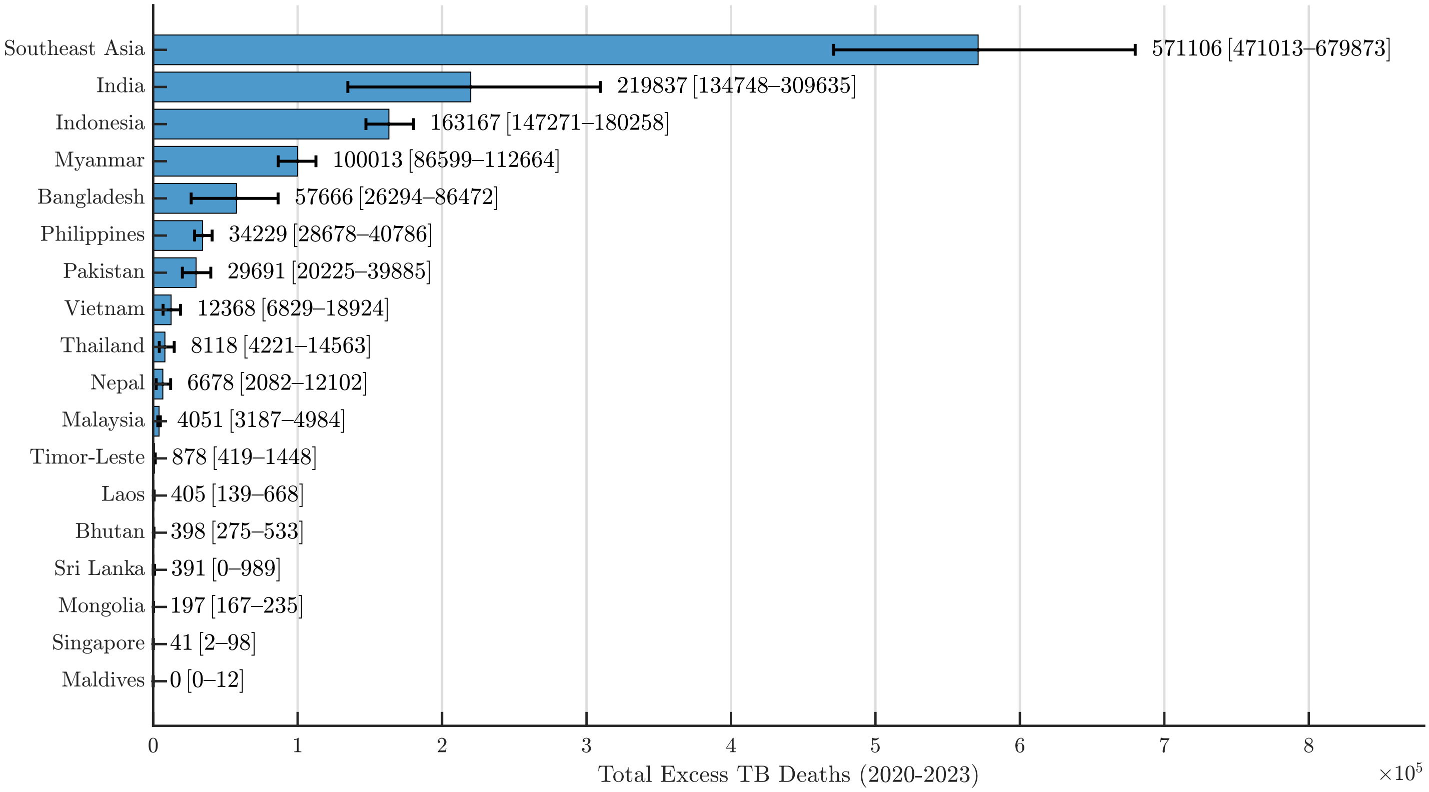


Figure S6: Total estimated excess tuberculosis (TB) deaths from 2020 to 2023 in Southeast Asian countries, based on an ensemble subepidemic modeling framework with two overlapping waves. The model was calibrated to pre-pandemic TB mortality data from 2010 to 2019 and used to forecast expected mortality for the four-year pandemic period. Horizontal bars represent the median cumulative excess deaths for each country, with horizontal lines indicating the 95% uncertainty intervals (lower and upper bounds). Exact cumulative excess mortality values are labeled adjacent to each bar. This figure summarizes the overall impact of the COVID-19 pandemic on TB mortality across the region, highlighting countries with the greatest sustained disruptions.

| | Excess TB mortality (LB,UB) | | | | |
|---|---|---|---|---|---|
| Country | **2020** | **2021** | **2022** | **2023** | **Total** |
| **Bangladesh** | 8867 (1368, 15795) | 12861 (5239, 19957) | 15922 (8021, 23323) | 20016 (11665, 27397) | 57666 (26294, 86472) |
| **Bhutan** | 61 (31, 92) | 93 (62, 127) | 126 (96, 160) | 118 (86, 154) | 398 (275, 533) |
| **India** | 38907 (17120, 60529) | 65918 (45172, 87927) | 54905 (33748, 77545) | 60106 (38709, 83635) | 219837 (134748, 309635) |
| **Indonesia** | 14808 (10938, 18675) | 48201 (44309, 52402) | 55568 (51556, 59874) | 44590 (40468, 49307) | 163167 (147271, 180258) |
| **Laos** | 0 (0, 0) | 405 (139, 668) | 0 (0, 0) | 0 (0, 0) | 405 (139, 668) |
| **Malaysia** | 233 (22, 437) | 875 (662, 1093) | 1712 (1492, 1953) | 1230 (1011, 1502) | 4051 (3187, 4984) |
| **Maldives** | 0 (0, 4) | 0 (0, 3) | 0 (0, 3) | 0 (0, 2) | 0 (0, 12) |
| **Mongolia** | 0 (0, 0) | 36 (27, 47) | 87 (77, 99) | 75 (64, 89) | 197 (167, 235) |
| **Myanmar** | 4548 (1295, 7691) | 22084 (18680, 25231) | 38136 (34746, 41233) | 35246 (31879, 38510) | 100013 (86599, 112664) |
| **Nepal** | 995 (0, 2270) | 1932 (749, 3242) | 1952 (739, 3339) | 1800 (594, 3252) | 6678 (2082, 12102) |
| **Pakistan** | 6373 (4000, 8787) | 10957 (8543, 13391) | 6259 (4005, 8869) | 6102 (3678, 8838) | 29691 (20225, 39885) |
| **Philippines** | 5963 (4629, 7338) | 11316 (9939, 12859) | 9069 (7654, 10804) | 7881 (6456, 9786) | 34229 (28678, 40786) |
| **Singapore** | 4 (0, 19) | 8 (0, 23) | 12 (0, 26) | 16 (2, 30) | 41 (2, 98) |
| **Sri Lanka** | 67 (0, 203) | 87 (0, 227) | 108 (0, 264) | 129 (0, 296) | 391 (0, 989) |
| **Thailand** | 0 (0, 1283) | 542 (0, 2248) | 3898 (2236, 5593) | 3678 (1985, 5439) | 8118 (4221, 14563) |
| **Timor-Leste** | 396 (188, 627) | 482 (232, 758) | 0 (0, 63) | 0 (0, 0) | 878 (419, 1448) |
| **Vietnam** | 560 (0, 2170) | 1744 (79, 3359) | 5299 (3656, 6931) | 4766 (3094, 6463) | 12368 (6829, 18924) |
| **Southeast Asia** | 69935 (45440, 95346) | 154196 (129280, 180773) | 175178 (150131, 203189) | 171798 (146162, 200565) | 571106 (471013, 679873) |

Table S1: Estimated excess tuberculosis (TB) deaths in Southeast Asian countries from 2020 to 2023, presented both by individual year and as a cumulative total, using an ensemble subepidemic modeling framework with two overlapping waves. The model was calibrated to TB mortality data from 2010 to 2019 and forecasted expected deaths for each pandemic year. Bars represent median excess mortality estimates for each year and for the total period, with horizontal lines denoting the 95% uncertainty intervals (lower and upper bounds). This figure enables comparison of the temporal evolution and cumulative burden of excess TB deaths across countries in the region, highlighting differential impacts throughout the pandemic.

## S1.2 Excess mortality rate

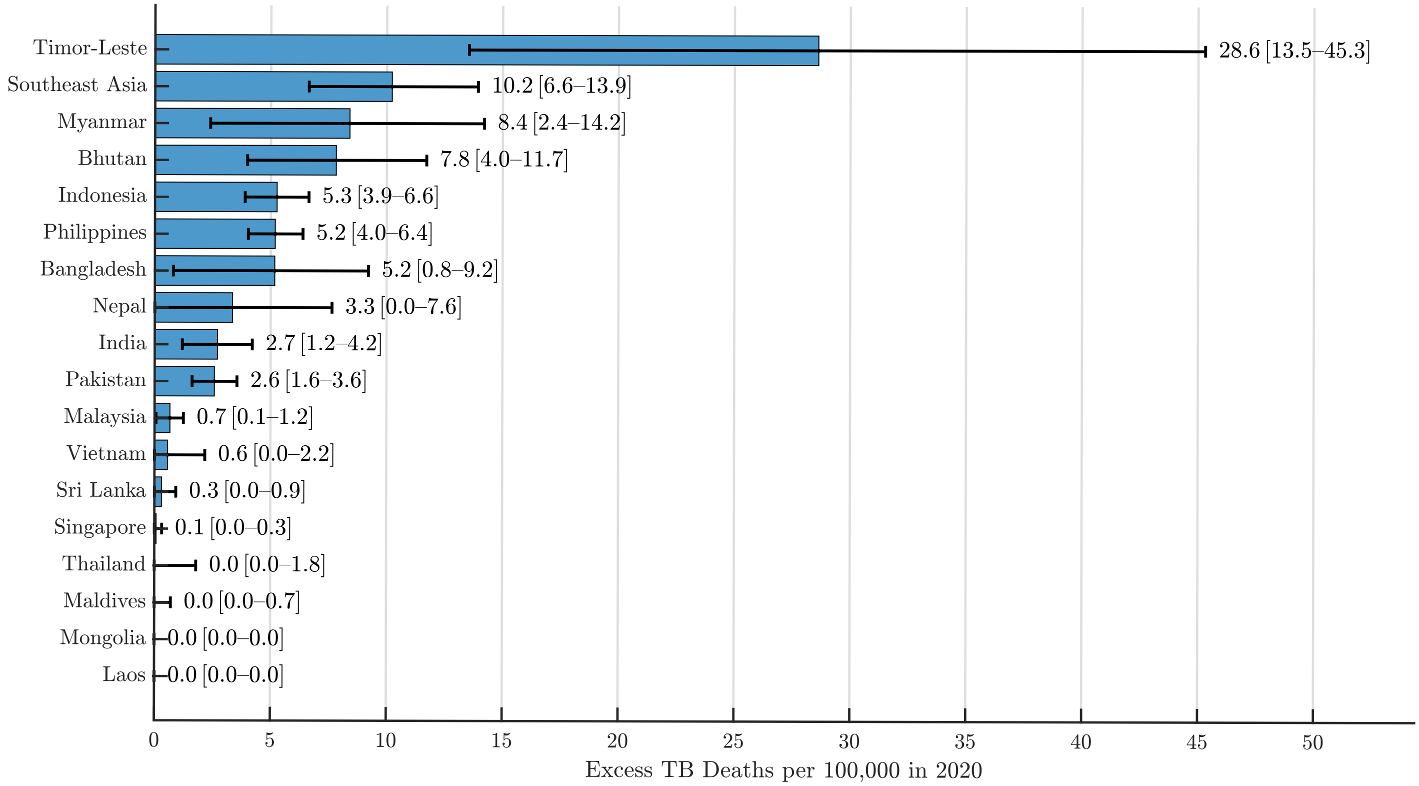


Figure S7: Estimated excess tuberculosis (TB) mortality rates (per 100,000 population) in Southeast Asian countries in 2020, using an ensemble subepidemic modeling framework with two overlapping waves. The model was calibrated to TB mortality data from 2010 to 2019 and used to estimate expected deaths for the first year of the COVID-19 pandemic. Horizontal bars display the median excess mortality rate per country, with horizontal lines representing the 95% uncertainty intervals (lower and upper bounds). Exact rate values are annotated next to each bar to facilitate comparison. This figure highlights the country-specific impact of pandemic-related disruptions on TB mortality during 2020.

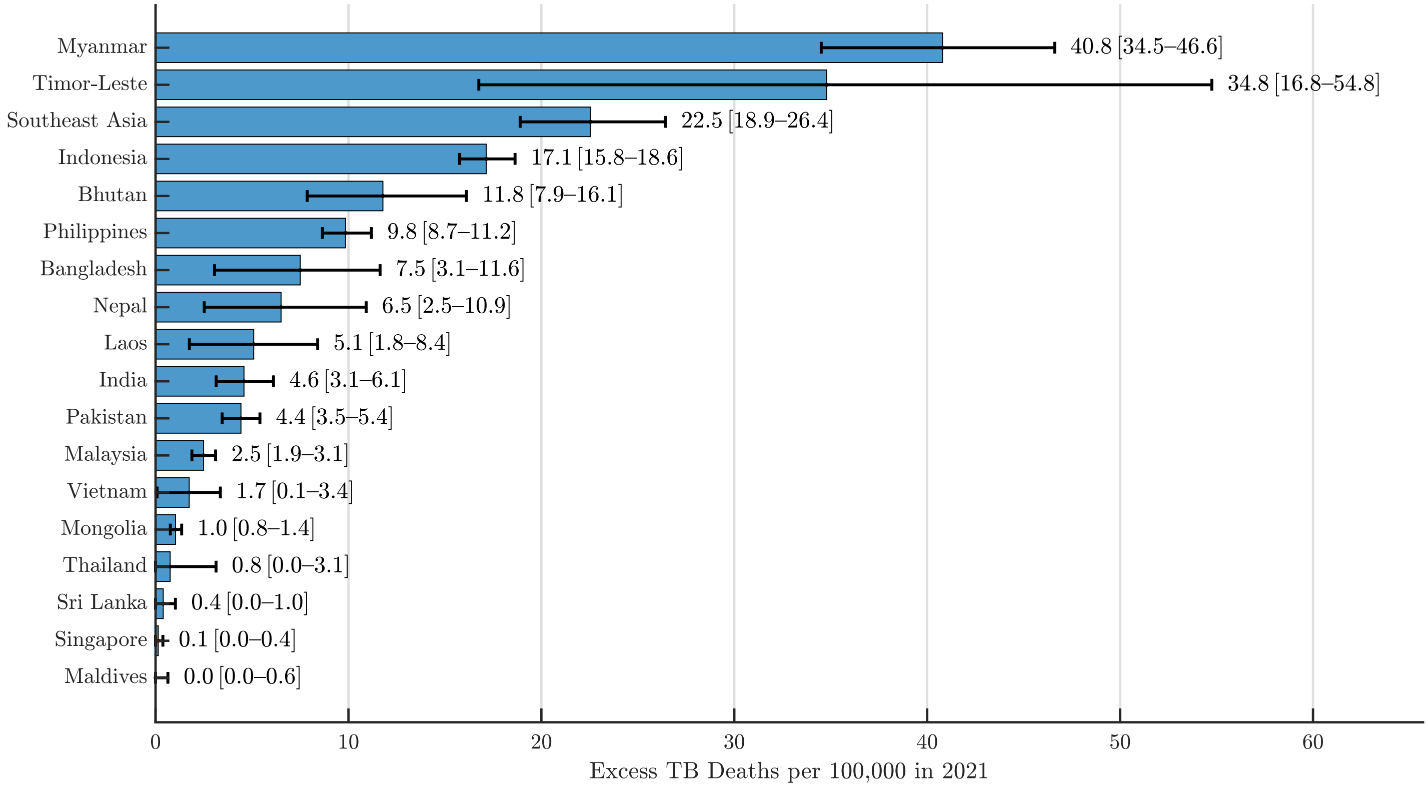


Figure S8: Estimated excess tuberculosis (TB) mortality rates (per 100,000 population) in Southeast Asian countries in 2021 using an ensemble subepidemic modeling framework with two overlapping waves. The model was calibrated to TB mortality data from 2010 to 2019 and used to forecast expected mortality for the second year of the COVID-19 pandemic. Horizontal bars show the median excess mortality rate for each country, and horizontal lines indicate the 95% uncertainty intervals (lower and upper bounds). Exact rate estimates are labeled next to each bar to support direct comparison. This figure captures the evolving impact of pandemic-related disruptions on TB mortality across Southeast Asia in 2021.

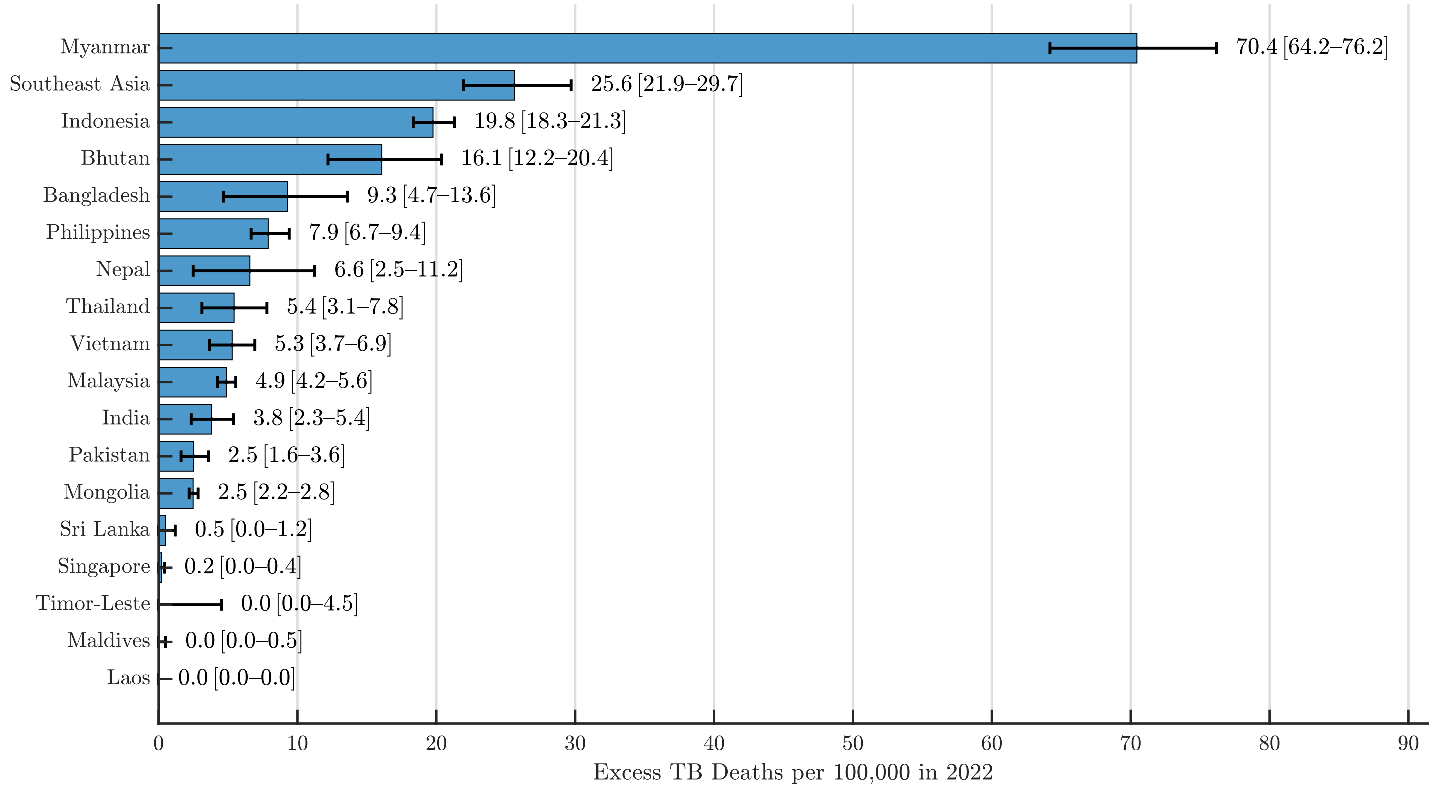


Figure S9: Estimated excess tuberculosis (TB) mortality rates (per 100,000 population) in Southeast Asian countries in 2022 using an ensemble subepidemic modeling framework with two overlapping waves. The model was calibrated using pre-pandemic mortality data from 2010 to 2019 and used to forecast expected deaths during the third year of the COVID-19 pandemic. Horizontal bars represent the median excess mortality rate for each country, with horizontal lines indicating the 95% uncertainty intervals (lower and upper bounds). Exact rate values are shown alongside each bar for clarity and comparison. This figure reflects the continued influence of pandemic-related health system disruptions on TB mortality across the region in 2022.

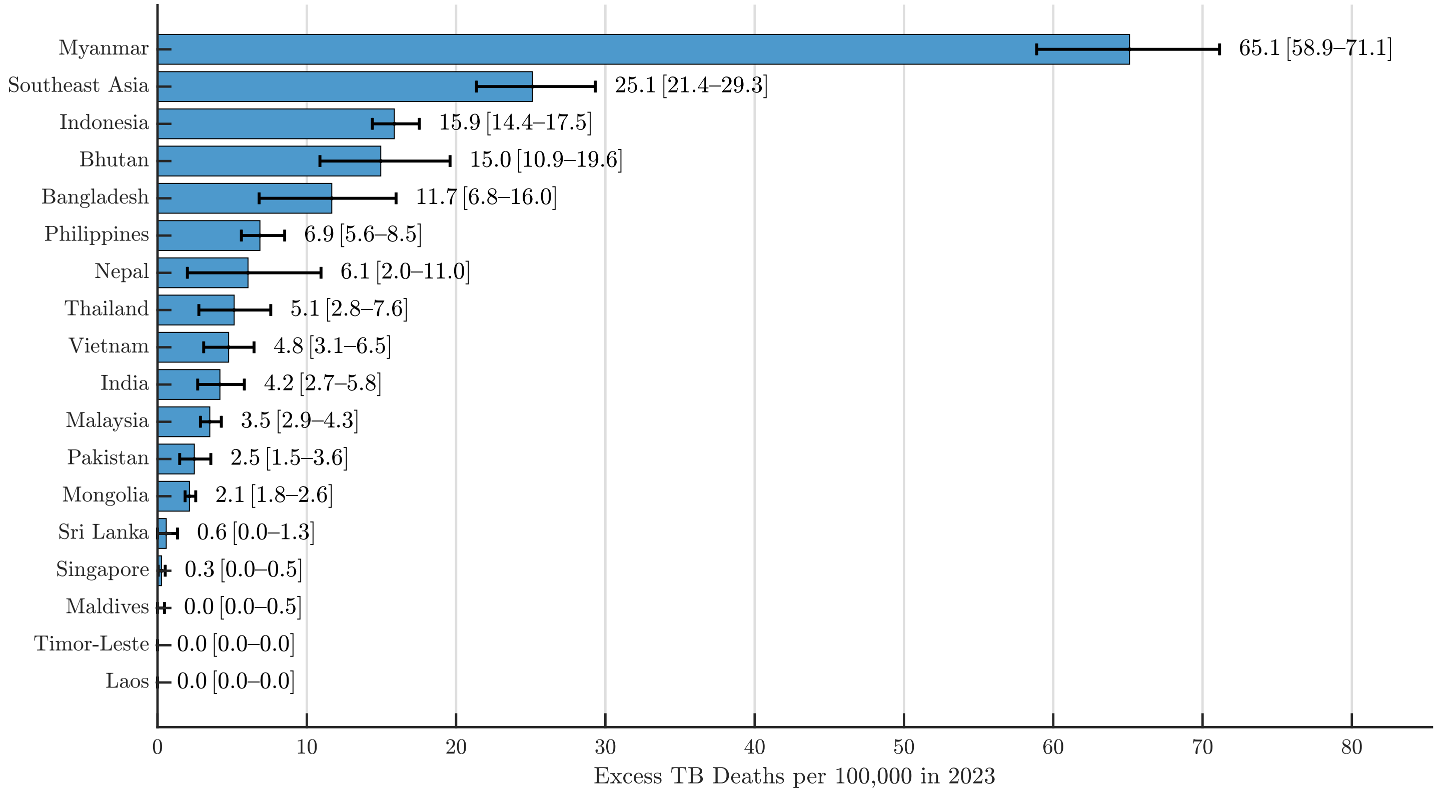


Figure S10: Estimated excess tuberculosis (TB) mortality rates (per 100,000 population) in Southeast Asian countries in 2023 using an ensemble subepidemic modeling framework with two overlapping waves. The model was calibrated to TB mortality data from 2010 to 2019 and used to estimate expected mortality during the fourth year of the COVID-19 pandemic. Horizontal bars represent the median excess mortality rate for each country, with horizontal lines indicating the 95% uncertainty intervals (lower and upper bounds). Exact rate values are labeled alongside each bar to facilitate comparison. This figure illustrates the lingering effects of pandemic-related disruptions on TB mortality across Southeast Asia in 2023, as countries began transitioning toward post-pandemic recovery.

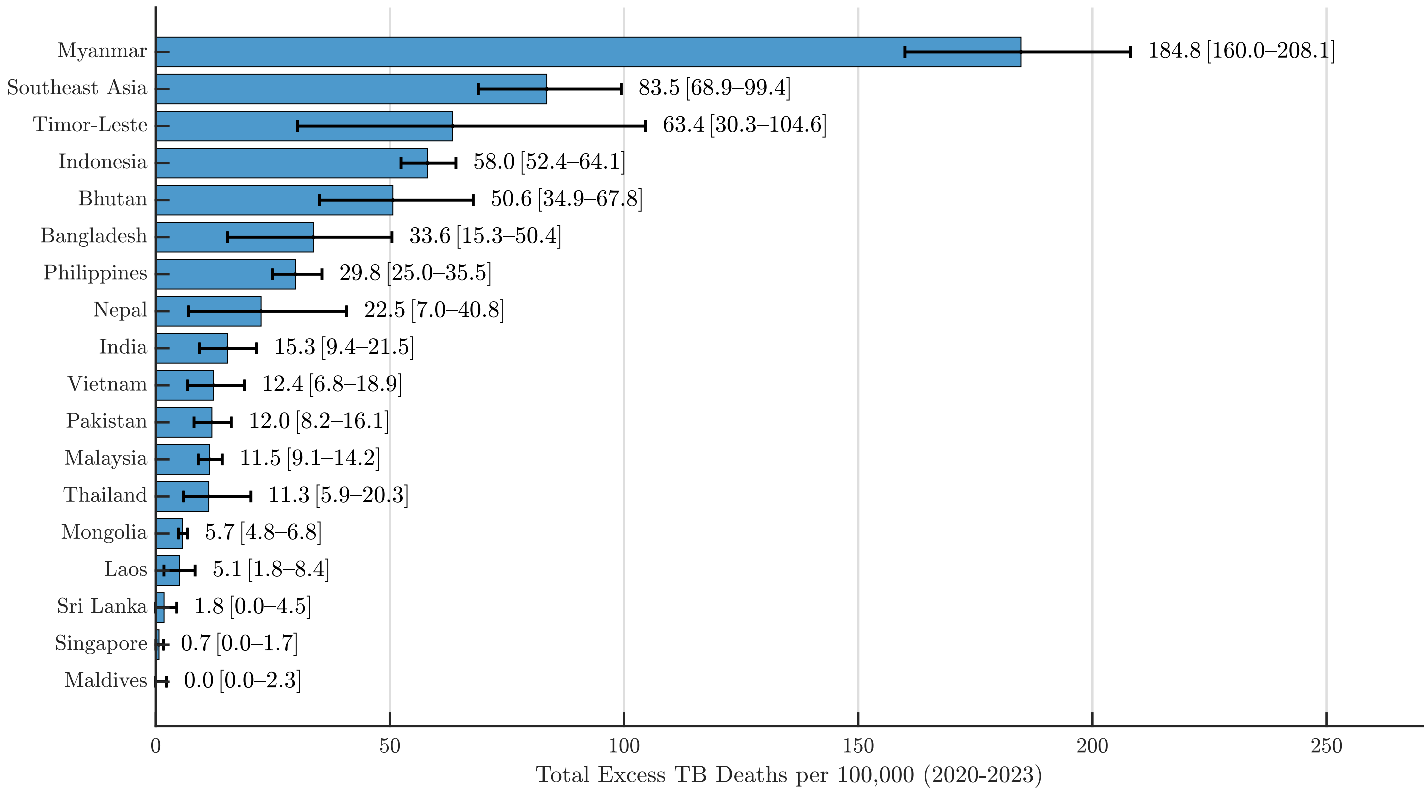


Figure S11: Cumulative excess tuberculosis (TB) mortality rates (per 100,000 population) in Southeast Asian countries from 2020 to 2023, estimated using an ensemble subepidemic modeling framework with two overlapping waves. The model was calibrated to TB mortality data from 2010 to 2019 and used to forecast expected deaths during the four-year COVID-19 pandemic period. Horizontal bars represent the median total excess mortality rate for each country, with horizontal lines indicating the 95% uncertainty intervals (lower and upper bounds). Exact rate values are annotated next to each bar to support direct country-level comparisons. This figure summarizes the cumulative impact of pandemic-related disruptions on TB mortality in the region and highlights persistent cross-country disparities.

| | Excess TB mortality rate (LB,UB) | | | | |
|---|---|---|---|---|---|
| Country | **2020** | **2021** | **2022** | **2023** | **Total** |
| **Bangladesh** | 5.2 (0.8, 9.2) | 7.5 (3.1, 11.6) | 9.3 (4.7, 13.6) | 11.7 (6.8, 16.0) | 33.6 (15.3, 50.4) |
| **Bhutan** | 7.8 (4.0, 11.7) | 11.8 (7.9, 16.1) | 16.1 (12.2, 20.4) | 15.0 (10.9, 19.6) | 50.6 (34.9, 67.8) |
| **India** | 2.7 (1.2, 4.2) | 4.6 (3.1, 6.1) | 3.8 (2.3, 5.4) | 4.2 (2.7, 5.8) | 15.3 (9.4, 21.5) |
| **Indonesia** | 5.3 (3.9, 6.6) | 17.1 (15.8, 18.6) | 19.8 (18.3, 21.3) | 15.9 (14.4, 17.5) | 58.0 (52.4, 64.1) |
| **Laos** | 0.0 (0.0, 0.0) | 5.1 (1.8, 8.4) | 0.0 (0.0, 0.0) | 0.0 (0.0, 0.0) | 5.1 (1.8, 8.4) |
| **Malaysia** | 0.7 (0.1, 1.2) | 2.5 (1.9, 3.1) | 4.9 (4.2, 5.6) | 3.5 (2.9, 4.3) | 11.5 (9.1, 14.2) |
| **Maldives** | 0.0 (0.0, 0.7) | 0.0 (0.0, 0.6) | 0.0 (0.0, 0.5) | 0.0 (0.0, 0.5) | 0.0 (0.0, 2.3) |
| **Mongolia** | 0.0 (0.0, 0.0) | 1.0 (0.8, 1.4) | 2.5 (2.2, 2.8) | 2.1 (1.8, 2.6) | 5.7 (4.8, 6.8) |
| **Myanmar** | 8.4 (2.4, 14.2) | 40.8 (34.5, 46.6) | 70.4 (64.2, 76.2) | 65.1 (58.9, 71.1) | 184.8 (160.0, 208.1) |
| **Nepal** | 3.3 (0.0, 7.6) | 6.5 (2.5, 10.9) | 6.6 (2.5, 11.2) | 6.1 (2.0, 11.0) | 22.5 (7.0, 40.8) |
| **Pakistan** | 2.6 (1.6, 3.6) | 4.4 (3.5, 5.4) | 2.5 (1.6, 3.6) | 2.5 (1.5, 3.6) | 12.0 (8.2, 16.1) |
| **Philippines** | 5.2 (4.0, 6.4) | 9.8 (8.7, 11.2) | 7.9 (6.7, 9.4) | 6.9 (5.6, 8.5) | 29.8 (25.0, 35.5) |
| **Singapore** | 0.1 (0.0, 0.3) | 0.1 (0.0, 0.4) | 0.2 (0.0, 0.4) | 0.3 (0.0, 0.5) | 0.7 (0.0, 1.7) |
| **Sri Lanka** | 0.3 (0.0, 0.9) | 0.4 (0.0, 1.0) | 0.5 (0.0, 1.2) | 0.6 (0.0, 1.3) | 1.8 (0.0, 4.5) |
| **Thailand** | 0.0 (0.0, 1.8) | 0.8 (0.0, 3.1) | 5.4 (3.1, 7.8) | 5.1 (2.8, 7.6) | 11.3 (5.9, 20.3) |
| **Timor-Leste** | 28.6 (13.5, 45.3) | 34.8 (16.8, 54.8) | 0.0 (0.0, 4.5) | 0.0 (0.0, 0.0) | 63.4 (30.3, 104.6) |
| **Vietnam** | 0.6 (0.0, 2.2) | 1.7 (0.1, 3.4) | 5.3 (3.7, 6.9) | 4.8 (3.1, 6.5) | 12.4 (6.8, 18.9) |
| **Southeast Asia** | 10.2 (6.6, 13.9) | 22.5 (18.9, 26.4) | 25.6 (21.9, 29.7) | 25.1 (21.4, 29.3) | 83.5 (68.9, 99.4) |

Table S2: Estimated excess tuberculosis (TB) mortality rates (per 100,000 population) in Southeast Asian countries from 2020 to 2023, presented both by individual year and as a cumulative total, using an ensemble subepidemic modeling framework with two overlapping waves. The model was calibrated to TB mortality data from 2010 to 2019 and used to forecast expected deaths for each pandemic year. Bars represent median excess mortality rates per country for each year and for the total 2020–2023 period, with horizontal lines showing the corresponding 95% uncertainty intervals (lower and upper bounds). Exact rate values are labeled alongside each bar to facilitate comparisons across time and between countries. This figure illustrates temporal changes and cumulative patterns in TB mortality burden during the COVID-19 pandemic across Southeast Asia.

### S1.3 SMR

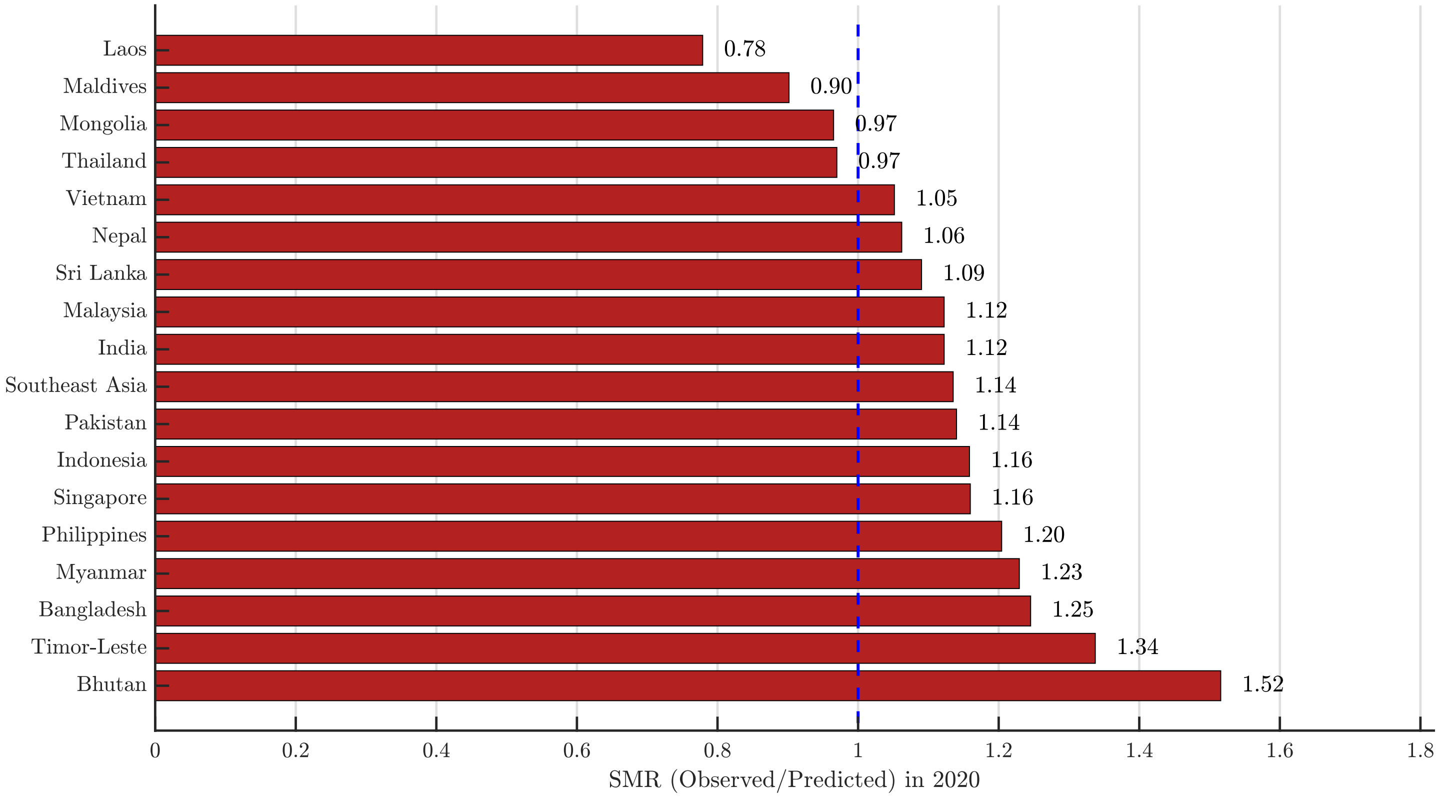


Figure S12: Estimated standardized mortality ratios (SMRs) for tuberculosis (TB) in Southeast Asian countries in 2020, based on an ensemble subepidemic modeling framework with two overlapping waves. The model was calibrated using TB mortality data from 2010 to 2019 and used to estimate expected deaths during the first year of the COVID-19 pandemic. Horizontal bars represent the SMR for each country, defined as the ratio of observed to expected TB deaths. Exact SMR values are labeled next to each bar to facilitate comparison. An SMR above 1 indicates excess mortality, while values below 1 suggest fewer TB deaths than expected. This figure illustrates the initial disruption in TB control efforts during the pandemic across Southeast Asia.

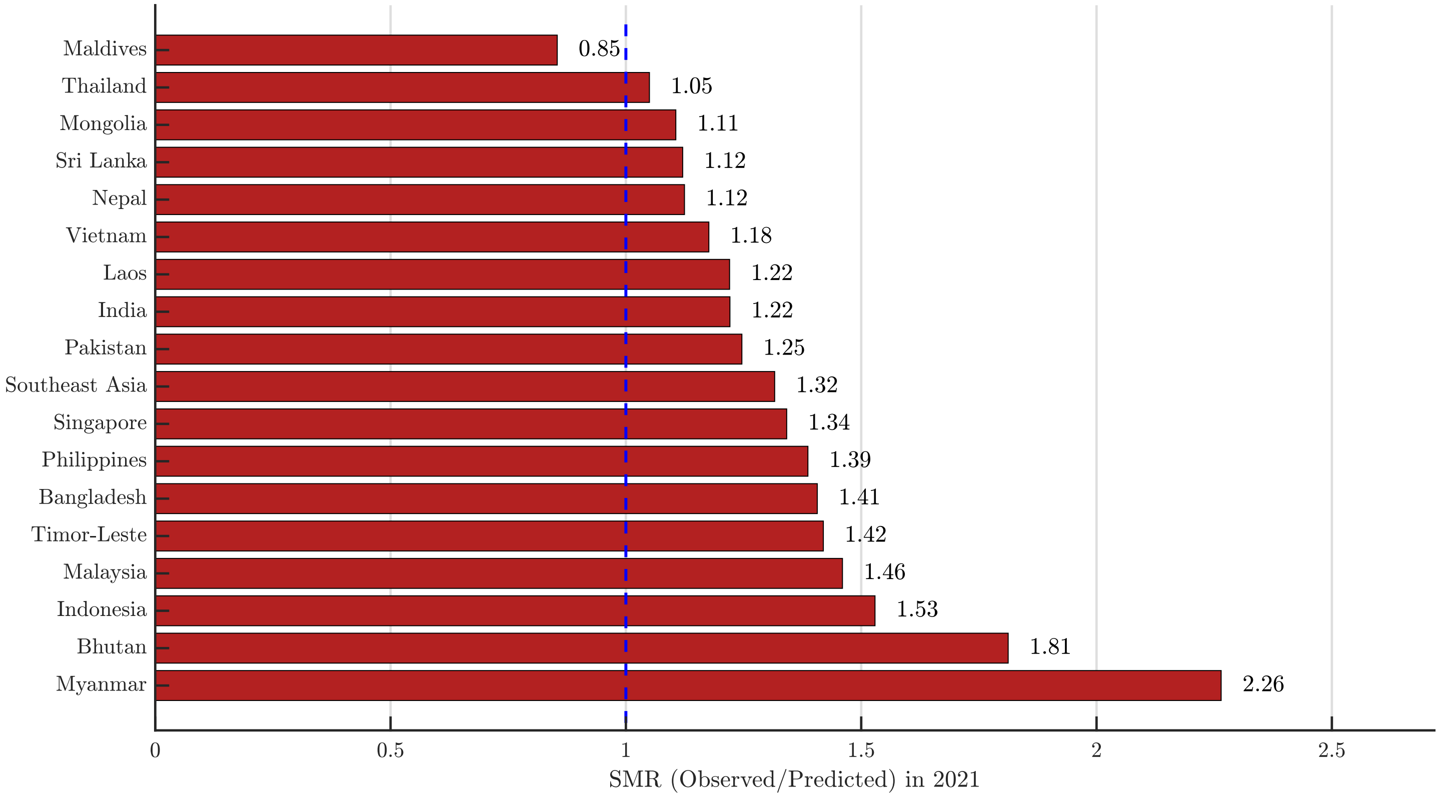


Figure S13: Estimated standardized mortality ratios (SMRs) for tuberculosis (TB) in Southeast Asian countries in 2021, based on an ensemble subepidemic modeling framework with two overlapping waves. The model was calibrated using TB mortality data from 2010 to 2019 and used to estimate expected deaths during the second year of the COVID-19 pandemic. Horizontal bars represent the SMR for each country, calculated as the ratio of observed to expected TB deaths, with exact values labeled beside each bar. An SMR greater than 1 indicates excess mortality relative to baseline trends, while an SMR below 1 suggests lower-than-expected TB mortality. This figure highlights regional variations in TB mortality during the prolonged disruption of health services in 2021.

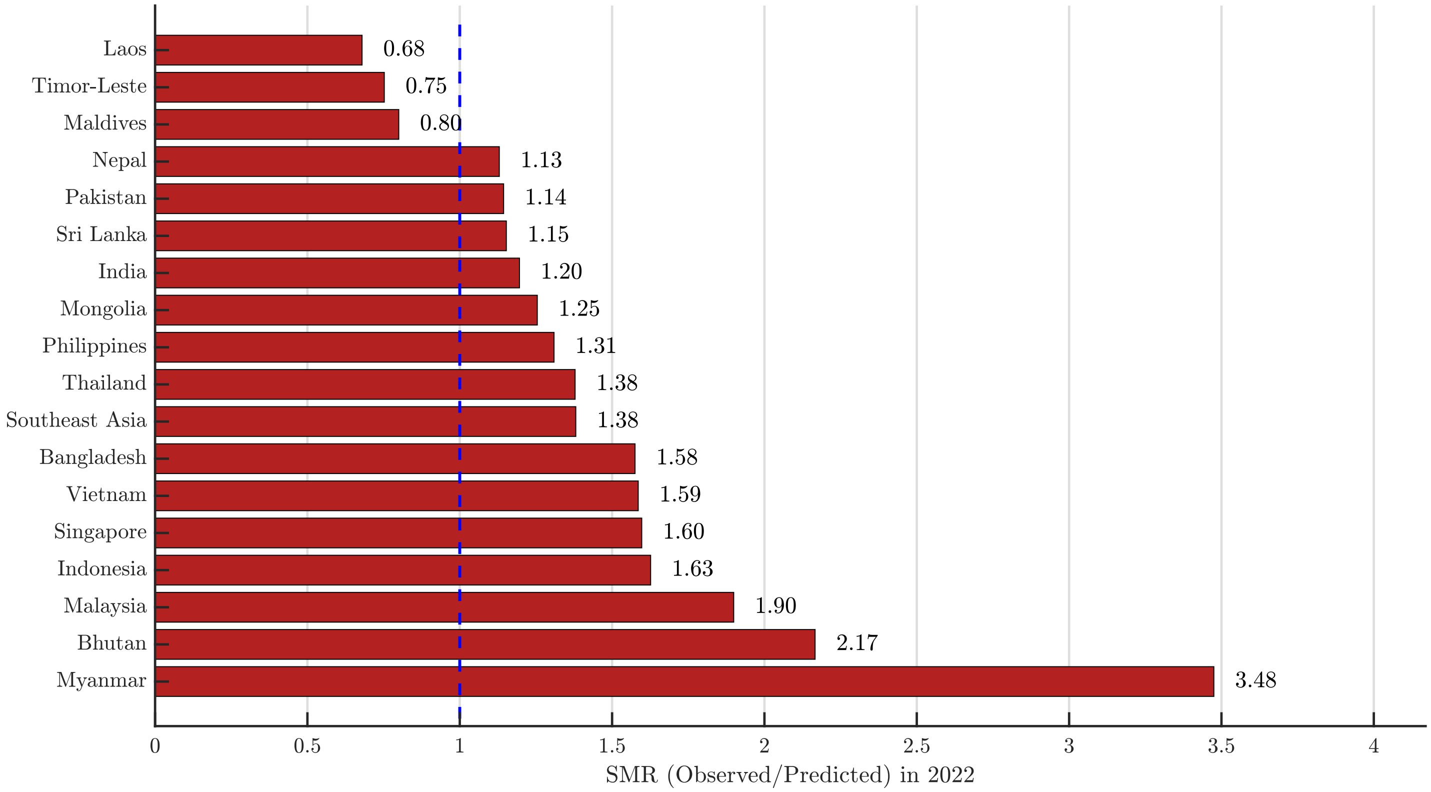


Figure S14: Estimated standardized mortality ratios (SMRs) for tuberculosis (TB) in Southeast Asian countries in 2022, using an ensemble subepidemic modeling framework with two overlapping waves. The model was calibrated to TB mortality data from 2010 to 2019 and used to estimate expected mortality during the third year of the COVID-19 pandemic. Horizontal bars represent the SMR for each country, calculated as the ratio of observed to expected TB deaths, with exact values shown next to each bar. An SMR greater than 1 indicates excess TB mortality, while values below 1 reflect lower-than-expected mortality. This figure captures how TB mortality patterns evolved across Southeast Asia as health systems began adapting to prolonged pandemic-related disruptions.

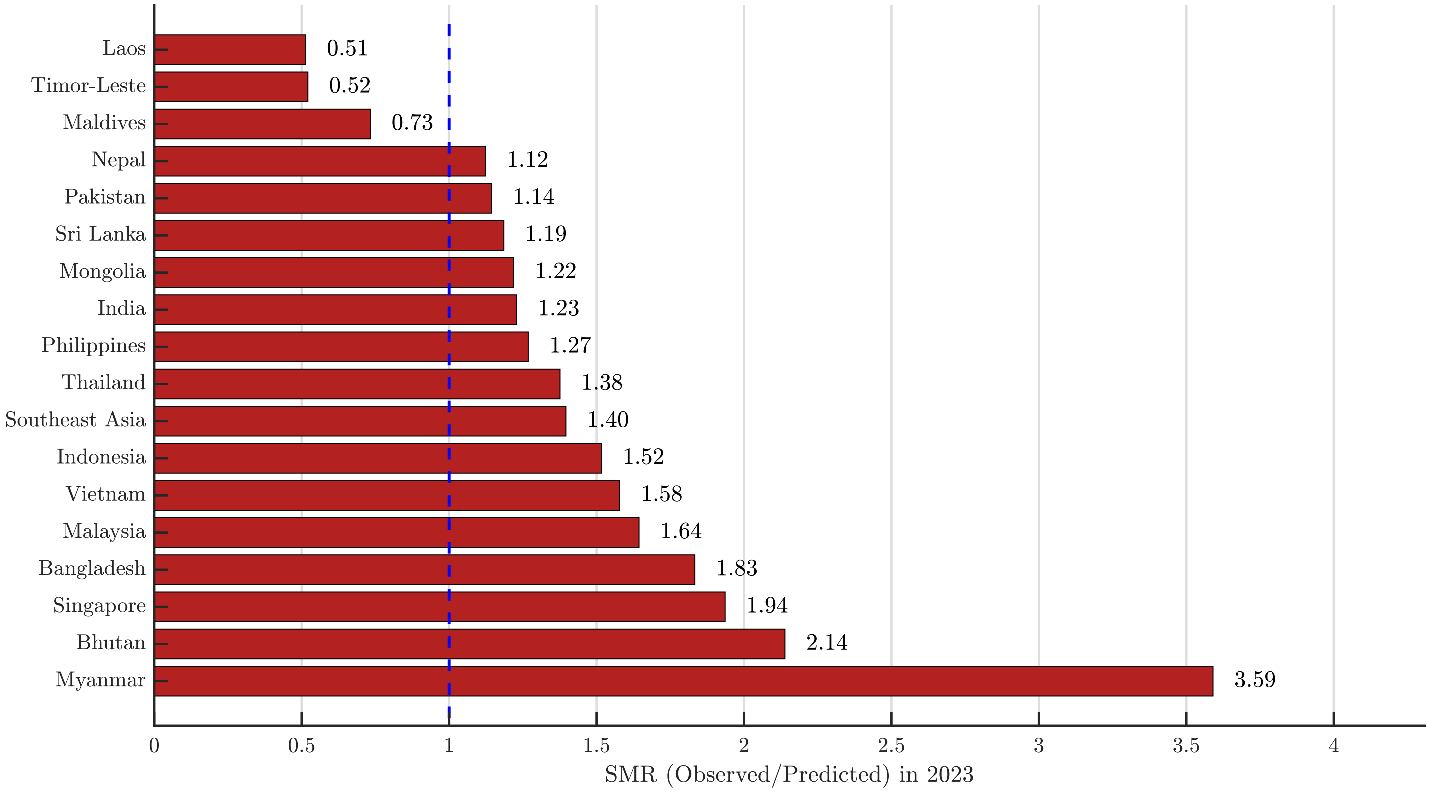


Figure S15: Estimated standardized mortality ratios (SMRs) for tuberculosis (TB) in Southeast Asian countries in 2023, based on an ensemble subepidemic modeling framework with two overlapping waves. The model was calibrated to TB mortality data from 2010 to 2019 and used to forecast expected mortality during the fourth year of the COVID-19 pandemic. Horizontal bars display the SMR for each country, calculated as the ratio of observed to expected TB deaths, with exact values labeled next to each bar. An SMR above 1 indicates excess mortality, while an SMR below 1 suggests fewer deaths than expected. This figure reflects the TB mortality burden in 2023 as countries moved toward pandemic recovery and the long-term effects of service disruptions became apparent.

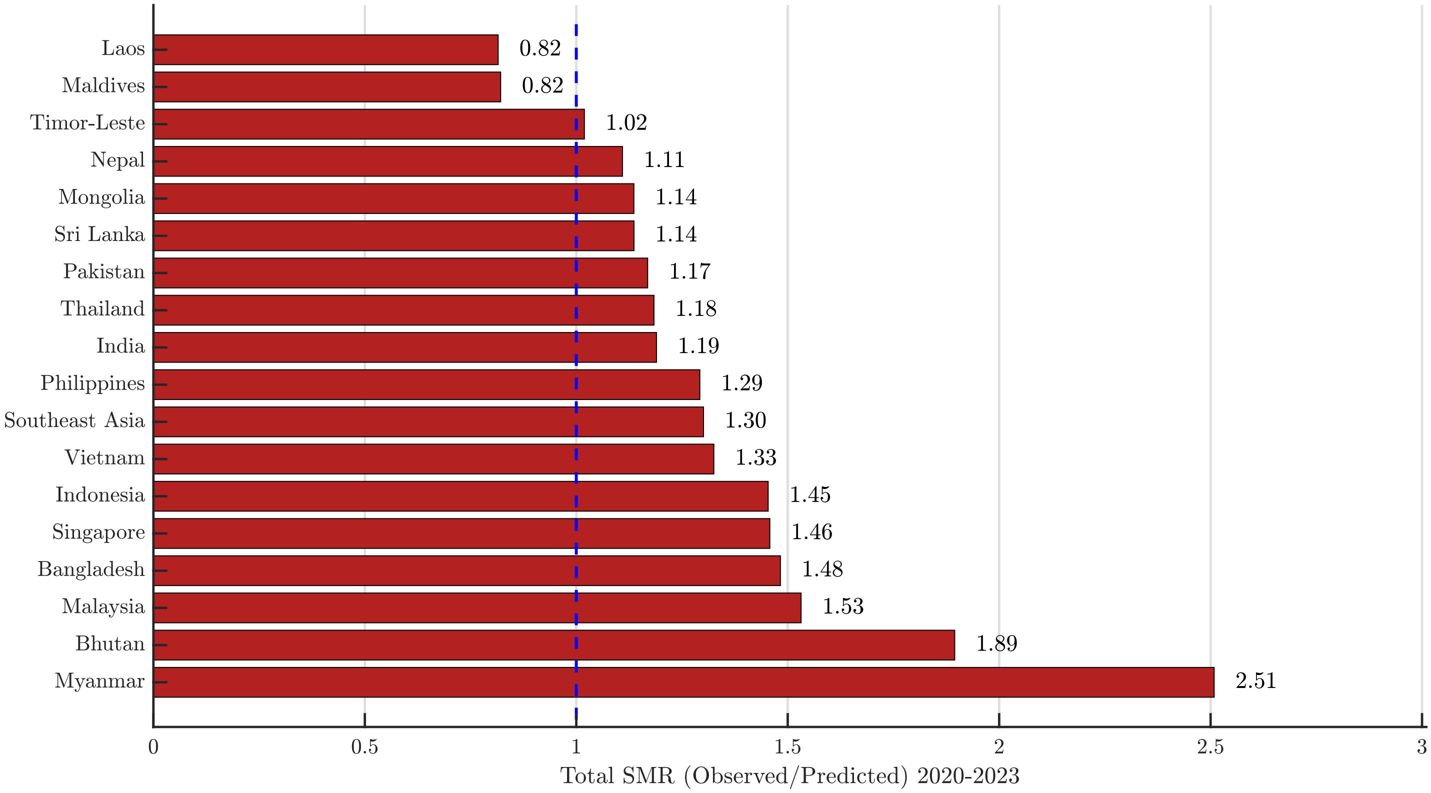


Figure S16: Cumulative standardized mortality ratios (SMRs) for tuberculosis (TB) in Southeast Asian countries from 2020 to 2023, estimated using an ensemble subepidemic modeling framework with two overlapping waves. The model was calibrated to TB mortality data from 2010 to 2019 and used to forecast expected deaths over the four-year COVID-19 pandemic period. Horizontal bars represent the SMR for each country, calculated as the ratio of total observed to total expected TB deaths from 2020 to 2023, with exact values displayed next to each bar. An SMR greater than 1 indicates excess TB mortality, while values below 1 indicate mortality lower than expected. This figure summarizes the long-term impact of the pandemic on TB mortality in Southeast Asia and highlights persistent inter-country variation.

# S2 The Americas

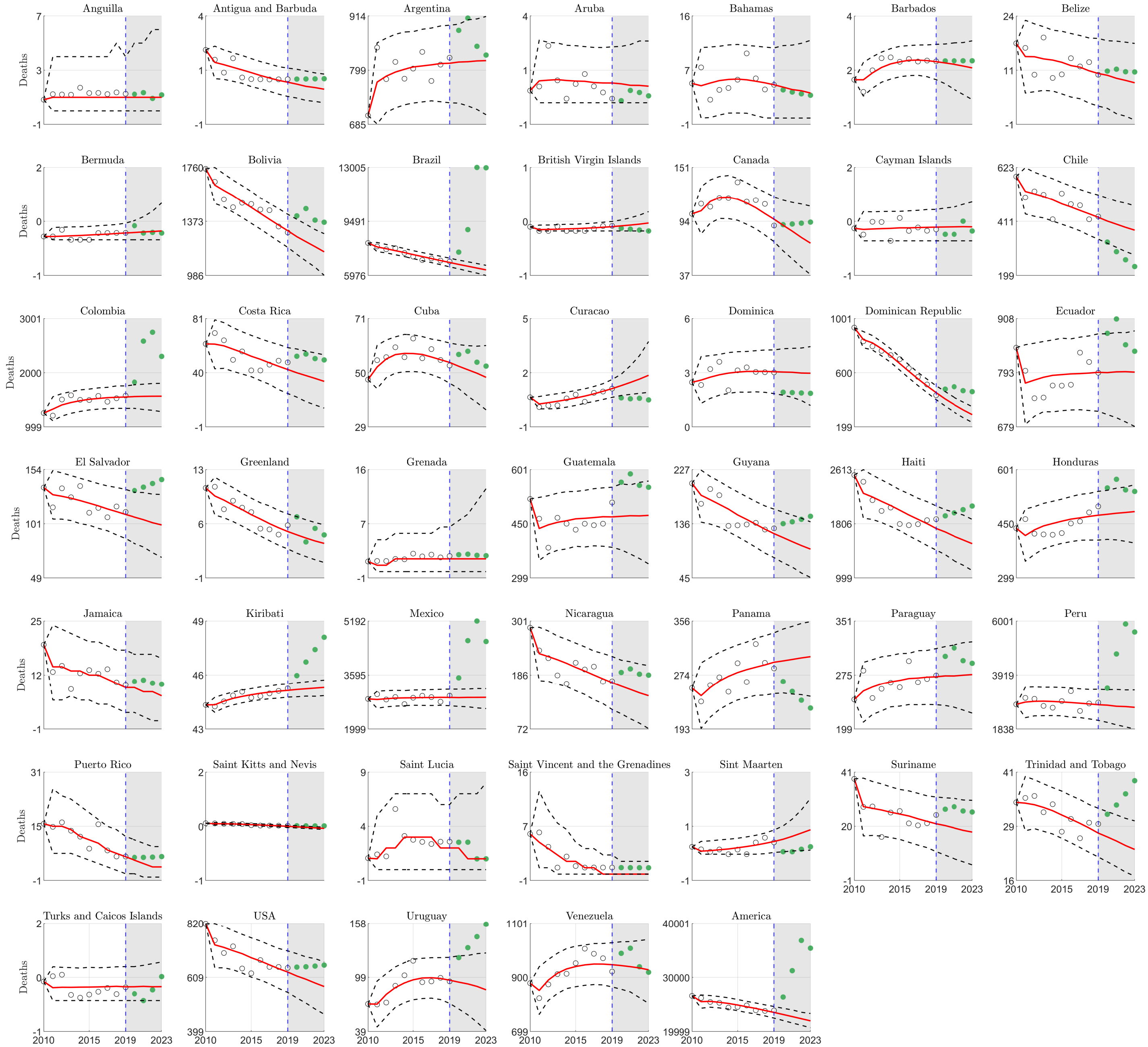

Figure S17: Forecasted tuberculosis (TB) deaths in the Americas from 2020 to 2023 using a subepidemic ensemble modeling framework. The model was calibrated to TB mortality data from 2010 to 2019 and used to estimate expected deaths during the COVID-19 pandemic, with the Ranked 1 model selected based on best fit criteria. The red curve indicates the median forecast, and black dashed lines represent the 95% prediction interval (PI). Observed annual TB deaths are shown as circles: black circles represent the calibration period, and green-filled circles represent actual data points during the forecast period.

## S2.1 Excess mortality

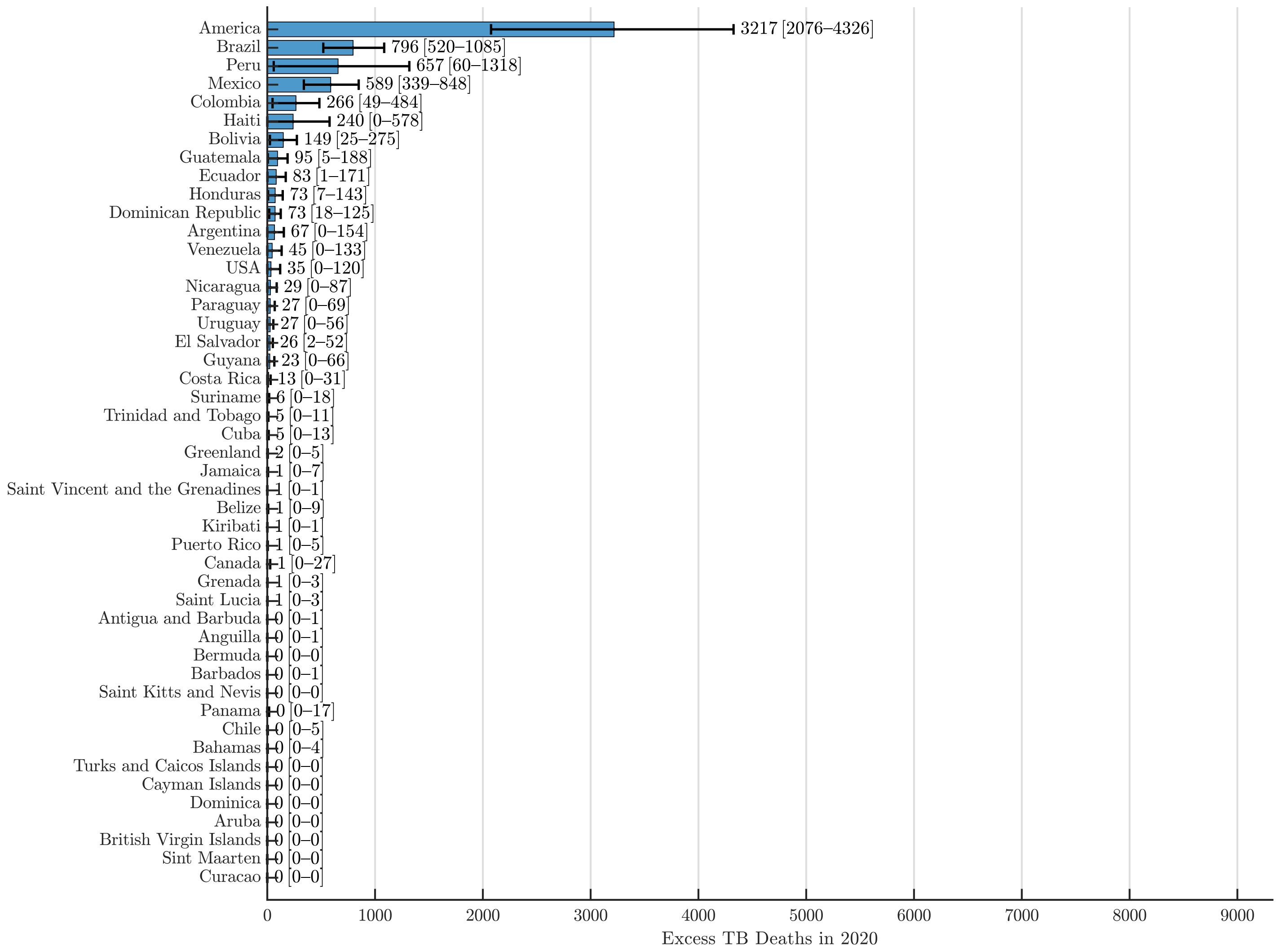


Figure S18: Estimated excess tuberculosis (TB) deaths in countries across the Americas in 2020, based on an ensemble subepidemic modeling framework with two overlapping waves. The model was calibrated using WHO-reported TB mortality data from 2010 to 2019 and used to estimate expected deaths for the first year of the COVID-19 pandemic. Horizontal bars represent the median excess TB deaths per country, with horizontal lines denoting the 95% uncertainty intervals (lower and upper bounds). Exact mortality figures are labeled next to each bar for ease of comparison. This figure illustrates the early pandemic disruptions in TB control across the region and highlights country-level variation in excess mortality.

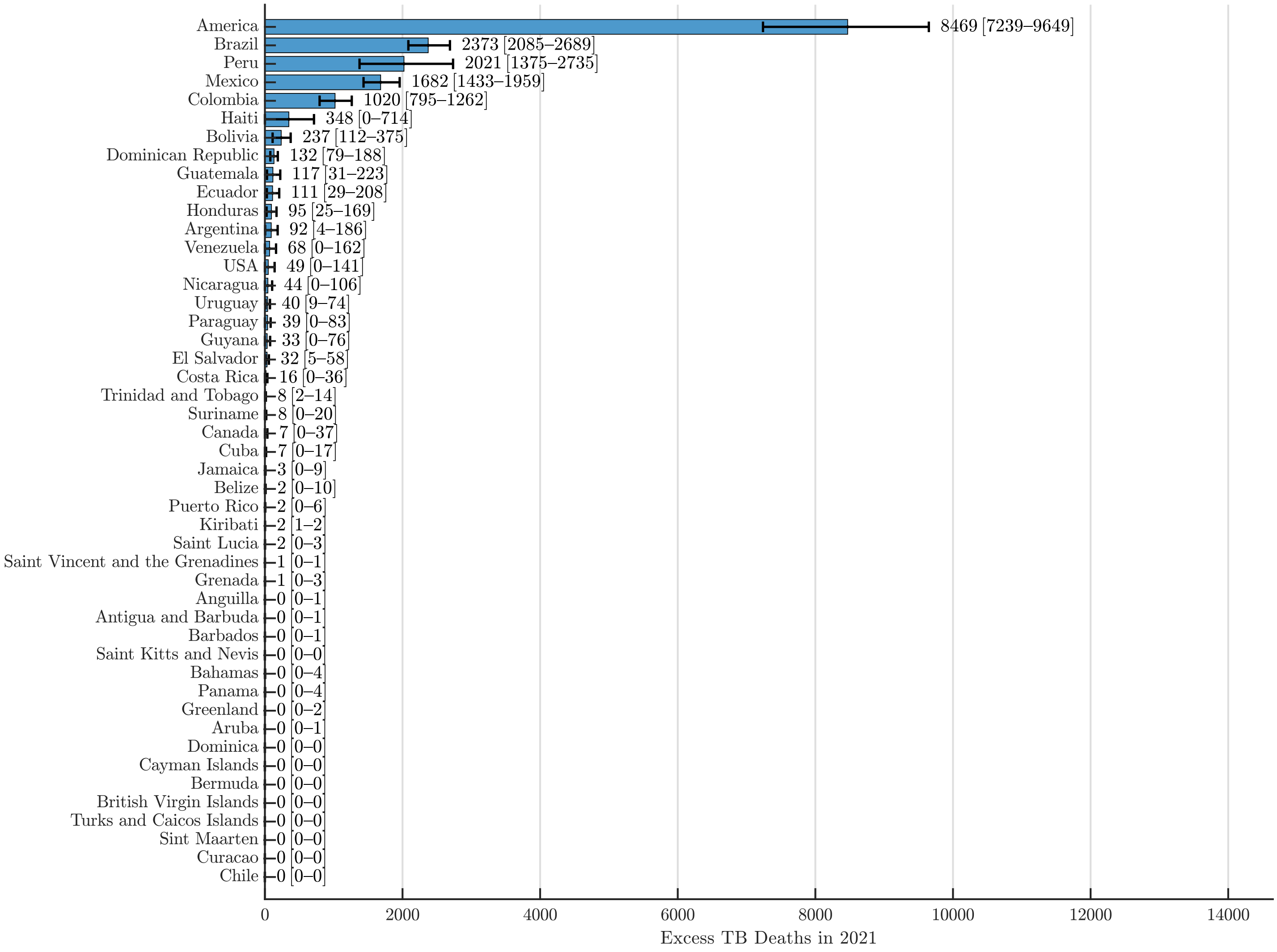


Figure S19: Estimated excess tuberculosis (TB) deaths in countries across the Americas in 2021, using an ensemble subepidemic modeling framework with two overlapping waves. The model was calibrated to TB mortality data from 2010 to 2019 and used to forecast expected deaths during the second year of the COVID-19 pandemic. Horizontal bars represent the median excess TB deaths per country, while horizontal lines indicate the 95% uncertainty intervals (lower and upper bounds). Exact excess death values are labeled adjacent to each bar to facilitate comparison. This figure captures the continued impact of pandemic-related service disruptions on TB mortality across the Americas in 2021.

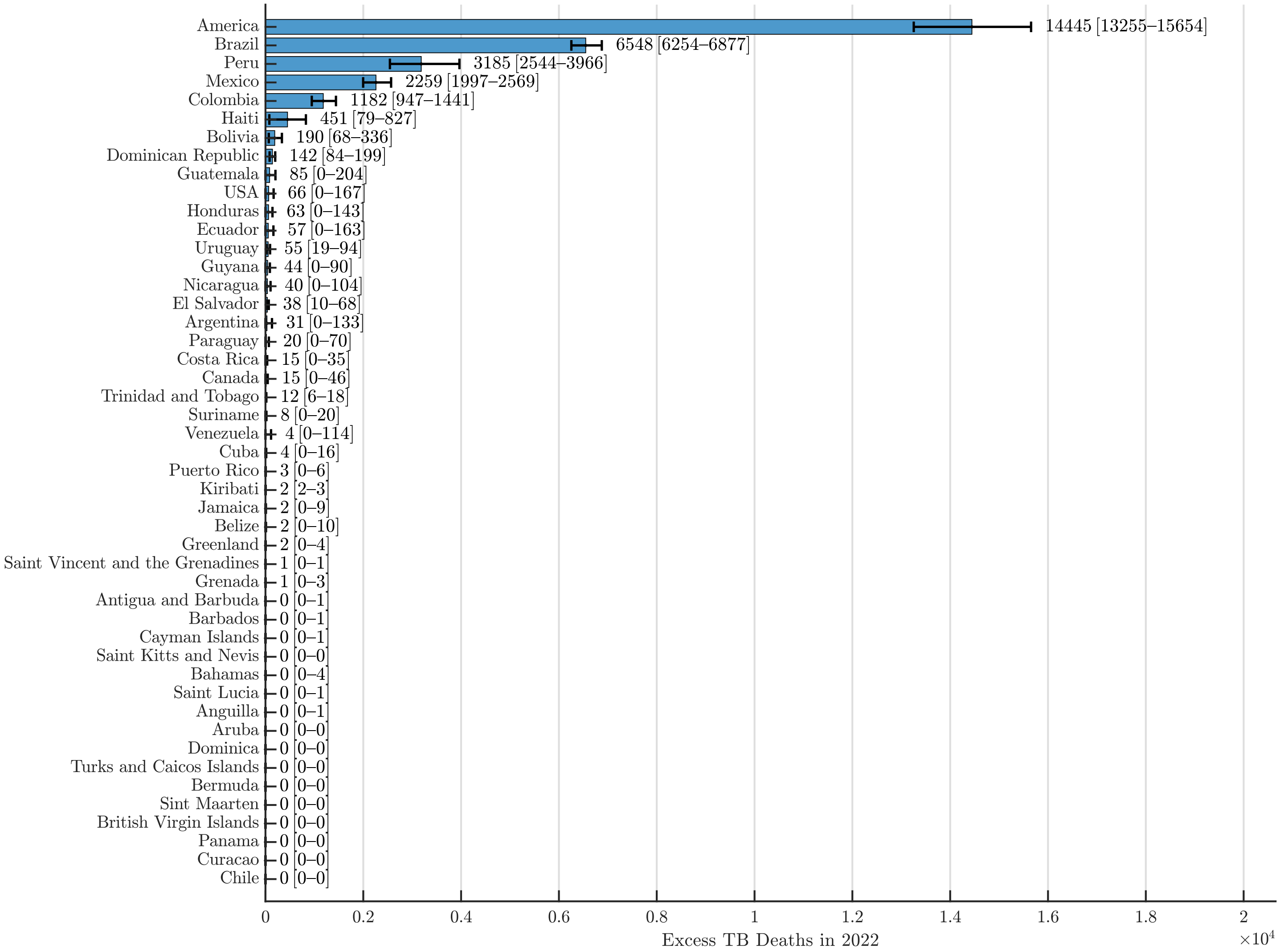


Figure S20: Estimated excess tuberculosis (TB) deaths in countries across the Americas in 2022, using an ensemble subepidemic modeling framework with two overlapping waves. The model was calibrated to TB mortality data from 2010 to 2019 and used to estimate expected deaths during the third year of the COVID-19 pandemic. Horizontal bars show the median excess TB deaths per country, with horizontal lines representing the 95% uncertainty intervals (lower and upper bounds). Exact mortality estimates are displayed next to each bar for cross-country comparison. This figure reflects evolving pandemic-related disruptions and recovery trajectories in TB mortality across the Americas in 2022.

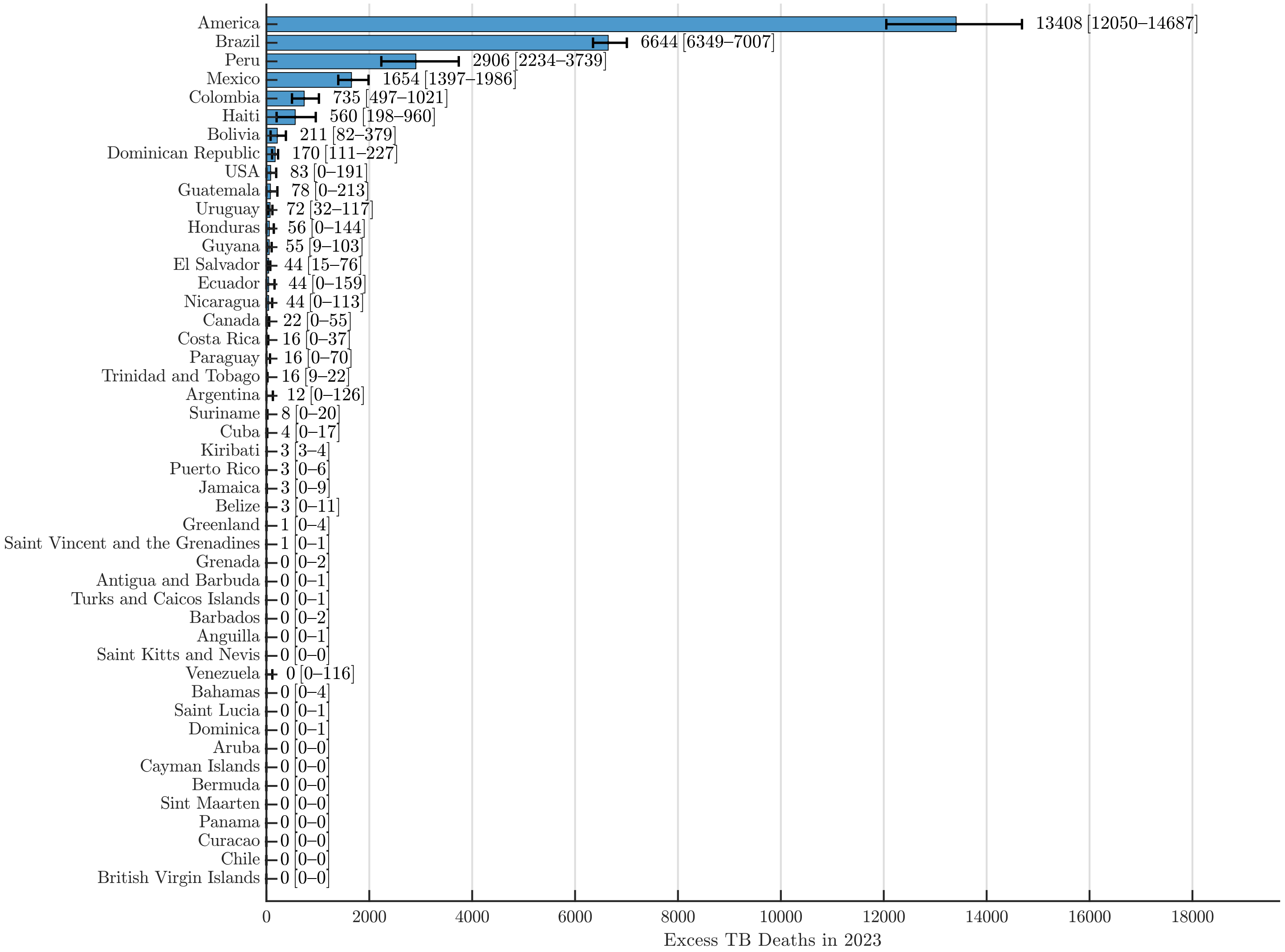


Figure S21: Estimated excess tuberculosis (TB) deaths in countries across the Americas in 2023, based on an ensemble subepidemic modeling framework with two overlapping waves. The model was calibrated to TB mortality data from 2010 to 2019 and used to forecast expected deaths during the fourth year of the COVID-19 pandemic. Horizontal bars represent the median excess TB deaths per country, with horizontal lines showing the 95% uncertainty intervals (lower and upper bounds). Exact mortality values are labeled next to each bar to enable direct comparisons. This figure highlights the residual impact of pandemic-related health system disruptions on TB mortality as countries across the Americas transitioned toward post-pandemic recovery.

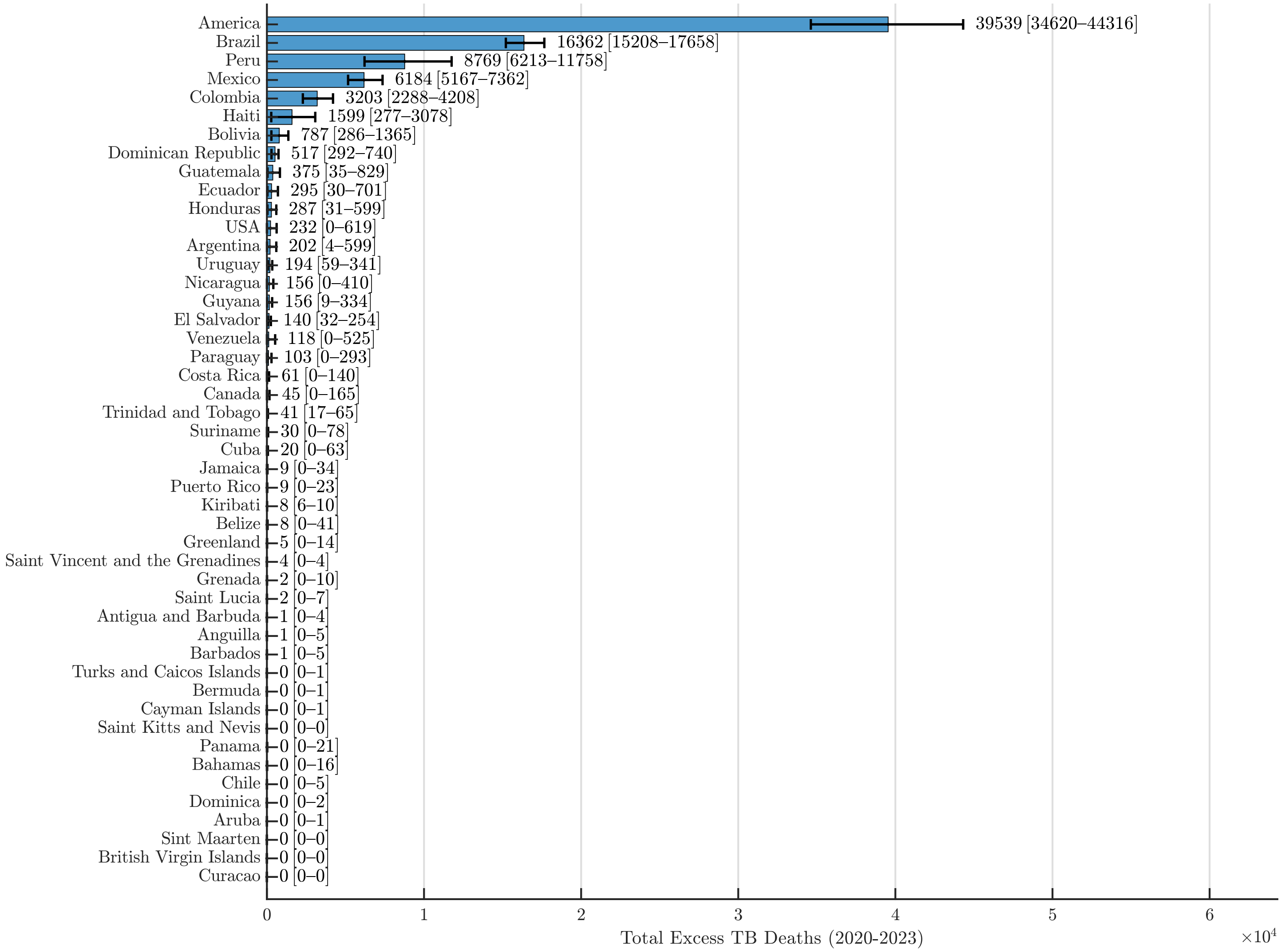


Figure S22: Total estimated excess tuberculosis (TB) deaths in countries across the Americas from 2020 to 2023, using an ensemble subepidemic modeling framework with two overlapping waves. The model was calibrated to TB mortality data from 2010 to 2019 and used to forecast expected deaths over the four-year COVID-19 pandemic period. Horizontal bars represent the median cumulative excess TB deaths per country, with horizontal lines denoting the 95% uncertainty intervals (lower and upper bounds). Exact mortality figures are labeled next to each bar to facilitate comparison. This figure summarizes the long-term impact of the pandemic on TB mortality across the Americas and highlights inter-country differences in cumulative excess burden.

| | Excess TB mortality (LB,UB) | | | | |
|---|---|---|---|---|---|
| Country | **2020** | **2021** | **2022** | **2023** | **Total** |
| **Anguilla** | 0 (0, 1) | 0 (0, 1) | 0 (0, 1) | 0 (0, 1) | 1 (0, 5) |
| **Antigua and Barbuda** | 0 (0, 1) | 0 (0, 1) | 0 (0, 1) | 0 (0, 1) | 1 (0, 4) |
| **Argentina** | 67 (0, 154) | 92 (4, 186) | 31 (0, 133) | 12 (0, 126) | 202 (4, 599) |
| **Aruba** | 0 (0, 0) | 0 (0, 1) | 0 (0, 0) | 0 (0, 0) | 0 (0, 1) |
| **Bahamas** | 0 (0, 4) | 0 (0, 4) | 0 (0, 4) | 0 (0, 4) | 0 (0, 16) |
| **Barbados** | 0 (0, 1) | 0 (0, 1) | 0 (0, 1) | 0 (0, 2) | 1 (0, 5) |
| **Belize** | 1 (0, 9) | 2 (0, 10) | 2 (0, 10) | 3 (0, 11) | 8 (0, 41) |
| **Bermuda** | 0 (0, 0) | 0 (0, 0) | 0 (0, 0) | 0 (0, 0) | 0 (0, 1) |
| **Bolivia** | 149 (25, 275) | 237 (112, 375) | 190 (68, 336) | 211 (82, 379) | 787 (286, 1365) |
| **Brazil** | 796 (520, 1085) | 2373 (2085, 2689) | 6548 (6254, 6877) | 6644 (6349, 7007) | 16362 (15208, 17658) |
| **British Virgin Islands** | 0 (0, 0) | 0 (0, 0) | 0 (0, 0) | 0 (0, 0) | 0 (0, 0) |
| **Canada** | 1 (0, 27) | 7 (0, 37) | 15 (0, 46) | 22 (0, 55) | 45 (0, 165) |
| **Cayman Islands** | 0 (0, 0) | 0 (0, 0) | 0 (0, 1) | 0 (0, 0) | 0 (0, 1) |
| **Chile** | 0 (0, 5) | 0 (0, 0) | 0 (0, 0) | 0 (0, 0) | 0 (0, 5) |
| **Colombia** | 266 (49, 484) | 1020 (795, 1262) | 1182 (947, 1441) | 735 (497, 1021) | 3203 (2288, 4208) |
| **Costa Rica** | 13 (0, 31) | 16 (0, 36) | 15 (0, 35) | 16 (0, 37) | 61 (0, 140) |
| **Cuba** | 5 (0, 13) | 7 (0, 17) | 4 (0, 16) | 4 (0, 17) | 20 (0, 63) |
| **Curacao** | 0 (0, 0) | 0 (0, 0) | 0 (0, 0) | 0 (0, 0) | 0 (0, 0) |
| **Dominica** | 0 (0, 0) | 0 (0, 0) | 0 (0, 0) | 0 (0, 1) | 0 (0, 2) |
| **Dominican Republic** | 73 (18, 125) | 132 (79, 188) | 142 (84, 199) | 170 (111, 227) | 517 (292, 740) |
| **Ecuador** | 83 (1, 171) | 111 (29, 208) | 57 (0, 163) | 44 (0, 159) | 295 (30, 701) |
| **El Salvador** | 26 (2, 52) | 32 (5, 58) | 38 (10, 68) | 44 (15, 76) | 140 (32, 254) |
| **Greenland** | 2 (0, 5) | 0 (0, 2) | 2 (0, 4) | 1 (0, 4) | 5 (0, 14) |
| **Grenada** | 1 (0, 3) | 1 (0, 3) | 1 (0, 3) | 0 (0, 2) | 2 (0, 10) |
| **Guatemala** | 95 (5, 188) | 117 (31, 223) | 85 (0, 204) | 78 (0, 213) | 375 (35, 829) |
| **Guyana** | 23 (0, 66) | 33 (0, 76) | 44 (0, 90) | 55 (9, 103) | 156 (9, 334) |
| **Haiti** | 240 (0, 578) | 348 (0, 714) | 451 (79, 827) | 560 (198, 960) | 1599 (277, 3078) |
| **Honduras** | 73 (7, 143) | 95 (25, 169) | 63 (0, 143) | 56 (0, 144) | 287 (31, 599) |
| **Jamaica** | 1 (0, 7) | 3 (0, 9) | 2 (0, 9) | 3 (0, 9) | 9 (0, 34) |
| **Kiribati** | 1 (0, 1) | 2 (1, 2) | 2 (2, 3) | 3 (3, 4) | 8 (6, 10) |
| **Mexico** | 589 (339, 848) | 1682 (1433, 1959) | 2259 (1997, 2569) | 1654 (1397, 1986) | 6184 (5167, 7362) |
| **Nicaragua** | 29 (0, 87) | 44 (0, 106) | 40 (0, 104) | 44 (0, 113) | 156 (0, 410) |
| **Panama** | 0 (0, 17) | 0 (0, 4) | 0 (0, 0) | 0 (0, 0) | 0 (0, 21) |
| **Paraguay** | 27 (0, 69) | 39 (0, 83) | 20 (0, 70) | 16 (0, 70) | 103 (0, 293) |
| **Peru** | 657 (60, 1318) | 2021 (1375, 2735) | 3185 (2544, 3966) | 2906 (2234, 3739) | 8769 (6213, 11758) |
| **Puerto Rico** | 1 (0, 5) | 2 (0, 6) | 3 (0, 6) | 3 (0, 6) | 9 (0, 23) |
| **Saint Kitts and Nevis** | 0 (0, 0) | 0 (0, 0) | 0 (0, 0) | 0 (0, 0) | 0 (0, 0) |
| **Saint Lucia** | 1 (0, 3) | 2 (0, 3) | 0 (0, 1) | 0 (0, 1) | 2 (0, 7) |
| **Saint Vincent and the Grenadines** | 1 (0, 1) | 1 (0, 1) | 1 (0, 1) | 1 (0, 1) | 4 (0, 4) |
| **Sint Maarten** | 0 (0, 0) | 0 (0, 0) | 0 (0, 0) | 0 (0, 0) | 0 (0, 0) |
| **Suriname** | 6 (0, 18) | 8 (0, 20) | 8 (0, 20) | 8 (0, 20) | 30 (0, 78) |
| **Trinidad and Tobago** | 5 (0, 11) | 8 (2, 14) | 12 (6, 18) | 16 (9, 22) | 41 (17, 65) |
| **Turks and Caicos Islands** | 0 (0, 0) | 0 (0, 0) | 0 (0, 0) | 0 (0, 1) | 0 (0, 1) |
| **USA** | 35 (0, 120) | 49 (0, 141) | 66 (0, 167) | 83 (0, 191) | 232 (0, 619) |
| **Uruguay** | 27 (0, 56) | 40 (9, 74) | 55 (19, 94) | 72 (32, 117) | 194 (59, 341) |
| **Venezuela** | 45 (0, 133) | 68 (0, 162) | 4 (0, 114) | 0 (0, 116) | 118 (0, 525) |
| **America** | 3217 (2076, 4326) | 8469 (7239, 9649) | 14445 (13255, 15654) | 13408 (12050, 14687) | 39539 (34620, 44316) |

Table S3: Estimated excess tuberculosis (TB) deaths in countries across the Americas from 2020 to 2023, presented by individual year and as a cumulative total, using an ensemble subepidemic modeling framework with two overlapping waves. The model was calibrated to TB mortality data from 2010 to 2019 and used to estimate expected deaths during the COVID-19 pandemic. Bars represent the median excess deaths for each year and for the total period, with horizontal lines showing the 95% uncertainty intervals (lower and upper bounds). Exact excess mortality values are labeled next to each bar to facilitate temporal and cross-country comparisons. This figure illustrates the progression and aggregate burden of pandemic-associated TB mortality in the Americas.

## S2.2 Excess mortality rate

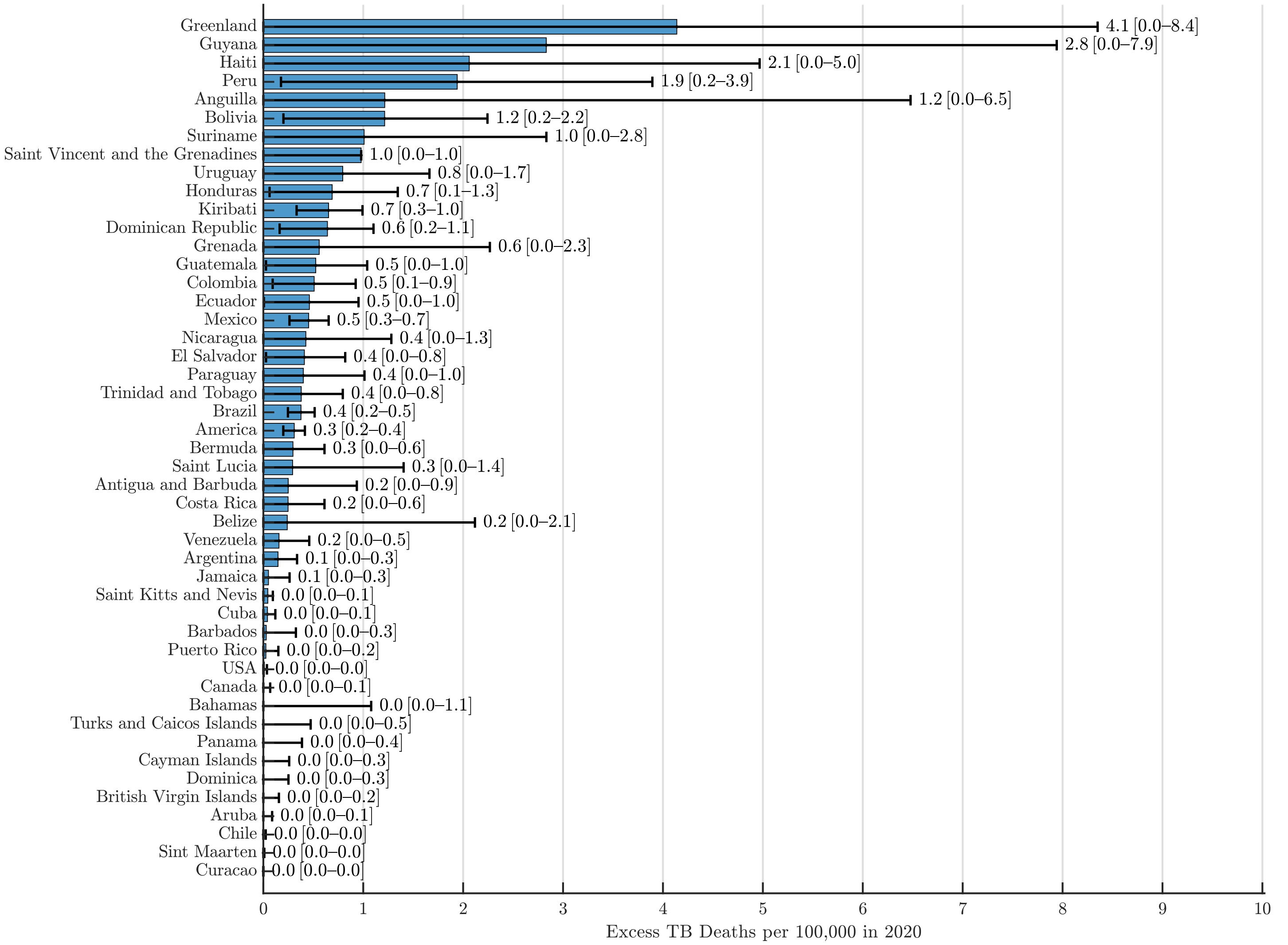


Figure S23: Excess TB mortality rate per 100,000 in the Americas in 2020, estimated using an ensemble framework with two sub-epidemics. The horizontal bar charts display the median excess mortality rate for each country, with horizontal lines extending from each bar representing the corresponding confidence intervals (lower and upper bounds). Exact mortality rate numbers are also provided for each country.

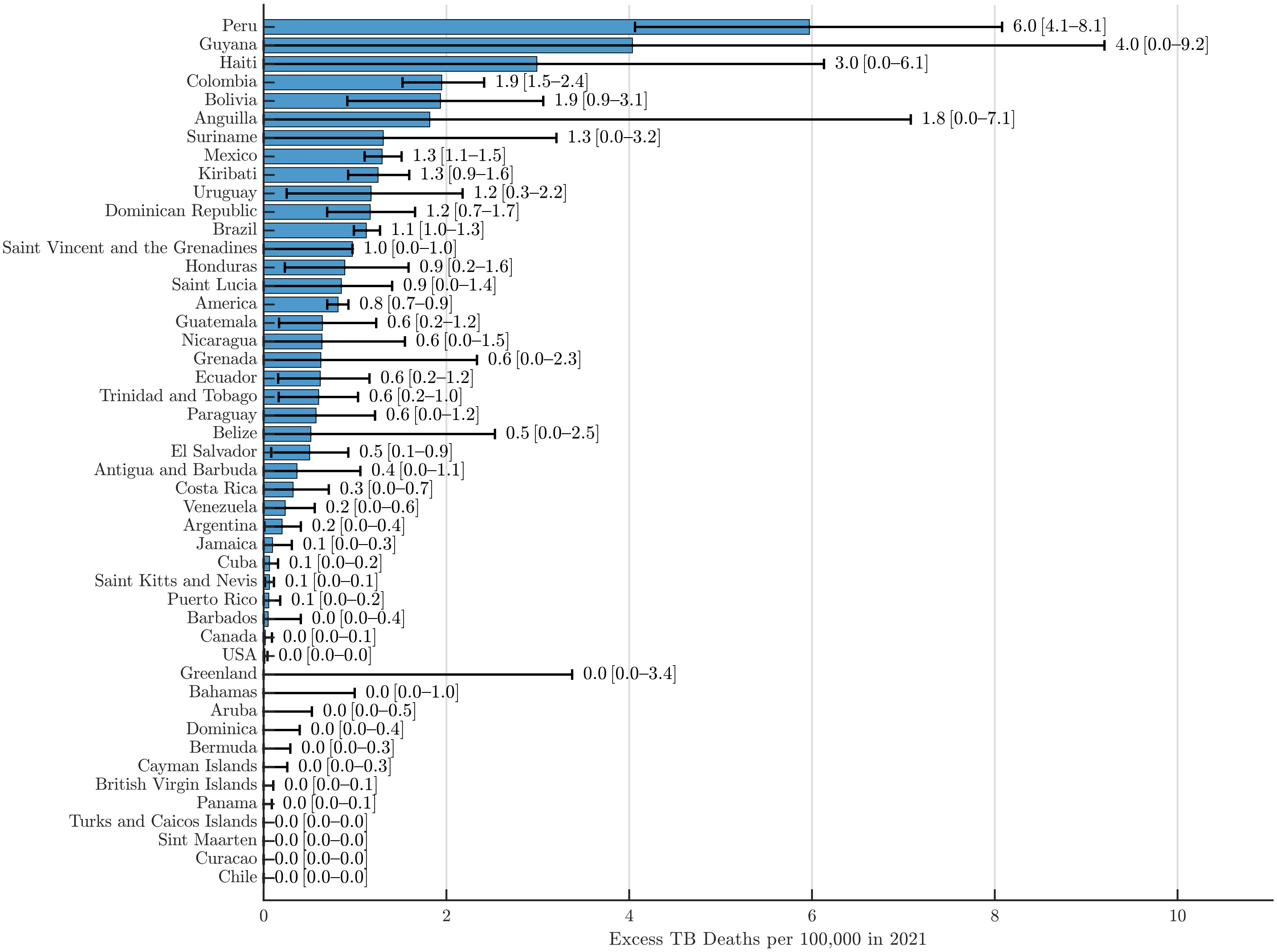


Figure S24: Excess TB mortality rate per 100,000 in the Americas in 2021, estimated using an ensemble framework with two sub-epidemics. The horizontal bar charts display the median excess mortality rate for each country, with horizontal lines extending from each bar representing the corresponding confidence intervals (lower and upper bounds). Exact mortality rate numbers are also provided for each country.

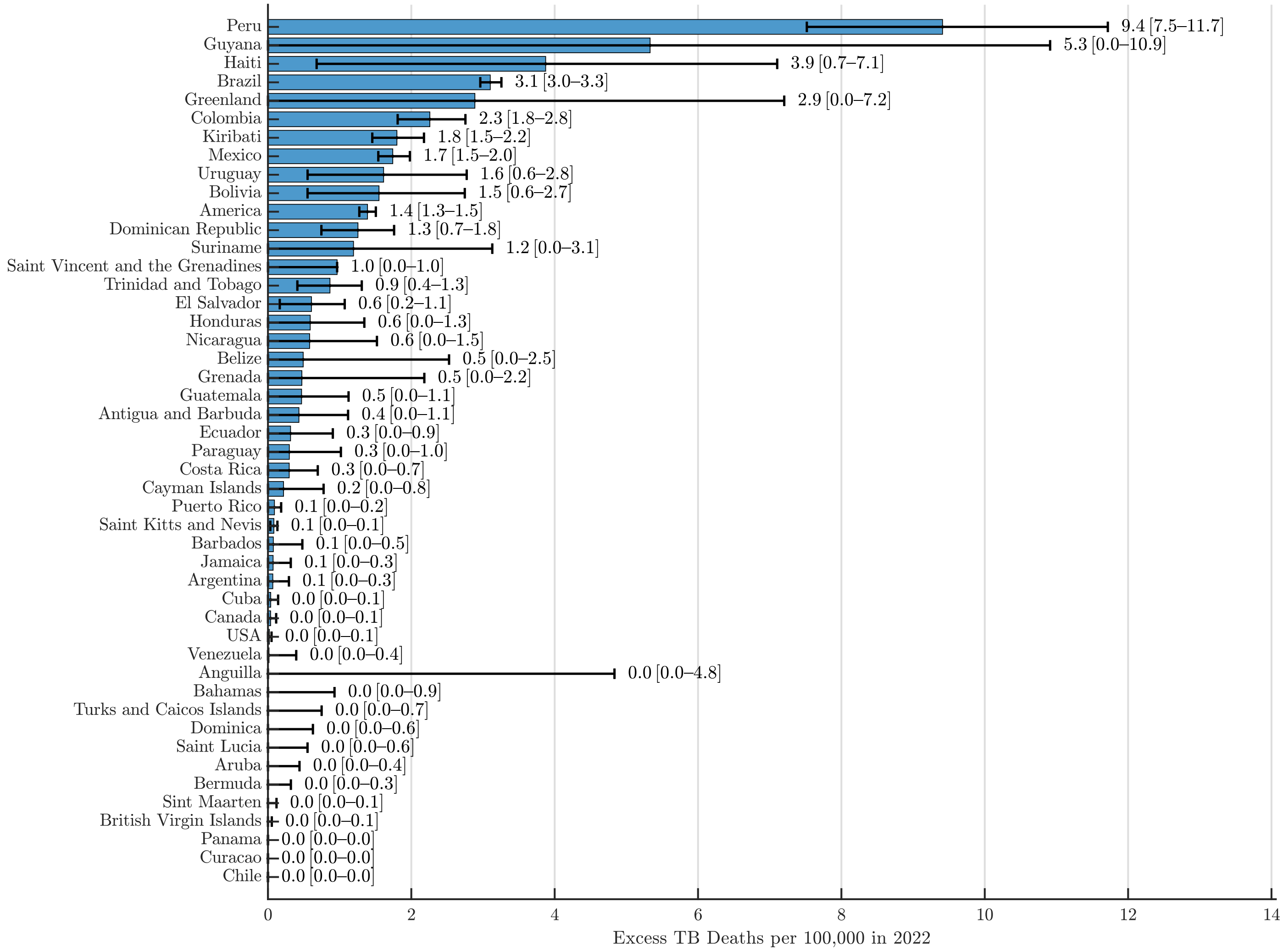


Figure S25: Excess TB mortality rate per 100,000 in the Americas in 2022, estimated using an ensemble framework with two sub-epidemics. The horizontal bar charts display the median excess mortality rate for each country, with horizontal lines extending from each bar representing the corresponding confidence intervals (lower and upper bounds). Exact mortality rate numbers are also provided for each country.

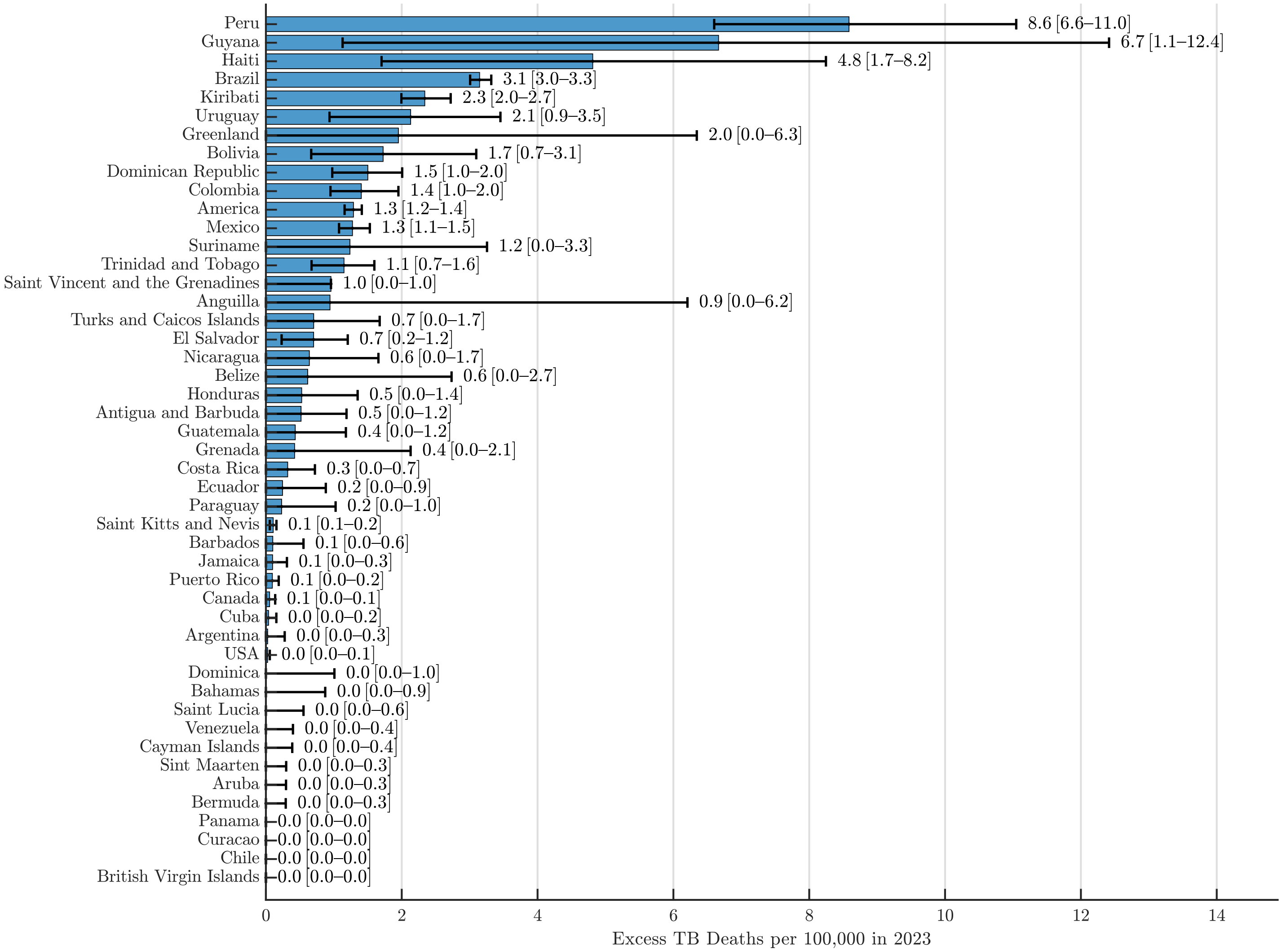


Figure S26: Excess TB mortality rate per 100,000 in the Americas in 2023, estimated using an ensemble framework with two sub-epidemics. The horizontal bar charts display the median excess mortality rate for each country, with horizontal lines extending from each bar representing the corresponding confidence intervals (lower and upper bounds). Exact mortality rate numbers are also provided for each country.

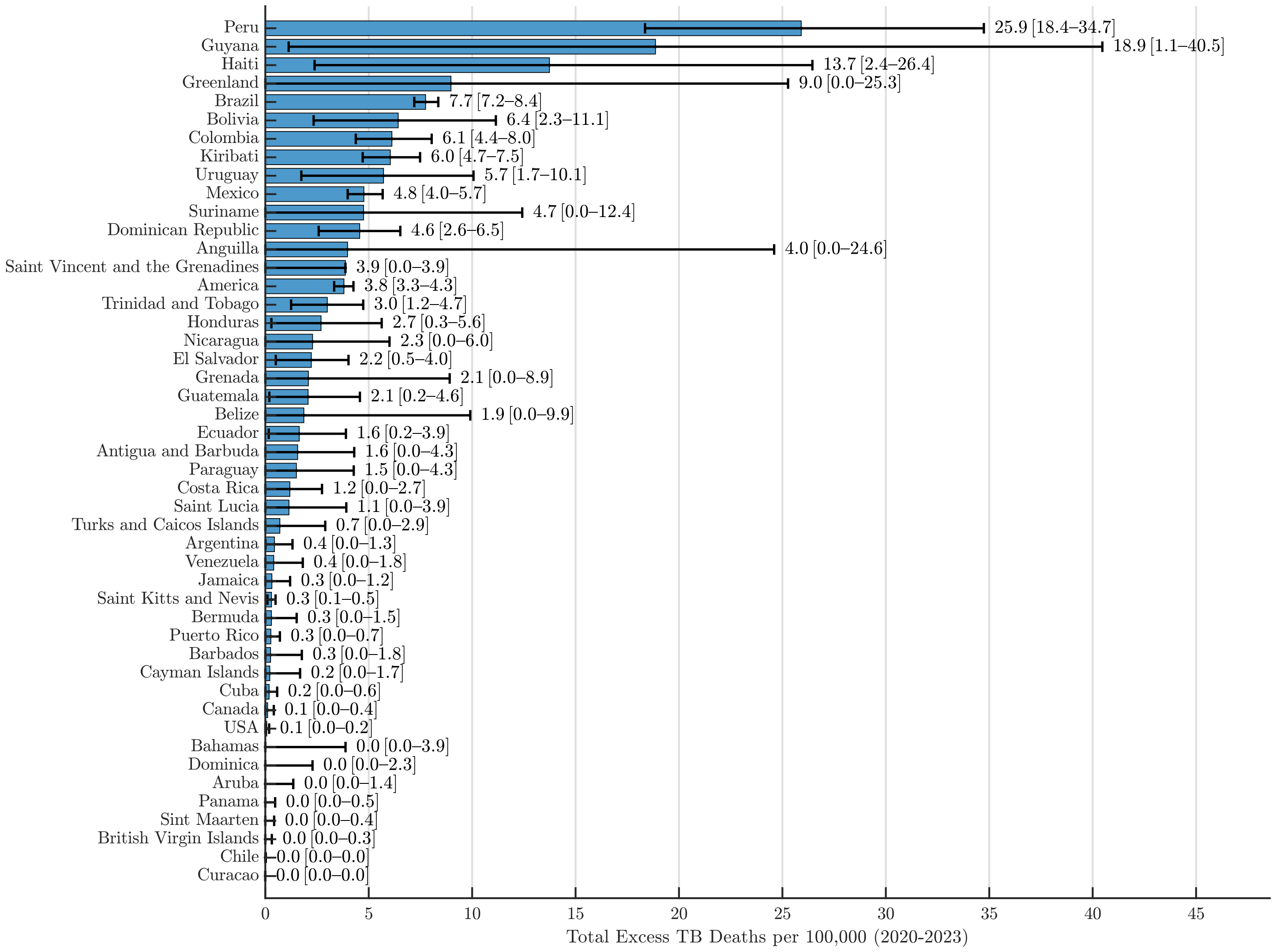


Figure S27: Total excess TB mortality rate per 100,000 in the Americas in 2020-2023, estimated using an ensemble framework with two sub-epidemics. The horizontal bar charts display the median excess mortality rate for each country, with horizontal lines extending from each bar representing the corresponding confidence intervals (lower and upper bounds). Exact mortality rate numbers are also provided for each country.

| | Excess TB mortality (LB,UB) | | | | |
|---|---|---|---|---|---|
| Country | **2020** | **2021** | **2022** | **2023** | **Total** |
| **Anguilla** | 1.2 (0.0, 6.5) | 1.8 (0.0, 7.1) | 0.0 (0.0, 4.8) | 0.9 (0.0, 6.2) | 4.0 (0.0, 24.6) |
| **Antigua and Barbuda** | 0.2 (0.0, 0.9) | 0.4 (0.0, 1.1) | 0.4 (0.0, 1.1) | 0.5 (0.0, 1.2) | 1.6 (0.0, 4.3) |
| **Argentina** | 0.1 (0.0, 0.3) | 0.2 (0.0, 0.4) | 0.1 (0.0, 0.3) | 0.0 (0.0, 0.3) | 0.4 (0.0, 1.3) |
| **Aruba** | 0.0 (0.0, 0.1) | 0.0 (0.0, 0.5) | 0.0 (0.0, 0.4) | 0.0 (0.0, 0.3) | 0.0 (0.0, 1.4) |
| **Bahamas** | 0.0 (0.0, 1.1) | 0.0 (0.0, 1.0) | 0.0 (0.0, 0.9) | 0.0 (0.0, 0.9) | 0.0 (0.0, 3.9) |
| **Barbados** | 0.0 (0.0, 0.3) | 0.0 (0.0, 0.4) | 0.1 (0.0, 0.5) | 0.1 (0.0, 0.6) | 0.3 (0.0, 1.8) |
| **Belize** | 0.2 (0.0, 2.1) | 0.5 (0.0, 2.5) | 0.5 (0.0, 2.5) | 0.6 (0.0, 2.7) | 1.9 (0.0, 9.9) |
| **Bermuda** | 0.3 (0.0, 0.6) | 0.0 (0.0, 0.3) | 0.0 (0.0, 0.3) | 0.0 (0.0, 0.3) | 0.3 (0.0, 1.5) |
| **Bolivia** | 1.2 (0.2, 2.2) | 1.9 (0.9, 3.1) | 1.5 (0.6, 2.7) | 1.7 (0.7, 3.1) | 6.4 (2.3, 11.1) |
| **Brazil** | 0.4 (0.2, 0.5) | 1.1 (1.0, 1.3) | 3.1 (3.0, 3.3) | 3.1 (3.0, 3.3) | 7.7 (7.2, 8.4) |
| **British Virgin Islands** | 0.0 (0.0, 0.2) | 0.0 (0.0, 0.1) | 0.0 (0.0, 0.1) | 0.0 (0.0, 0.0) | 0.0 (0.0, 0.3) |
| **Canada** | 0.0 (0.0, 0.1) | 0.0 (0.0, 0.1) | 0.0 (0.0, 0.1) | 0.1 (0.0, 0.1) | 0.1 (0.0, 0.4) |
| **Cayman Islands** | 0.0 (0.0, 0.3) | 0.0 (0.0, 0.3) | 0.2 (0.0, 0.8) | 0.0 (0.0, 0.4) | 0.2 (0.0, 1.7) |
| **Chile** | 0.0 (0.0, 0.0) | 0.0 (0.0, 0.0) | 0.0 (0.0, 0.0) | 0.0 (0.0, 0.0) | 0.0 (0.0, 0.0) |
| **Colombia** | 0.5 (0.1, 0.9) | 1.9 (1.5, 2.4) | 2.3 (1.8, 2.8) | 1.4 (1.0, 2.0) | 6.1 (4.4, 8.0) |
| **Costa Rica** | 0.2 (0.0, 0.6) | 0.3 (0.0, 0.7) | 0.3 (0.0, 0.7) | 0.3 (0.0, 0.7) | 1.2 (0.0, 2.7) |
| **Cuba** | 0.0 (0.0, 0.1) | 0.1 (0.0, 0.2) | 0.0 (0.0, 0.1) | 0.0 (0.0, 0.2) | 0.2 (0.0, 0.6) |
| **Curacao** | 0.0 (0.0, 0.0) | 0.0 (0.0, 0.0) | 0.0 (0.0, 0.0) | 0.0 (0.0, 0.0) | 0.0 (0.0, 0.0) |
| **Dominica** | 0.0 (0.0, 0.3) | 0.0 (0.0, 0.4) | 0.0 (0.0, 0.6) | 0.0 (0.0, 1.0) | 0.0 (0.0, 2.3) |
| **Dominican Republic** | 0.6 (0.2, 1.1) | 1.2 (0.7, 1.7) | 1.3 (0.7, 1.8) | 1.5 (1.0, 2.0) | 4.6 (2.6, 6.5) |
| **Ecuador** | 0.5 (0.0, 1.0) | 0.6 (0.2, 1.2) | 0.3 (0.0, 0.9) | 0.2 (0.0, 0.9) | 1.6 (0.2, 3.9) |
| **El Salvador** | 0.4 (0.0, 0.8) | 0.5 (0.1, 0.9) | 0.6 (0.2, 1.1) | 0.7 (0.2, 1.2) | 2.2 (0.5, 4.0) |
| **Greenland** | 4.1 (0.0, 8.4) | 0.0 (0.0, 3.4) | 2.9 (0.0, 7.2) | 2.0 (0.0, 6.3) | 9.0 (0.0, 25.3) |
| **Grenada** | 0.6 (0.0, 2.3) | 0.6 (0.0, 2.3) | 0.5 (0.0, 2.2) | 0.4 (0.0, 2.1) | 2.1 (0.0, 8.9) |
| **Guatemala** | 0.5 (0.0, 1.0) | 0.6 (0.2, 1.2) | 0.5 (0.0, 1.1) | 0.4 (0.0, 1.2) | 2.1 (0.2, 4.6) |
| **Guyana** | 2.8 (0.0, 7.9) | 4.0 (0.0, 9.2) | 5.3 (0.0, 10.9) | 6.7 (1.1, 12.4) | 18.9 (1.1, 40.5) |
| **Haiti** | 2.1 (0.0, 5.0) | 3.0 (0.0, 6.1) | 3.9 (0.7, 7.1) | 4.8 (1.7, 8.2) | 13.7 (2.4, 26.4) |
| **Honduras** | 0.7 (0.1, 1.3) | 0.9 (0.2, 1.6) | 0.6 (0.0, 1.3) | 0.5 (0.0, 1.4) | 2.7 (0.3, 5.6) |
| **Jamaica** | 0.1 (0.0, 0.3) | 0.1 (0.0, 0.3) | 0.1 (0.0, 0.3) | 0.1 (0.0, 0.3) | 0.3 (0.0, 1.2) |
| **Kiribati** | 0.7 (0.3, 1.0) | 1.3 (0.9, 1.6) | 1.8 (1.5, 2.2) | 2.3 (2.0, 2.7) | 6.0 (4.7, 7.5) |
| **Mexico** | 0.5 (0.3, 0.7) | 1.3 (1.1, 1.5) | 1.7 (1.5, 2.0) | 1.3 (1.1, 1.5) | 4.8 (4.0, 5.7) |
| **Nicaragua** | 0.4 (0.0, 1.3) | 0.6 (0.0, 1.5) | 0.6 (0.0, 1.5) | 0.6 (0.0, 1.7) | 2.3 (0.0, 6.0) |
| **Panama** | 0.0 (0.0, 0.4) | 0.0 (0.0, 0.1) | 0.0 (0.0, 0.0) | 0.0 (0.0, 0.0) | 0.0 (0.0, 0.5) |
| **Paraguay** | 0.4 (0.0, 1.0) | 0.6 (0.0, 1.2) | 0.3 (0.0, 1.0) | 0.2 (0.0, 1.0) | 1.5 (0.0, 4.3) |
| **Peru** | 1.9 (0.2, 3.9) | 6.0 (4.1, 8.1) | 9.4 (7.5, 11.7) | 8.6 (6.6, 11.0) | 25.9 (18.4, 34.7) |
| **Puerto Rico** | 0.0 (0.0, 0.2) | 0.1 (0.0, 0.2) | 0.1 (0.0, 0.2) | 0.1 (0.0, 0.2) | 0.3 (0.0, 0.7) |
| **Saint Kitts and Nevis** | 0.0 (0.0, 0.1) | 0.1 (0.0, 0.1) | 0.1 (0.0, 0.1) | 0.1 (0.1, 0.2) | 0.3 (0.1, 0.5) |
| **Saint Lucia** | 0.3 (0.0, 1.4) | 0.9 (0.0, 1.4) | 0.0 (0.0, 0.6) | 0.0 (0.0, 0.6) | 1.1 (0.0, 3.9) |
| **Saint Vincent and the Grenadines** | 1.0 (0.0, 1.0) | 1.0 (0.0, 1.0) | 1.0 (0.0, 1.0) | 1.0 (0.0, 1.0) | 3.9 (0.0, 3.9) |
| **Sint Maarten** | 0.0 (0.0, 0.0) | 0.0 (0.0, 0.0) | 0.0 (0.0, 0.1) | 0.0 (0.0, 0.3) | 0.0 (0.0, 0.4) |
| **Suriname** | 1.0 (0.0, 2.8) | 1.3 (0.0, 3.2) | 1.2 (0.0, 3.1) | 1.2 (0.0, 3.3) | 4.7 (0.0, 12.4) |
| **Trinidad and Tobago** | 0.4 (0.0, 0.8) | 0.6 (0.2, 1.0) | 0.9 (0.4, 1.3) | 1.1 (0.7, 1.6) | 3.0 (1.2, 4.7) |
| **Turks and Caicos Islands** | 0.0 (0.0, 0.5) | 0.0 (0.0, 0.0) | 0.0 (0.0, 0.7) | 0.7 (0.0, 1.7) | 0.7 (0.0, 2.9) |
| **USA** | 0.0 (0.0, 0.0) | 0.0 (0.0, 0.0) | 0.0 (0.0, 0.1) | 0.0 (0.0, 0.1) | 0.1 (0.0, 0.2) |
| **Uruguay** | 0.8 (0.0, 1.7) | 1.2 (0.3, 2.2) | 1.6 (0.6, 2.8) | 2.1 (0.9, 3.5) | 5.7 (1.7, 10.1) |
| **Venezuela** | 0.2 (0.0, 0.5) | 0.2 (0.0, 0.6) | 0.0 (0.0, 0.4) | 0.0 (0.0, 0.4) | 0.4 (0.0, 1.8) |
| **America** | 0.3 (0.2, 0.4) | 0.8 (0.7, 0.9) | 1.4 (1.3, 1.5) | 1.3 (1.2, 1.4) | 3.8 (3.3, 4.3) |

Table S4: Estimated excess TB mortality rate in the Americas for the years 2020 to 2023, presented both individually by year and as a total. The estimates include the median value along with the corresponding lower and upper bounds, calculated using an ensemble framework that models two distinct sub-epidemics.

## S2.3 SMR

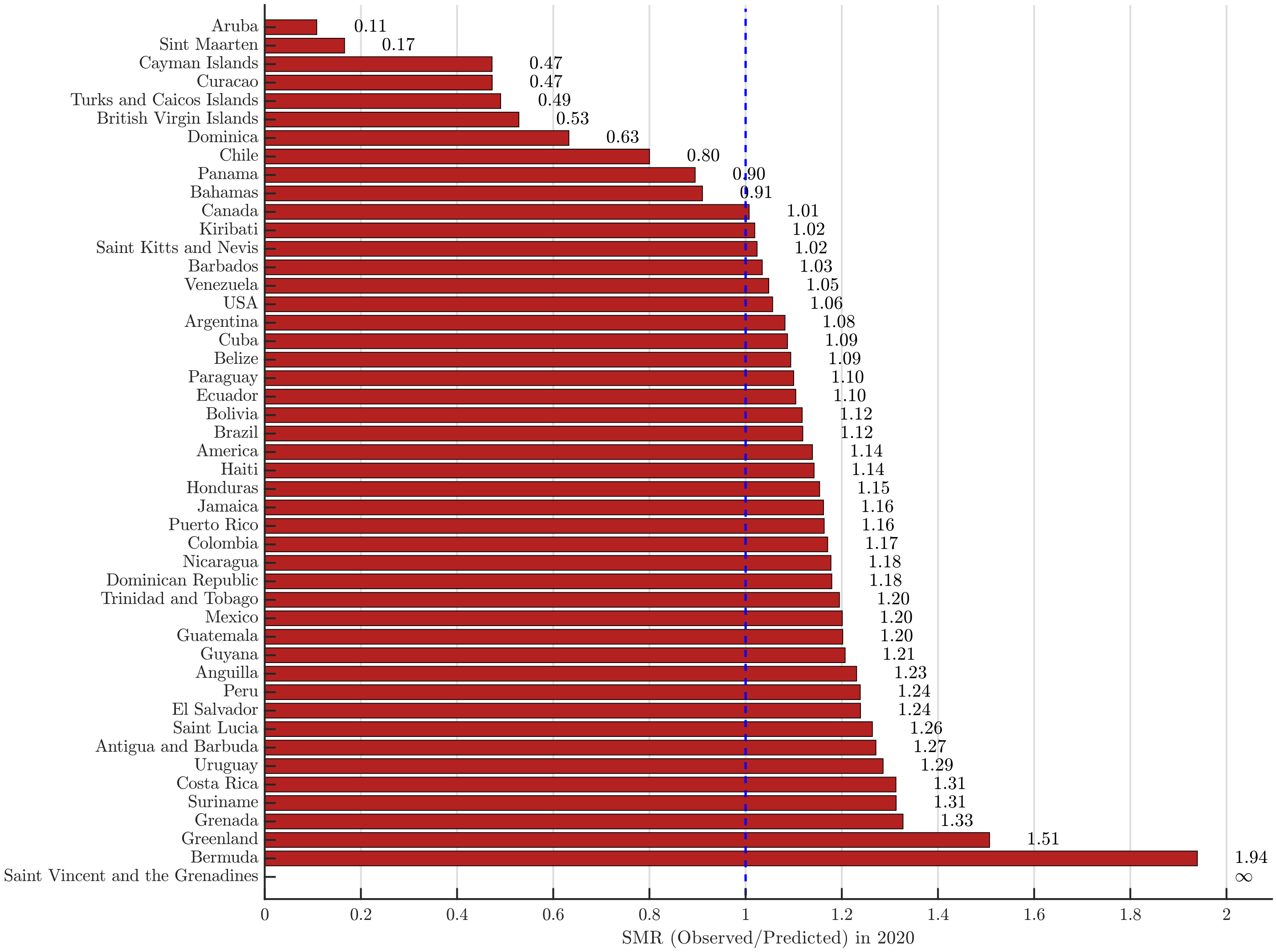


Figure S28: SMR in the Americas in 2020, estimated using an ensemble framework with two sub-epidemics. SMR is displayed horizontally in bar charts, with the exact number displayed in front of the bars.

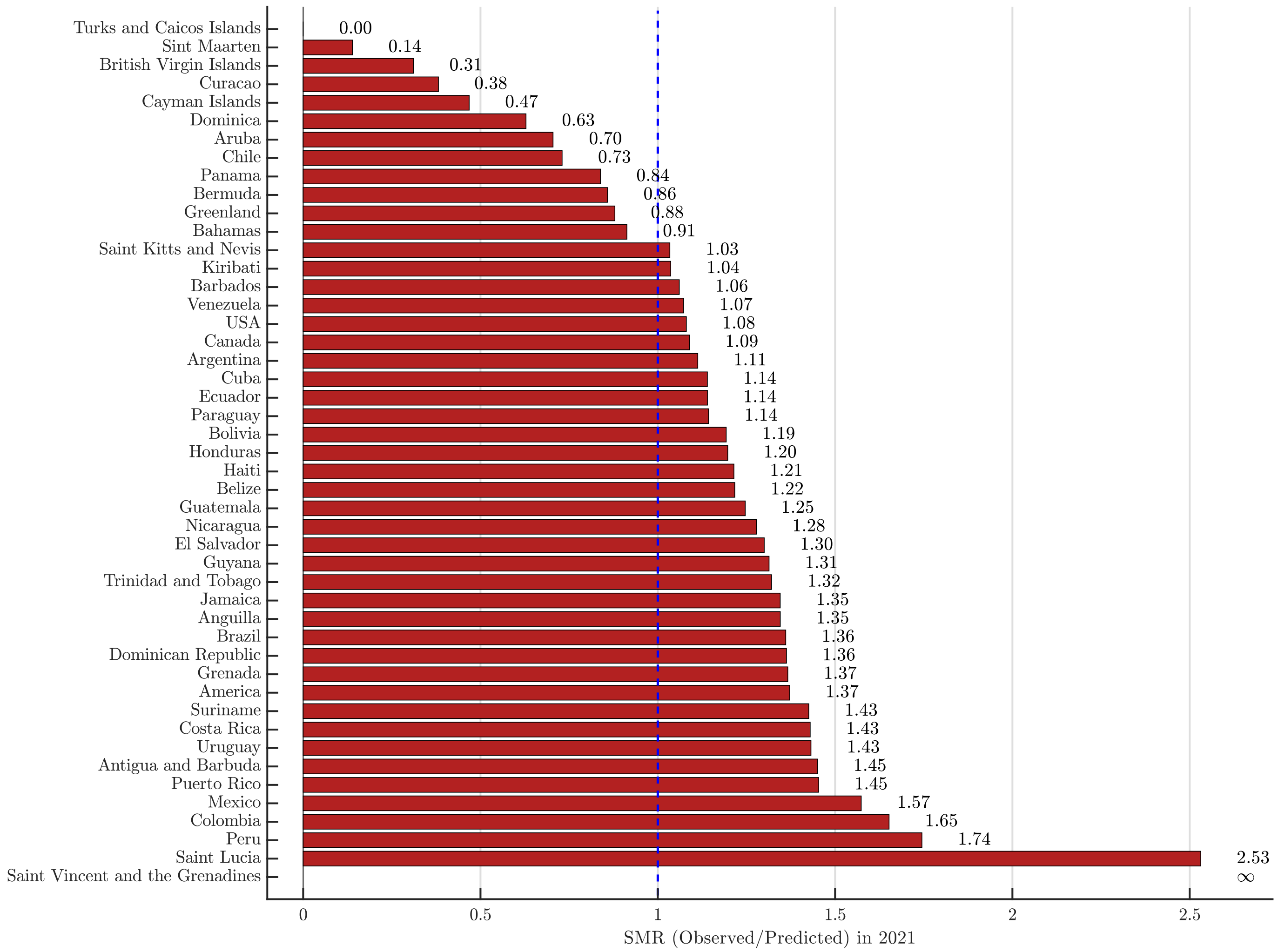


Figure S29: SMR in the Americas in 2021, estimated using an ensemble framework with two sub-epidemics. SMR is displayed horizontally in bar charts, with the exact number displayed in front of the bars.

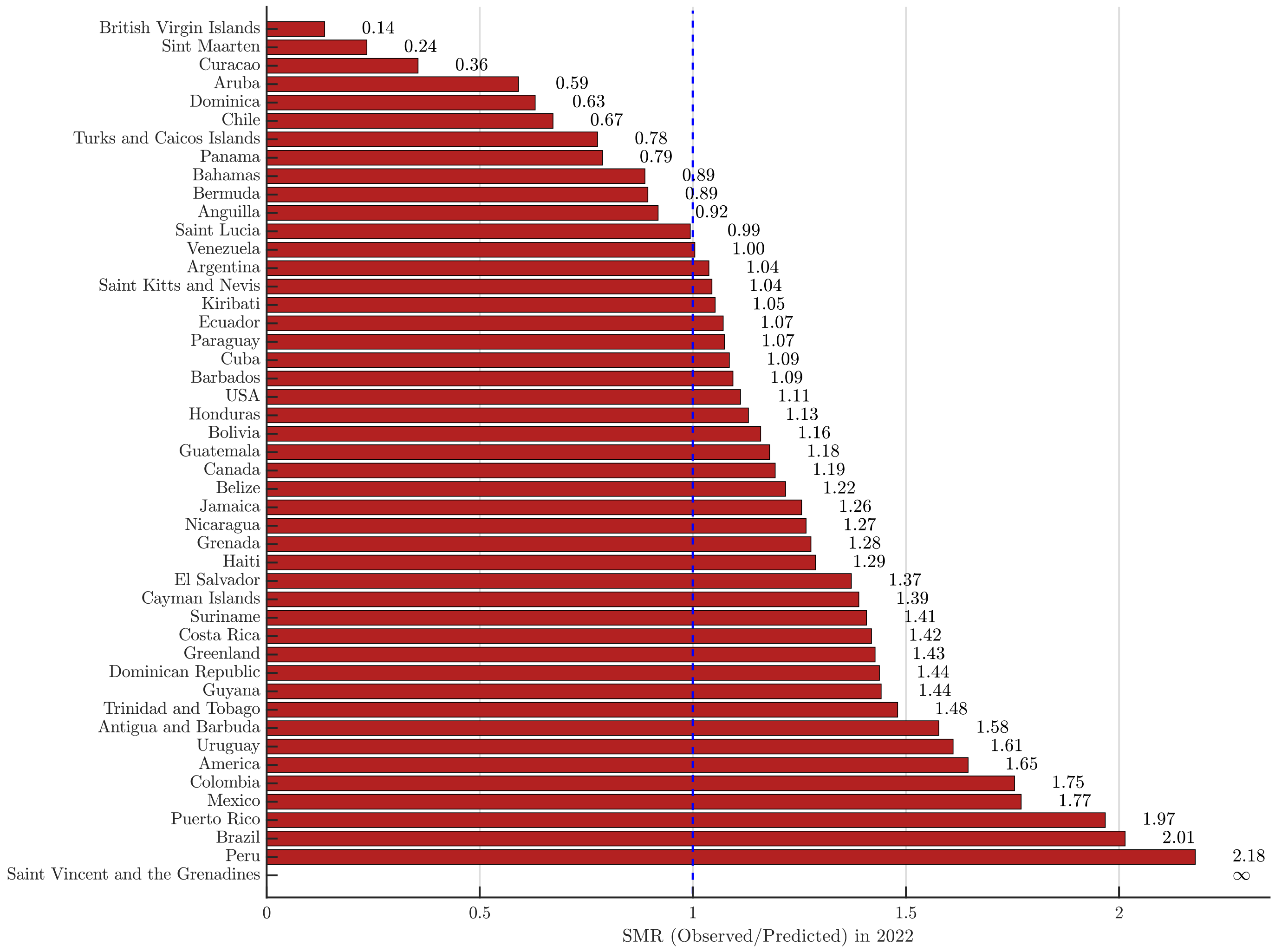


Figure S30: SMR in the Americas in 2022, estimated using an ensemble framework with two sub-epidemics. SMR is displayed horizontally in bar charts, with the exact number displayed in front of the bars.

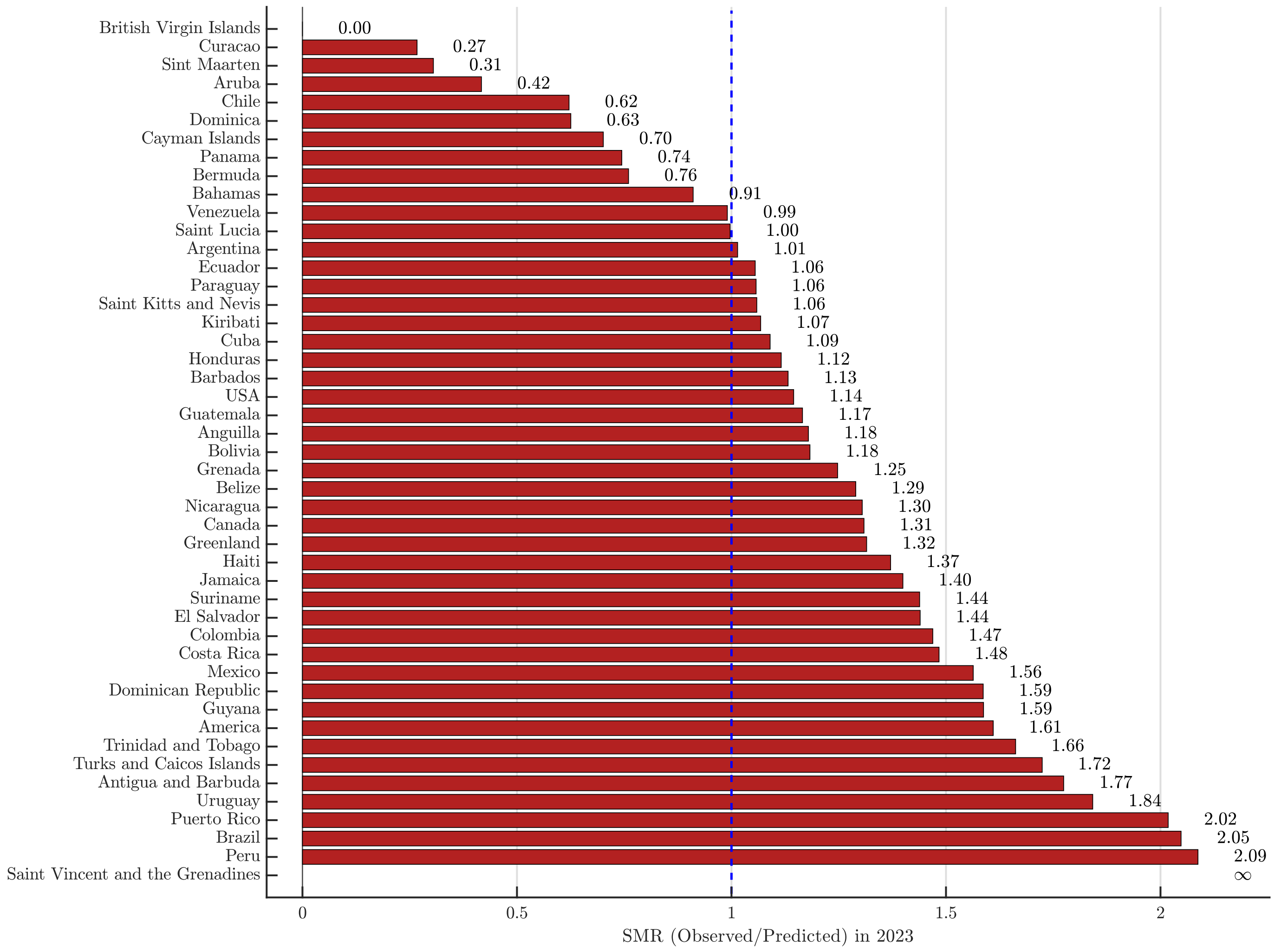


Figure S31: SMR in the Americas in 2023, estimated using an ensemble framework with two sub-epidemics. SMR is displayed horizontally in bar charts, with the exact number displayed in front of the bars.

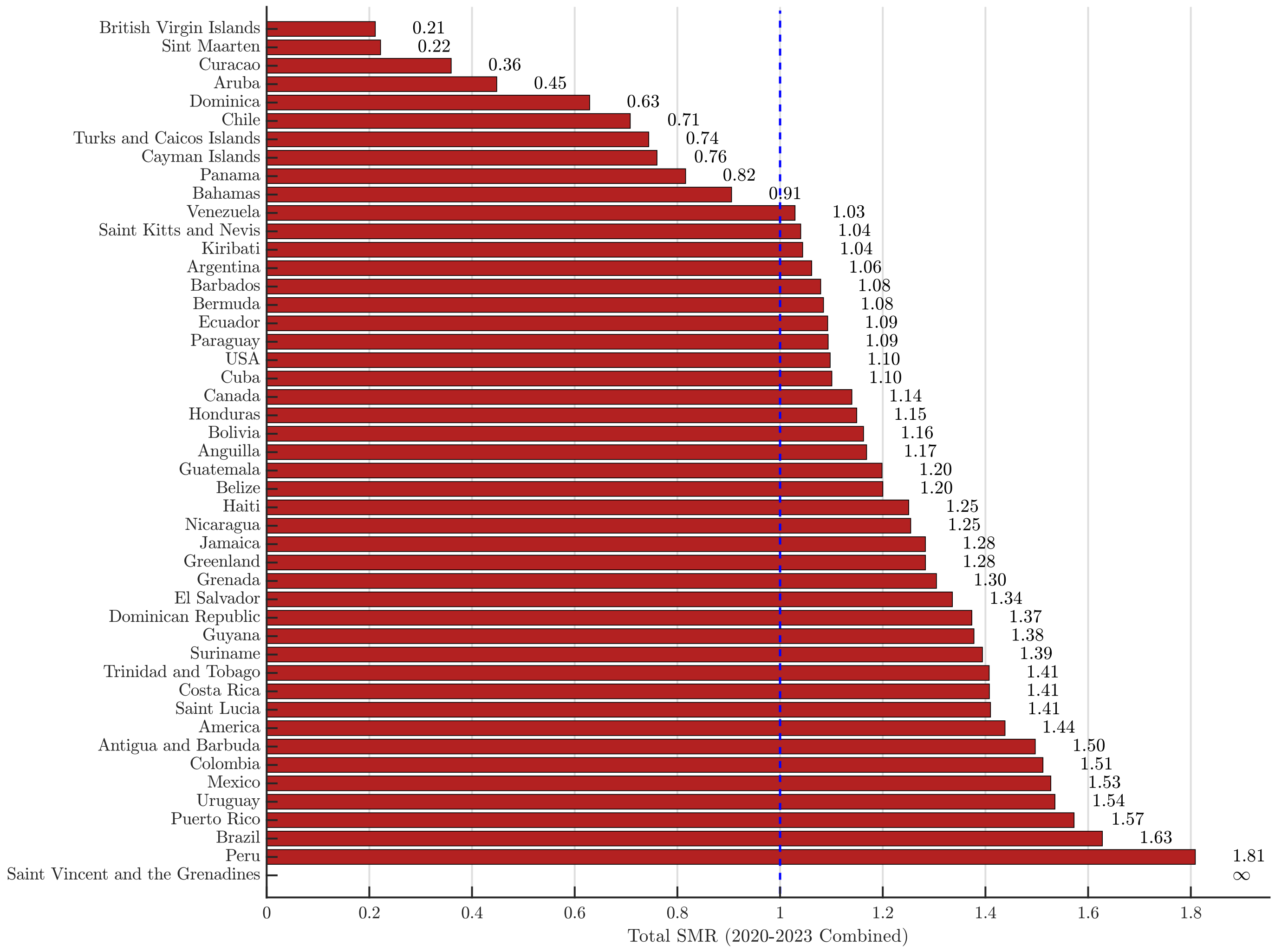


Figure S32: SMR in the Americas during 2020-2023, estimated using an ensemble framework with two sub-epidemics. SMR is displayed horizontally in bar charts, with the exact number displayed in front of the bars.

## S3 Pacific

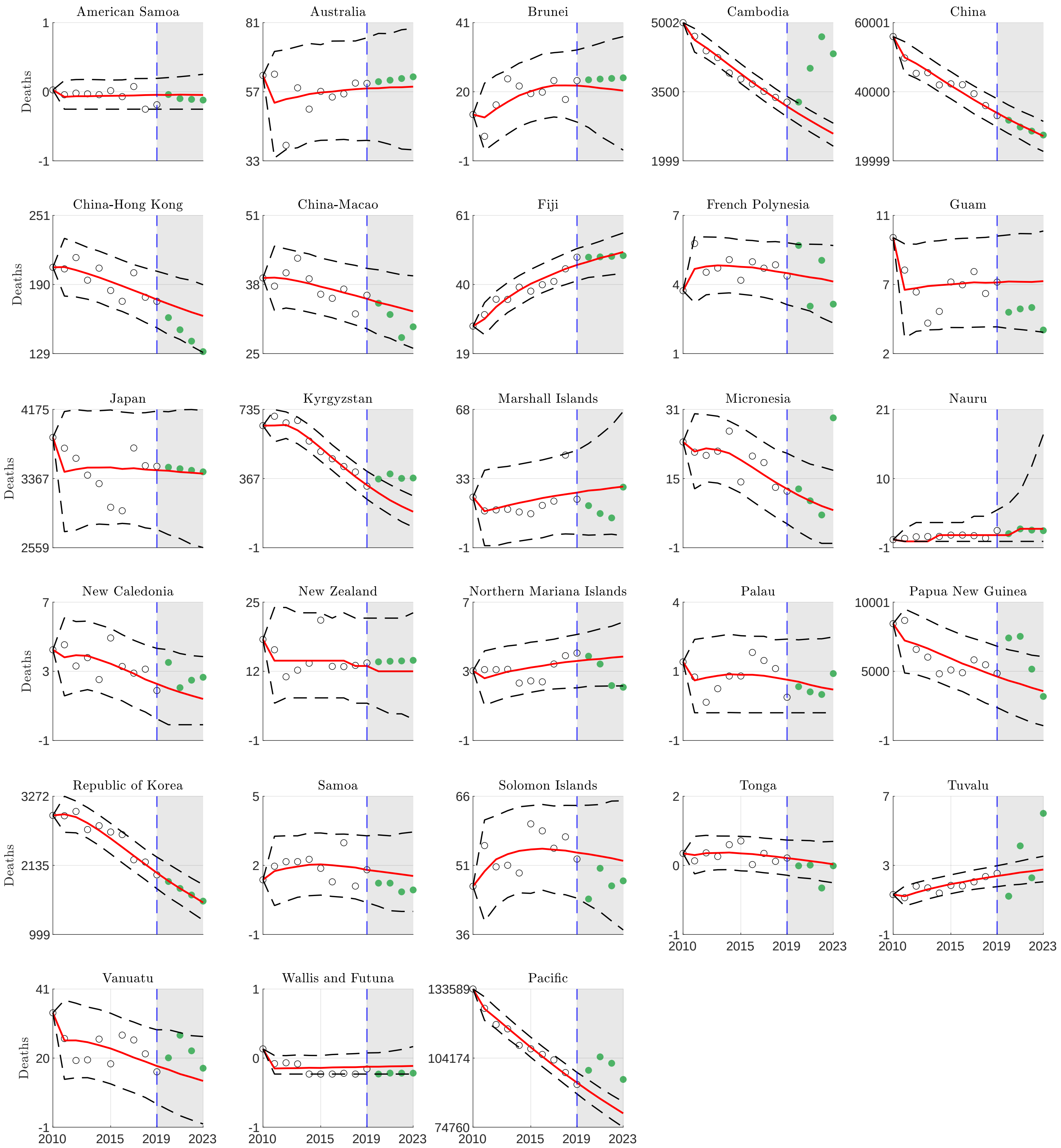


Figure S33: Forecasting panel for the number of deaths in the Pacific, based on a 10-year calibration period (2010–2019) and a four-year forecasting period (2020–2023), generated using the Ranked 1 method. The red curve denotes the median forecast, while the black dashed lines mark the 95% PI. Reported data points are shown as circles: black circles represent the calibration period, and green-filled circles represent actual data points during the forecast period.

## S3.1 Excess mortality

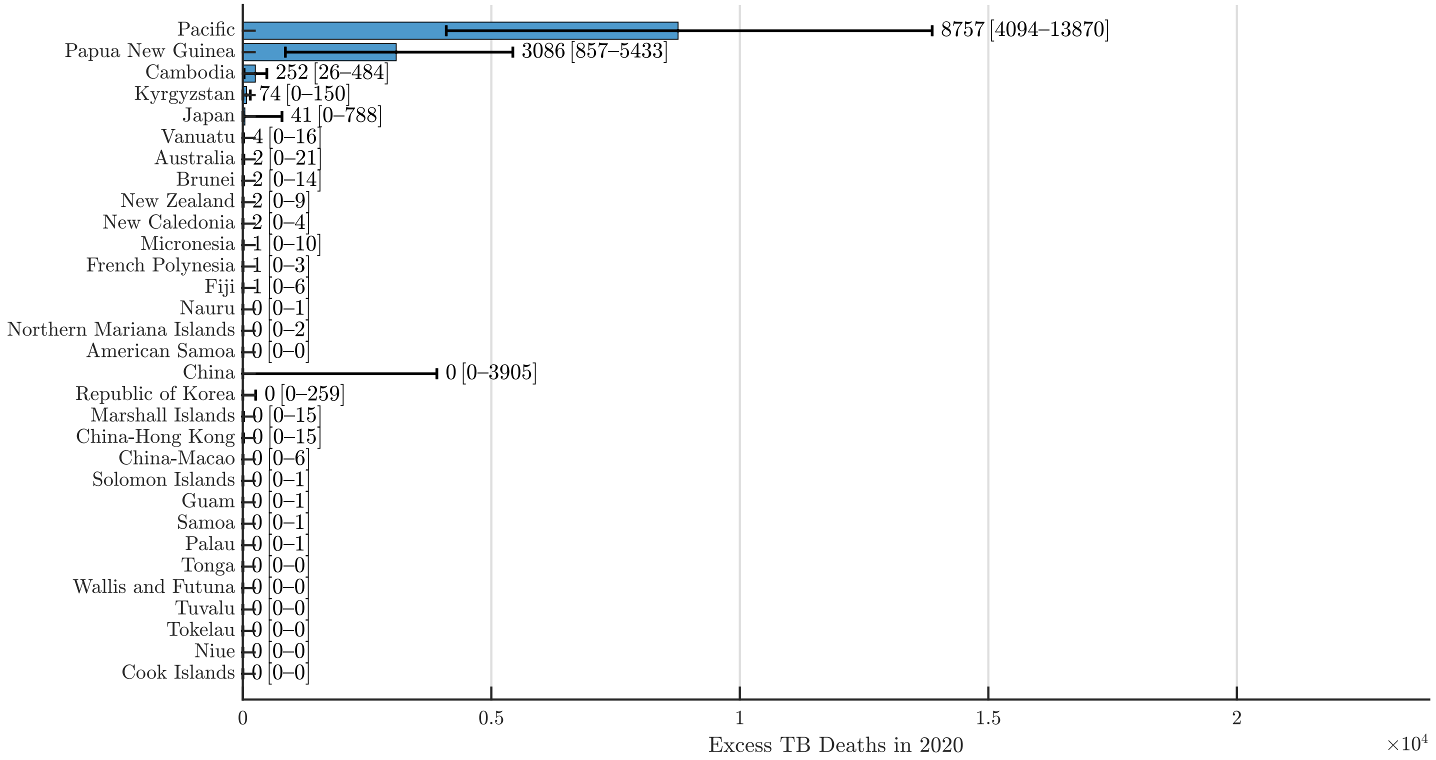


Figure S34: Excess TB mortality in Pacific in 2020, estimated using an ensemble framework with two sub-epidemics. The horizontal bar charts display the median excess mortality for each country, with horizontal lines extending from each bar representing the corresponding confidence intervals (lower and upper bounds). Exact mortality numbers are also provided for each country.

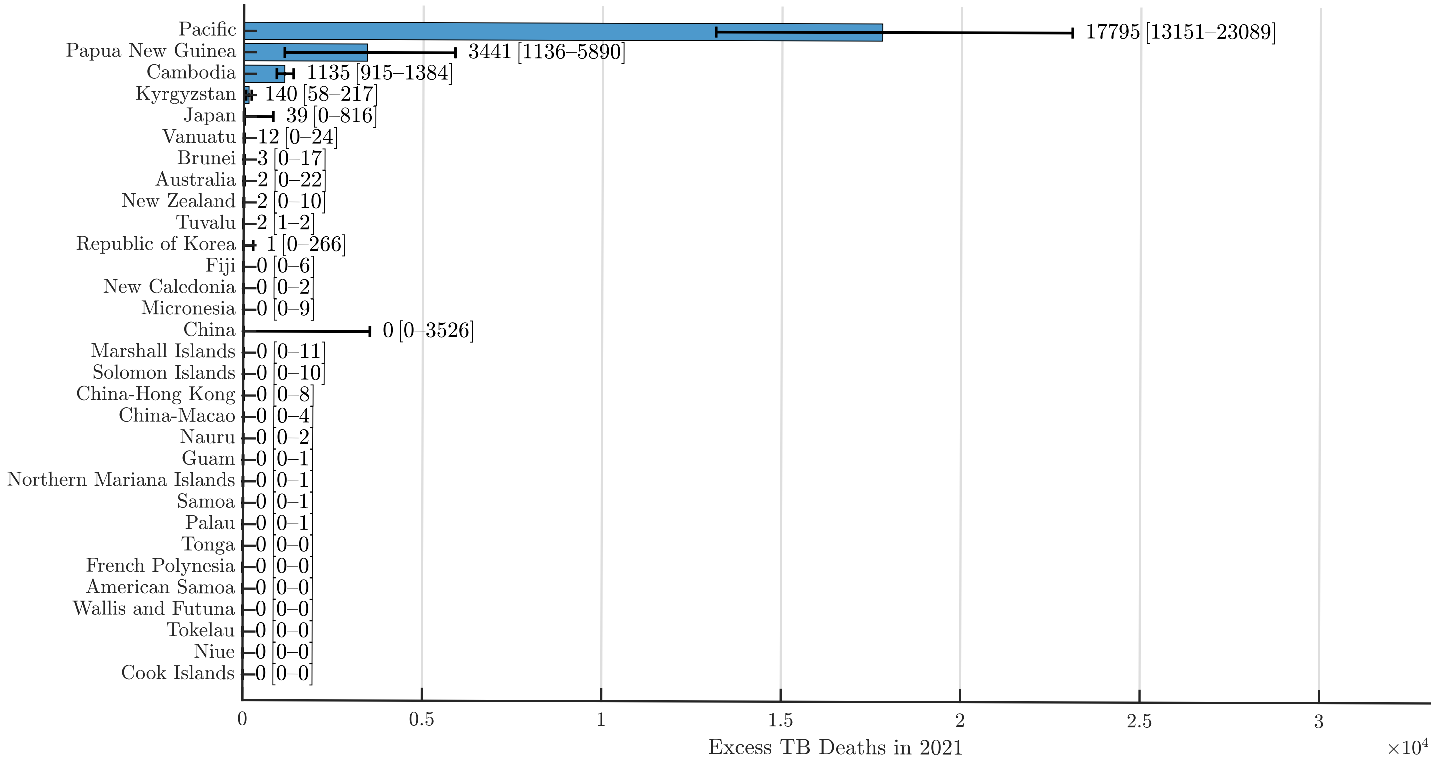


Figure S35: Excess TB mortality in Pacific in 2021, estimated using an ensemble framework with two sub-epidemics. The horizontal bar charts display the median excess mortality for each country, with horizontal lines extending from each bar representing the corresponding confidence intervals (lower and upper bounds). Exact mortality numbers are also provided for each country.

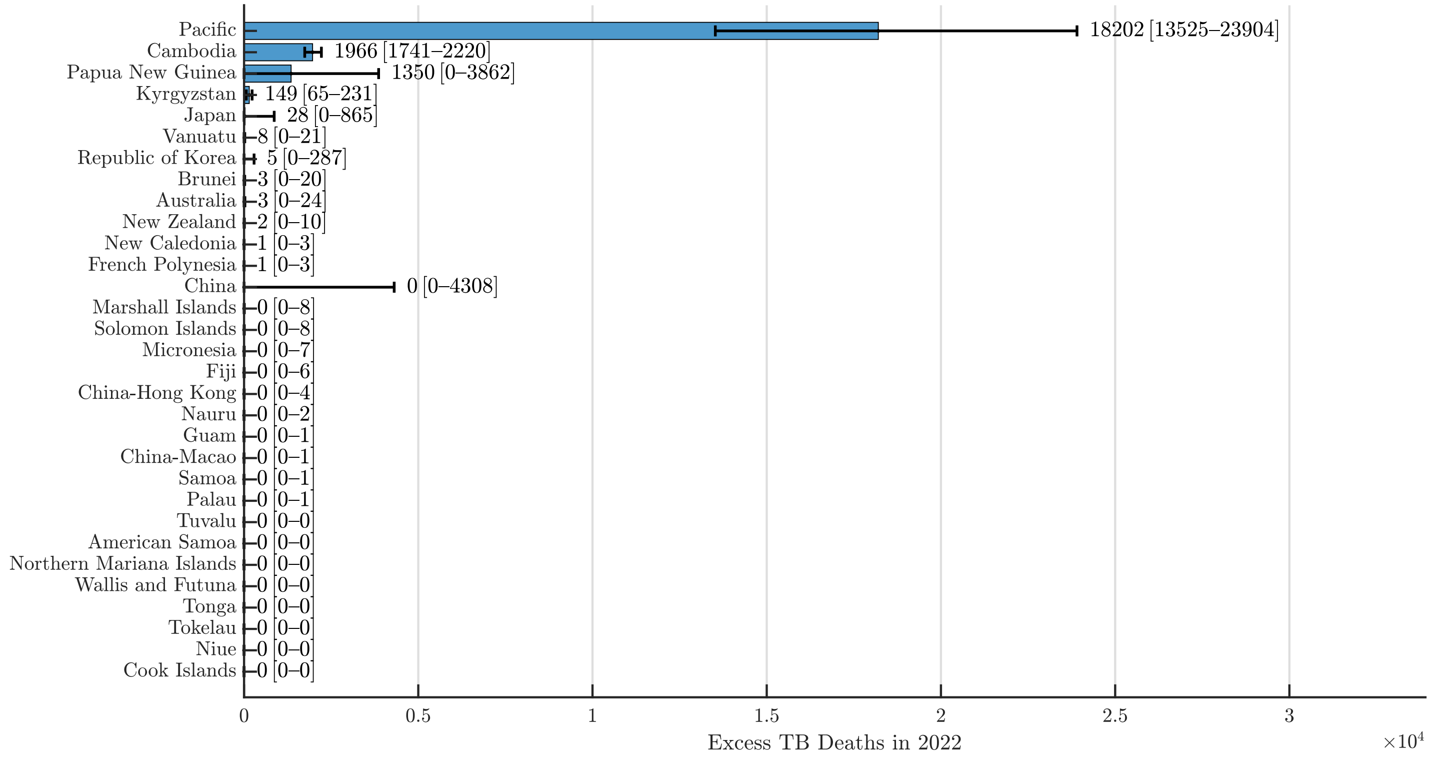


Figure S36: Excess TB mortality in Pacific in 2022, estimated using an ensemble framework with two sub-epidemics. The horizontal bar charts display the median excess mortality for each country, with horizontal lines extending from each bar representing the corresponding confidence intervals (lower and upper bounds). Exact mortality numbers are also provided for each country.

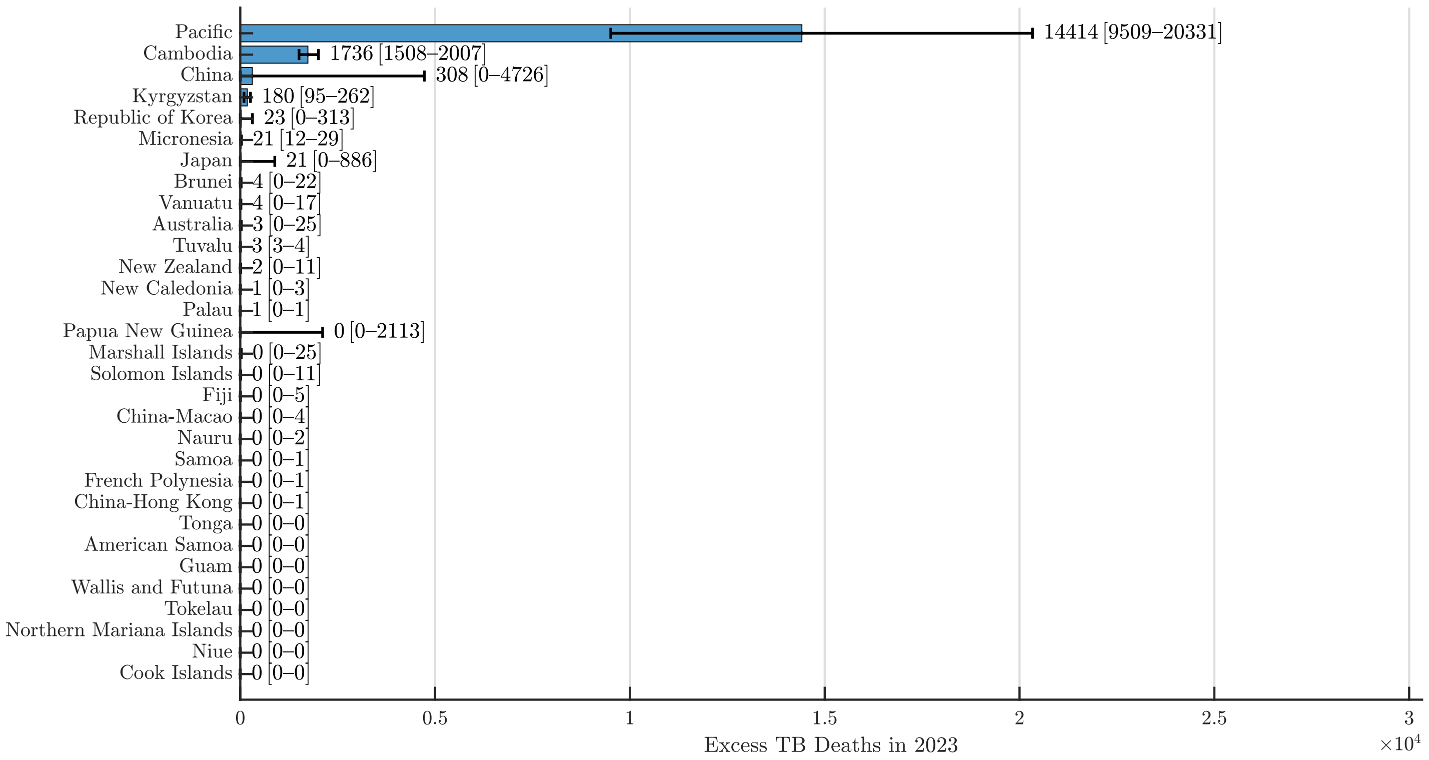


Figure S37: Excess TB mortality in Pacific in 2023, estimated using an ensemble framework with two sub-epidemics. The horizontal bar charts display the median excess mortality for each country, with horizontal lines extending from each bar representing the corresponding confidence intervals (lower and upper bounds). Exact mortality numbers are also provided for each country.

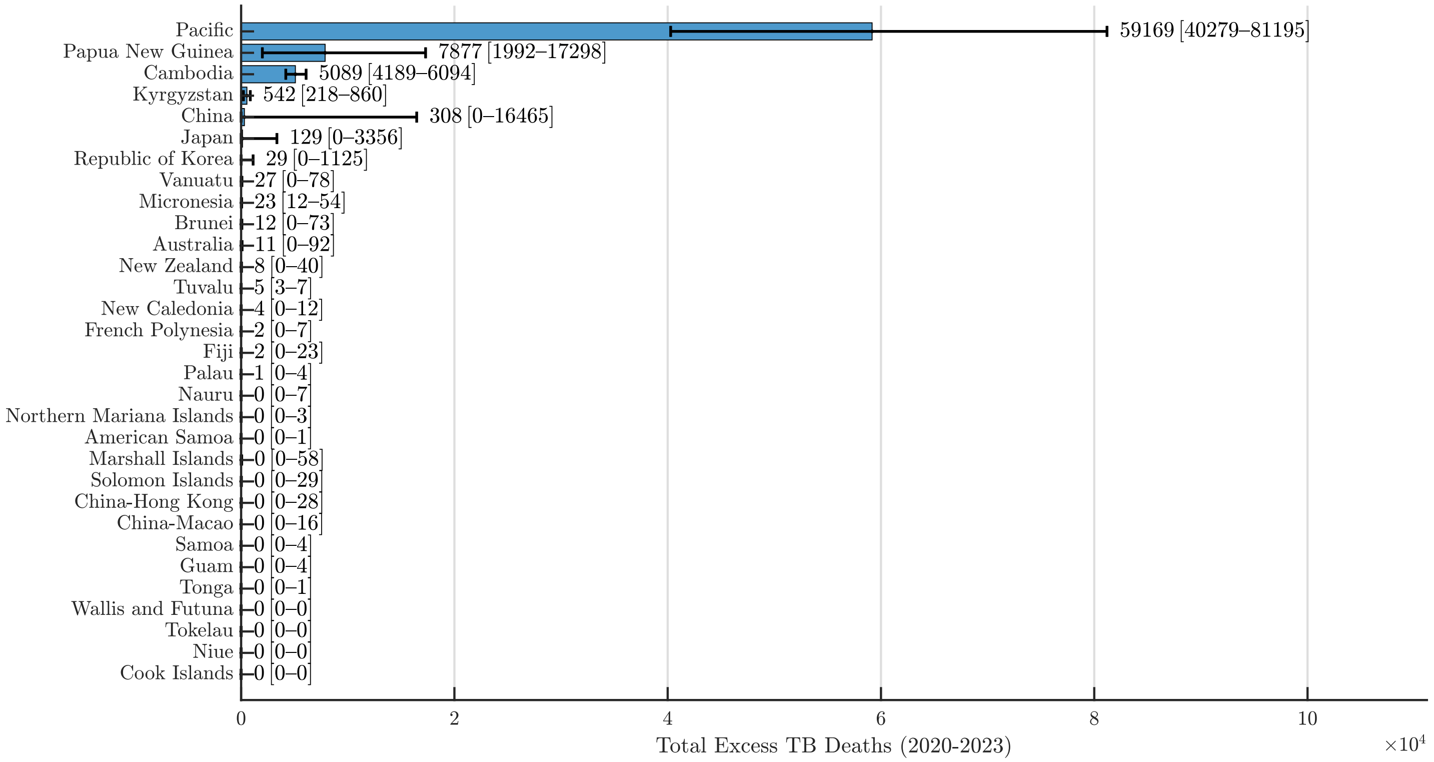


Figure S38: Total excess TB mortality in Pacific in 2020-2023, estimated using an ensemble framework with two sub-epidemics. The horizontal bar charts display the median excess mortality for each country, with horizontal lines extending from each bar representing the corresponding confidence intervals (lower and upper bounds). Exact mortality numbers are also provided for each country.

| | Excess TB mortality (LB,UB) | | | | |
|---|---|---|---|---|---|
| Country | **2020** | **2021** | **2022** | **2023** | **Total** |
| **American Samoa** | 0 (0, 0) | 0 (0, 0) | 0 (0, 0) | 0 (0, 0) | 0 (0, 1) |
| **Australia** | 2 (0, 21) | 2 (0, 22) | 3 (0, 24) | 3 (0, 25) | 11 (0, 92) |
| **Brunei** | 2 (0, 14) | 3 (0, 17) | 3 (0, 20) | 4 (0, 22) | 12 (0, 73) |
| **Cambodia** | 252 (26, 484) | 1135 (915, 1384) | 1966 (1741, 2220) | 1736 (1508, 2007) | 5089 (4189, 6094) |
| **China** | 0 (0, 3905) | 0 (0, 3526) | 0 (0, 4308) | 308 (0, 4726) | 308 (0, 16465) |
| **China-Hong Kong** | 0 (0, 15) | 0 (0, 8) | 0 (0, 4) | 0 (0, 1) | 0 (0, 28) |
| **China-Macao** | 0 (0, 6) | 0 (0, 4) | 0 (0, 1) | 0 (0, 4) | 0 (0, 16) |
| **Fiji** | 1 (0, 6) | 0 (0, 6) | 0 (0, 6) | 0 (0, 5) | 2 (0, 23) |
| **French Polynesia** | 1 (0, 3) | 0 (0, 0) | 1 (0, 3) | 0 (0, 1) | 2 (0, 7) |
| **Guam** | 0 (0, 1) | 0 (0, 1) | 0 (0, 1) | 0 (0, 0) | 0 (0, 4) |
| **Japan** | 41 (0, 788) | 39 (0, 816) | 28 (0, 865) | 21 (0, 886) | 129 (0, 3356) |
| **Kyrgyzstan** | 74 (0, 150) | 140 (58, 217) | 149 (65, 231) | 180 (95, 262) | 542 (218, 860) |
| **Marshall Islands** | 0 (0, 15) | 0 (0, 11) | 0 (0, 8) | 0 (0, 25) | 0 (0, 58) |
| **Micronesia** | 1 (0, 10) | 0 (0, 9) | 0 (0, 7) | 21 (12, 29) | 23 (12, 54) |
| **Nauru** | 0 (0, 1) | 0 (0, 2) | 0 (0, 2) | 0 (0, 2) | 0 (0, 7) |
| **New Caledonia** | 2 (0, 4) | 0 (0, 2) | 1 (0, 3) | 1 (0, 3) | 4 (0, 12) |
| **New Zealand** | 2 (0, 9) | 2 (0, 10) | 2 (0, 10) | 2 (0, 11) | 8 (0, 40) |
| **Northern Mariana Islands** | 0 (0, 2) | 0 (0, 1) | 0 (0, 0) | 0 (0, 0) | 0 (0, 3) |
| **Palau** | 0 (0, 1) | 0 (0, 1) | 0 (0, 1) | 1 (0, 1) | 1 (0, 4) |
| **Papua New Guinea** | 3086 (857, 5433) | 3441 (1136, 5890) | 1350 (0, 3862) | 0 (0, 2113) | 7877 (1992, 17298) |
| **Republic of Korea** | 0 (0, 259) | 1 (0, 266) | 5 (0, 287) | 23 (0, 313) | 29 (0, 1125) |
| **Samoa** | 0 (0, 1) | 0 (0, 1) | 0 (0, 1) | 0 (0, 1) | 0 (0, 4) |
| **Solomon Islands** | 0 (0, 1) | 0 (0, 10) | 0 (0, 8) | 0 (0, 11) | 0 (0, 29) |
| **Tonga** | 0 (0, 0) | 0 (0, 0) | 0 (0, 0) | 0 (0, 0) | 0 (0, 1) |
| **Tuvalu** | 0 (0, 0) | 2 (1, 2) | 0 (0, 0) | 3 (3, 4) | 5 (3, 7) |
| **Vanuatu** | 4 (0, 16) | 12 (0, 24) | 8 (0, 21) | 4 (0, 17) | 27 (0, 78) |
| **Wallis and Futuna** | 0 (0, 0) | 0 (0, 0) | 0 (0, 0) | 0 (0, 0) | 0 (0, 0) |
| **Pacific** | 8757 (4094, 13870) | 17795 (13151, 23089) | 18202 (13525, 23904) | 14414 (9509, 20331) | 59169 (40279, 81195) |

Table S5: Estimated excess TB mortality in Africa for the years 2020 to 2023, presented both individually by year and as a total. The estimates include the median value along with the corresponding lower and upper bounds, calculated using an ensemble framework that models two distinct sub-epidemics.

## S3.2 Excess mortality rate

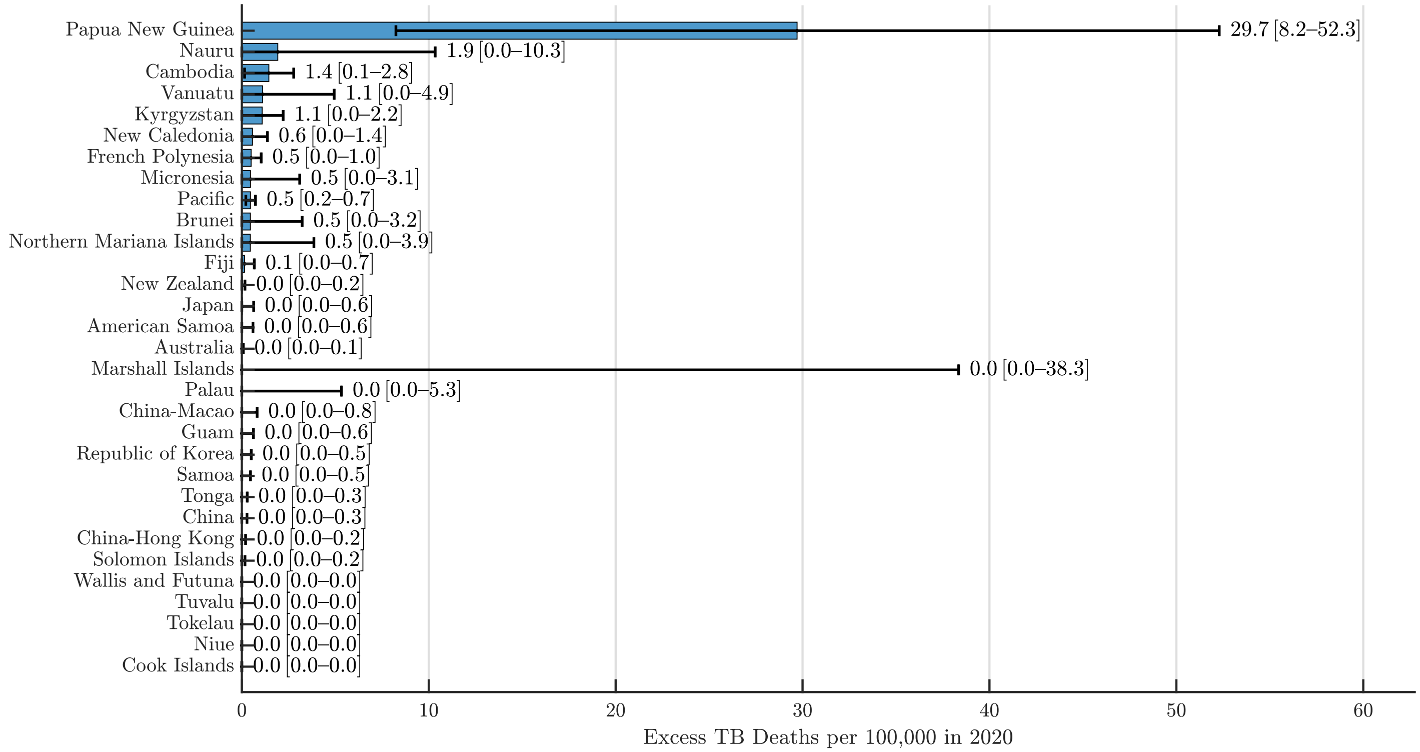


Figure S39: Excess TB mortality rate per 100,000 in Pacific in 2020, estimated using an ensemble framework with two sub-epidemics. The horizontal bar charts display the median excess mortality rate for each country, with horizontal lines extending from each bar representing the corresponding confidence intervals (lower and upper bounds). Exact mortality rate numbers are also provided for each country.

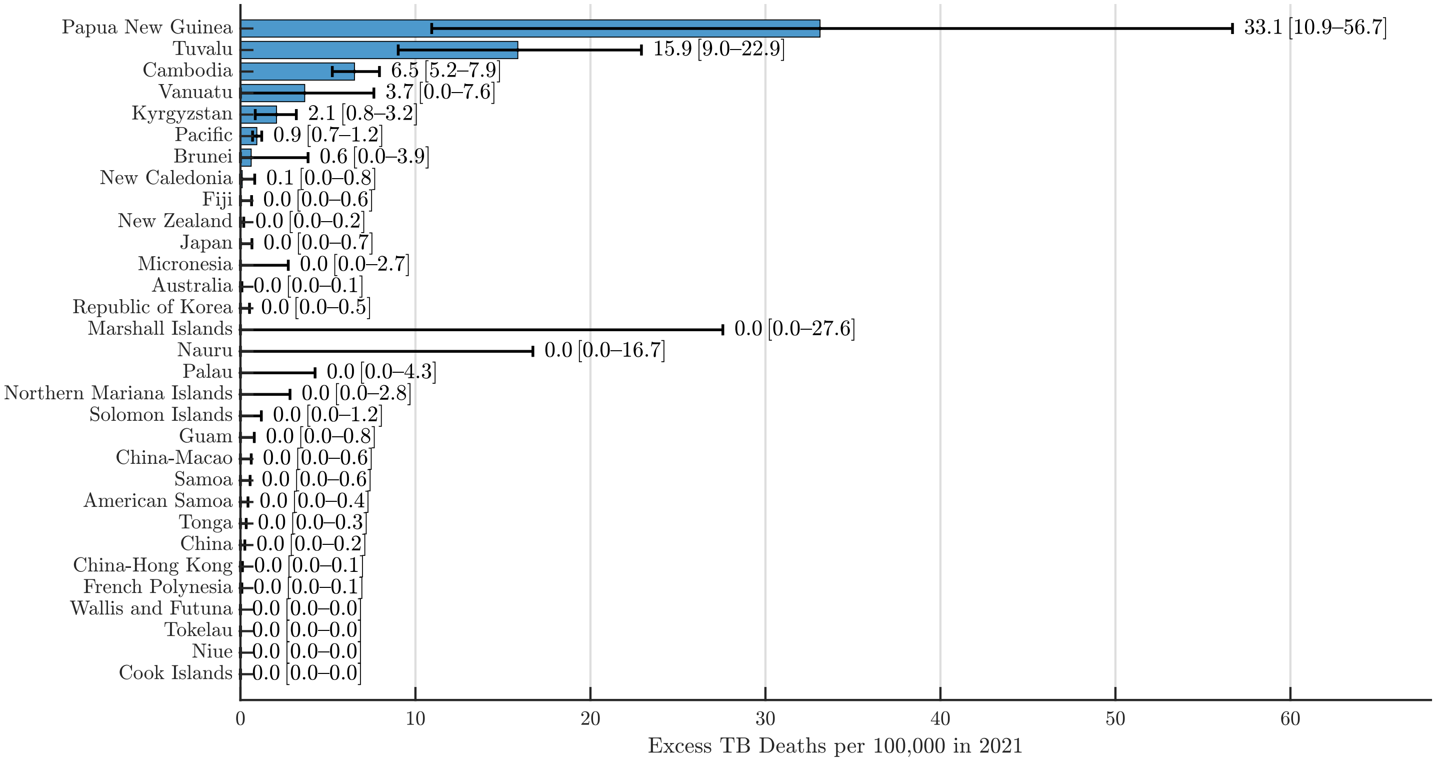


Figure S40: Excess TB mortality rate per 100,000 in Pacific in 2021, estimated using an ensemble framework with two sub-epidemics. The horizontal bar charts display the median excess mortality rate for each country, with horizontal lines extending from each bar representing the corresponding confidence intervals (lower and upper bounds). Exact mortality rate numbers are also provided for each country.

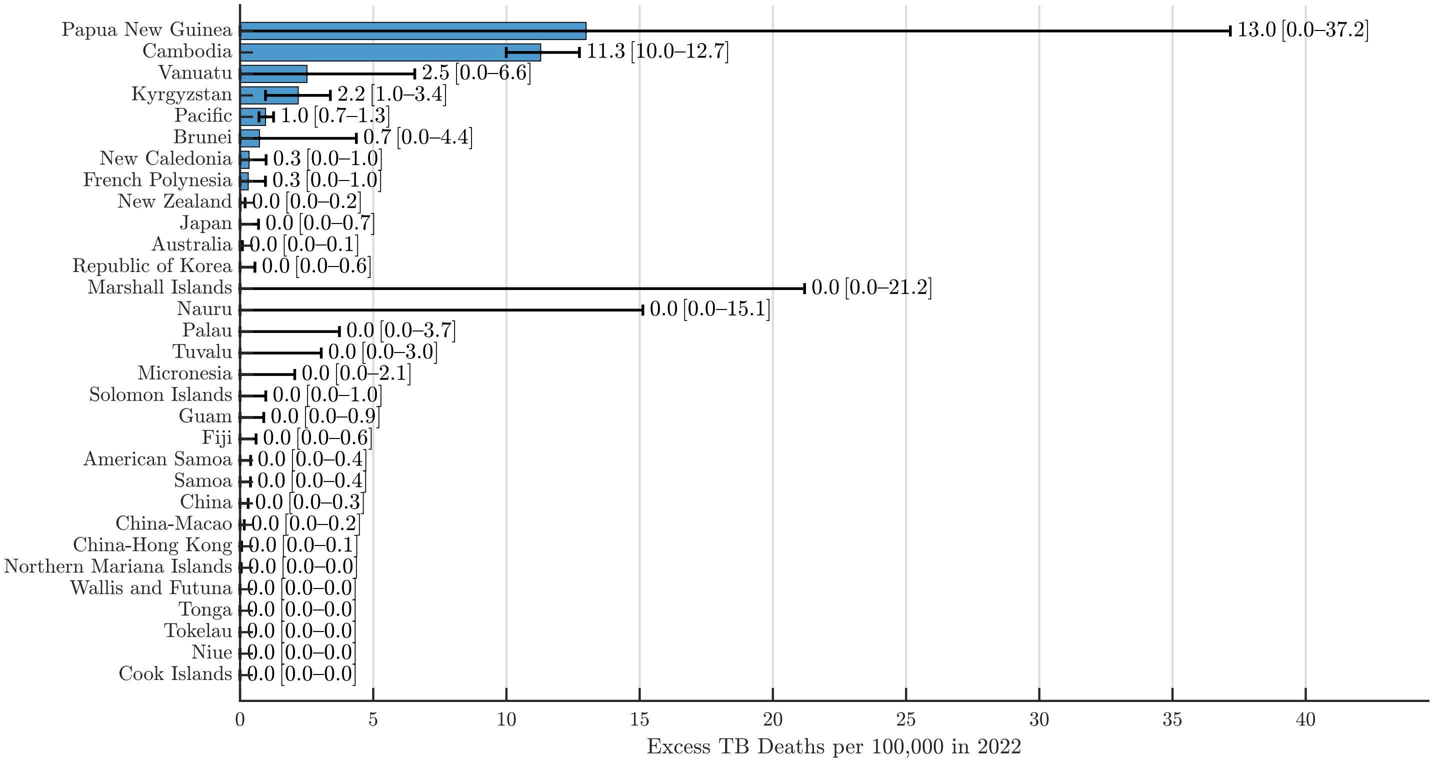


Figure S41: Excess TB mortality rate per 100,000 in Pacific in 2022, estimated using an ensemble framework with two sub-epidemics. The horizontal bar charts display the median excess mortality rate for each country, with horizontal lines extending from each bar representing the corresponding confidence intervals (lower and upper bounds). Exact mortality rate numbers are also provided for each country.

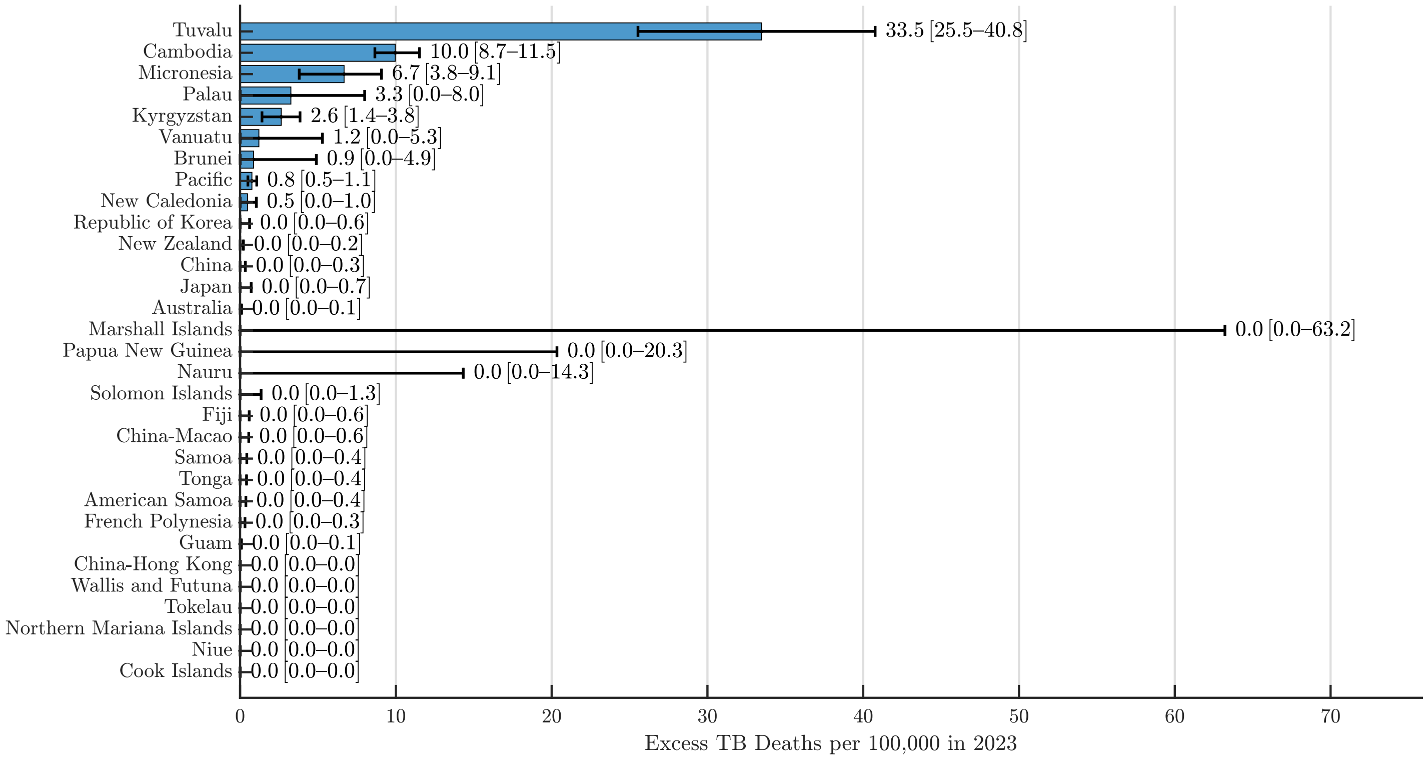


Figure S42: Excess TB mortality rate per 100,000 in Pacific in 2023, estimated using an ensemble framework with two sub-epidemics. The horizontal bar charts display the median excess mortality rate for each country, with horizontal lines extending from each bar representing the corresponding confidence intervals (lower and upper bounds). Exact mortality rate numbers are also provided for each country.

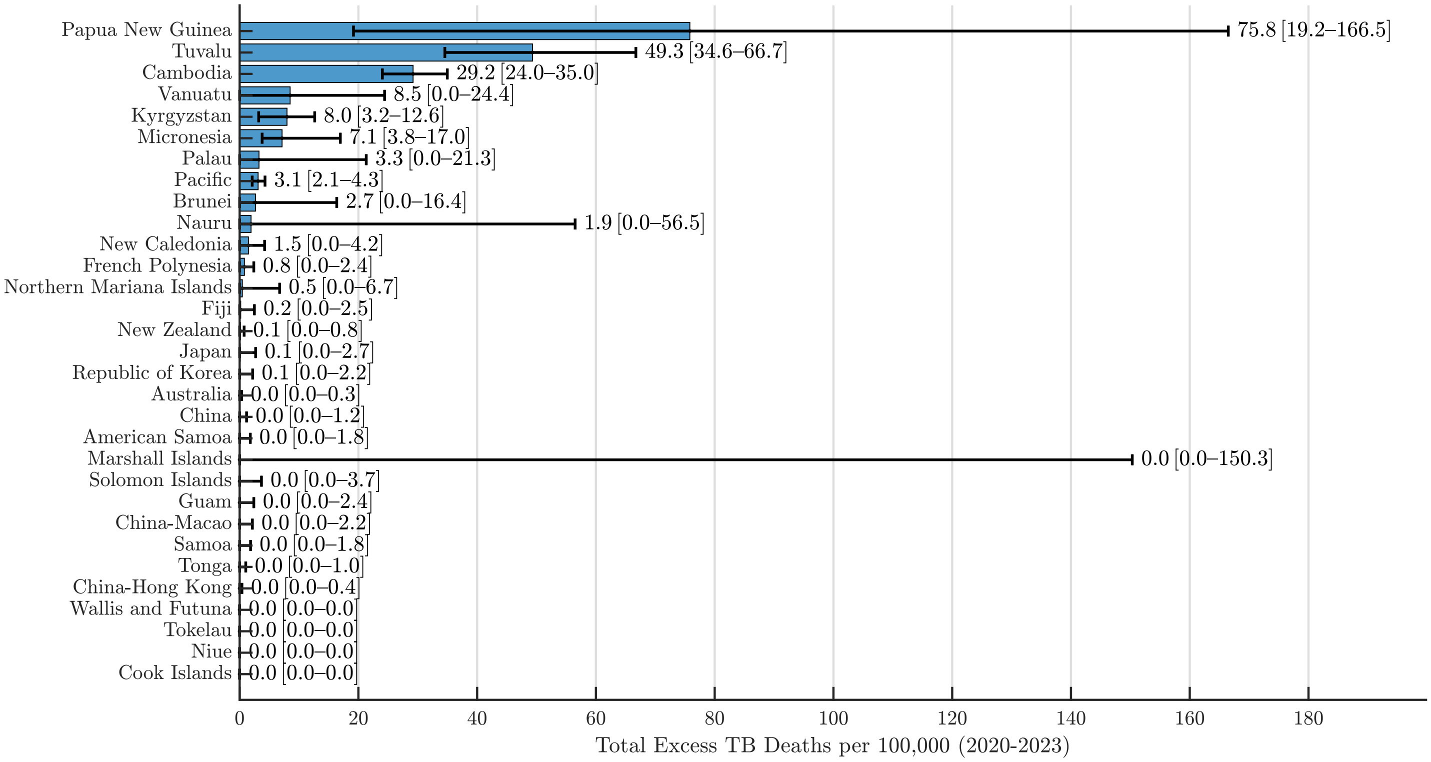


Figure S43: Total excess TB mortality rate per 100,000 in Pacific in 2020-2023, estimated using an ensemble framework with two sub-epidemics. The horizontal bar charts display the median excess mortality rate for each country, with horizontal lines extending from each bar representing the corresponding confidence intervals (lower and upper bounds). Exact mortality rate numbers are also provided for each country.

| | Excess TB mortality rate (LB,UB) | | | | |
|---|---|---|---|---|---|
| Country | **2020** | **2021** | **2022** | **2023** | **Total** |
| **American Samoa** | 0.0 (0.0, 0.6) | 0.0 (0.0, 0.4) | 0.0 (0.0, 0.4) | 0.0 (0.0, 0.4) | 0.0 (0.0, 1.8) |
| **Australia** | 0.0 (0.0, 0.1) | 0.0 (0.0, 0.1) | 0.0 (0.0, 0.1) | 0.0 (0.0, 0.1) | 0.0 (0.0, 0.3) |
| **Brunei** | 0.5 (0.0, 3.2) | 0.6 (0.0, 3.9) | 0.7 (0.0, 4.4) | 0.9 (0.0, 4.9) | 2.7 (0.0, 16.4) |
| **Cambodia** | 1.4 (0.1, 2.8) | 6.5 (5.2, 7.9) | 11.3 (10.0, 12.7) | 10.0 (8.7, 11.5) | 29.2 (24.0, 35.0) |
| **China** | 0.0 (0.0, 0.3) | 0.0 (0.0, 0.2) | 0.0 (0.0, 0.3) | 0.0 (0.0, 0.3) | 0.0 (0.0, 1.2) |
| **China-Hong Kong** | 0.0 (0.0, 0.2) | 0.0 (0.0, 0.1) | 0.0 (0.0, 0.1) | 0.0 (0.0, 0.0) | 0.0 (0.0, 0.4) |
| **China-Macao** | 0.0 (0.0, 0.8) | 0.0 (0.0, 0.6) | 0.0 (0.0, 0.2) | 0.0 (0.0, 0.6) | 0.0 (0.0, 2.2) |
| **Fiji** | 0.1 (0.0, 0.7) | 0.0 (0.0, 0.6) | 0.0 (0.0, 0.6) | 0.0 (0.0, 0.6) | 0.2 (0.0, 2.5) |
| **French Polynesia** | 0.5 (0.0, 1.0) | 0.0 (0.0, 0.1) | 0.3 (0.0, 1.0) | 0.0 (0.0, 0.3) | 0.8 (0.0, 2.4) |
| **Guam** | 0.0 (0.0, 0.6) | 0.0 (0.0, 0.8) | 0.0 (0.0, 0.9) | 0.0 (0.0, 0.1) | 0.0 (0.0, 2.4) |
| **Japan** | 0.0 (0.0, 0.6) | 0.0 (0.0, 0.7) | 0.0 (0.0, 0.7) | 0.0 (0.0, 0.7) | 0.1 (0.0, 2.7) |
| **Kyrgyzstan** | 1.1 (0.0, 2.2) | 2.1 (0.8, 3.2) | 2.2 (1.0, 3.4) | 2.6 (1.4, 3.8) | 8.0 (3.2, 12.6) |
| **Marshall Islands** | 0.0 (0.0, 38.3) | 0.0 (0.0, 27.6) | 0.0 (0.0, 21.2) | 0.0 (0.0, 63.2) | 0.0 (0.0, 150.3) |
| **Micronesia** | 0.5 (0.0, 3.1) | 0.0 (0.0, 2.7) | 0.0 (0.0, 2.1) | 6.7 (3.8, 9.1) | 7.1 (3.8, 17.0) |
| **Nauru** | 1.9 (0.0, 10.3) | 0.0 (0.0, 16.7) | 0.0 (0.0, 15.1) | 0.0 (0.0, 14.3) | 1.9 (0.0, 56.5) |
| **New Caledonia** | 0.6 (0.0, 1.4) | 0.1 (0.0, 0.8) | 0.3 (0.0, 1.0) | 0.5 (0.0, 1.0) | 1.5 (0.0, 4.2) |
| **New Zealand** | 0.0 (0.0, 0.2) | 0.0 (0.0, 0.2) | 0.0 (0.0, 0.2) | 0.0 (0.0, 0.2) | 0.1 (0.0, 0.8) |
| **Northern Mariana Islands** | 0.5 (0.0, 3.9) | 0.0 (0.0, 2.8) | 0.0 (0.0, 0.0) | 0.0 (0.0, 0.0) | 0.5 (0.0, 6.7) |
| **Palau** | 0.0 (0.0, 5.3) | 0.0 (0.0, 4.3) | 0.0 (0.0, 3.7) | 3.3 (0.0, 8.0) | 3.3 (0.0, 21.3) |
| **Papua New Guinea** | 29.7 (8.2, 52.3) | 33.1 (10.9, 56.7) | 13.0 (0.0, 37.2) | 0.0 (0.0, 20.3) | 75.8 (19.2, 166.5) |
| **Republic of Korea** | 0.0 (0.0, 0.5) | 0.0 (0.0, 0.5) | 0.0 (0.0, 0.6) | 0.0 (0.0, 0.6) | 0.1 (0.0, 2.2) |
| **Samoa** | 0.0 (0.0, 0.5) | 0.0 (0.0, 0.6) | 0.0 (0.0, 0.4) | 0.0 (0.0, 0.4) | 0.0 (0.0, 1.8) |
| **Solomon Islands** | 0.0 (0.0, 0.2) | 0.0 (0.0, 1.2) | 0.0 (0.0, 1.0) | 0.0 (0.0, 1.3) | 0.0 (0.0, 3.7) |
| **Tonga** | 0.0 (0.0, 0.3) | 0.0 (0.0, 0.3) | 0.0 (0.0, 0.0) | 0.0 (0.0, 0.4) | 0.0 (0.0, 1.0) |
| **Tuvalu** | 0.0 (0.0, 0.0) | 15.9 (9.0, 22.9) | 0.0 (0.0, 3.0) | 33.5 (25.5, 40.8) | 49.3 (34.6, 66.7) |
| **Vanuatu** | 1.1 (0.0, 4.9) | 3.7 (0.0, 7.6) | 2.5 (0.0, 6.6) | 1.2 (0.0, 5.3) | 8.5 (0.0, 24.4) |
| **Wallis and Futuna** | 0.0 (0.0, 0.0) | 0.0 (0.0, 0.0) | 0.0 (0.0, 0.0) | 0.0 (0.0, 0.0) | 0.0 (0.0, 0.0) |
| **Pacific** | 0.5 (0.2, 0.7) | 0.9 (0.7, 1.2) | 1.0 (0.7, 1.3) | 0.8 (0.5, 1.1) | 3.1 (2.1, 4.3) |

Table S6: Estimated excess TB mortality rate in Pacific for the years 2020 to 2023, presented both individually by year and as a total. The estimates include the median value along with the corresponding lower and upper bounds, calculated using an ensemble framework that models two distinct sub-epidemics.

### S3.3 SMR

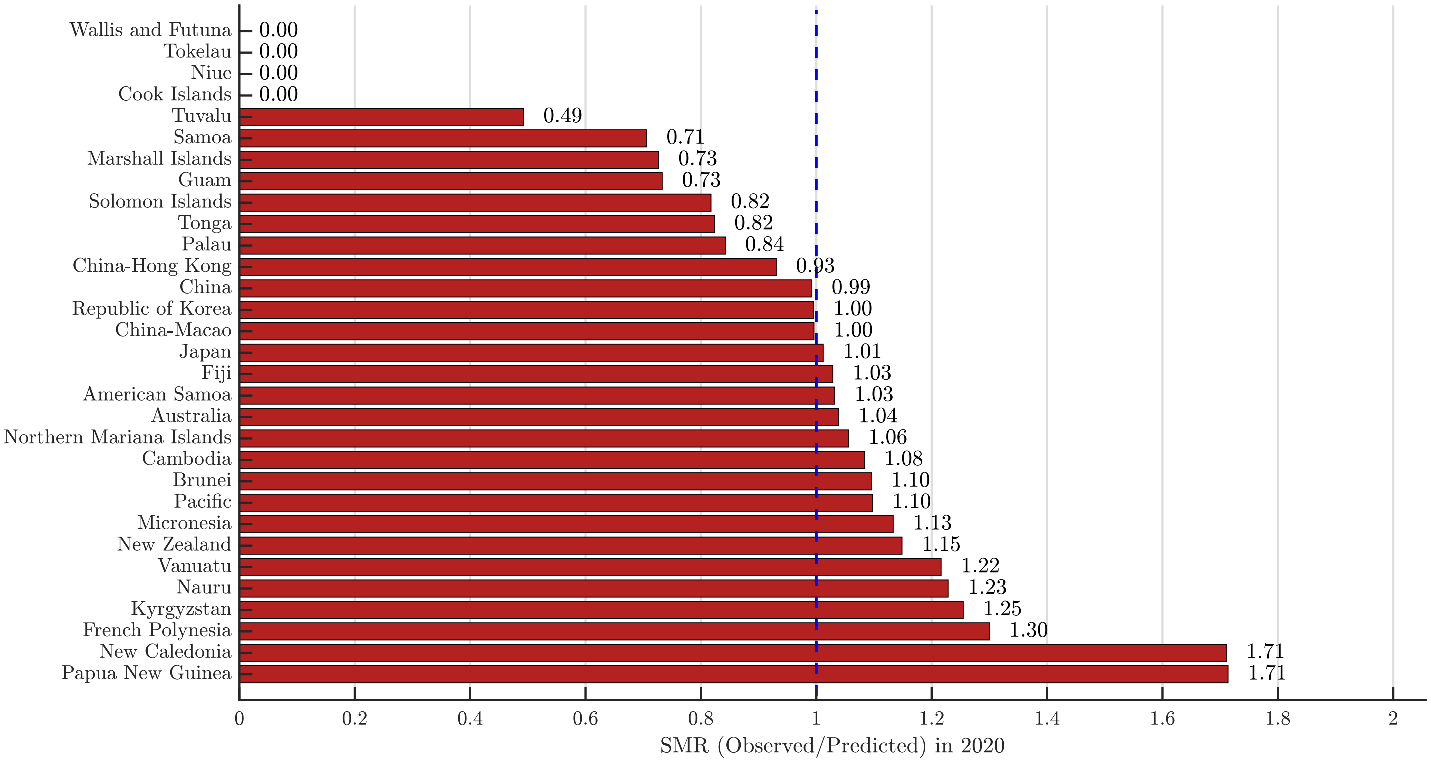


Figure S44: SMR in Pacific in 2020, estimated using an ensemble framework with two sub-epidemics. SMR is displayed horizontally in bar charts, with the exact number displayed in front of the bars.

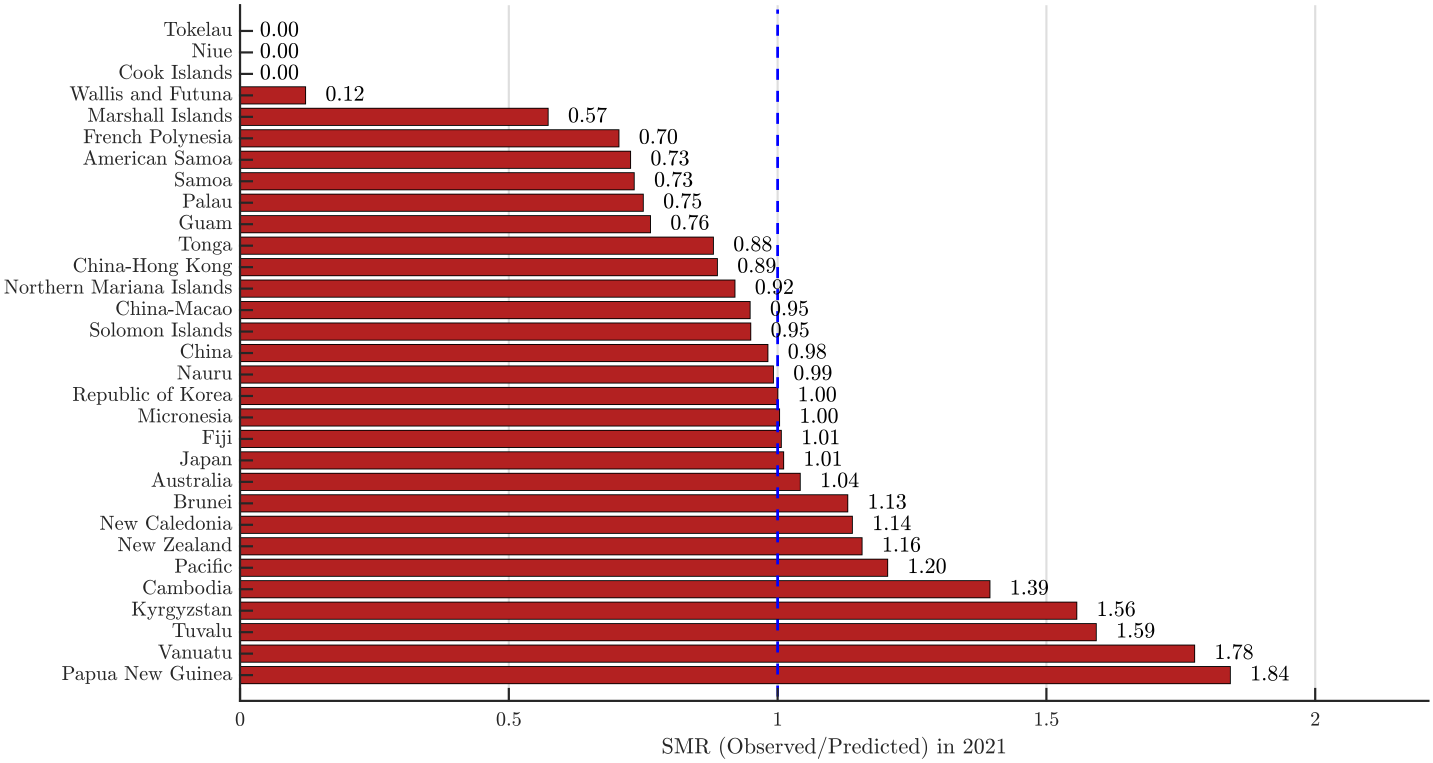


Figure S45: SMR in Pacific in 2021, estimated using an ensemble framework with two sub-epidemics. SMR is displayed horizontally in bar charts, with the exact number displayed in front of the bars.

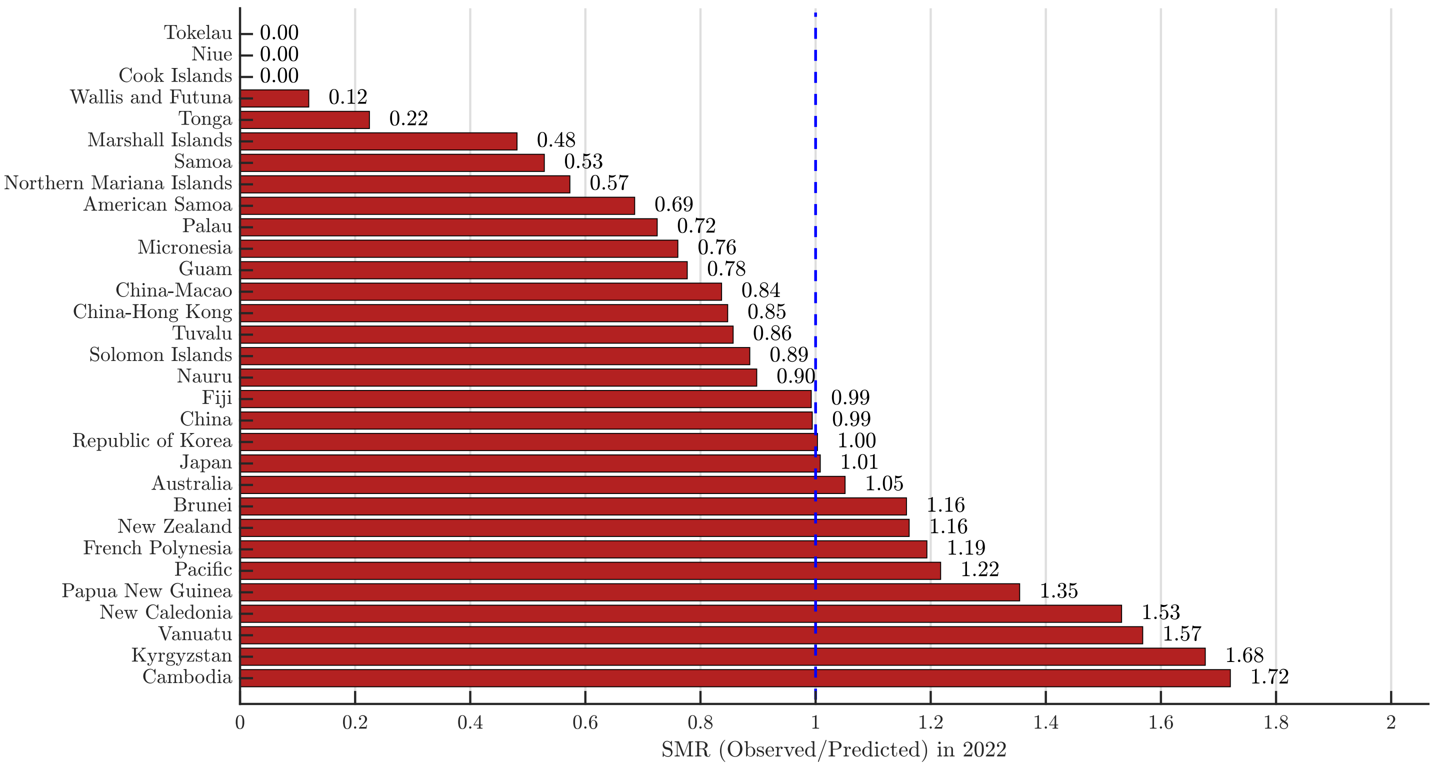


Figure S46: SMR in Pacific in 2022, estimated using an ensemble framework with two sub-epidemics. SMR is displayed horizontally in bar charts, with the exact number displayed in front of the bars.

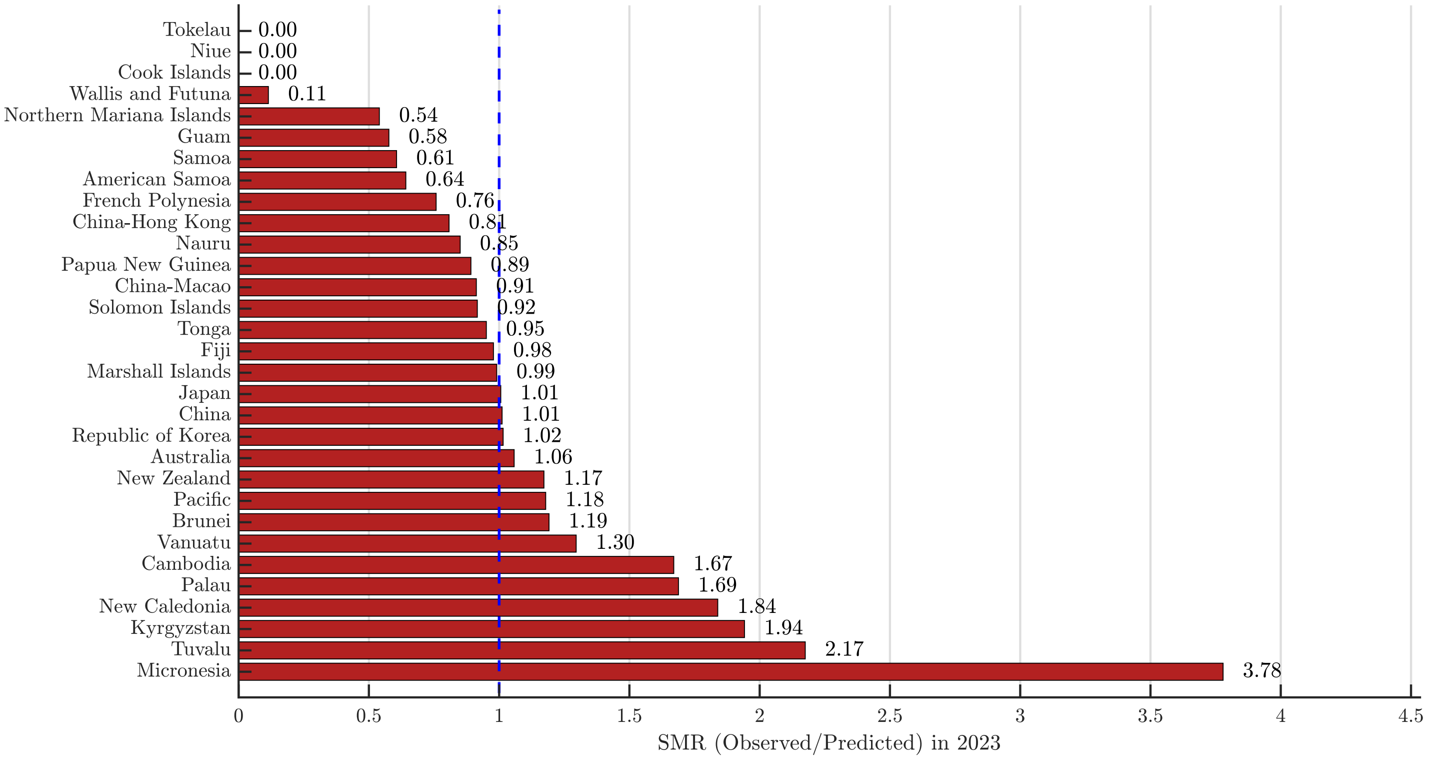


Figure S47: SMR in Pacific in 2023, estimated using an ensemble framework with two sub-epidemics. SMR is displayed horizontally in bar charts, with the exact number displayed in front of the bars.

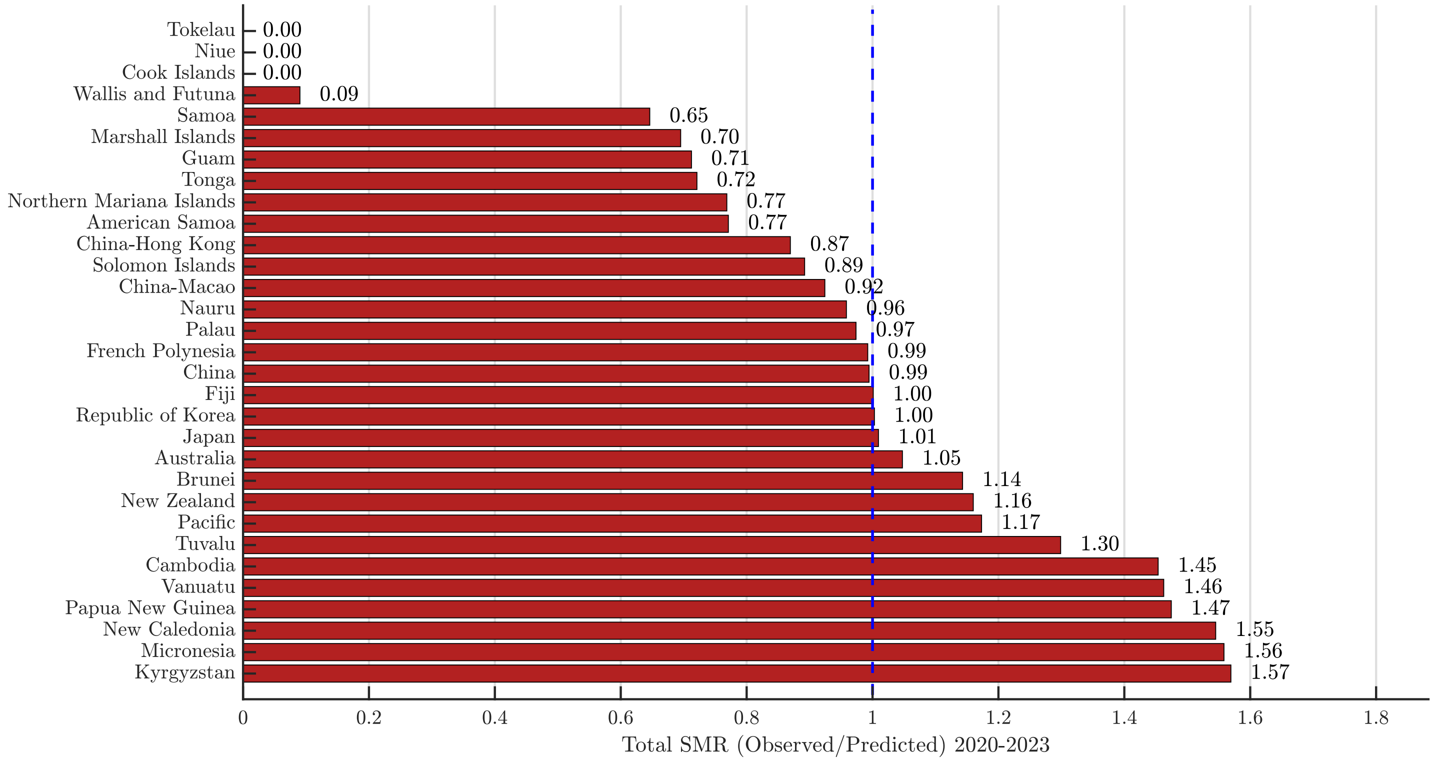


Figure S48: SMR in Pacific during 2020-2023, estimated using an ensemble framework with two sub-epidemics. SMR is displayed horizontally in bar charts, with the exact number displayed in front of the bars.

# S4 Africa

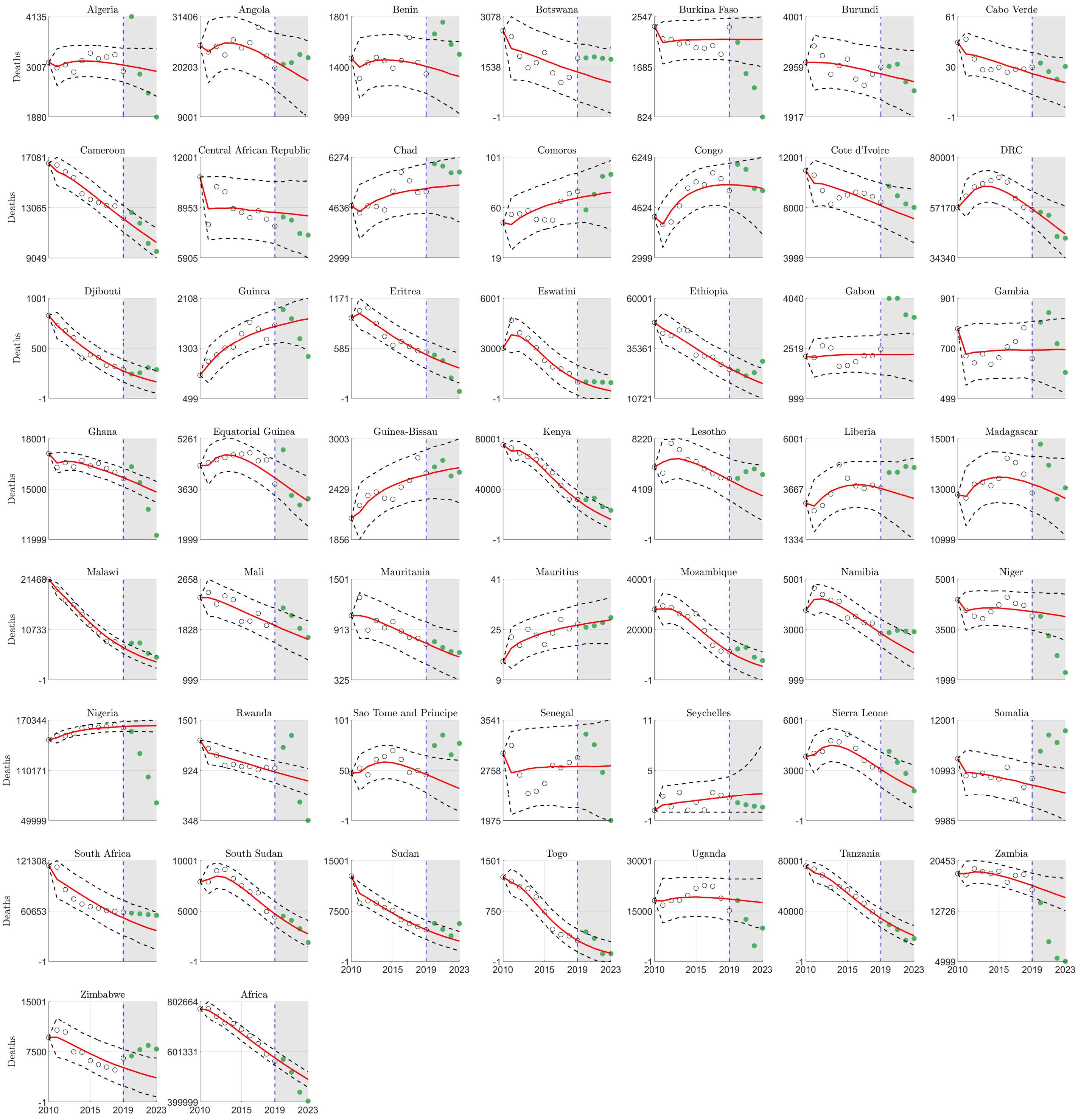

Figure S49: Forecasting panel for the number of deaths in Africa, based on a 10-year calibration period (2010–2019) and a four-year forecasting period (2020–2023), generated using the Ranked 1 method. The red curve denotes the median forecast, while the black dashed lines mark the 95% PI. Reported data points are shown as circles: black circles represent the calibration period, and green-filled circles represent actual data points during the forecast period.

## S4.1 Excess mortality

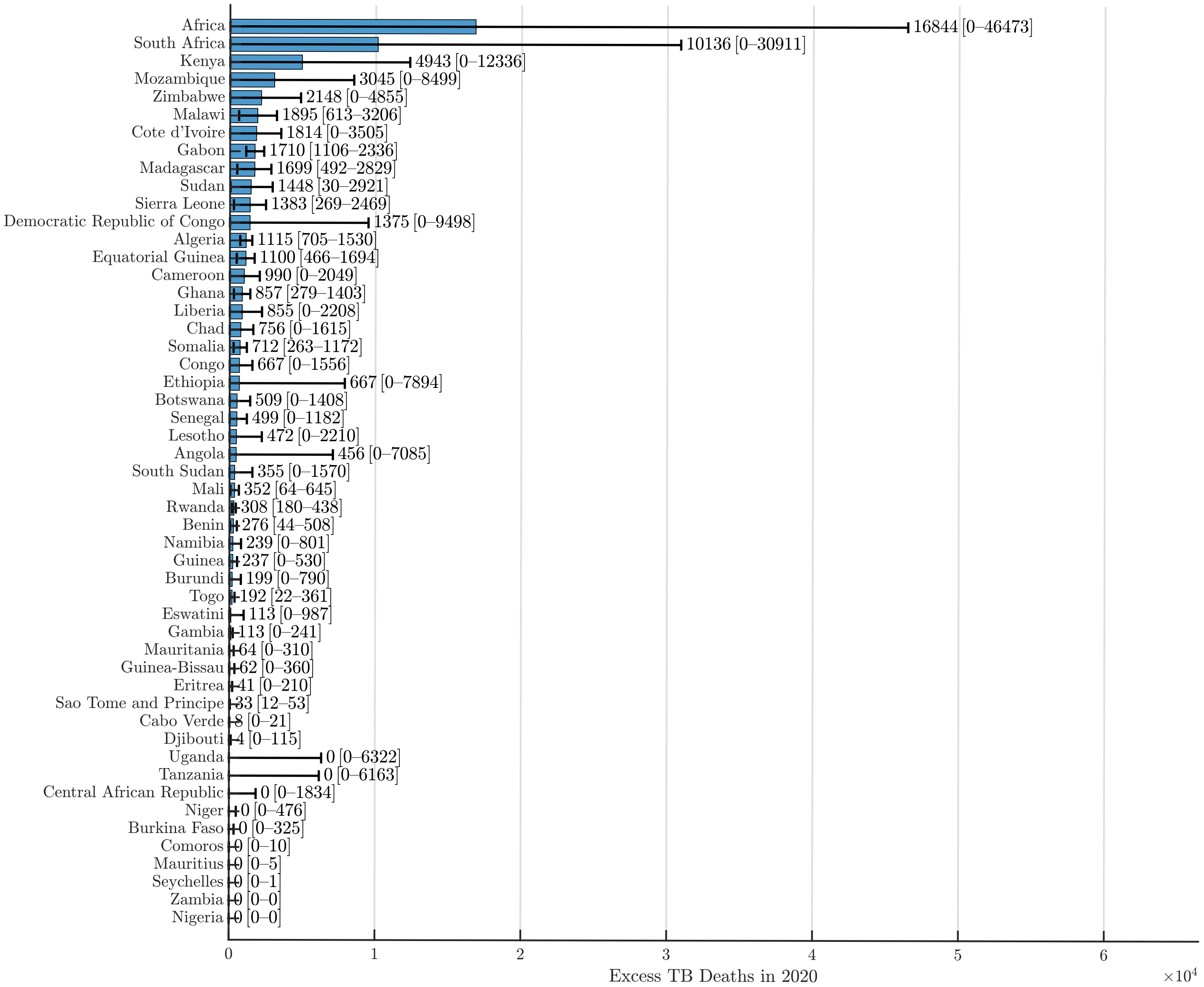


Figure S50: Excess TB mortality in Africa in 2020, estimated using an ensemble framework with two sub-epidemics. The horizontal bar charts display the median excess mortality for each country, with horizontal lines extending from each bar representing the corresponding confidence intervals (lower and upper bounds). Exact mortality numbers are also provided for each country.

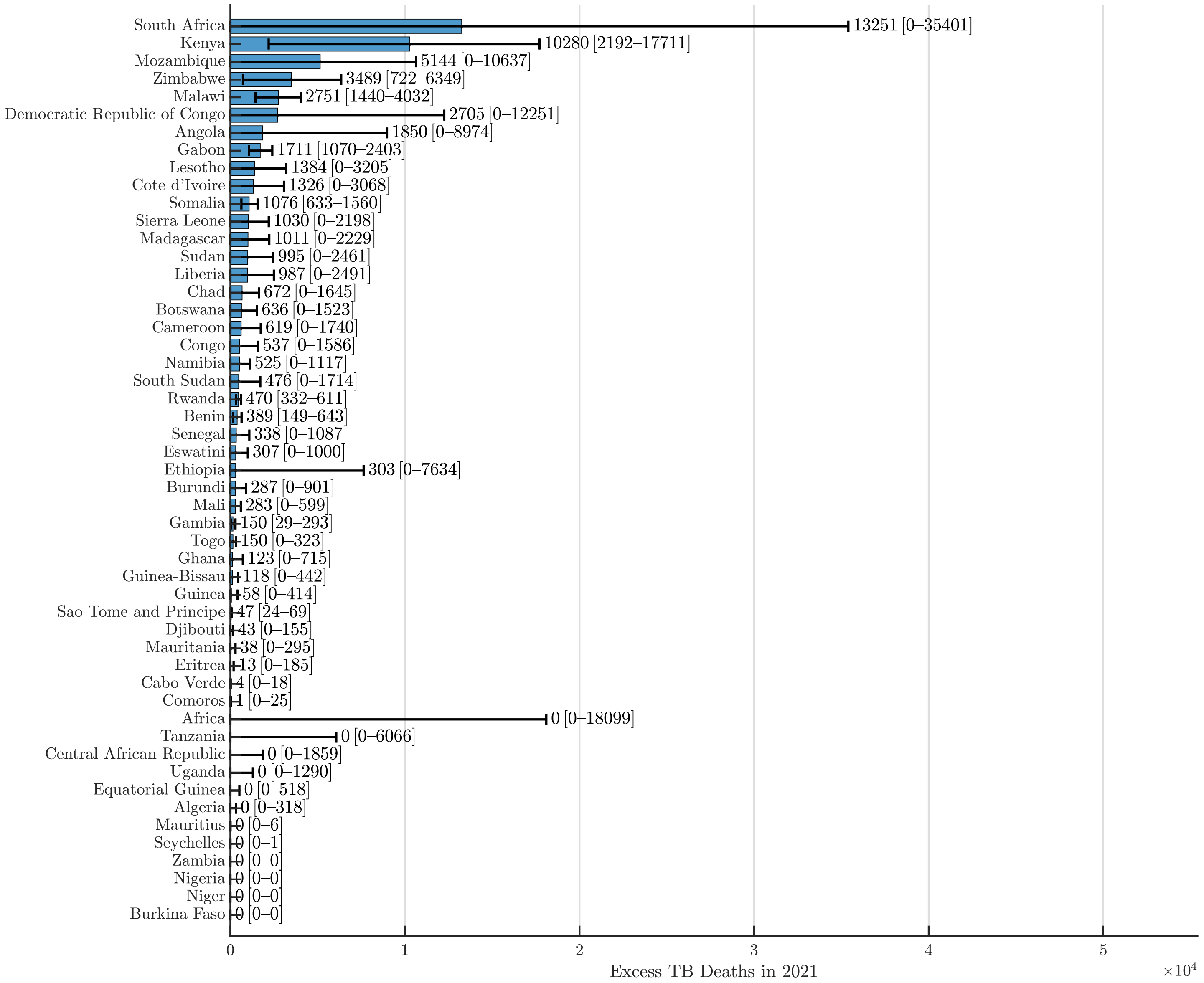


Figure S51: Excess TB mortality in Africa in 2021, estimated using an ensemble framework with two sub-epidemics. The horizontal bar charts display the median excess mortality for each country, with horizontal lines extending from each bar representing the corresponding confidence intervals (lower and upper bounds). Exact mortality numbers are also provided for each country.

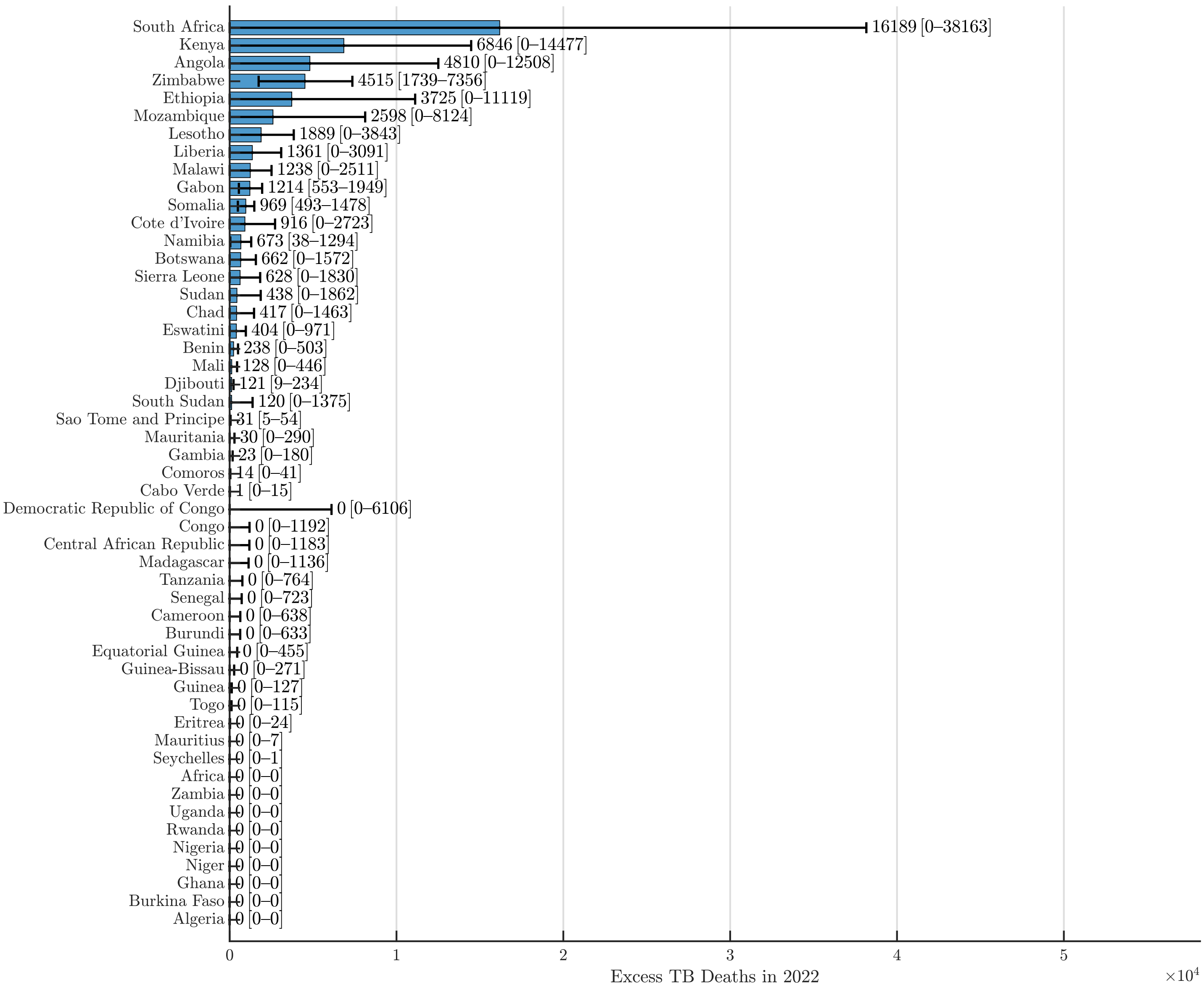


Figure S52: Excess TB mortality in Africa in 2022, estimated using an ensemble framework with two sub-epidemics. The horizontal bar charts display the median excess mortality for each country, with horizontal lines extending from each bar representing the corresponding confidence intervals (lower and upper bounds). Exact mortality numbers are also provided for each country.

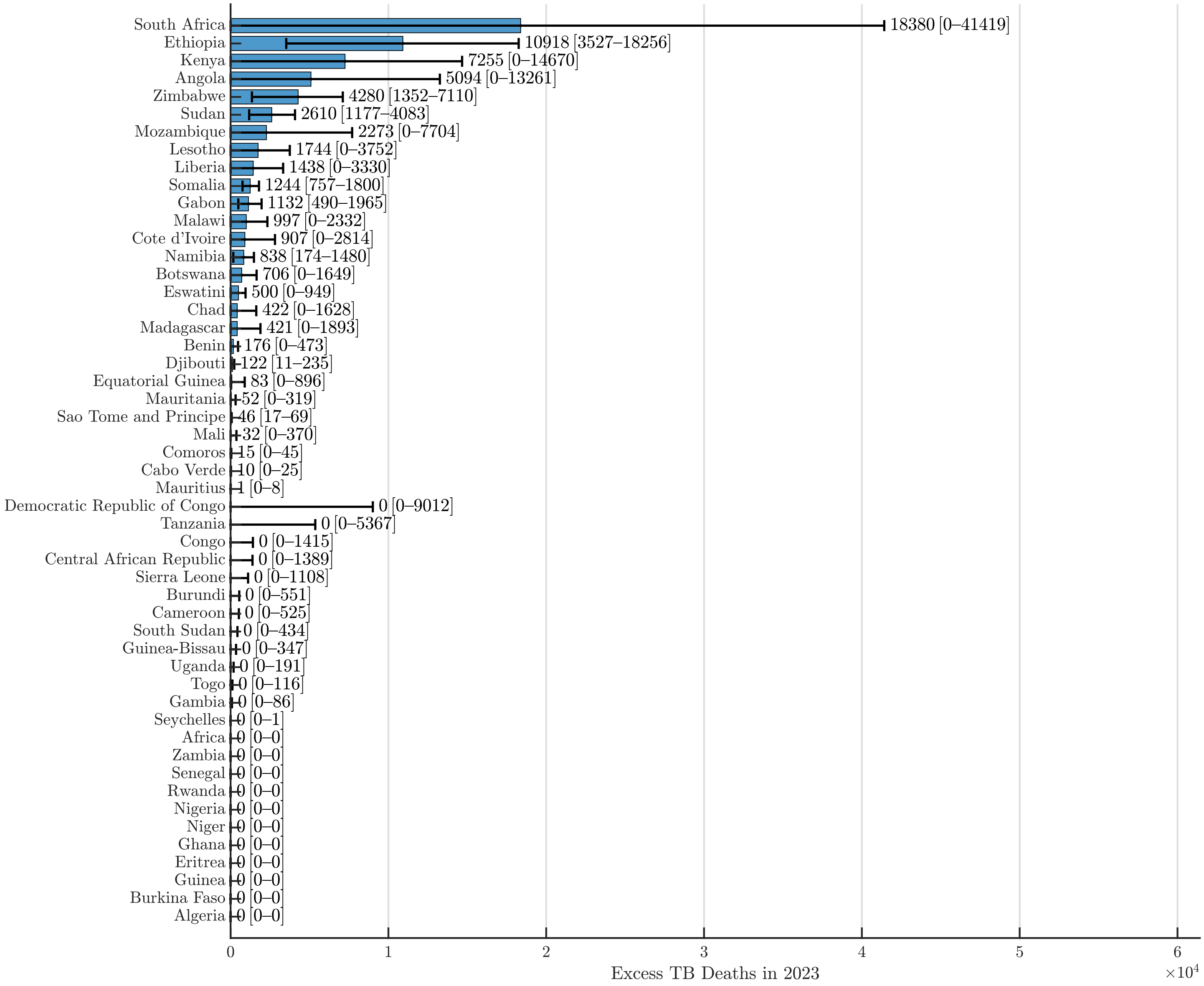


Figure S53: Excess TB mortality in Africa in 2023, estimated using an ensemble framework with two sub-epidemics. The horizontal bar charts display the median excess mortality for each country, with horizontal lines extending from each bar representing the corresponding confidence intervals (lower and upper bounds). Exact mortality numbers are also provided for each country.

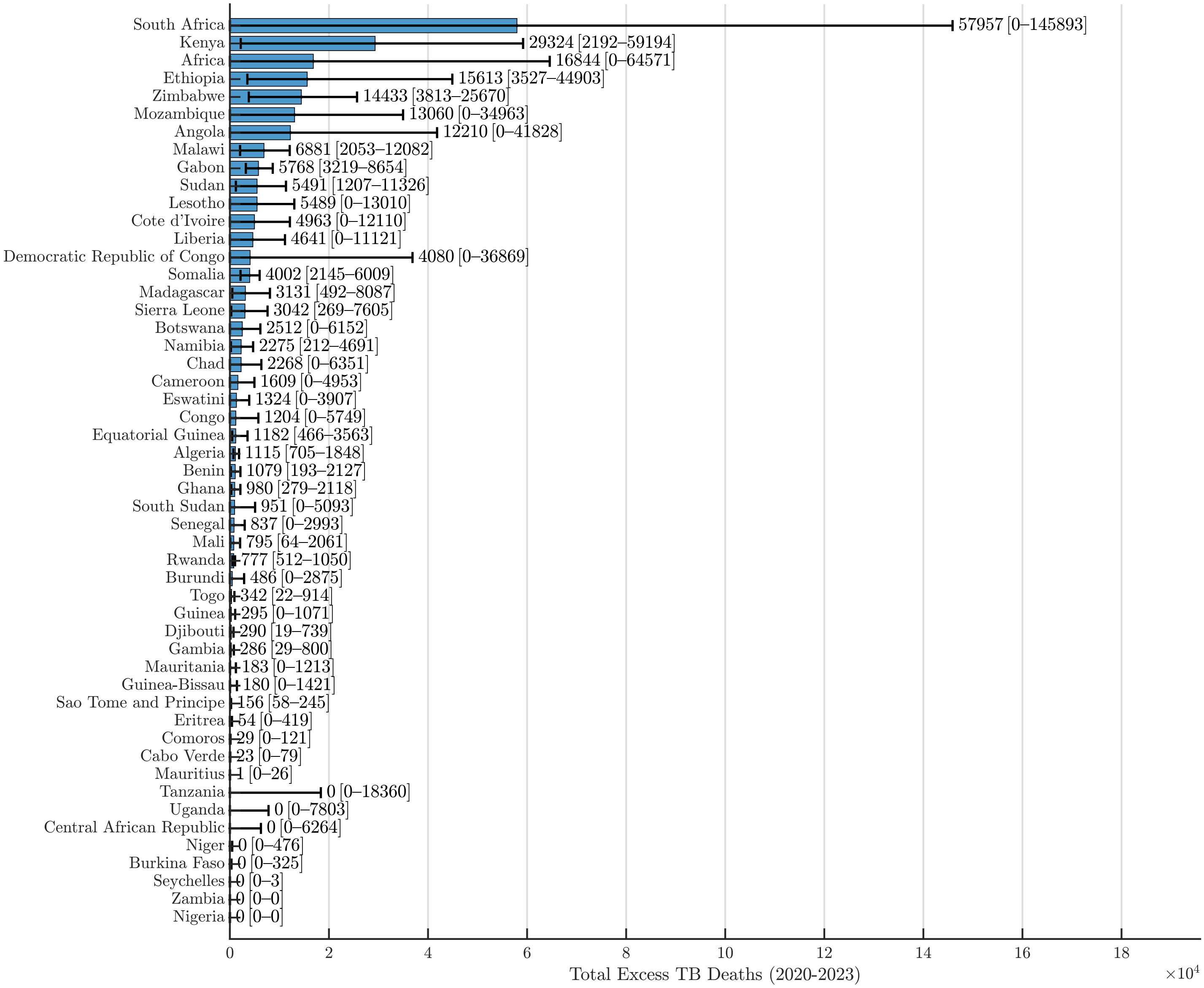


Figure S54: Total excess TB mortality in Africa in 2020-2023, estimated using an ensemble framework with two sub-epidemics. The horizontal bar charts display the median excess mortality for each country, with horizontal lines extending from each bar representing the corresponding confidence intervals (lower and upper bounds). Exact mortality numbers are also provided for each country.

| | Excess TB mortality (LB,UB) | | | | |
|---|---|---|---|---|---|
| Country | **2020** | **2021** | **2022** | **2023** | **Total** |
| **Algeria** | 1115 (705, 1530) | 0 (0, 318) | 0 (0, 0) | 0 (0, 0) | 1115 (705, 1848) |
| **Angola** | 456 (0, 7085) | 1850 (0, 8974) | 4810 (0, 12508) | 5094 (0, 13261) | 12210 (0, 41828) |
| **Benin** | 276 (44, 508) | 389 (149, 643) | 238 (0, 503) | 176 (0, 473) | 1079 (193, 2127) |
| **Botswana** | 509 (0, 1408) | 636 (0, 1523) | 662 (0, 1572) | 706 (0, 1649) | 2512 (0, 6152) |
| **Burkina Faso** | 0 (0, 325) | 0 (0, 0) | 0 (0, 0) | 0 (0, 0) | 0 (0, 325) |
| **Burundi** | 199 (0, 790) | 287 (0, 901) | 0 (0, 633) | 0 (0, 551) | 486 (0, 2875) |
| **Cabo Verde** | 8 (0, 21) | 4 (0, 18) | 1 (0, 15) | 10 (0, 25) | 23 (0, 79) |
| **Cameroon** | 990 (0, 2049) | 619 (0, 1740) | 0 (0, 638) | 0 (0, 525) | 1609 (0, 4953) |
| **Central African Republic** | 0 (0, 1834) | 0 (0, 1859) | 0 (0, 1183) | 0 (0, 1389) | 0 (0, 6264) |
| **Chad** | 756 (0, 1615) | 672 (0, 1645) | 417 (0, 1463) | 422 (0, 1628) | 2268 (0, 6351) |
| **Comoros** | 0 (0, 10) | 1 (0, 25) | 14 (0, 41) | 15 (0, 45) | 29 (0, 121) |
| **Congo** | 667 (0, 1556) | 537 (0, 1586) | 0 (0, 1192) | 0 (0, 1415) | 1204 (0, 5749) |
| **Cote d'Ivoire** | 1814 (0, 3505) | 1326 (0, 3068) | 916 (0, 2723) | 907 (0, 2814) | 4963 (0, 12110) |
| **Democratic Republic of Congo** | 1375 (0, 9498) | 2705 (0, 12251) | 0 (0, 6106) | 0 (0, 9012) | 4080 (0, 36869) |
| **Djibouti** | 4 (0, 115) | 43 (0, 155) | 121 (9, 234) | 122 (11, 235) | 290 (19, 739) |
| **Guinea** | 237 (0, 530) | 58 (0, 414) | 0 (0, 127) | 0 (0, 0) | 295 (0, 1071) |
| **Eritrea** | 41 (0, 210) | 13 (0, 185) | 0 (0, 24) | 0 (0, 0) | 54 (0, 419) |
| **Eswatini** | 113 (0, 987) | 307 (0, 1000) | 404 (0, 971) | 500 (0, 949) | 1324 (0, 3907) |
| **Ethiopia** | 667 (0, 7894) | 303 (0, 7634) | 3725 (0, 11119) | 10918 (3527, 18256) | 15613 (3527, 44903) |
| **Gabon** | 1710 (1106, 2336) | 1711 (1070, 2403) | 1214 (553, 1949) | 1132 (490, 1965) | 5768 (3219, 8654) |
| **Gambia** | 113 (0, 241) | 150 (29, 293) | 23 (0, 180) | 0 (0, 86) | 286 (29, 800) |
| **Ghana** | 857 (279, 1403) | 123 (0, 715) | 0 (0, 0) | 0 (0, 0) | 980 (279, 2118) |
| **Equatorial Guinea** | 1100 (466, 1694) | 0 (0, 518) | 0 (0, 455) | 83 (0, 896) | 1182 (466, 3563) |
| **Guinea-Bissau** | 62 (0, 360) | 118 (0, 442) | 0 (0, 271) | 0 (0, 347) | 180 (0, 1421) |
| **Kenya** | 4943 (0, 12336) | 10280 (2192, 17711) | 6846 (0, 14477) | 7255 (0, 14670) | 29324 (2192, 59194) |
| **Lesotho** | 472 (0, 2210) | 1384 (0, 3205) | 1889 (0, 3843) | 1744 (0, 3752) | 5489 (0, 13010) |
| **Liberia** | 855 (0, 2208) | 987 (0, 2491) | 1361 (0, 3091) | 1438 (0, 3330) | 4641 (0, 11121) |
| **Madagascar** | 1699 (492, 2829) | 1011 (0, 2229) | 0 (0, 1136) | 421 (0, 1893) | 3131 (492, 8087) |
| **Malawi** | 1895 (613, 3206) | 2751 (1440, 4032) | 1238 (0, 2511) | 997 (0, 2332) | 6881 (2053, 12082) |
| **Mali** | 352 (64, 645) | 283 (0, 599) | 128 (0, 446) | 32 (0, 370) | 795 (64, 2061) |
| **Mauritania** | 64 (0, 310) | 38 (0, 295) | 30 (0, 290) | 52 (0, 319) | 183 (0, 1213) |
| **Mauritius** | 0 (0, 5) | 0 (0, 6) | 0 (0, 7) | 1 (0, 8) | 1 (0, 26) |
| **Mozambique** | 3045 (0, 8499) | 5144 (0, 10637) | 2598 (0, 8124) | 2273 (0, 7704) | 13060 (0, 34963) |
| **Namibia** | 239 (0, 801) | 525 (0, 1117) | 673 (38, 1294) | 838 (174, 1480) | 2275 (212, 4691) |
| **Niger** | 0 (0, 476) | 0 (0, 0) | 0 (0, 0) | 0 (0, 0) | 0 (0, 476) |
| **Nigeria** | 0 (0, 0) | 0 (0, 0) | 0 (0, 0) | 0 (0, 0) | 0 (0, 0) |
| **Rwanda** | 308 (180, 438) | 470 (332, 611) | 0 (0, 0) | 0 (0, 0) | 777 (512, 1050) |
| **Sao Tome and Principe** | 33 (12, 53) | 47 (24, 69) | 31 (5, 54) | 46 (17, 69) | 156 (58, 245) |
| **Senegal** | 499 (0, 1182) | 338 (0, 1087) | 0 (0, 723) | 0 (0, 0) | 837 (0, 2993) |
| **Seychelles** | 0 (0, 1) | 0 (0, 1) | 0 (0, 1) | 0 (0, 1) | 0 (0, 3) |
| **Sierra Leone** | 1383 (269, 2469) | 1030 (0, 2198) | 628 (0, 1830) | 0 (0, 1108) | 3042 (269, 7605) |
| **Somalia** | 712 (263, 1172) | 1076 (633, 1560) | 969 (493, 1478) | 1244 (757, 1800) | 4002 (2145, 6009) |
| **South Africa** | 10136 (0, 30911) | 13251 (0, 35401) | 16189 (0, 38163) | 18380 (0, 41419) | 57957 (0, 145893) |
| **South Sudan** | 355 (0, 1570) | 476 (0, 1714) | 120 (0, 1375) | 0 (0, 434) | 951 (0, 5093) |
| **Sudan** | 1448 (30, 2921) | 995 (0, 2461) | 438 (0, 1862) | 2610 (1177, 4083) | 5491 (1207, 11326) |
| **Togo** | 192 (22, 361) | 150 (0, 323) | 0 (0, 115) | 0 (0, 116) | 342 (22, 914) |
| **Uganda** | 0 (0, 6322) | 0 (0, 1290) | 0 (0, 0) | 0 (0, 191) | 0 (0, 7803) |
| **Tanzania** | 0 (0, 6163) | 0 (0, 6066) | 0 (0, 764) | 0 (0, 5367) | 0 (0, 18360) |
| **Zambia** | 0 (0, 0) | 0 (0, 0) | 0 (0, 0) | 0 (0, 0) | 0 (0, 0) |
| **Zimbabwe** | 2148 (0, 4855) | 3489 (722, 6349) | 4515 (1739, 7356) | 4280 (1352, 7110) | 14433 (3813, 25670) |
| **Africa** | 16844 (0, 46473) | 0 (0, 18099) | 0 (0, 0) | 0 (0, 0) | 16844 (0, 64571) |

Table S7: Estimated excess TB mortality in Africa for the years 2020 to 2023, presented both individually by year and as a total. The estimates include the median value along with the corresponding lower and upper bounds, calculated using an ensemble framework that models two distinct sub-epidemics.

## S4.2 Excess mortality rate

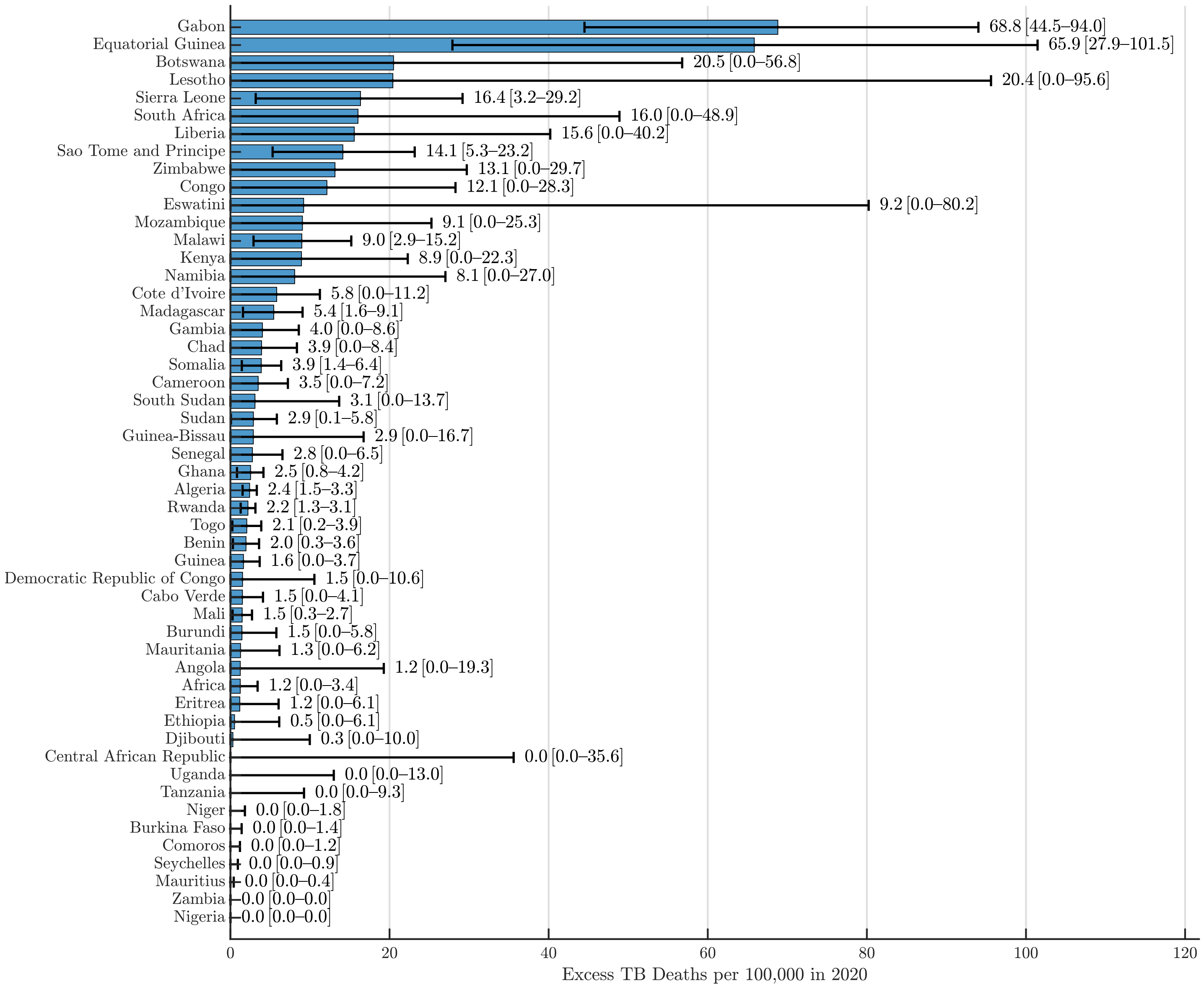


Figure S55: Excess TB mortality rate per 100,000 in Africa in 2020, estimated using an ensemble framework with two sub-epidemics. The horizontal bar charts display the median excess mortality rate for each country, with horizontal lines extending from each bar representing the corresponding confidence intervals (lower and upper bounds). Exact mortality rate numbers are also provided for each country.

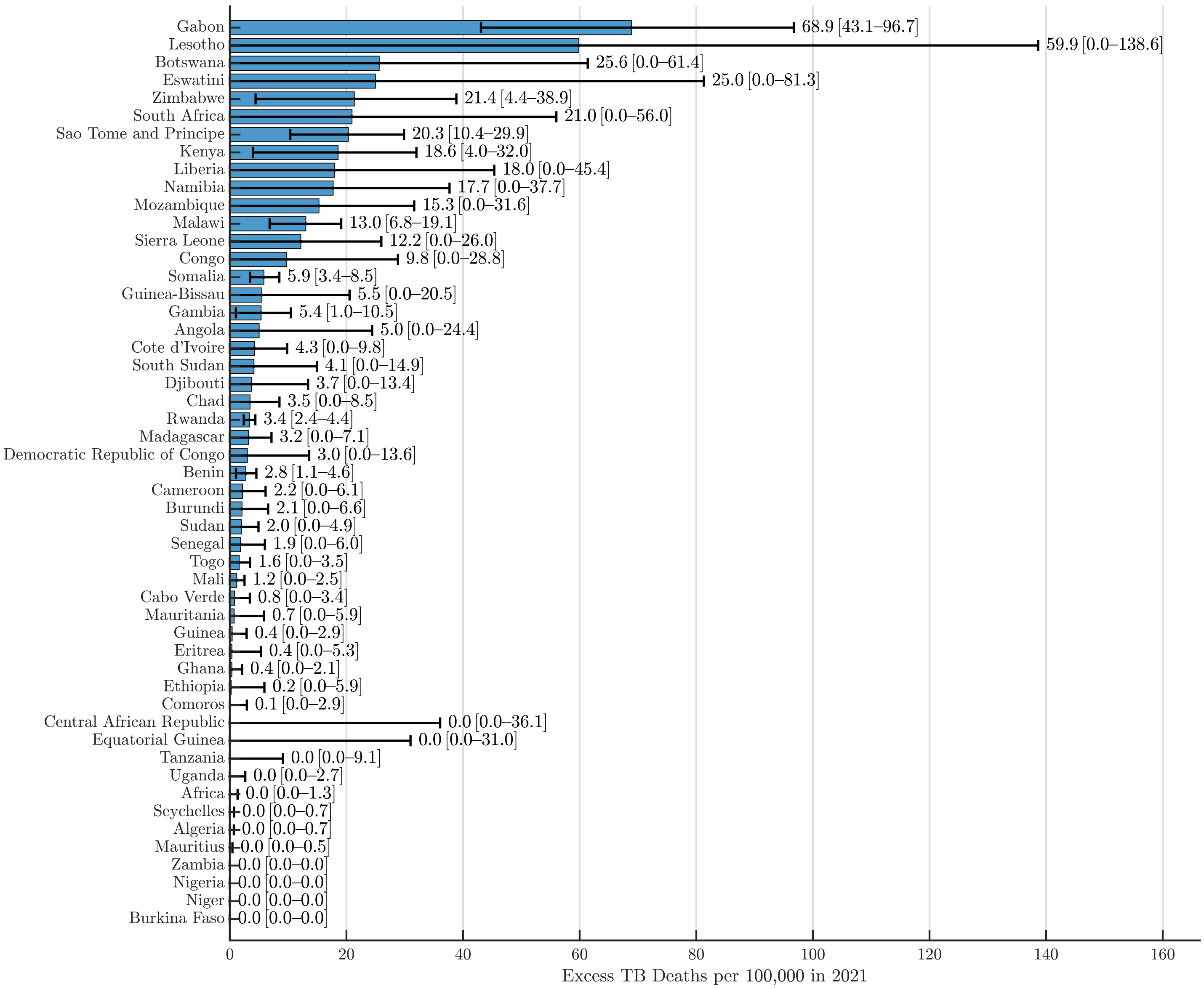


Figure S56: Excess TB mortality rate per 100,000 in Africa in 2021, estimated using an ensemble framework with two sub-epidemics. The horizontal bar charts display the median excess mortality rate for each country, with horizontal lines extending from each bar representing the corresponding confidence intervals (lower and upper bounds). Exact mortality rate numbers are also provided for each country.

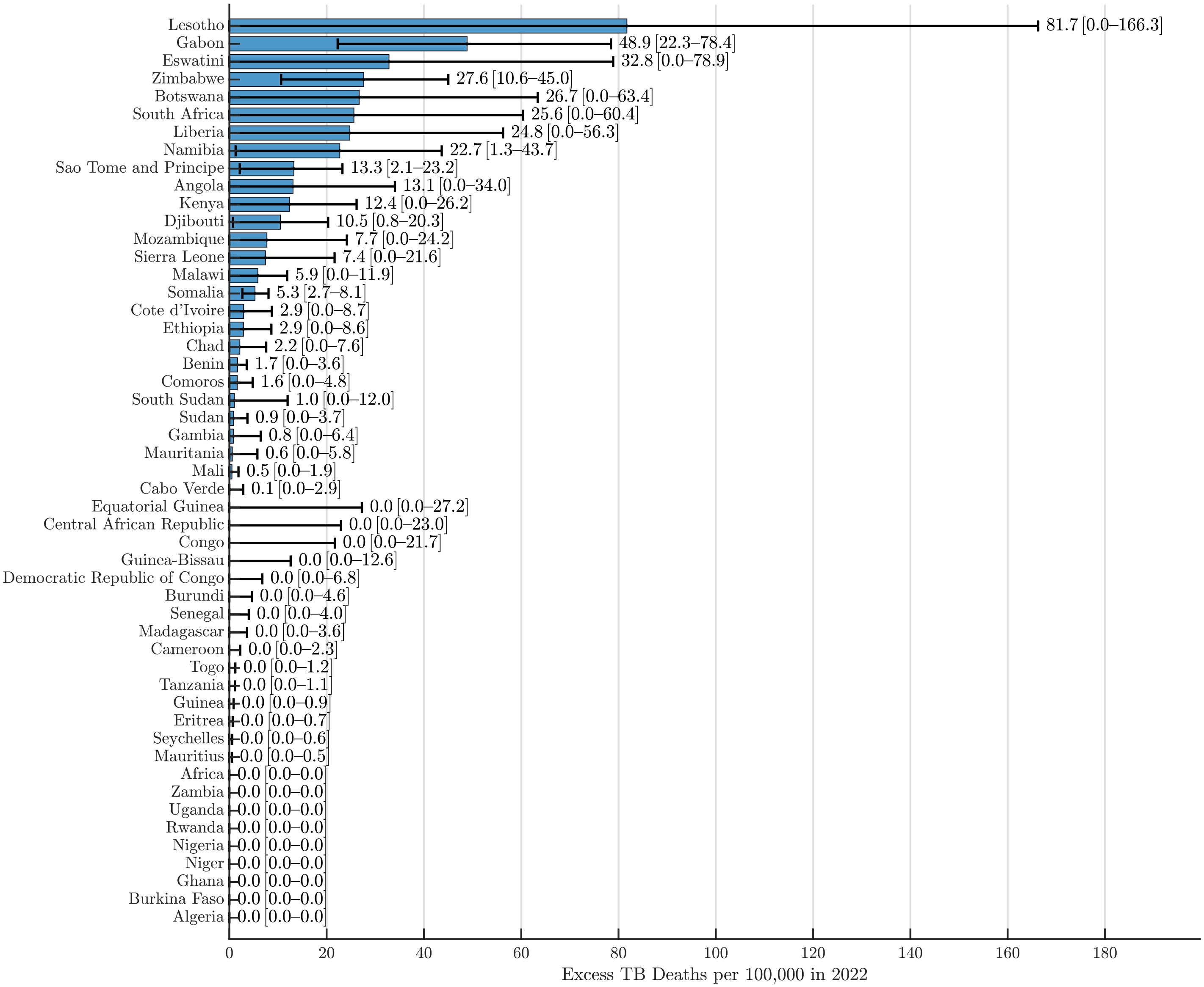


Figure S57: Excess TB mortality rate per 100,000 in Africa in 2022, estimated using an ensemble framework with two sub-epidemics. The horizontal bar charts display the median excess mortality rate for each country, with horizontal lines extending from each bar representing the corresponding confidence intervals (lower and upper bounds). Exact mortality rate numbers are also provided for each country.

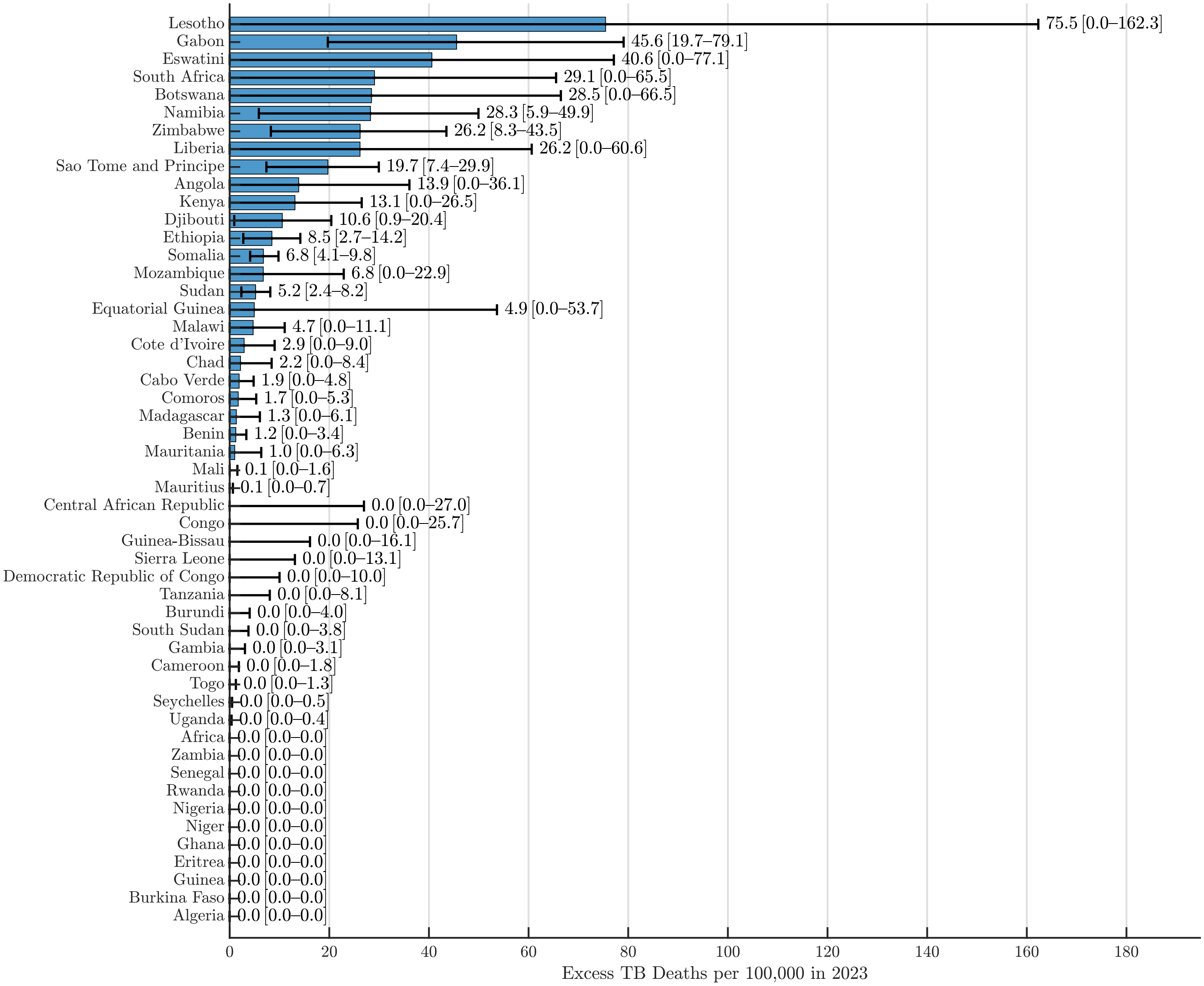


Figure S58: Excess TB mortality rate per 100,000 in Africa in 2023, estimated using an ensemble framework with two sub-epidemics. The horizontal bar charts display the median excess mortality rate for each country, with horizontal lines extending from each bar representing the corresponding confidence intervals (lower and upper bounds). Exact mortality rate numbers are also provided for each country.

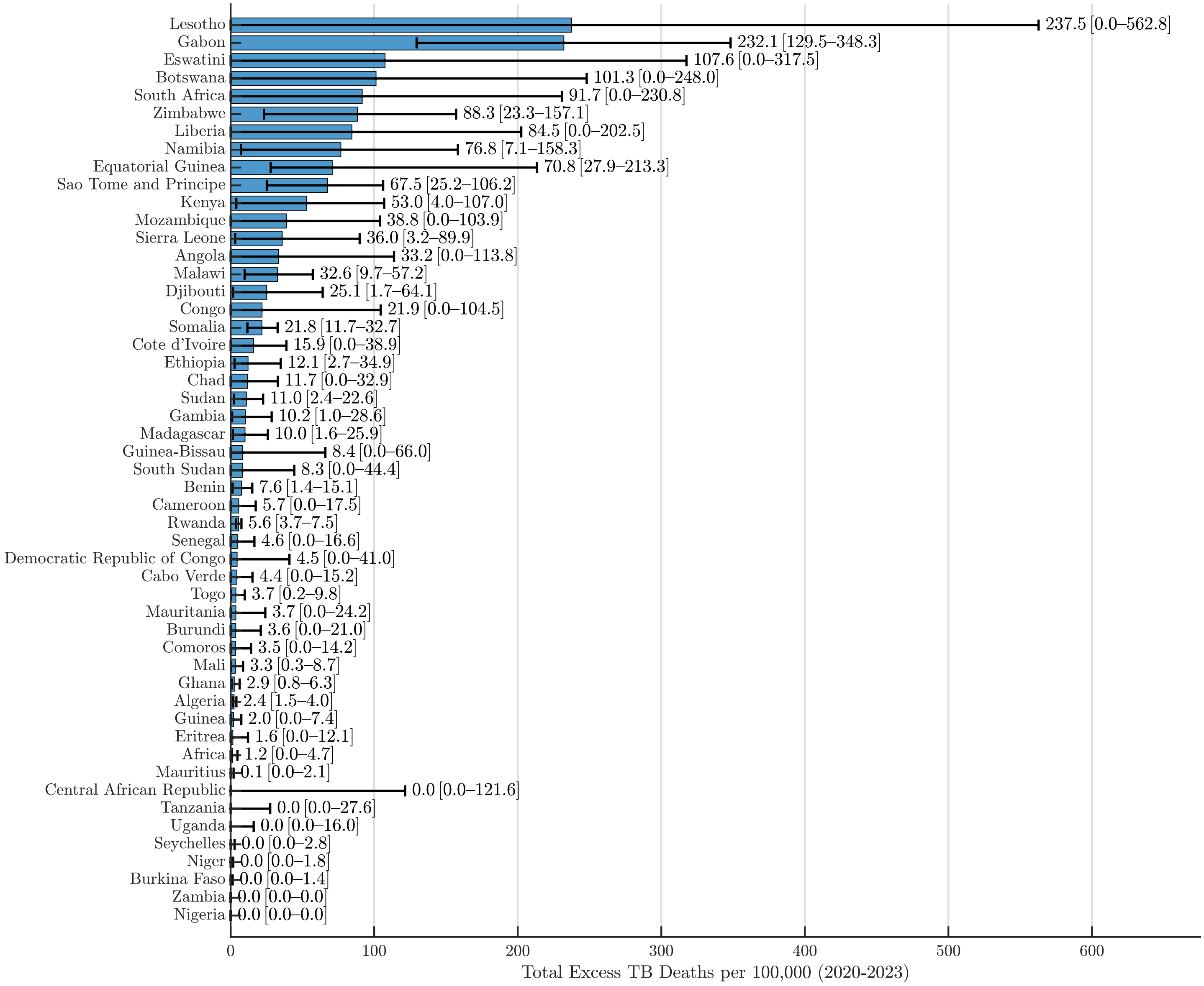


Figure S59: Total excess TB mortality rate per 100,000 in Africa in 2020-2023, estimated using an ensemble framework with two sub-epidemics. The horizontal bar charts display the median excess mortality rate for each country, with horizontal lines extending from each bar representing the corresponding confidence intervals (lower and upper bounds). Exact mortality rate numbers are also provided for each country.

| | Excess TB mortality rate (LB,UB) | | | | |
|---|---|---|---|---|---|
| Country | **2020** | **2021** | **2022** | **2023** | **Total** |
| **Algeria** | 2.4 (1.5, 3.3) | 0.0 (0.0, 0.7) | 0.0 (0.0, 0.0) | 0.0 (0.0, 0.0) | 2.4 (1.5, 4.0) |
| **Angola** | 1.2 (0.0, 19.3) | 5.0 (0.0, 24.4) | 13.1 (0.0, 34.0) | 13.9 (0.0, 36.1) | 33.2 (0.0, 113.8) |
| **Benin** | 2.0 (0.3, 3.6) | 2.8 (1.1, 4.6) | 1.7 (0.0, 3.6) | 1.2 (0.0, 3.4) | 7.6 (1.4, 15.1) |
| **Botswana** | 20.5 (0.0, 56.8) | 25.6 (0.0, 61.4) | 26.7 (0.0, 63.4) | 28.5 (0.0, 66.5) | 101.3 (0.0, 248.0) |
| **Burkina Faso** | 0.0 (0.0, 1.4) | 0.0 (0.0, 0.0) | 0.0 (0.0, 0.0) | 0.0 (0.0, 0.0) | 0.0 (0.0, 1.4) |
| **Burundi** | 1.5 (0.0, 5.8) | 2.1 (0.0, 6.6) | 0.0 (0.0, 4.6) | 0.0 (0.0, 4.0) | 3.6 (0.0, 21.0) |
| **Cabo Verde** | 1.5 (0.0, 4.1) | 0.8 (0.0, 3.4) | 0.1 (0.0, 2.9) | 1.9 (0.0, 4.8) | 4.4 (0.0, 15.2) |
| **Cameroon** | 3.5 (0.0, 7.2) | 2.2 (0.0, 6.1) | 0.0 (0.0, 2.3) | 0.0 (0.0, 1.8) | 5.7 (0.0, 17.5) |
| **Central African Republic** | 0.0 (0.0, 35.6) | 0.0 (0.0, 36.1) | 0.0 (0.0, 23.0) | 0.0 (0.0, 27.0) | 0.0 (0.0, 121.6) |
| **Chad** | 3.9 (0.0, 8.4) | 3.5 (0.0, 8.5) | 2.2 (0.0, 7.6) | 2.2 (0.0, 8.4) | 11.7 (0.0, 32.9) |
| **Comoros** | 0.0 (0.0, 1.2) | 0.1 (0.0, 2.9) | 1.6 (0.0, 4.8) | 1.7 (0.0, 5.3) | 3.5 (0.0, 14.2) |
| **Congo** | 12.1 (0.0, 28.3) | 9.8 (0.0, 28.8) | 0.0 (0.0, 21.7) | 0.0 (0.0, 25.7) | 21.9 (0.0, 104.5) |
| **Cote d'Ivoire** | 5.8 (0.0, 11.2) | 4.3 (0.0, 9.8) | 2.9 (0.0, 8.7) | 2.9 (0.0, 9.0) | 15.9 (0.0, 38.9) |
| **Democratic Republic of Congo** | 1.5 (0.0, 10.6) | 3.0 (0.0, 13.6) | 0.0 (0.0, 6.8) | 0.0 (0.0, 10.0) | 4.5 (0.0, 41.0) |
| **Djibouti** | 0.3 (0.0, 10.0) | 3.7 (0.0, 13.4) | 10.5 (0.8, 20.3) | 10.6 (0.9, 20.4) | 25.1 (1.7, 64.1) |
| **Guinea** | 1.6 (0.0, 3.7) | 0.4 (0.0, 2.9) | 0.0 (0.0, 0.9) | 0.0 (0.0, 0.0) | 2.0 (0.0, 7.4) |
| **Eritrea** | 1.2 (0.0, 6.1) | 0.4 (0.0, 5.3) | 0.0 (0.0, 0.7) | 0.0 (0.0, 0.0) | 1.6 (0.0, 12.1) |
| **Eswatini** | 9.2 (0.0, 80.2) | 25.0 (0.0, 81.3) | 32.8 (0.0, 78.9) | 40.6 (0.0, 77.1) | 107.6 (0.0, 317.5) |
| **Ethiopia** | 0.5 (0.0, 6.1) | 0.2 (0.0, 5.9) | 2.9 (0.0, 8.6) | 8.5 (2.7, 14.2) | 12.1 (2.7, 34.9) |
| **Gabon** | 68.8 (44.5, 94.0) | 68.9 (43.1, 96.7) | 48.9 (22.3, 78.4) | 45.6 (19.7, 79.1) | 232.1 (129.5, 348.3) |
| **Gambia** | 4.0 (0.0, 8.6) | 5.4 (1.0, 10.5) | 0.8 (0.0, 6.4) | 0.0 (0.0, 3.1) | 10.2 (1.0, 28.6) |
| **Ghana** | 2.5 (0.8, 4.2) | 0.4 (0.0, 2.1) | 0.0 (0.0, 0.0) | 0.0 (0.0, 0.0) | 2.9 (0.8, 6.3) |
| **Equatorial Guinea** | 65.9 (27.9, 101.5) | 0.0 (0.0, 31.0) | 0.0 (0.0, 27.2) | 4.9 (0.0, 53.7) | 70.8 (27.9, 213.3) |
| **Guinea-Bissau** | 2.9 (0.0, 16.7) | 5.5 (0.0, 20.5) | 0.0 (0.0, 12.6) | 0.0 (0.0, 16.1) | 8.4 (0.0, 66.0) |
| **Kenya** | 8.9 (0.0, 22.3) | 18.6 (4.0, 32.0) | 12.4 (0.0, 26.2) | 13.1 (0.0, 26.5) | 53.0 (4.0, 107.0) |
| **Lesotho** | 20.4 (0.0, 95.6) | 59.9 (0.0, 138.6) | 81.7 (0.0, 166.3) | 75.5 (0.0, 162.3) | 237.5 (0.0, 562.8) |
| **Liberia** | 15.6 (0.0, 40.2) | 18.0 (0.0, 45.4) | 24.8 (0.0, 56.3) | 26.2 (0.0, 60.6) | 84.5 (0.0, 202.5) |
| **Madagascar** | 5.4 (1.6, 9.1) | 3.2 (0.0, 7.1) | 0.0 (0.0, 3.6) | 1.3 (0.0, 6.1) | 10.0 (1.6, 25.9) |
| **Malawi** | 9.0 (2.9, 15.2) | 13.0 (6.8, 19.1) | 5.9 (0.0, 11.9) | 4.7 (0.0, 11.1) | 32.6 (9.7, 57.2) |
| **Mali** | 1.5 (0.3, 2.7) | 1.2 (0.0, 2.5) | 0.5 (0.0, 1.9) | 0.1 (0.0, 1.6) | 3.3 (0.3, 8.7) |
| **Mauritania** | 1.3 (0.0, 6.2) | 0.7 (0.0, 5.9) | 0.6 (0.0, 5.8) | 1.0 (0.0, 6.3) | 3.7 (0.0, 24.2) |
| **Mauritius** | 0.0 (0.0, 0.4) | 0.0 (0.0, 0.5) | 0.0 (0.0, 0.5) | 0.1 (0.0, 0.7) | 0.1 (0.0, 2.1) |
| **Mozambique** | 9.1 (0.0, 25.3) | 15.3 (0.0, 31.6) | 7.7 (0.0, 24.2) | 6.8 (0.0, 22.9) | 38.8 (0.0, 103.9) |
| **Namibia** | 8.1 (0.0, 27.0) | 17.7 (0.0, 37.7) | 22.7 (1.3, 43.7) | 28.3 (5.9, 49.9) | 76.8 (7.1, 158.3) |
| **Niger** | 0.0 (0.0, 1.8) | 0.0 (0.0, 0.0) | 0.0 (0.0, 0.0) | 0.0 (0.0, 0.0) | 0.0 (0.0, 1.8) |
| **Nigeria** | 0.0 (0.0, 0.0) | 0.0 (0.0, 0.0) | 0.0 (0.0, 0.0) | 0.0 (0.0, 0.0) | 0.0 (0.0, 0.0) |
| **Rwanda** | 2.2 (1.3, 3.1) | 3.4 (2.4, 4.4) | 0.0 (0.0, 0.0) | 0.0 (0.0, 0.0) | 5.6 (3.7, 7.5) |
| **Sao Tome and Principe** | 14.1 (5.3, 23.2) | 20.3 (10.4, 29.9) | 13.3 (2.1, 23.2) | 19.7 (7.4, 29.9) | 67.5 (25.2, 106.2) |
| **Senegal** | 2.8 (0.0, 6.5) | 1.9 (0.0, 6.0) | 0.0 (0.0, 4.0) | 0.0 (0.0, 0.0) | 4.6 (0.0, 16.6) |
| **Seychelles** | 0.0 (0.0, 0.9) | 0.0 (0.0, 0.7) | 0.0 (0.0, 0.6) | 0.0 (0.0, 0.5) | 0.0 (0.0, 2.8) |
| **Sierra Leone** | 16.4 (3.2, 29.2) | 12.2 (0.0, 26.0) | 7.4 (0.0, 21.6) | 0.0 (0.0, 13.1) | 36.0 (3.2, 89.9) |
| **Somalia** | 3.9 (1.4, 6.4) | 5.9 (3.4, 8.5) | 5.3 (2.7, 8.1) | 6.8 (4.1, 9.8) | 21.8 (11.7, 32.7) |
| **South Africa** | 16.0 (0.0, 48.9) | 21.0 (0.0, 56.0) | 25.6 (0.0, 60.4) | 29.1 (0.0, 65.5) | 91.7 (0.0, 230.8) |
| **South Sudan** | 3.1 (0.0, 13.7) | 4.1 (0.0, 14.9) | 1.0 (0.0, 12.0) | 0.0 (0.0, 3.8) | 8.3 (0.0, 44.4) |
| **Sudan** | 2.9 (0.1, 5.8) | 2.0 (0.0, 4.9) | 0.9 (0.0, 3.7) | 5.2 (2.4, 8.2) | 11.0 (2.4, 22.6) |
| **Togo** | 2.1 (0.2, 3.9) | 1.6 (0.0, 3.5) | 0.0 (0.0, 1.2) | 0.0 (0.0, 1.3) | 3.7 (0.2, 9.8) |
| **Uganda** | 0.0 (0.0, 13.0) | 0.0 (0.0, 2.7) | 0.0 (0.0, 0.0) | 0.0 (0.0, 0.4) | 0.0 (0.0, 16.0) |
| **Tanzania** | 0.0 (0.0, 9.3) | 0.0 (0.0, 9.1) | 0.0 (0.0, 1.1) | 0.0 (0.0, 8.1) | 0.0 (0.0, 27.6) |
| **Zambia** | 0.0 (0.0, 0.0) | 0.0 (0.0, 0.0) | 0.0 (0.0, 0.0) | 0.0 (0.0, 0.0) | 0.0 (0.0, 0.0) |
| **Zimbabwe** | 13.1 (0.0, 29.7) | 21.4 (4.4, 38.9) | 27.6 (10.6, 45.0) | 26.2 (8.3, 43.5) | 88.3 (23.3, 157.1) |
| **Africa** | 1.2 (0.0, 3.4) | 0.0 (0.0, 1.3) | 0.0 (0.0, 0.0) | 0.0 (0.0, 0.0) | 1.2 (0.0, 4.7) |

Table S8: Estimated excess TB mortality rate in Africa for the years 2020 to 2023, presented both individually by year and as a total. The estimates include the median value along with the corresponding lower and upper bounds, calculated using an ensemble framework that models two distinct sub-epidemics.

### S4.3 SMR

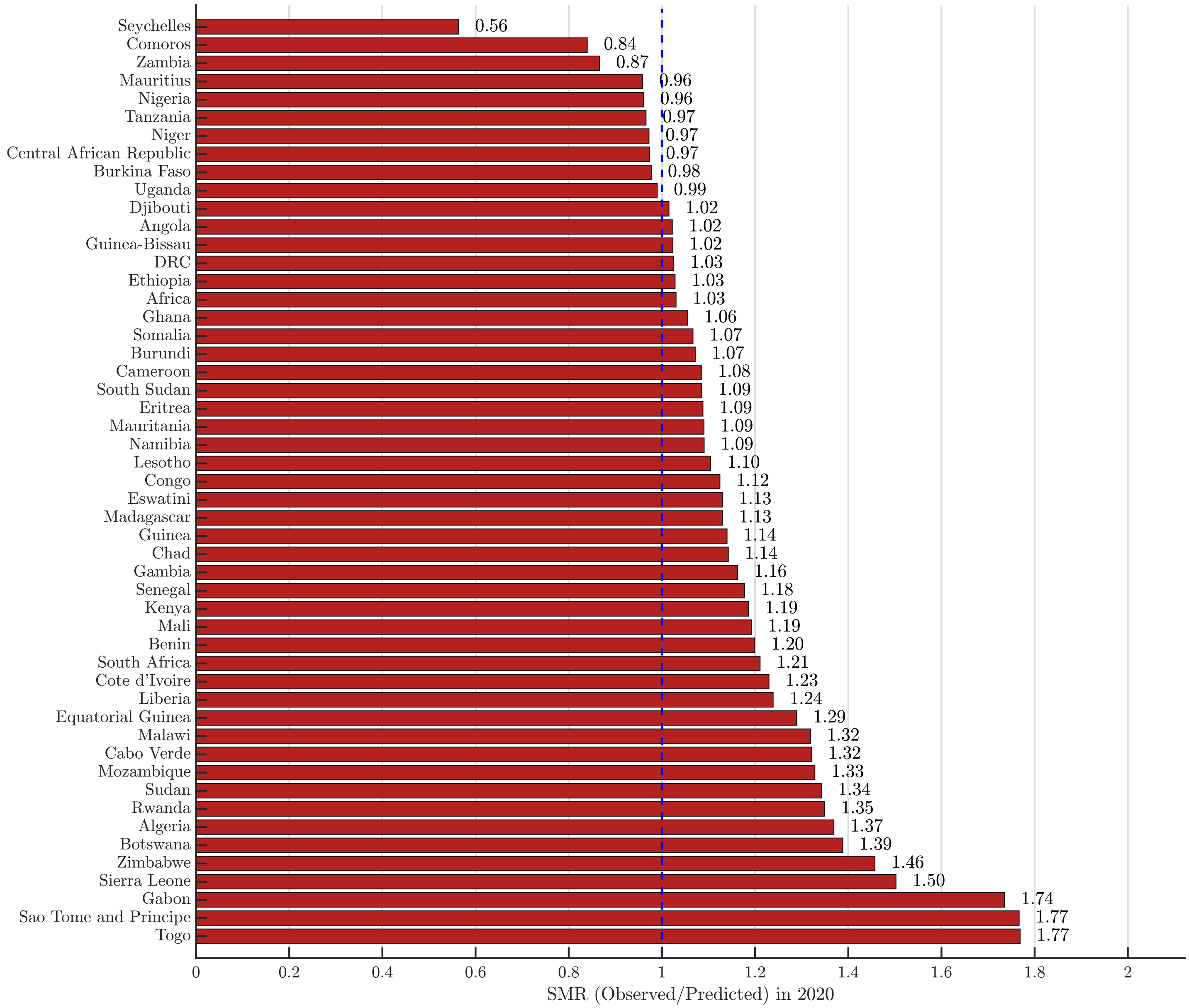


Figure S60: SMR in Africa in 2020, estimated using an ensemble framework with two sub-epidemics. SMR is displayed horizontally in bar charts, with the exact number displayed in front of the bars.

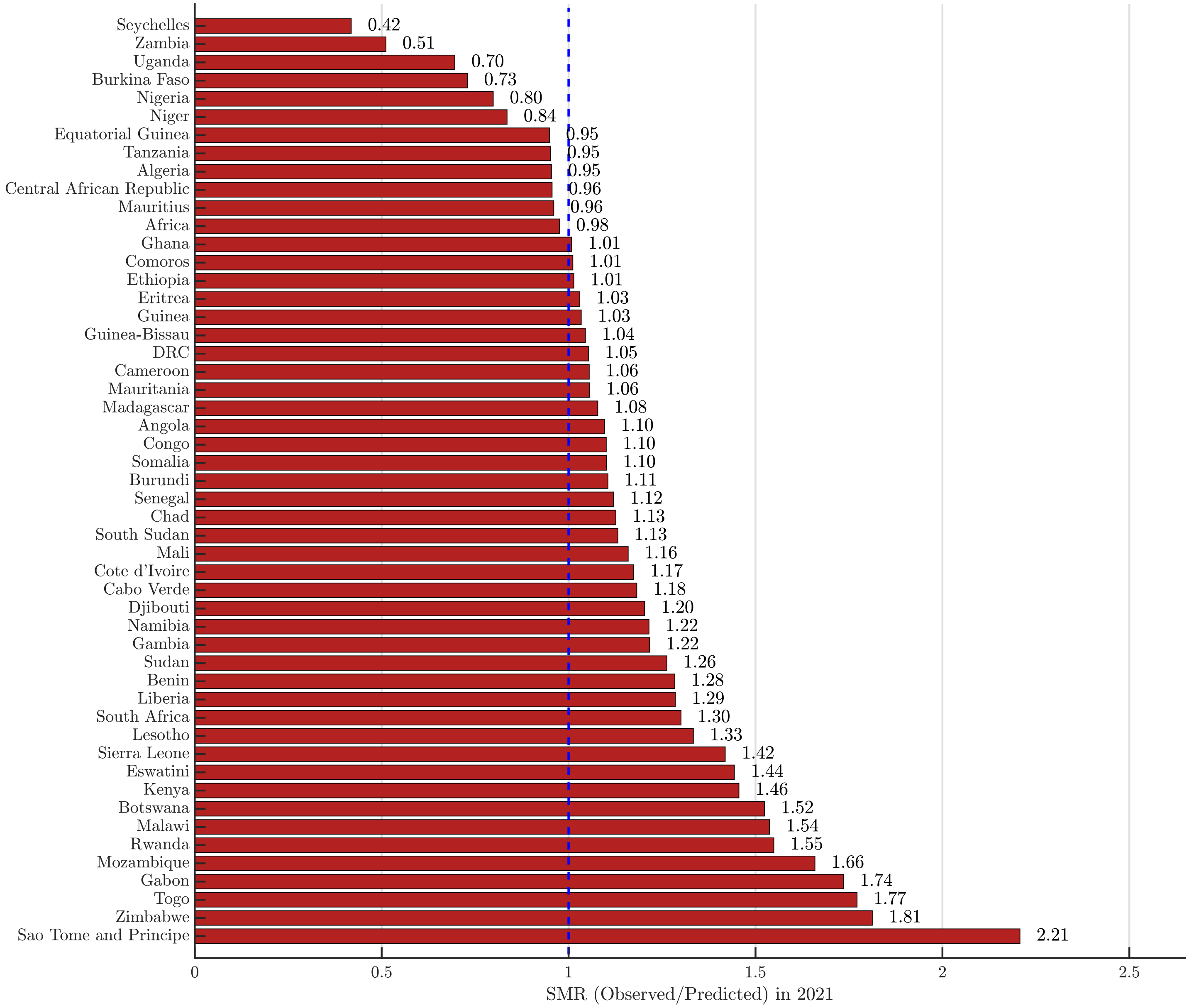


Figure S61: SMR in Africa in 2021, estimated using an ensemble framework with two sub-epidemics. SMR is displayed horizontally in bar charts, with the exact number displayed in front of the bars.

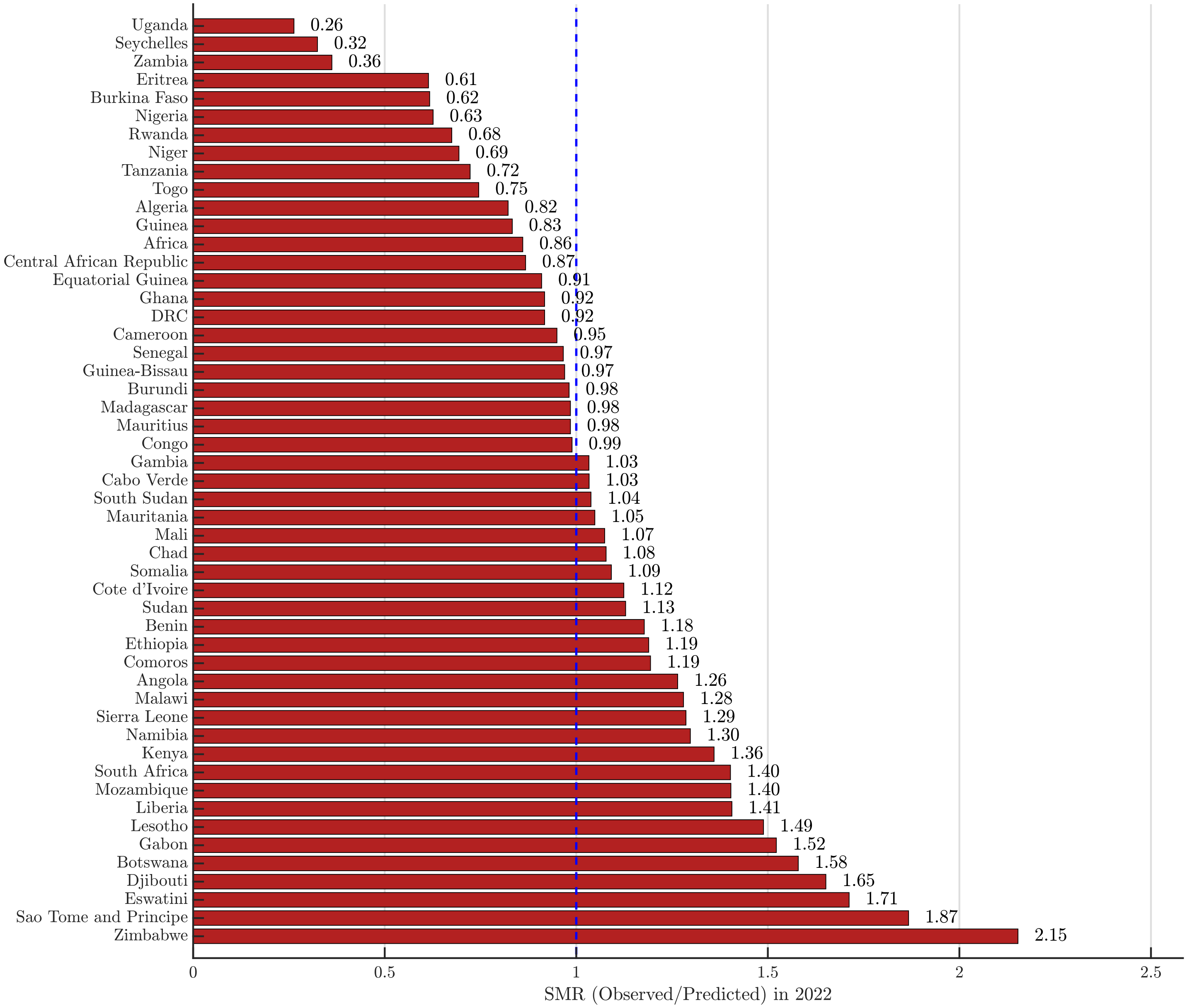


Figure S62: SMR in Africa in 2022, estimated using an ensemble framework with two sub-epidemics. SMR is displayed horizontally in bar charts, with the exact number displayed in front of the bars.

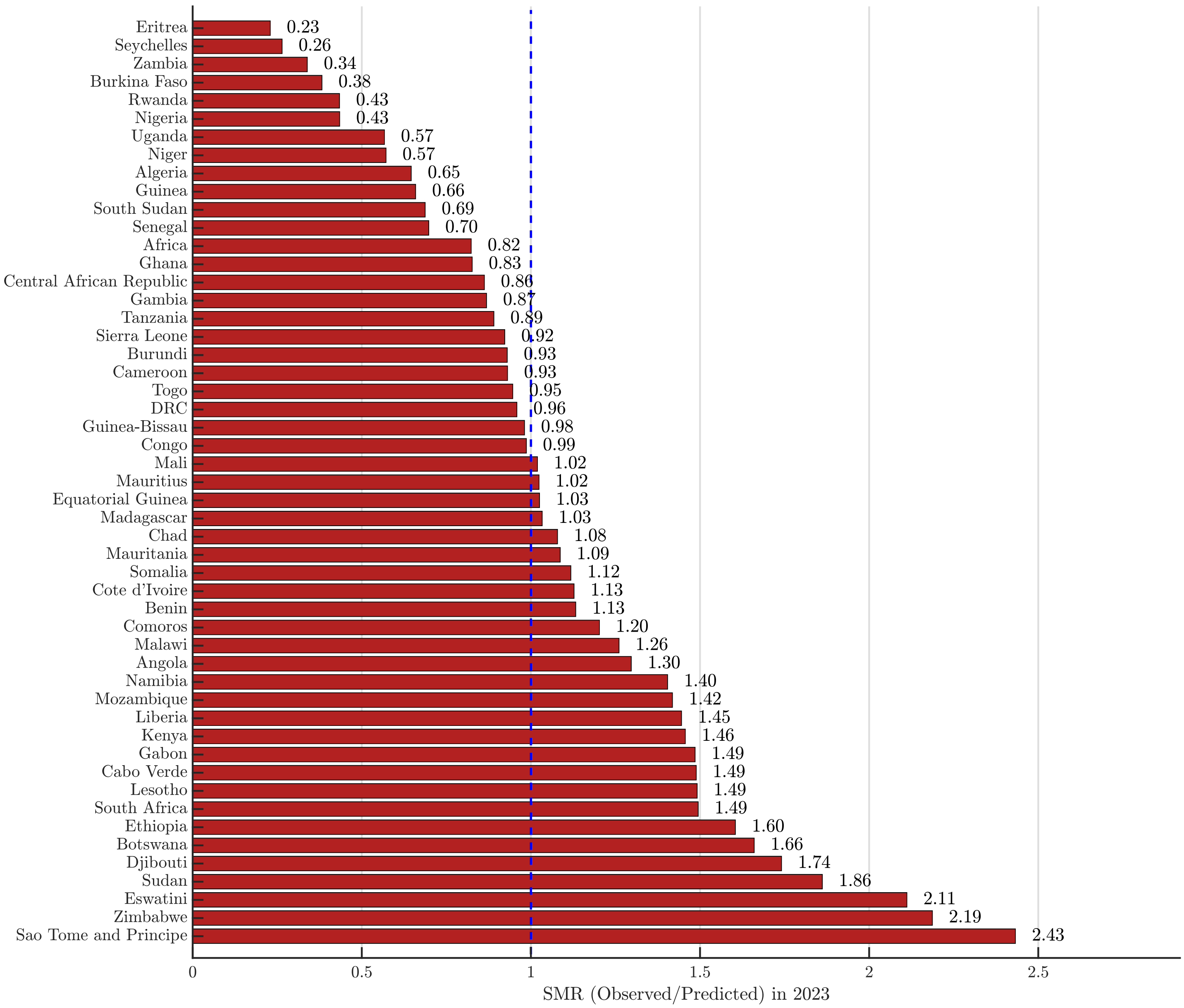


Figure S63: SMR in Africa in 2023, estimated using an ensemble framework with two sub-epidemics. SMR is displayed horizontally in bar charts, with the exact number displayed in front of the bars.

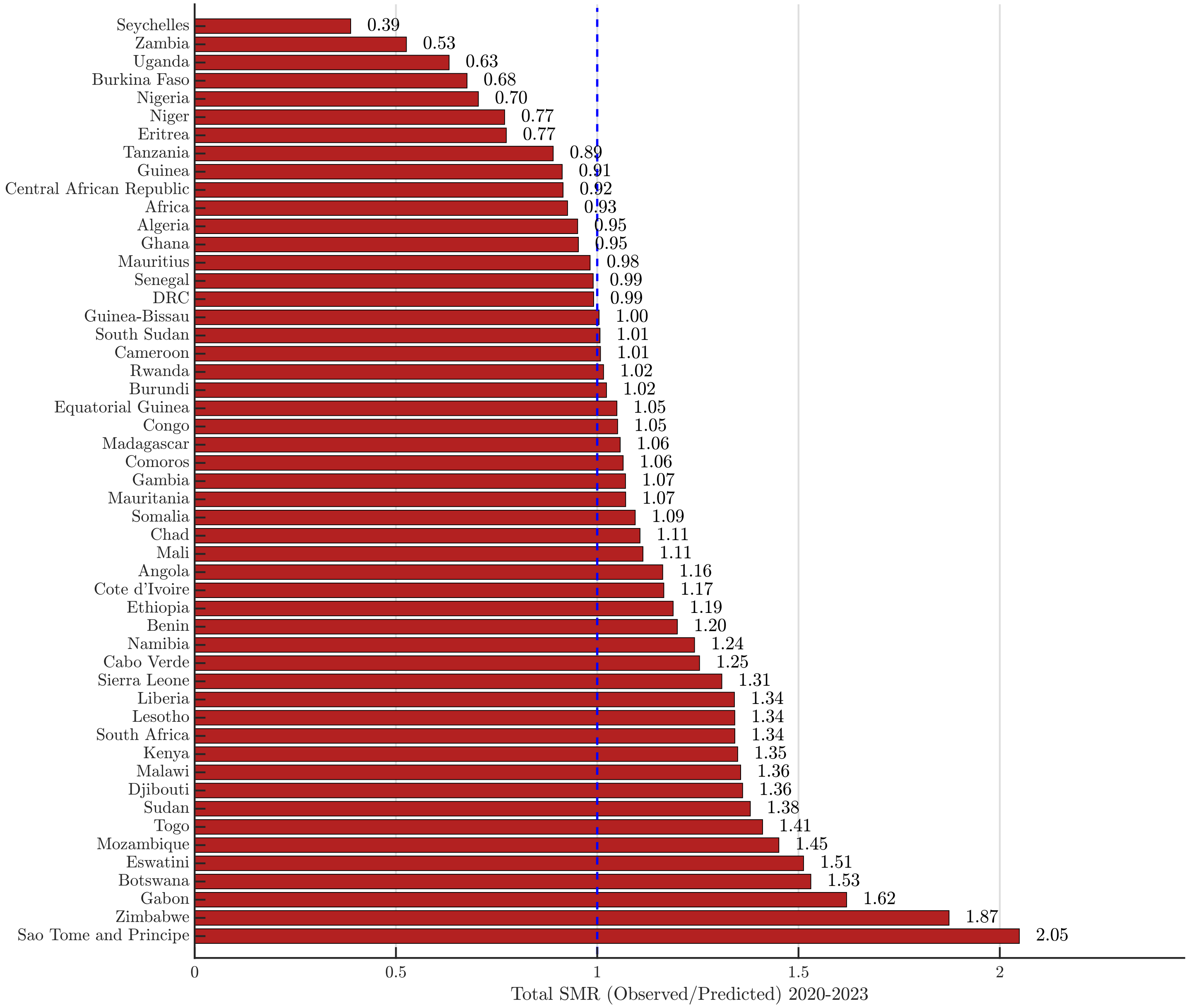


Figure S64: SMR in Africa during 2020-2023, estimated using an ensemble framework with two sub-epidemics. SMR is displayed horizontally in bar charts, with the exact number displayed in front of the bars.

# S5 Europe

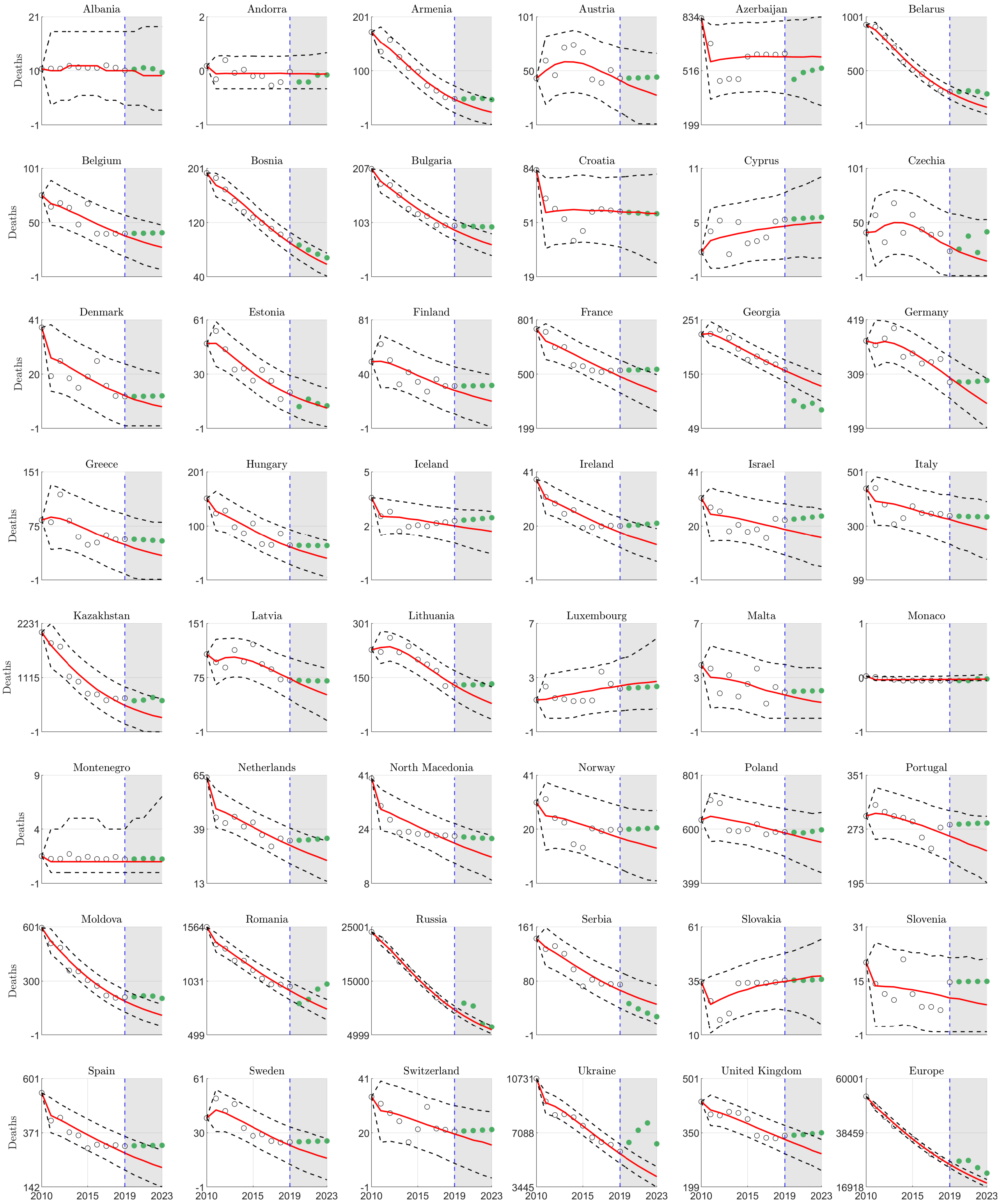


Figure S65: Forecasting panel for the number of deaths in Europe, based on a 10-year calibration period (2010–2019) and a four-year forecasting period (2020–2023), generated using the Ranked 1 method. The red curve denotes the median forecast, while the black dashed lines mark the 95% PI. Reported data points are shown as circles: black circles represent the calibration period, and green-filled circles represent actual data points during the forecast period.

## S5.1 Excess mortality

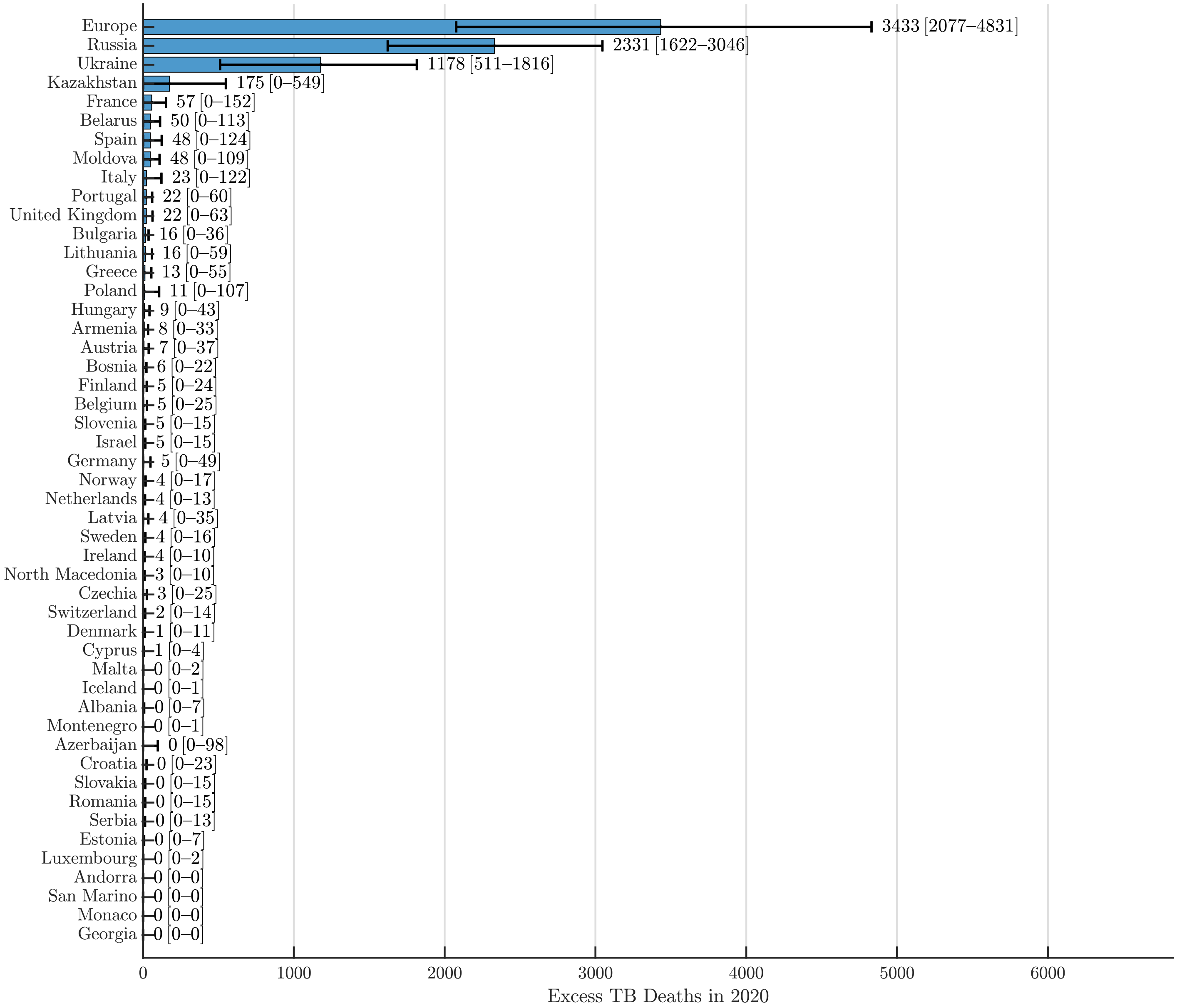


Figure S66: Excess TB mortality in Europe in 2020, estimated using an ensemble framework with two sub-epidemics. The horizontal bar charts display the median excess mortality for each country, with horizontal lines extending from each bar representing the corresponding confidence intervals (lower and upper bounds). Exact mortality numbers are also provided for each country.

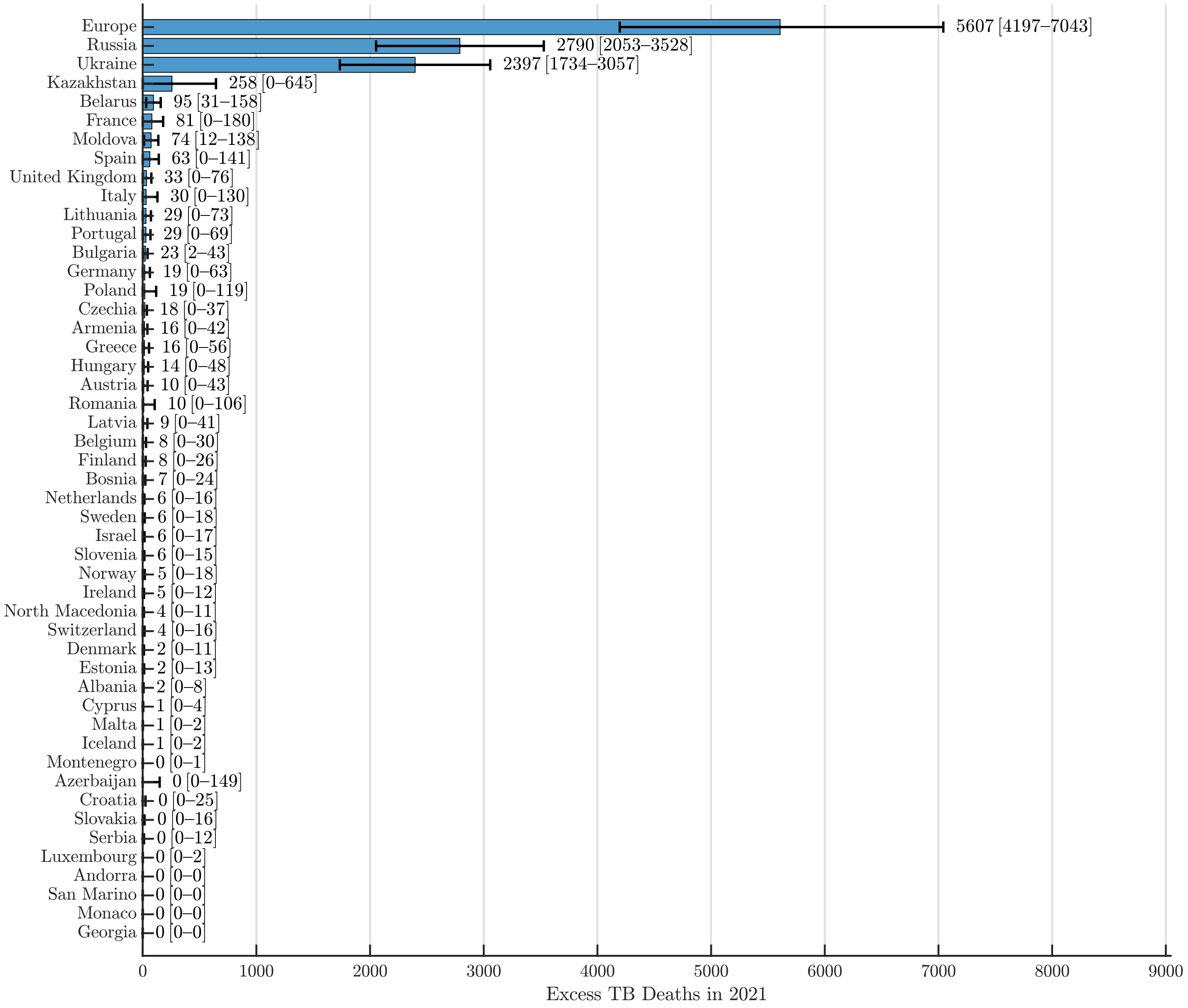


Figure S67: Excess TB mortality in Europe in 2021, estimated using an ensemble framework with two sub-epidemics. The horizontal bar charts display the median excess mortality for each country, with horizontal lines extending from each bar representing the corresponding confidence intervals (lower and upper bounds). Exact mortality numbers are also provided for each country.

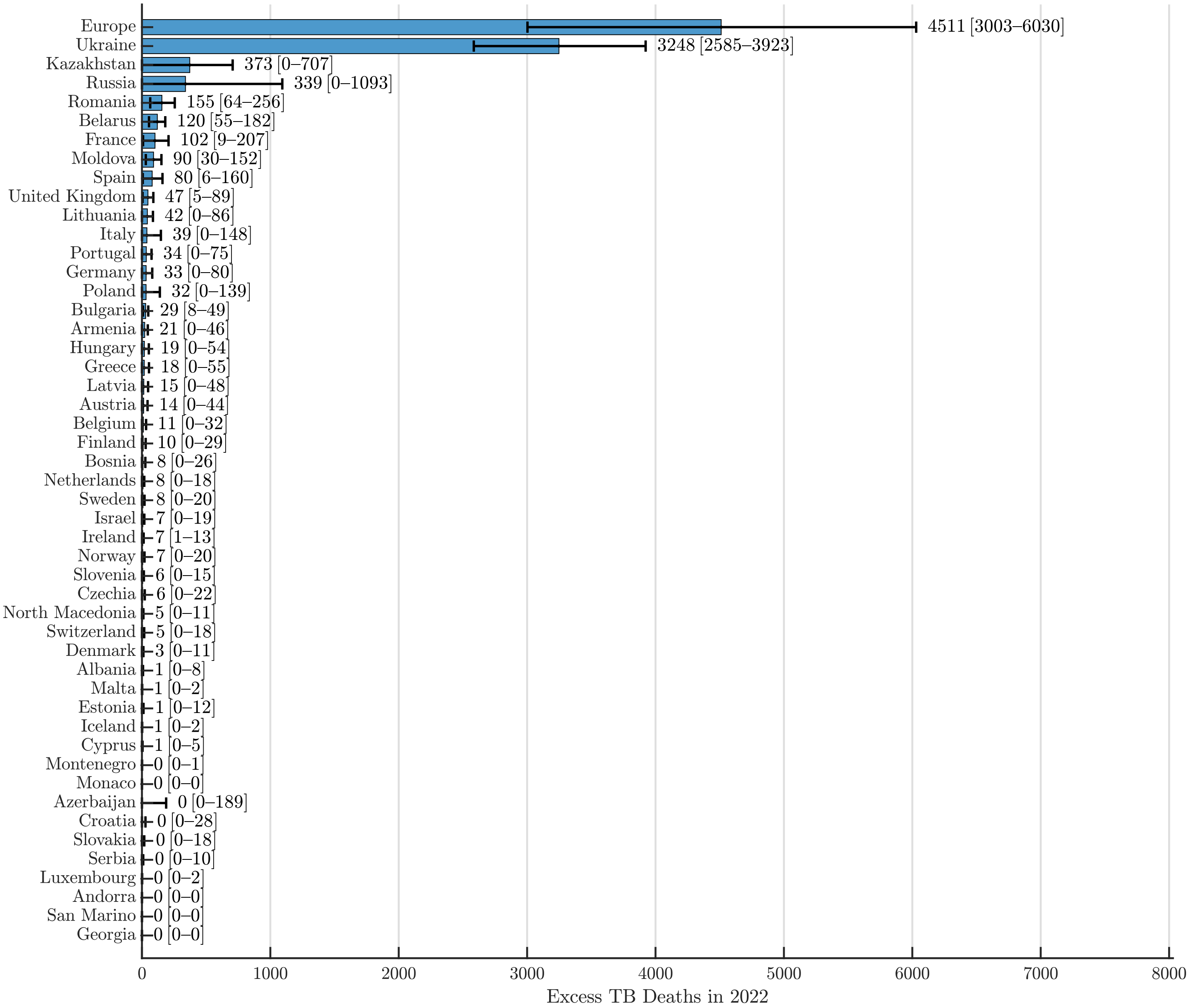


Figure S68: Excess TB mortality in Europe in 2022, estimated using an ensemble framework with two sub-epidemics. The horizontal bar charts display the median excess mortality for each country, with horizontal lines extending from each bar representing the corresponding confidence intervals (lower and upper bounds). Exact mortality numbers are also provided for each country.

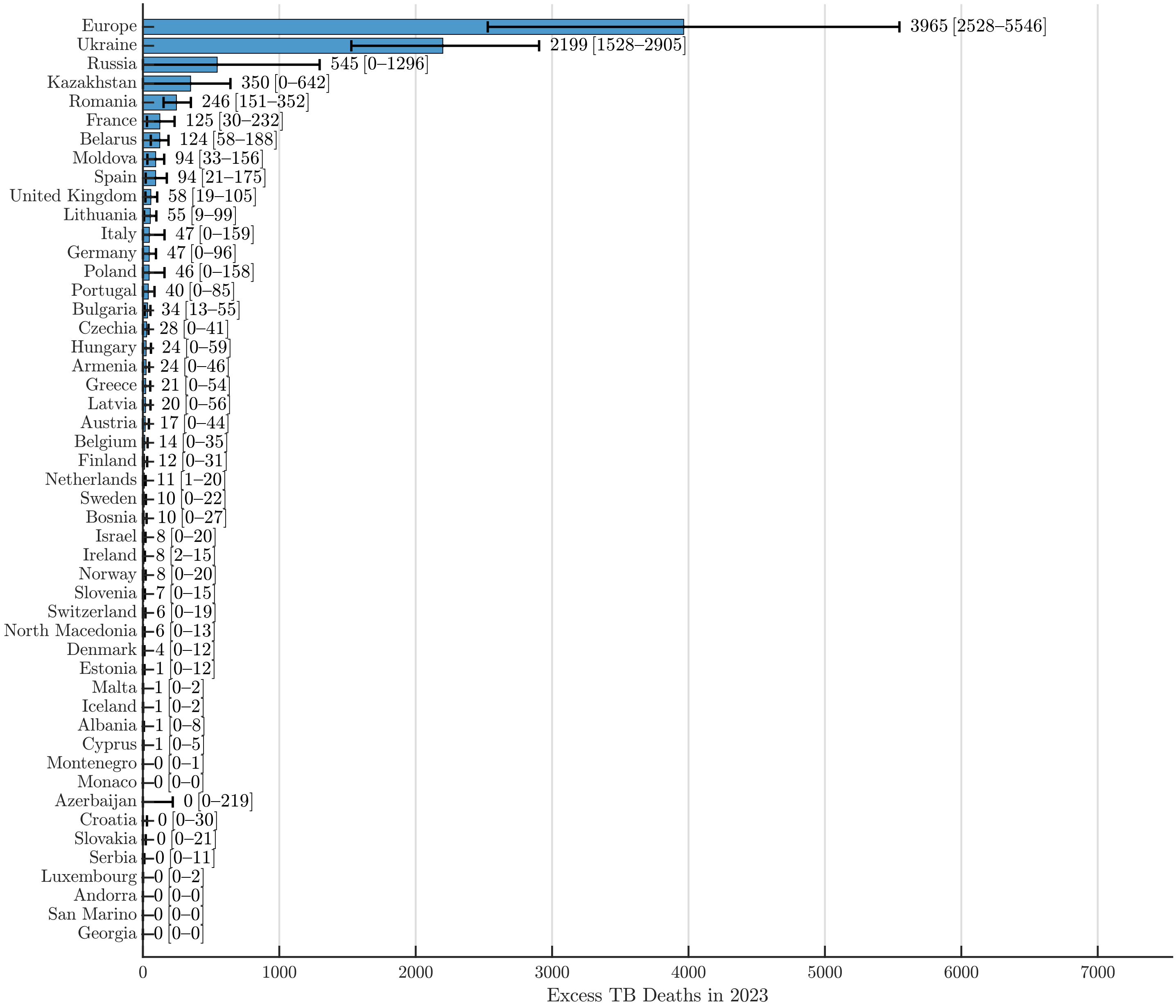


Figure S69: Excess TB mortality in Europe in 2023, estimated using an ensemble framework with two sub-epidemics. The horizontal bar charts display the median excess mortality for each country, with horizontal lines extending from each bar representing the corresponding confidence intervals (lower and upper bounds). Exact mortality numbers are also provided for each country.

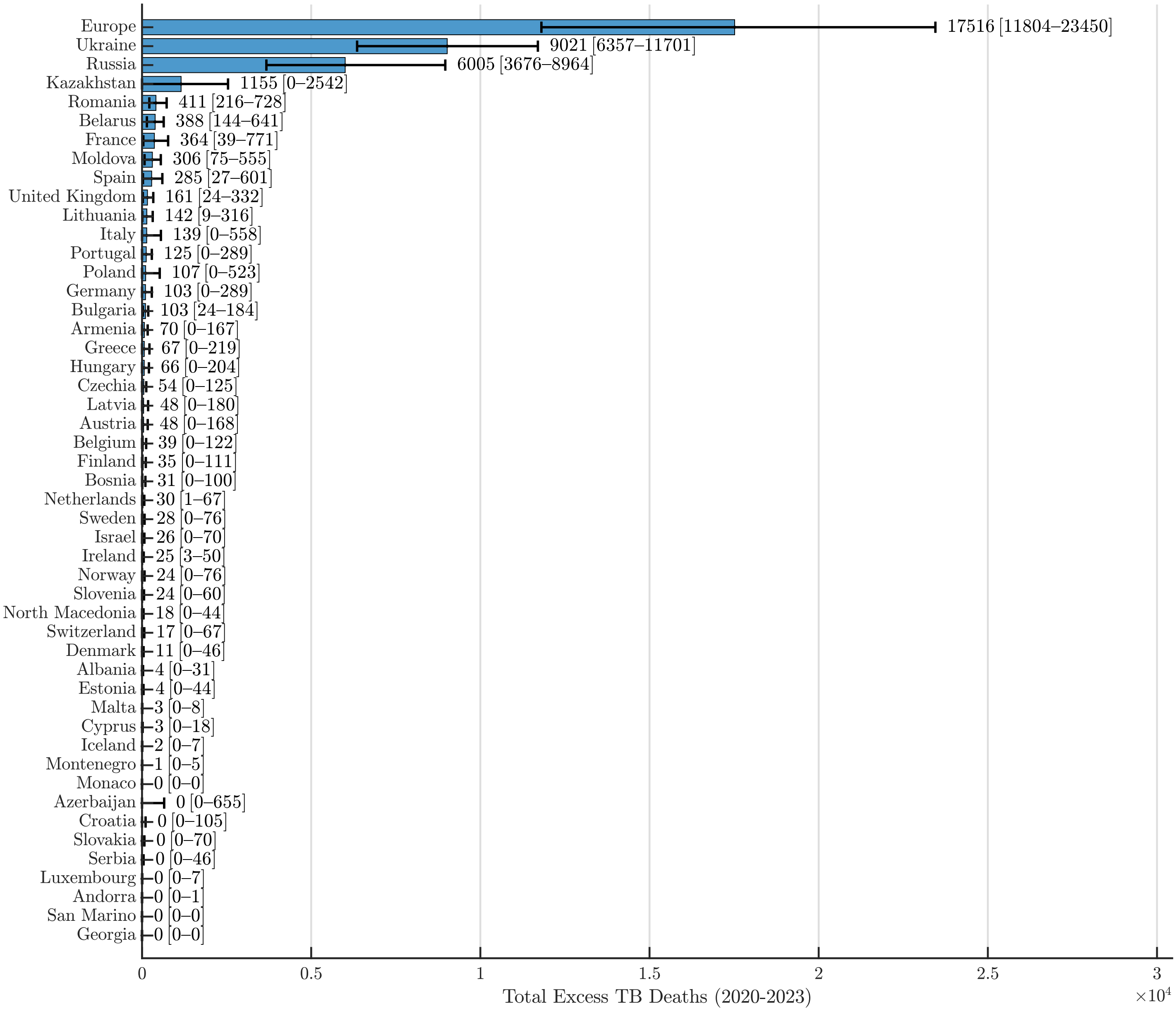


Figure S70: Total excess TB mortality in Europe in 2020-2023, estimated using an ensemble framework with two sub-epidemics. The horizontal bar charts display the median excess mortality for each country, with horizontal lines extending from each bar representing the corresponding confidence intervals (lower and upper bounds). Exact mortality numbers are also provided for each country.

| | Excess TB mortality (LB,UB) | | | | |
|---|---|---|---|---|---|
| Country | **2020** | **2021** | **2022** | **2023** | **Total** |
| **Albania** | 0 (0, 7) | 2 (0, 8) | 1 (0, 8) | 1 (0, 8) | 4 (0, 31) |
| **Andorra** | 0 (0, 0) | 0 (0, 0) | 0 (0, 0) | 0 (0, 0) | 0 (0, 1) |
| **Armenia** | 8 (0, 33) | 16 (0, 42) | 21 (0, 46) | 24 (0, 46) | 70 (0, 167) |
| **Austria** | 7 (0, 37) | 10 (0, 43) | 14 (0, 44) | 17 (0, 44) | 48 (0, 168) |
| **Azerbaijan** | 0 (0, 98) | 0 (0, 149) | 0 (0, 189) | 0 (0, 219) | 0 (0, 655) |
| **Belarus** | 50 (0, 113) | 95 (31, 158) | 120 (55, 182) | 124 (58, 188) | 388 (144, 641) |
| **Belgium** | 5 (0, 25) | 8 (0, 30) | 11 (0, 32) | 14 (0, 35) | 39 (0, 122) |
| **Bosnia** | 6 (0, 22) | 7 (0, 24) | 8 (0, 26) | 10 (0, 27) | 31 (0, 100) |
| **Bulgaria** | 16 (0, 36) | 23 (2, 43) | 29 (8, 49) | 34 (13, 55) | 103 (24, 184) |
| **Croatia** | 0 (0, 23) | 0 (0, 25) | 0 (0, 28) | 0 (0, 30) | 0 (0, 105) |
| **Cyprus** | 1 (0, 4) | 1 (0, 4) | 1 (0, 5) | 1 (0, 5) | 3 (0, 18) |
| **Czechia** | 3 (0, 25) | 18 (0, 37) | 6 (0, 22) | 28 (0, 41) | 54 (0, 125) |
| **Denmark** | 1 (0, 11) | 2 (0, 11) | 3 (0, 11) | 4 (0, 12) | 11 (0, 46) |
| **Estonia** | 0 (0, 7) | 2 (0, 13) | 1 (0, 12) | 1 (0, 12) | 4 (0, 44) |
| **Finland** | 5 (0, 24) | 8 (0, 26) | 10 (0, 29) | 12 (0, 31) | 35 (0, 111) |
| **France** | 57 (0, 152) | 81 (0, 180) | 102 (9, 207) | 125 (30, 232) | 364 (39, 771) |
| **Georgia** | 0 (0, 0) | 0 (0, 0) | 0 (0, 0) | 0 (0, 0) | 0 (0, 0) |
| **Germany** | 5 (0, 49) | 19 (0, 63) | 33 (0, 80) | 47 (0, 96) | 103 (0, 289) |
| **Greece** | 13 (0, 55) | 16 (0, 56) | 18 (0, 55) | 21 (0, 54) | 67 (0, 219) |
| **Hungary** | 9 (0, 43) | 14 (0, 48) | 19 (0, 54) | 24 (0, 59) | 66 (0, 204) |
| **Iceland** | 0 (0, 1) | 1 (0, 2) | 1 (0, 2) | 1 (0, 2) | 2 (0, 7) |
| **Ireland** | 4 (0, 10) | 5 (0, 12) | 7 (1, 13) | 8 (2, 15) | 25 (3, 50) |
| **Israel** | 5 (0, 15) | 6 (0, 17) | 7 (0, 19) | 8 (0, 20) | 26 (0, 70) |
| **Italy** | 23 (0, 122) | 30 (0, 130) | 39 (0, 148) | 47 (0, 159) | 139 (0, 558) |
| **Kazakhstan** | 175 (0, 549) | 258 (0, 645) | 373 (0, 707) | 350 (0, 642) | 1155 (0, 2542) |
| **Latvia** | 4 (0, 35) | 9 (0, 41) | 15 (0, 48) | 20 (0, 56) | 48 (0, 180) |
| **Lithuania** | 16 (0, 59) | 29 (0, 73) | 42 (0, 86) | 55 (9, 99) | 142 (9, 316) |
| **Luxembourg** | 0 (0, 2) | 0 (0, 2) | 0 (0, 2) | 0 (0, 2) | 0 (0, 7) |
| **Malta** | 0 (0, 2) | 1 (0, 2) | 1 (0, 2) | 1 (0, 2) | 3 (0, 8) |
| **Monaco** | 0 (0, 0) | 0 (0, 0) | 0 (0, 0) | 0 (0, 0) | 0 (0, 0) |
| **Montenegro** | 0 (0, 1) | 0 (0, 1) | 0 (0, 1) | 0 (0, 1) | 1 (0, 5) |
| **Netherlands** | 4 (0, 13) | 6 (0, 16) | 8 (0, 18) | 11 (1, 20) | 30 (1, 67) |
| **North Macedonia** | 3 (0, 10) | 4 (0, 11) | 5 (0, 11) | 6 (0, 13) | 18 (0, 44) |
| **Norway** | 4 (0, 17) | 5 (0, 18) | 7 (0, 20) | 8 (0, 20) | 24 (0, 76) |
| **Poland** | 11 (0, 107) | 19 (0, 119) | 32 (0, 139) | 46 (0, 158) | 107 (0, 523) |
| **Portugal** | 22 (0, 60) | 29 (0, 69) | 34 (0, 75) | 40 (0, 85) | 125 (0, 289) |
| **Moldova** | 48 (0, 109) | 74 (12, 138) | 90 (30, 152) | 94 (33, 156) | 306 (75, 555) |
| **Romania** | 0 (0, 15) | 10 (0, 106) | 155 (64, 256) | 246 (151, 352) | 411 (216, 728) |
| **Russia** | 2331 (1622, 3046) | 2790 (2053, 3528) | 339 (0, 1093) | 545 (0, 1296) | 6005 (3676, 8964) |
| **Serbia** | 0 (0, 13) | 0 (0, 12) | 0 (0, 10) | 0 (0, 11) | 0 (0, 46) |
| **Slovakia** | 0 (0, 15) | 0 (0, 16) | 0 (0, 18) | 0 (0, 21) | 0 (0, 70) |
| **Slovenia** | 5 (0, 15) | 6 (0, 15) | 6 (0, 15) | 7 (0, 15) | 24 (0, 60) |
| **Spain** | 48 (0, 124) | 63 (0, 141) | 80 (6, 160) | 94 (21, 175) | 285 (27, 601) |
| **Sweden** | 4 (0, 16) | 6 (0, 18) | 8 (0, 20) | 10 (0, 22) | 28 (0, 76) |
| **Switzerland** | 2 (0, 14) | 4 (0, 16) | 5 (0, 18) | 6 (0, 19) | 17 (0, 67) |
| **Ukraine** | 1178 (511, 1816) | 2397 (1734, 3057) | 3248 (2585, 3923) | 2199 (1528, 2905) | 9021 (6357, 11701) |
| **United Kingdom** | 22 (0, 63) | 33 (0, 76) | 47 (5, 89) | 58 (19, 105) | 161 (24, 332) |
| **Europe** | 3433 (2077, 4831) | 5607 (4197, 7043) | 4511 (3003, 6030) | 3965 (2528, 5546) | 17516 (11804, 23450) |

Table S9: Estimated excess TB mortality in Europe for the years 2020 to 2023, presented both individually by year and as a total. The estimates include the median value along with the corresponding lower and upper bounds, calculated using an ensemble framework that models two distinct sub-epidemics.

## S5.2 Excess mortality rate

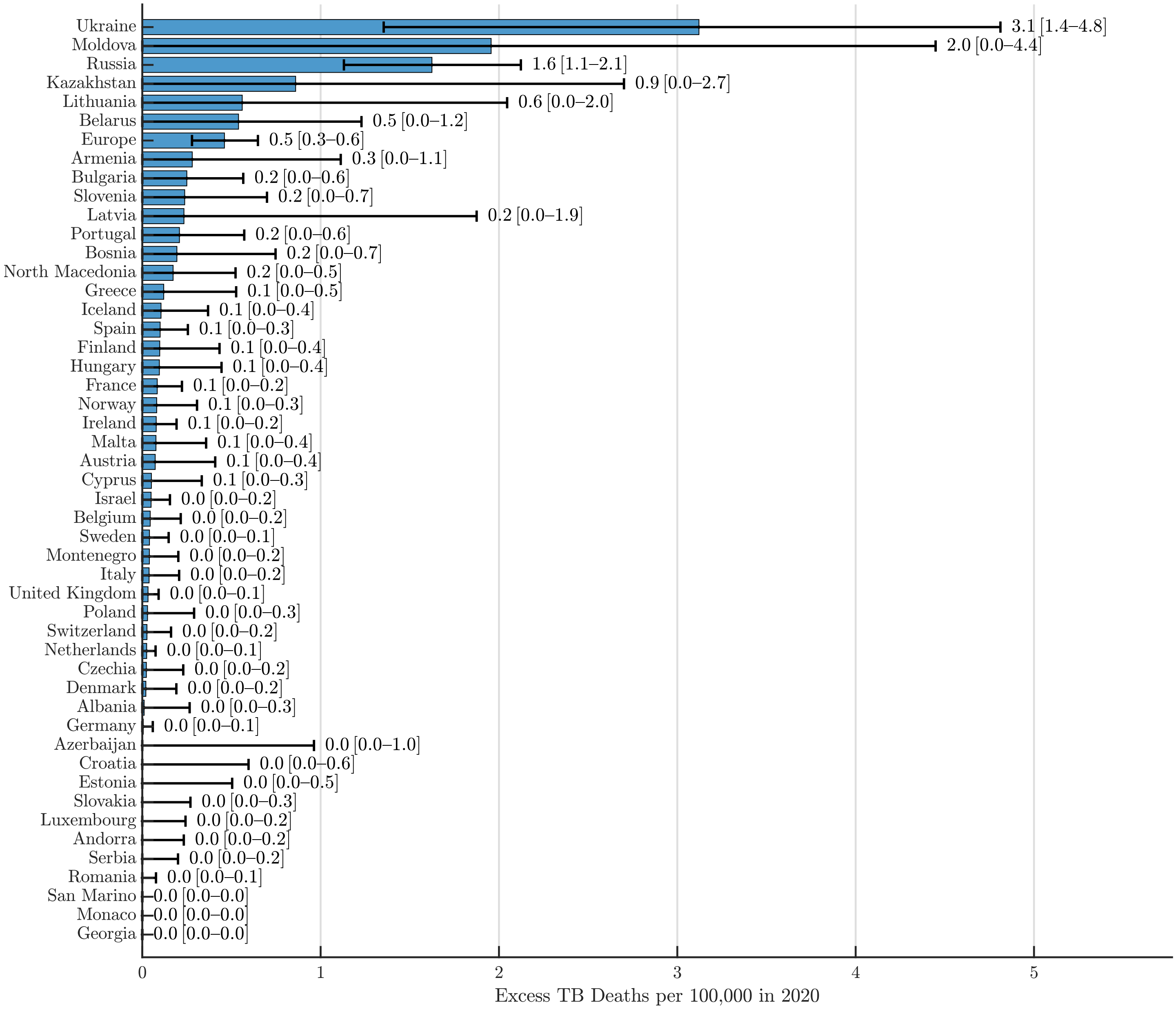


Figure S71: Excess TB mortality rate per 100,000 in Europe in 2020, estimated using an ensemble framework with two sub-epidemics. The horizontal bar charts display the median excess mortality rate for each country, with horizontal lines extending from each bar representing the corresponding confidence intervals (lower and upper bounds). Exact mortality rate numbers are also provided for each country.

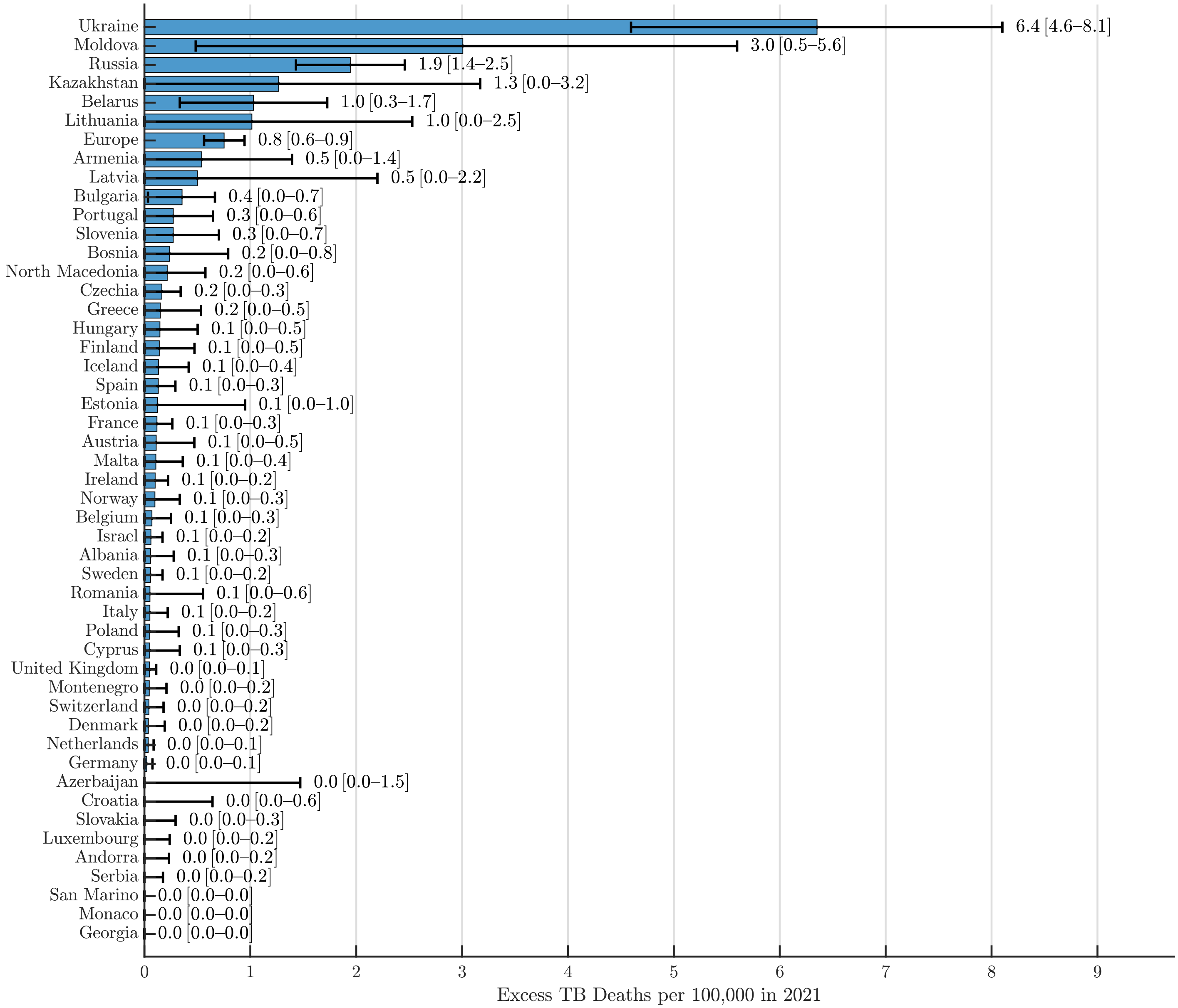


Figure S72: Excess TB mortality rate per 100,000 in Europe in 2021, estimated using an ensemble framework with two sub-epidemics. The horizontal bar charts display the median excess mortality rate for each country, with horizontal lines extending from each bar representing the corresponding confidence intervals (lower and upper bounds). Exact mortality rate numbers are also provided for each country.

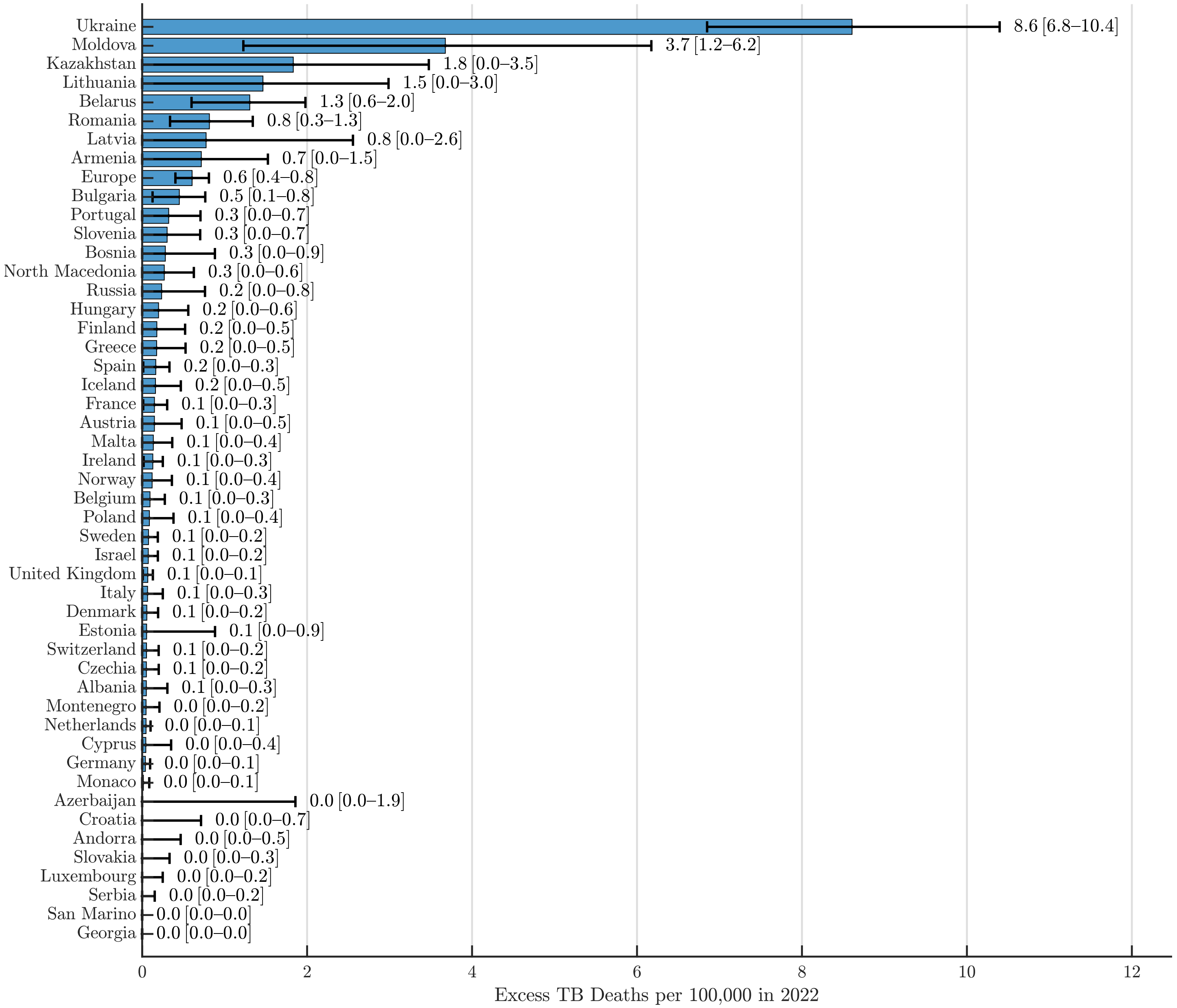


Figure S73: Excess TB mortality rate per 100,000 in Europe in 2022, estimated using an ensemble framework with two sub-epidemics. The horizontal bar charts display the median excess mortality rate for each country, with horizontal lines extending from each bar representing the corresponding confidence intervals (lower and upper bounds). Exact mortality rate numbers are also provided for each country.

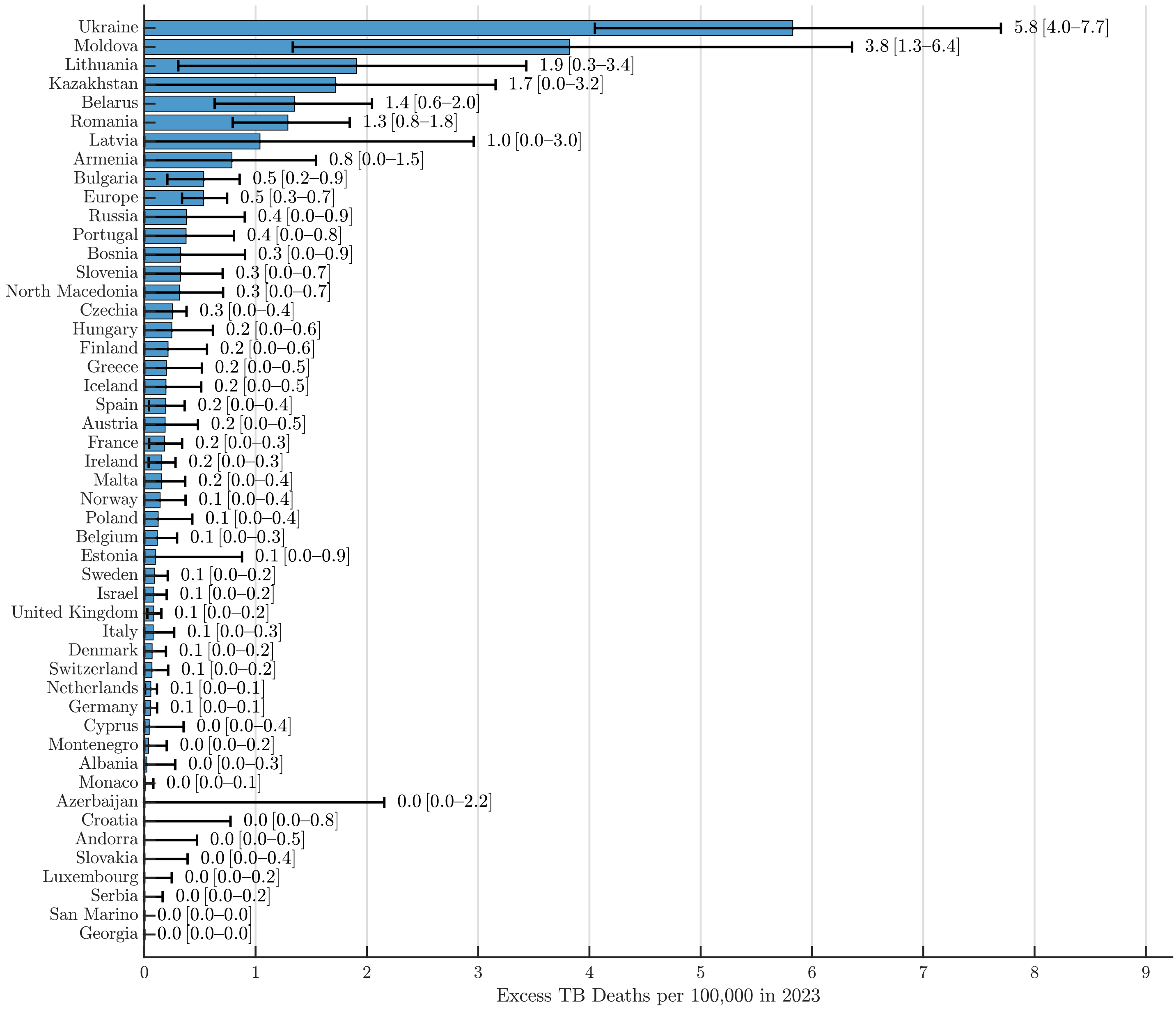


Figure S74: Excess TB mortality rate per 100,000 in Europe in 2023, estimated using an ensemble framework with two sub-epidemics. The horizontal bar charts display the median excess mortality rate for each country, with horizontal lines extending from each bar representing the corresponding confidence intervals (lower and upper bounds). Exact mortality rate numbers are also provided for each country.

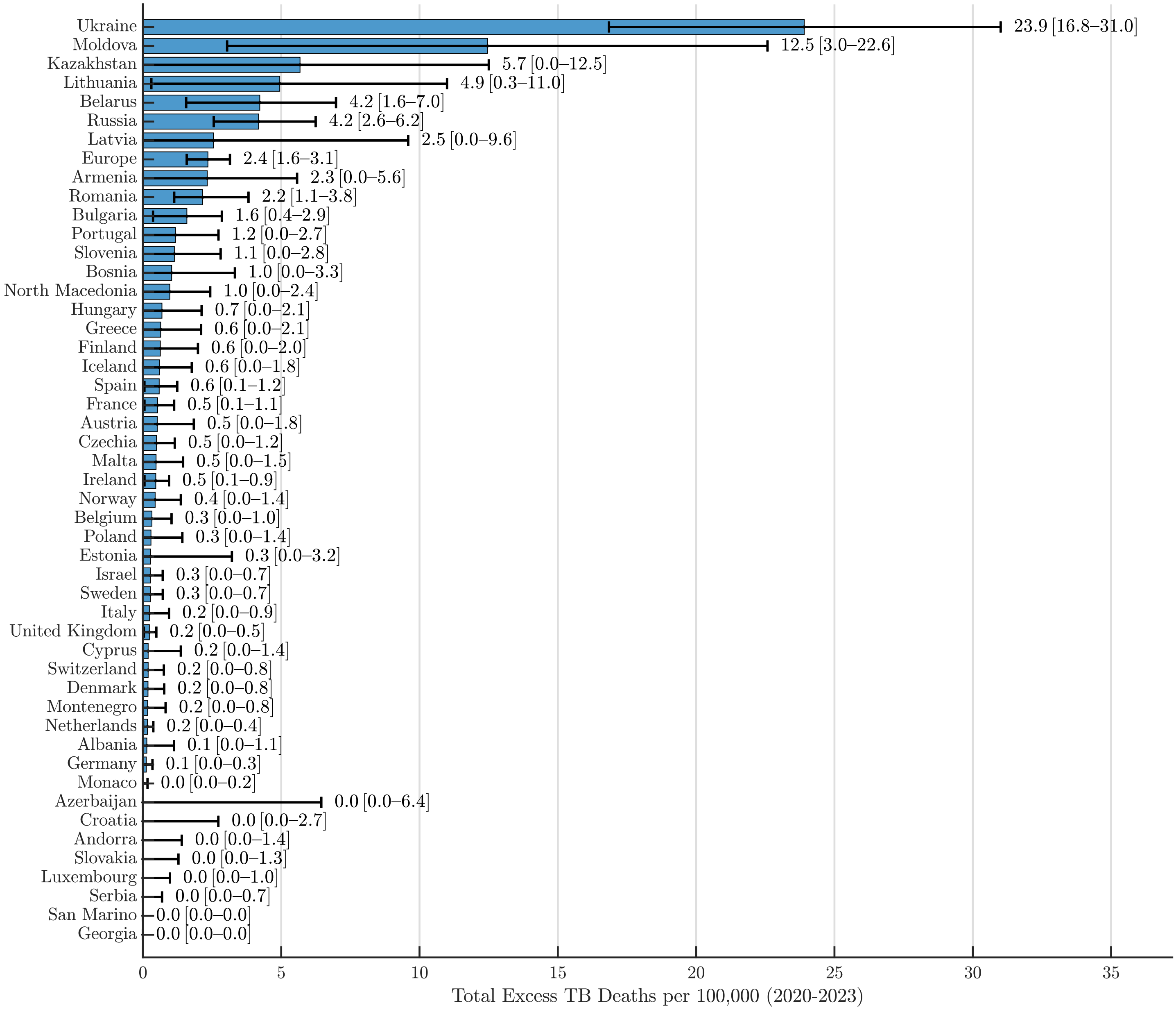


Figure S75: Total excess TB mortality rate per 100,000 in Europe in 2020-2023, estimated using an ensemble framework with two sub-epidemics. The horizontal bar charts display the median excess mortality rate for each country, with horizontal lines extending from each bar representing the corresponding confidence intervals (lower and upper bounds). Exact mortality rate numbers are also provided for each country.

| | Excess TB mortality rate (LB,UB) | | | | |
|---|---|---|---|---|---|
| Country | **2020** | **2021** | **2022** | **2023** | **Total** |
| **Albania** | 0.0 (0.0, 0.3) | 0.1 (0.0, 0.3) | 0.1 (0.0, 0.3) | 0.0 (0.0, 0.3) | 0.1 (0.0, 1.1) |
| **Andorra** | 0.0 (0.0, 0.2) | 0.0 (0.0, 0.2) | 0.0 (0.0, 0.5) | 0.0 (0.0, 0.5) | 0.0 (0.0, 1.4) |
| **Armenia** | 0.3 (0.0, 1.1) | 0.5 (0.0, 1.4) | 0.7 (0.0, 1.5) | 0.8 (0.0, 1.5) | 2.3 (0.0, 5.6) |
| **Austria** | 0.1 (0.0, 0.4) | 0.1 (0.0, 0.5) | 0.1 (0.0, 0.5) | 0.2 (0.0, 0.5) | 0.5 (0.0, 1.8) |
| **Azerbaijan** | 0.0 (0.0, 1.0) | 0.0 (0.0, 1.5) | 0.0 (0.0, 1.9) | 0.0 (0.0, 2.2) | 0.0 (0.0, 6.4) |
| **Belarus** | 0.5 (0.0, 1.2) | 1.0 (0.3, 1.7) | 1.3 (0.6, 2.0) | 1.4 (0.6, 2.0) | 4.2 (1.6, 7.0) |
| **Belgium** | 0.0 (0.0, 0.2) | 0.1 (0.0, 0.3) | 0.1 (0.0, 0.3) | 0.1 (0.0, 0.3) | 0.3 (0.0, 1.0) |
| **Bosnia** | 0.2 (0.0, 0.7) | 0.2 (0.0, 0.8) | 0.3 (0.0, 0.9) | 0.3 (0.0, 0.9) | 1.0 (0.0, 3.3) |
| **Bulgaria** | 0.2 (0.0, 0.6) | 0.4 (0.0, 0.7) | 0.5 (0.1, 0.8) | 0.5 (0.2, 0.9) | 1.6 (0.4, 2.9) |
| **Croatia** | 0.0 (0.0, 0.6) | 0.0 (0.0, 0.6) | 0.0 (0.0, 0.7) | 0.0 (0.0, 0.8) | 0.0 (0.0, 2.7) |
| **Cyprus** | 0.1 (0.0, 0.3) | 0.1 (0.0, 0.3) | 0.0 (0.0, 0.4) | 0.0 (0.0, 0.4) | 0.2 (0.0, 1.4) |
| **Czechia** | 0.0 (0.0, 0.2) | 0.2 (0.0, 0.3) | 0.1 (0.0, 0.2) | 0.3 (0.0, 0.4) | 0.5 (0.0, 1.2) |
| **Denmark** | 0.0 (0.0, 0.2) | 0.0 (0.0, 0.2) | 0.1 (0.0, 0.2) | 0.1 (0.0, 0.2) | 0.2 (0.0, 0.8) |
| **Estonia** | 0.0 (0.0, 0.5) | 0.1 (0.0, 1.0) | 0.1 (0.0, 0.9) | 0.1 (0.0, 0.9) | 0.3 (0.0, 3.2) |
| **Finland** | 0.1 (0.0, 0.4) | 0.1 (0.0, 0.5) | 0.2 (0.0, 0.5) | 0.2 (0.0, 0.6) | 0.6 (0.0, 2.0) |
| **France** | 0.1 (0.0, 0.2) | 0.1 (0.0, 0.3) | 0.1 (0.0, 0.3) | 0.2 (0.0, 0.3) | 0.5 (0.1, 1.1) |
| **Georgia** | 0.0 (0.0, 0.0) | 0.0 (0.0, 0.0) | 0.0 (0.0, 0.0) | 0.0 (0.0, 0.0) | 0.0 (0.0, 0.0) |
| **Germany** | 0.0 (0.0, 0.1) | 0.0 (0.0, 0.1) | 0.0 (0.0, 0.1) | 0.1 (0.0, 0.1) | 0.1 (0.0, 0.3) |
| **Greece** | 0.1 (0.0, 0.5) | 0.2 (0.0, 0.5) | 0.2 (0.0, 0.5) | 0.2 (0.0, 0.5) | 0.6 (0.0, 2.1) |
| **Hungary** | 0.1 (0.0, 0.4) | 0.1 (0.0, 0.5) | 0.2 (0.0, 0.6) | 0.2 (0.0, 0.6) | 0.7 (0.0, 2.1) |
| **Iceland** | 0.1 (0.0, 0.4) | 0.1 (0.0, 0.4) | 0.2 (0.0, 0.5) | 0.2 (0.0, 0.5) | 0.6 (0.0, 1.8) |
| **Ireland** | 0.1 (0.0, 0.2) | 0.1 (0.0, 0.2) | 0.1 (0.0, 0.3) | 0.2 (0.0, 0.3) | 0.5 (0.1, 0.9) |
| **Israel** | 0.0 (0.0, 0.2) | 0.1 (0.0, 0.2) | 0.1 (0.0, 0.2) | 0.1 (0.0, 0.2) | 0.3 (0.0, 0.7) |
| **Italy** | 0.0 (0.0, 0.2) | 0.1 (0.0, 0.2) | 0.1 (0.0, 0.3) | 0.1 (0.0, 0.3) | 0.2 (0.0, 0.9) |
| **Kazakhstan** | 0.9 (0.0, 2.7) | 1.3 (0.0, 3.2) | 1.8 (0.0, 3.5) | 1.7 (0.0, 3.2) | 5.7 (0.0, 12.5) |
| **Latvia** | 0.2 (0.0, 1.9) | 0.5 (0.0, 2.2) | 0.8 (0.0, 2.6) | 1.0 (0.0, 3.0) | 2.5 (0.0, 9.6) |
| **Lithuania** | 0.6 (0.0, 2.0) | 1.0 (0.0, 2.5) | 1.5 (0.0, 3.0) | 1.9 (0.3, 3.4) | 4.9 (0.3, 11.0) |
| **Luxembourg** | 0.0 (0.0, 0.2) | 0.0 (0.0, 0.2) | 0.0 (0.0, 0.2) | 0.0 (0.0, 0.2) | 0.0 (0.0, 1.0) |
| **Malta** | 0.1 (0.0, 0.4) | 0.1 (0.0, 0.4) | 0.1 (0.0, 0.4) | 0.2 (0.0, 0.4) | 0.5 (0.0, 1.5) |
| **Monaco** | 0.0 (0.0, 0.0) | 0.0 (0.0, 0.0) | 0.0 (0.0, 0.1) | 0.0 (0.0, 0.1) | 0.0 (0.0, 0.2) |
| **Montenegro** | 0.0 (0.0, 0.2) | 0.0 (0.0, 0.2) | 0.0 (0.0, 0.2) | 0.0 (0.0, 0.2) | 0.2 (0.0, 0.8) |
| **Netherlands** | 0.0 (0.0, 0.1) | 0.0 (0.0, 0.1) | 0.0 (0.0, 0.1) | 0.1 (0.0, 0.1) | 0.2 (0.0, 0.4) |
| **North Macedonia** | 0.2 (0.0, 0.5) | 0.2 (0.0, 0.6) | 0.3 (0.0, 0.6) | 0.3 (0.0, 0.7) | 1.0 (0.0, 2.4) |
| **Norway** | 0.1 (0.0, 0.3) | 0.1 (0.0, 0.3) | 0.1 (0.0, 0.4) | 0.1 (0.0, 0.4) | 0.4 (0.0, 1.4) |
| **Poland** | 0.0 (0.0, 0.3) | 0.1 (0.0, 0.3) | 0.1 (0.0, 0.4) | 0.1 (0.0, 0.4) | 0.3 (0.0, 1.4) |
| **Portugal** | 0.2 (0.0, 0.6) | 0.3 (0.0, 0.6) | 0.3 (0.0, 0.7) | 0.4 (0.0, 0.8) | 1.2 (0.0, 2.7) |
| **Moldova** | 2.0 (0.0, 4.4) | 3.0 (0.5, 5.6) | 3.7 (1.2, 6.2) | 3.8 (1.3, 6.4) | 12.5 (3.0, 22.6) |
| **Romania** | 0.0 (0.0, 0.1) | 0.1 (0.0, 0.6) | 0.8 (0.3, 1.3) | 1.3 (0.8, 1.8) | 2.2 (1.1, 3.8) |
| **Russia** | 1.6 (1.1, 2.1) | 1.9 (1.4, 2.5) | 0.2 (0.0, 0.8) | 0.4 (0.0, 0.9) | 4.2 (2.6, 6.2) |
| **Serbia** | 0.0 (0.0, 0.2) | 0.0 (0.0, 0.2) | 0.0 (0.0, 0.2) | 0.0 (0.0, 0.2) | 0.0 (0.0, 0.7) |
| **Slovakia** | 0.0 (0.0, 0.3) | 0.0 (0.0, 0.3) | 0.0 (0.0, 0.3) | 0.0 (0.0, 0.4) | 0.0 (0.0, 1.3) |
| **Slovenia** | 0.2 (0.0, 0.7) | 0.3 (0.0, 0.7) | 0.3 (0.0, 0.7) | 0.3 (0.0, 0.7) | 1.1 (0.0, 2.8) |
| **Spain** | 0.1 (0.0, 0.3) | 0.1 (0.0, 0.3) | 0.2 (0.0, 0.3) | 0.2 (0.0, 0.4) | 0.6 (0.1, 1.2) |
| **Sweden** | 0.0 (0.0, 0.1) | 0.1 (0.0, 0.2) | 0.1 (0.0, 0.2) | 0.1 (0.0, 0.2) | 0.3 (0.0, 0.7) |
| **Switzerland** | 0.0 (0.0, 0.2) | 0.0 (0.0, 0.2) | 0.1 (0.0, 0.2) | 0.1 (0.0, 0.2) | 0.2 (0.0, 0.8) |
| **Ukraine** | 3.1 (1.4, 4.8) | 6.4 (4.6, 8.1) | 8.6 (6.8, 10.4) | 5.8 (4.0, 7.7) | 23.9 (16.8, 31.0) |
| **United Kingdom** | 0.0 (0.0, 0.1) | 0.0 (0.0, 0.1) | 0.1 (0.0, 0.1) | 0.1 (0.0, 0.2) | 0.2 (0.0, 0.5) |
| **Europe** | 0.5 (0.3, 0.6) | 0.8 (0.6, 0.9) | 0.6 (0.4, 0.8) | 0.5 (0.3, 0.7) | 2.4 (1.6, 3.1) |

Table S10: Estimated excess TB mortality rate in Europe for the years 2020 to 2023, presented both individually by year and as a total. The estimates include the median value along with the corresponding lower and upper bounds, calculated using an ensemble framework that models two distinct sub-epidemics.

### S5.3 SMR

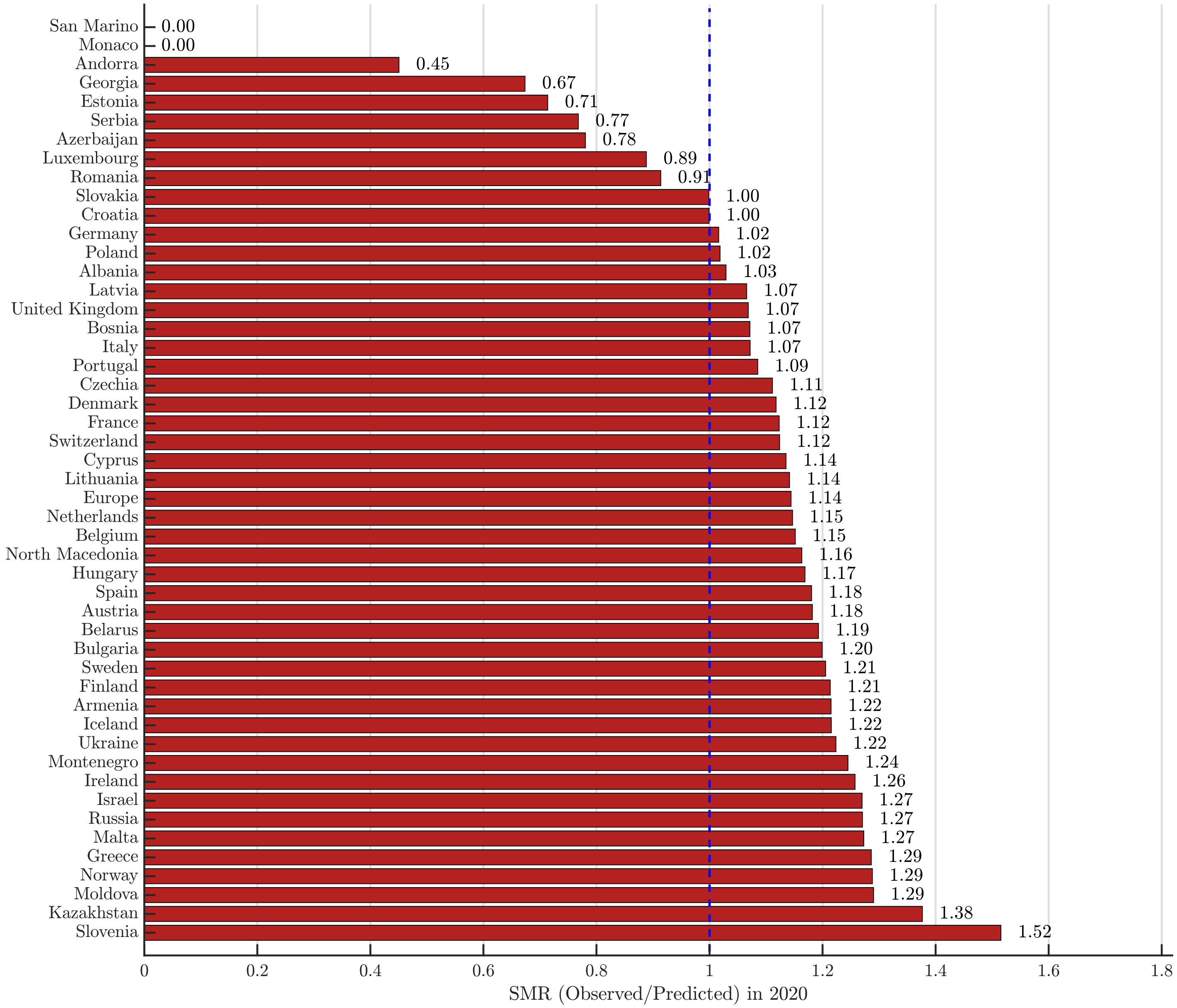


Figure S76: SMR in Europe in 2020, estimated using an ensemble framework with two sub-epidemics. SMR is displayed horizontally in bar charts, with the exact number displayed in front of the bars.

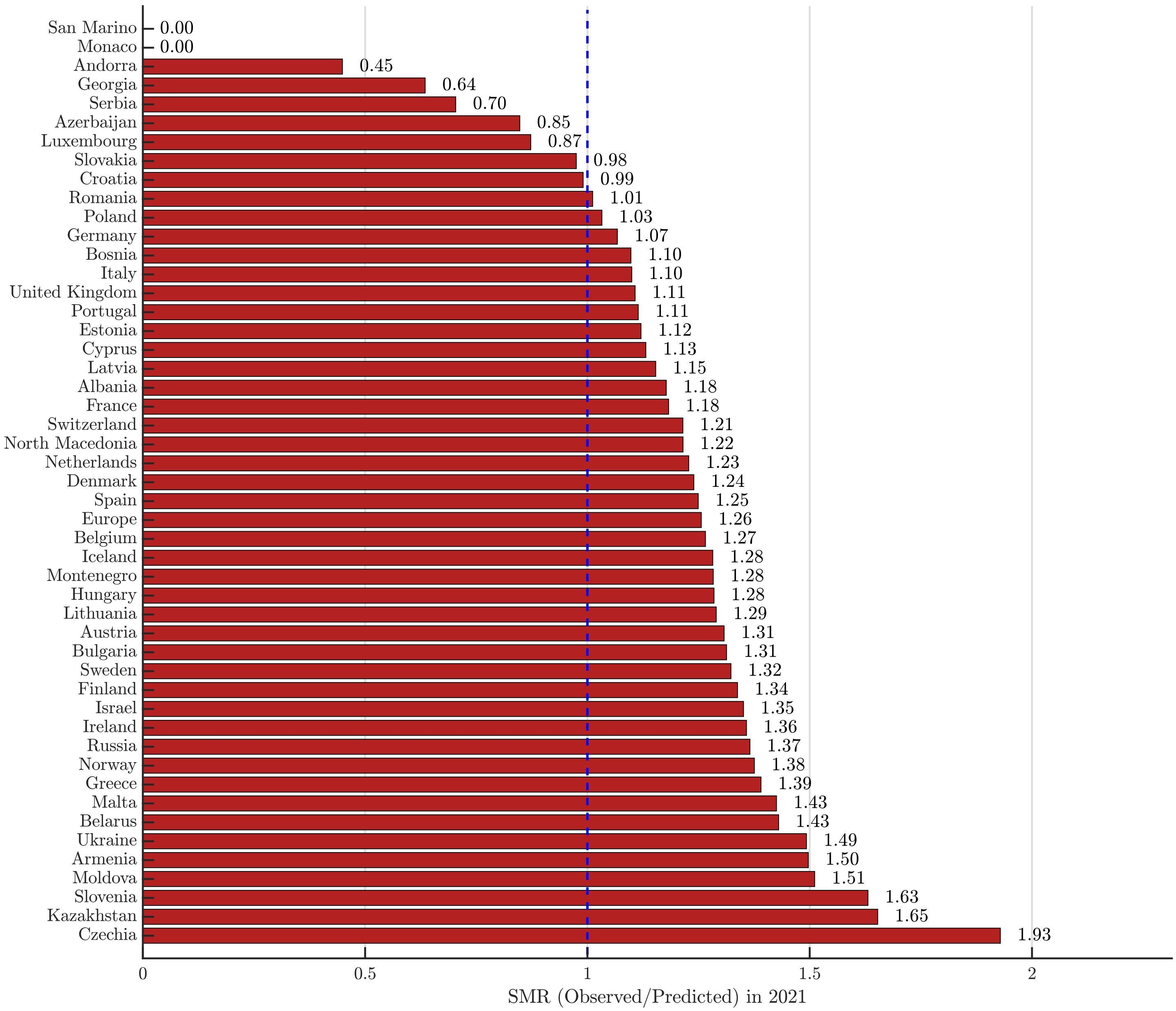


Figure S77: SMR in Europe in 2021, estimated using an ensemble framework with two sub-epidemics. SMR is displayed horizontally in bar charts, with the exact number displayed in front of the bars.

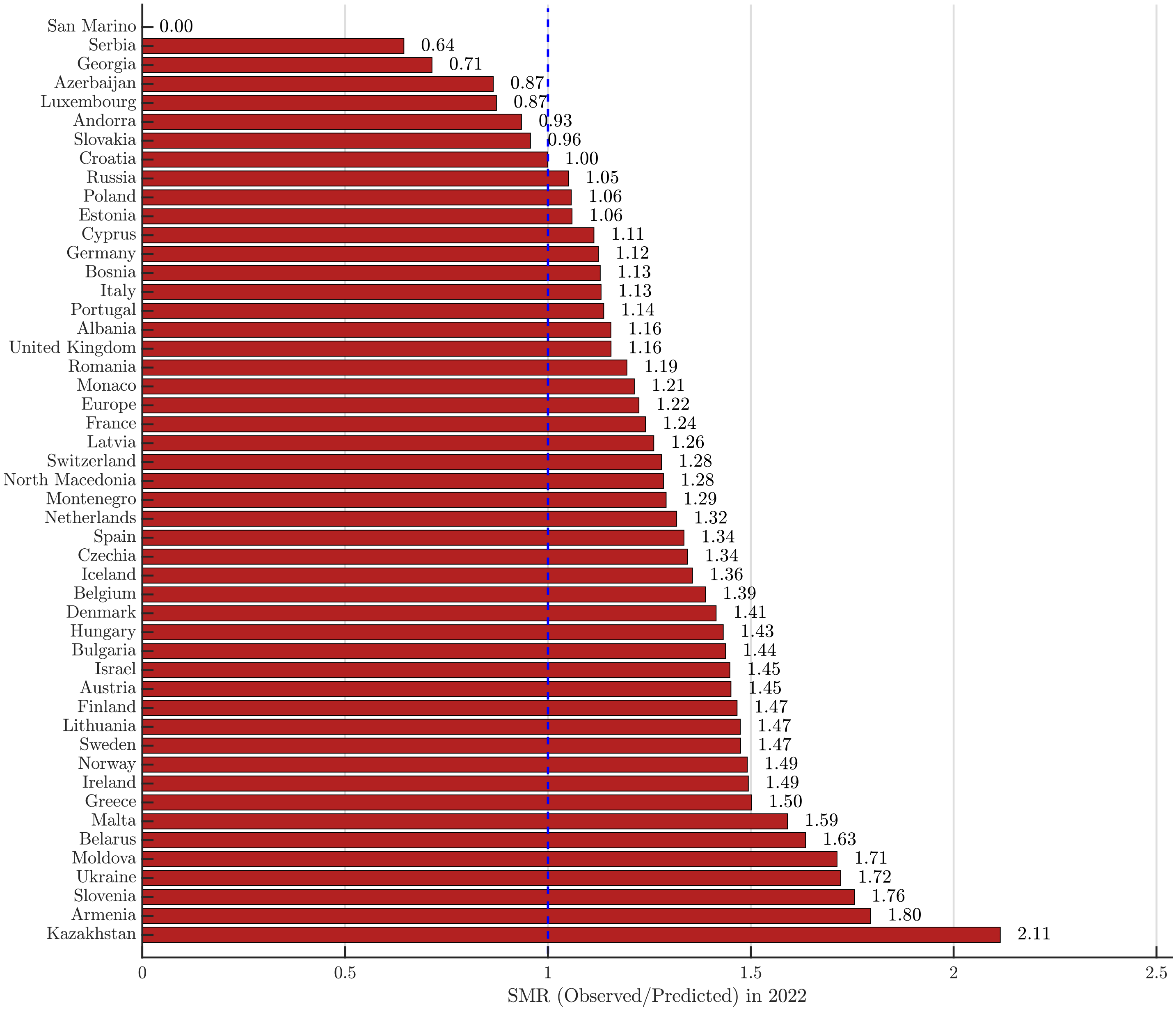


Figure S78: SMR in Europe in 2022, estimated using an ensemble framework with two sub-epidemics. SMR is displayed horizontally in bar charts, with the exact number displayed in front of the bars.

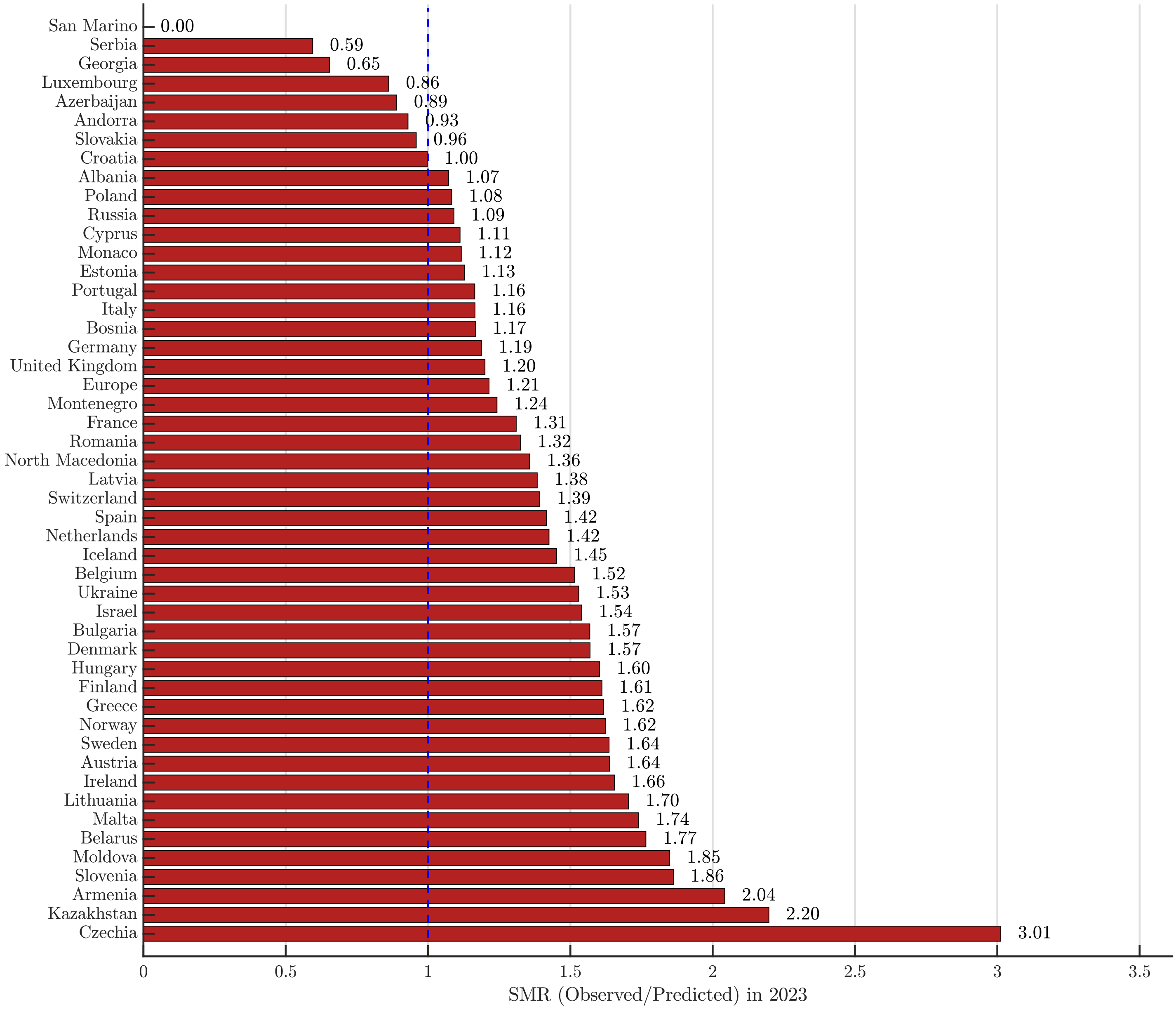


Figure S79: SMR in Europe in 2023, estimated using an ensemble framework with two sub-epidemics. SMR is displayed horizontally in bar charts, with the exact number displayed in front of the bars.

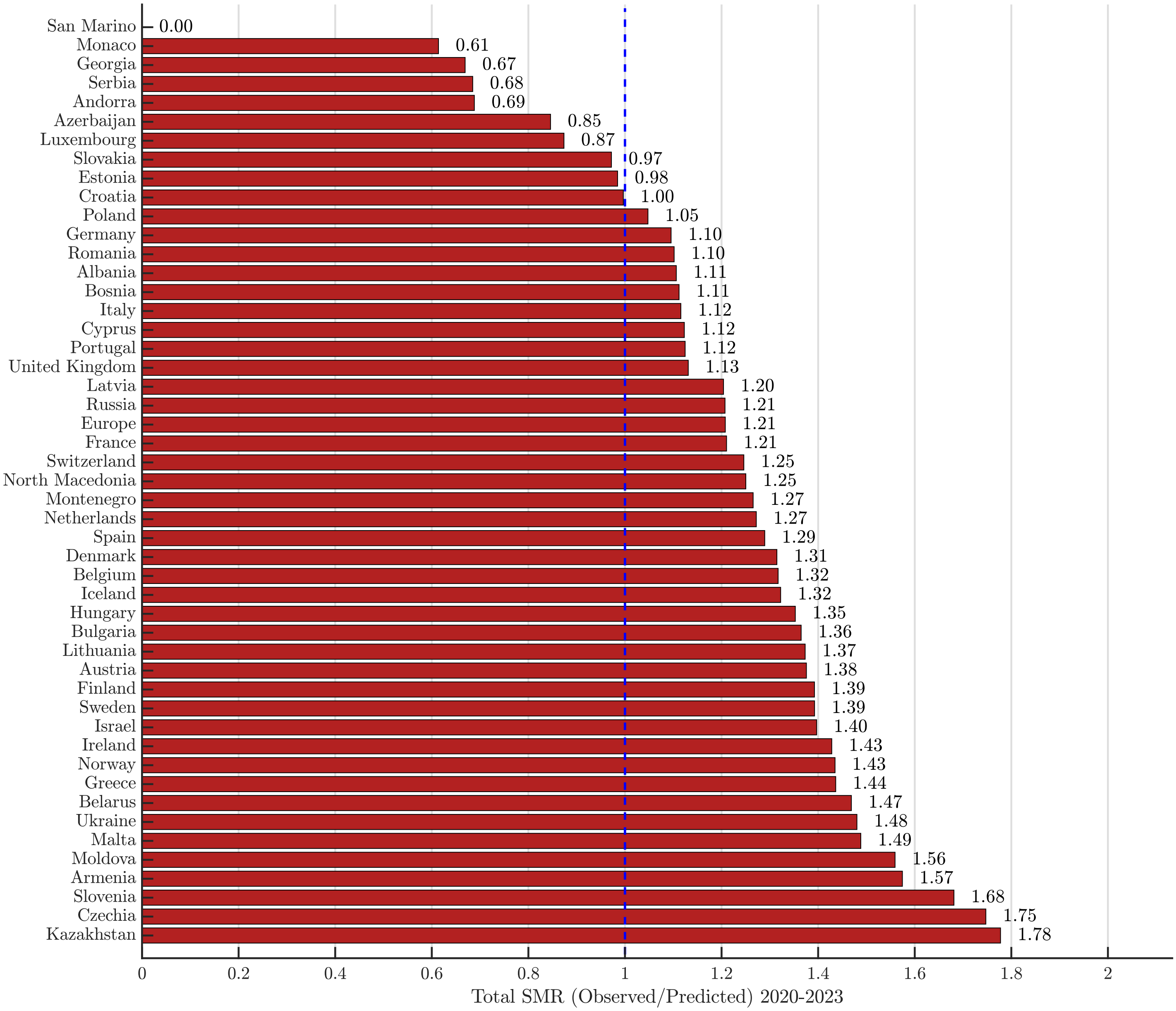


Figure S80: SMR in Europe during 2020-2023, estimated using an ensemble framework with two sub-epidemics. SMR is displayed horizontally in bar charts, with the exact number displayed in front of the bars.

## S6 Eastern Mediterranean

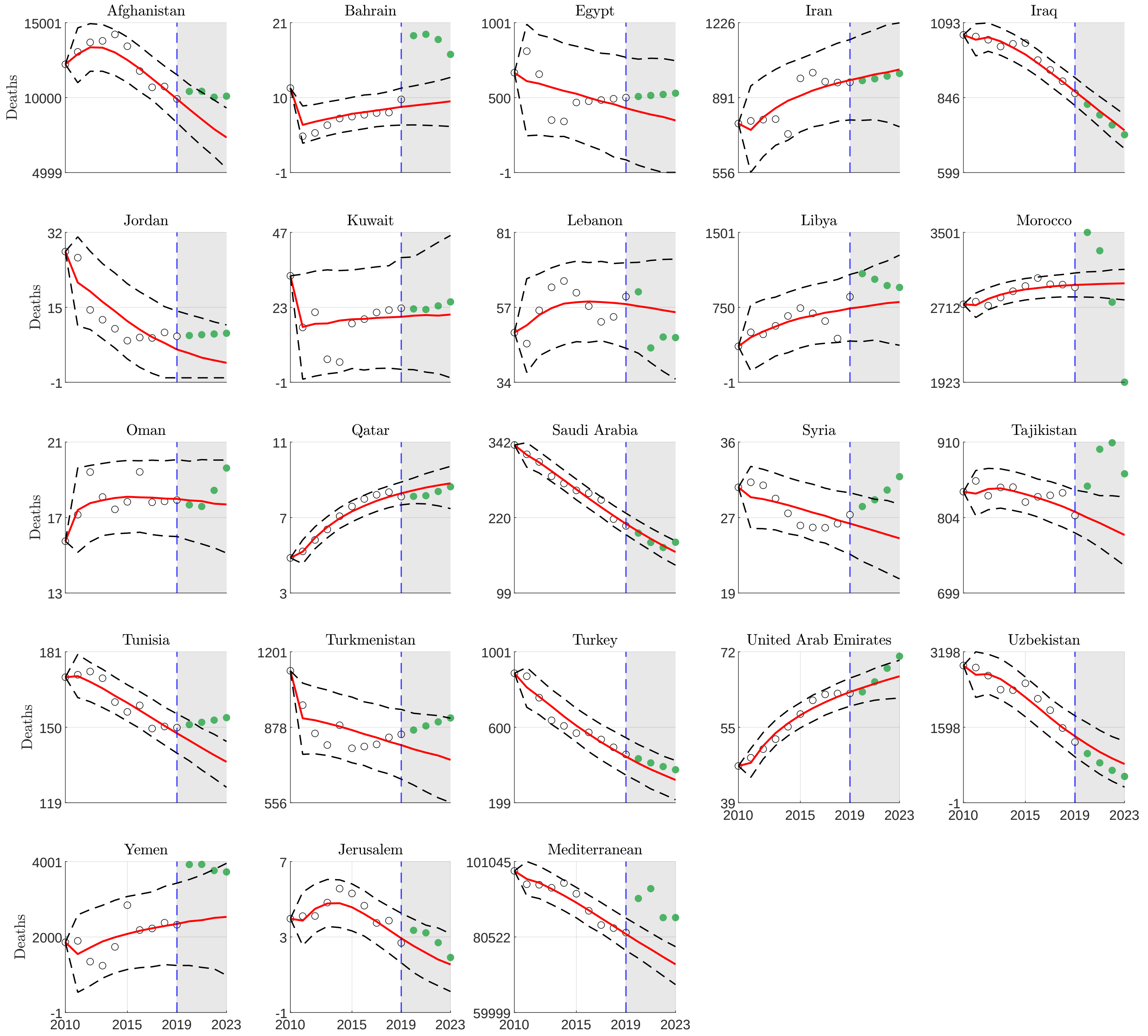


Figure S81: Forecasting panel for the number of deaths in the Eastern Mediterranean, with a calibration period spanning 10 years (2010–2019) and a four-year forecasting period (2020–2023), obtained using the Ranked 1 method. The red curve represents the median forecast, while the black dashed lines indicate the 95% PI. Circles represent reported data: black circles correspond to the calibration period, and green-filled circles correspond to actual data points during the forecast period.

## S6.1 Excess mortality

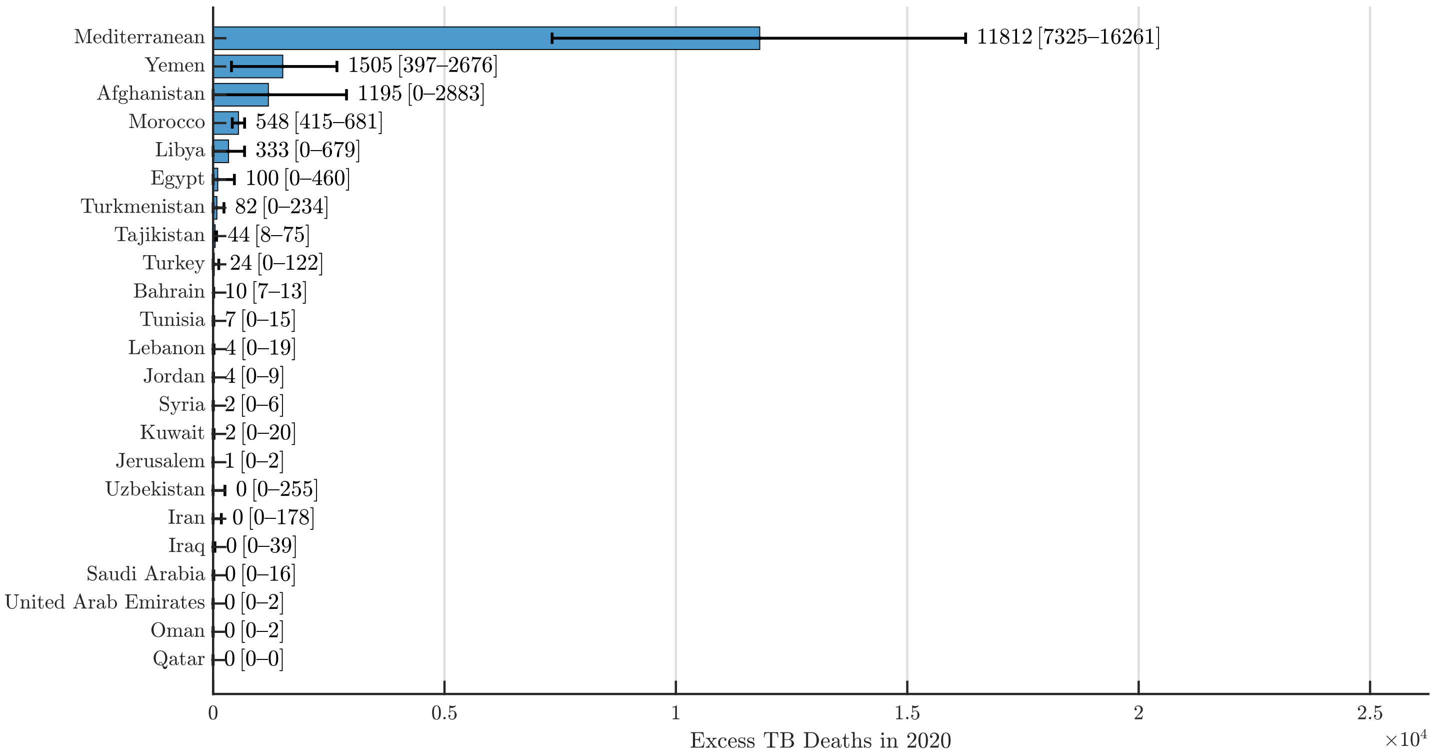


Figure S82: Excess TB mortality in the Eastern Mediterranean region in 2020, estimated using an ensemble framework with two sub-epidemics. The horizontal bar charts display the median excess mortality for each country, with horizontal lines extending from each bar representing the corresponding confidence intervals (lower and upper bounds). Exact mortality numbers are also provided for each country.

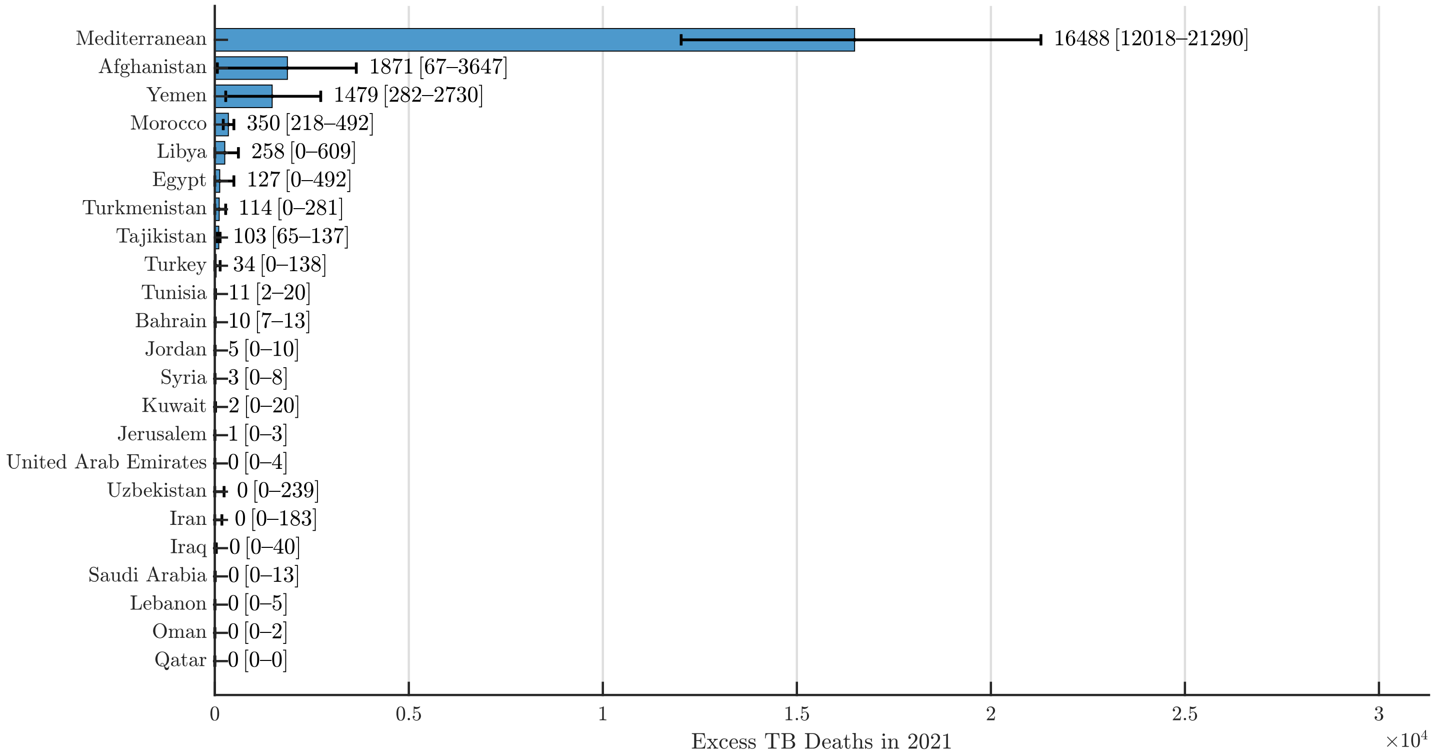


Figure S83: Excess TB mortality in the Eastern Mediterranean region in 2021, estimated using an ensemble framework with two sub-epidemics. The horizontal bar charts display the median excess mortality for each country, with horizontal lines extending from each bar representing the corresponding confidence intervals (lower and upper bounds). Exact mortality numbers are also provided for each country.

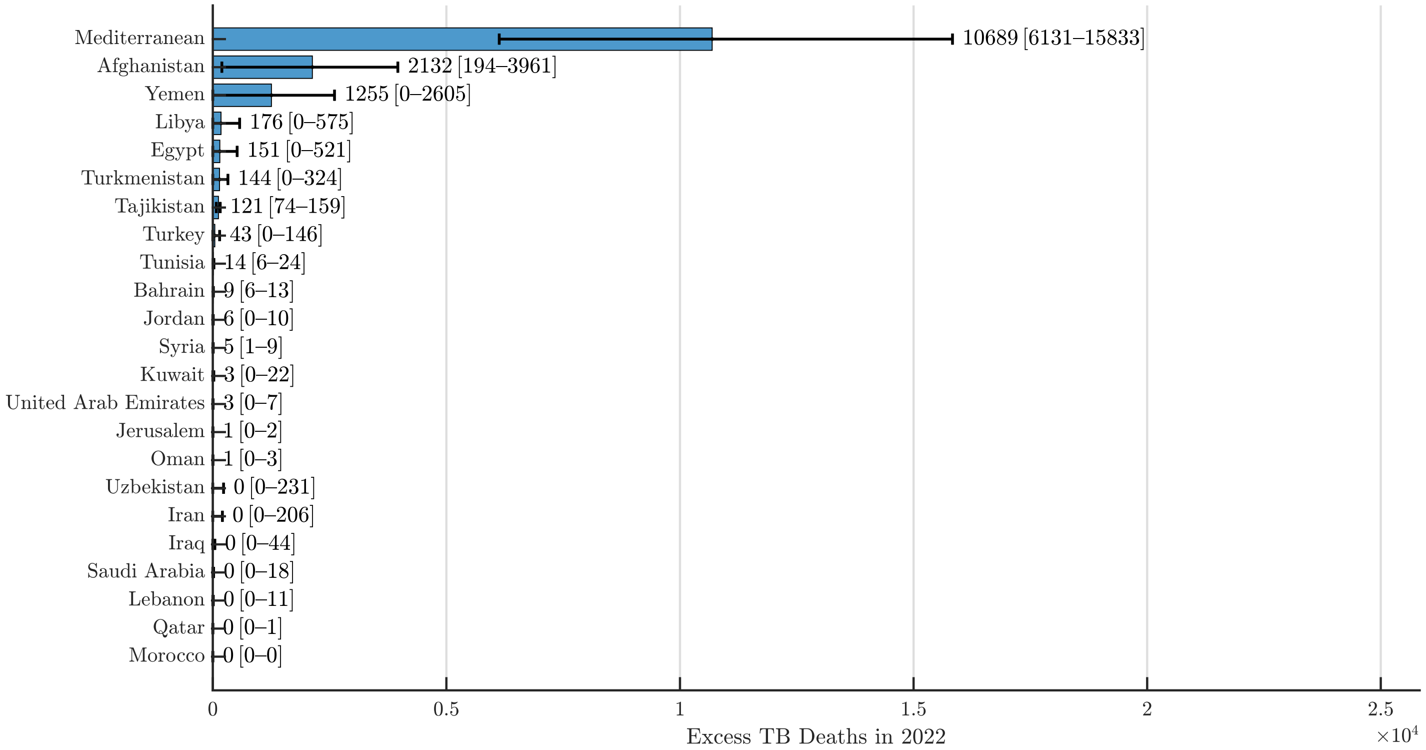


Figure S84: Excess TB mortality in the Eastern Mediterranean region in 2022, estimated using an ensemble framework with two sub-epidemics. The horizontal bar charts display the median excess mortality for each country, with horizontal lines extending from each bar representing the corresponding confidence intervals (lower and upper bounds). Exact mortality numbers are also provided for each country.

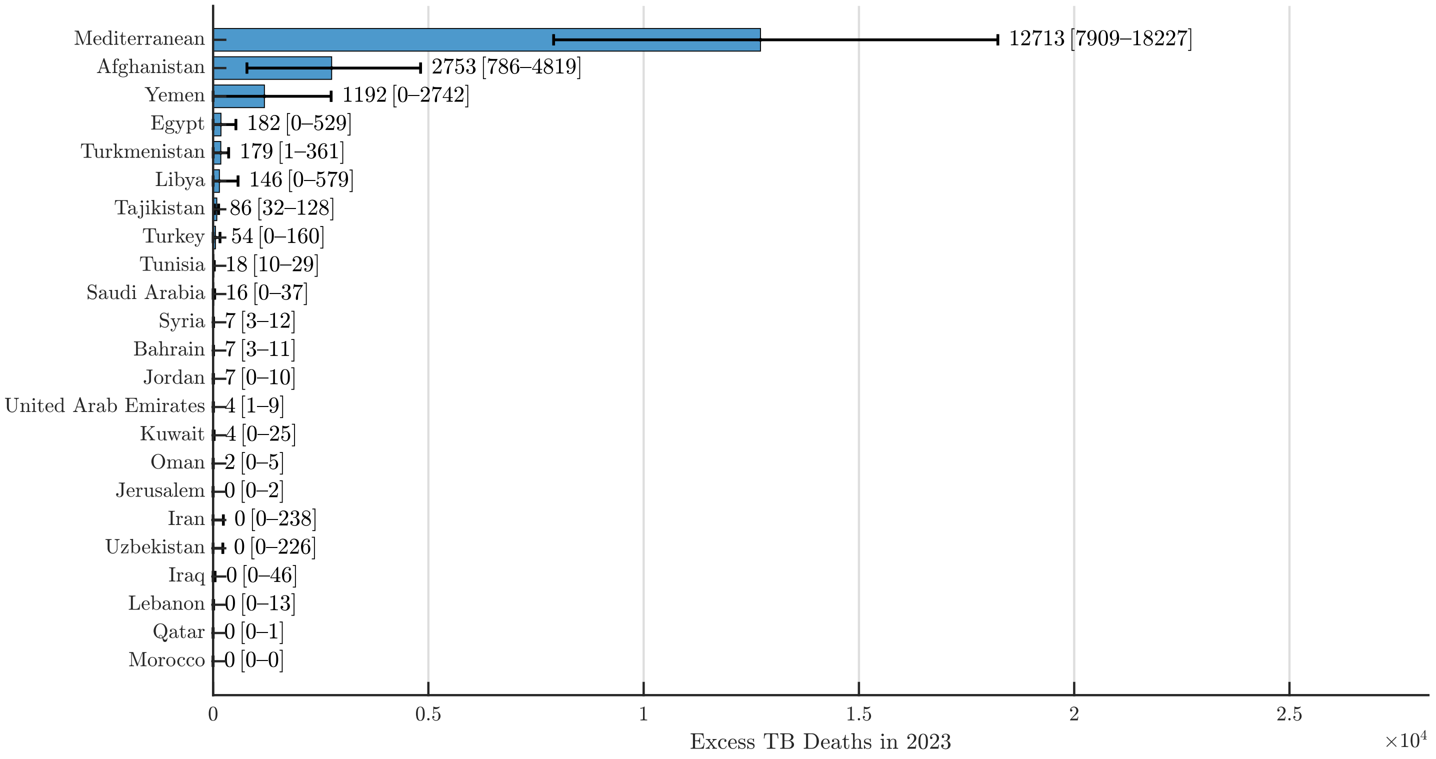


Figure S85: Excess TB mortality in the Eastern Mediterranean region in 2023, estimated using an ensemble framework with two sub-epidemics. The horizontal bar charts display the median excess mortality for each country, with horizontal lines extending from each bar representing the corresponding confidence intervals (lower and upper bounds). Exact mortality numbers are also provided for each country.

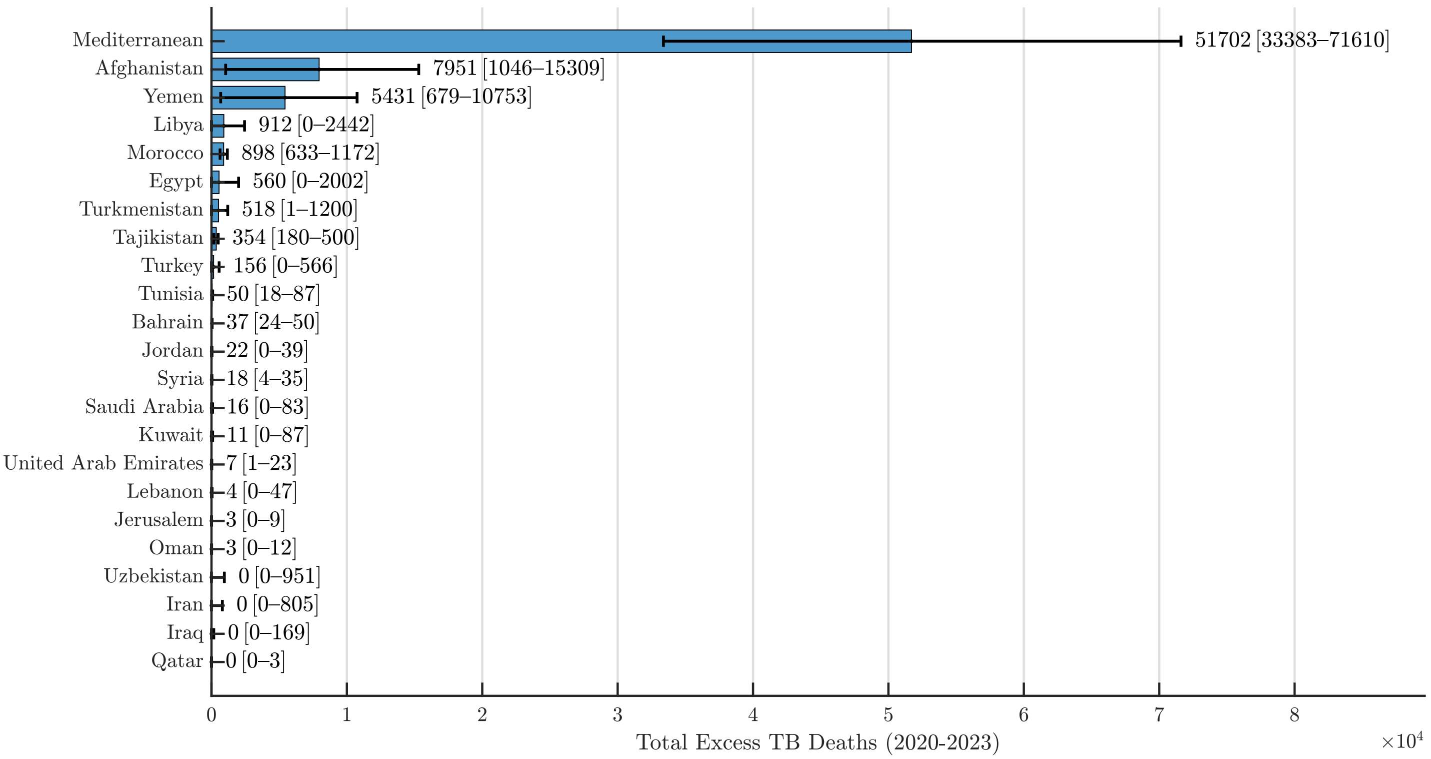


Figure S86: Total excess TB mortality in the Eastern Mediterranean region in 2020-2023, estimated using an ensemble framework with two sub-epidemics. The horizontal bar charts display the median excess mortality for each country, with horizontal lines extending from each bar representing the corresponding confidence intervals (lower and upper bounds). Exact mortality numbers are also provided for each country.

| | Excess TB mortality (LB,UB) | | | | |
|---|---|---|---|---|---|
| Country | **2020** | **2021** | **2022** | **2023** | **Total** |
| **Afghanistan** | 1195 (0, 2883) | 1871 (67, 3647) | 2132 (194, 3961) | 2753 (786, 4819) | 7951 (1046, 15309) |
| **Bahrain** | 10 (7, 13) | 10 (7, 13) | 9 (6, 13) | 7 (3, 11) | 37 (24, 50) |
| **Egypt** | 100 (0, 460) | 127 (0, 492) | 151 (0, 521) | 182 (0, 529) | 560 (0, 2002) |
| **Iran** | 0 (0, 178) | 0 (0, 183) | 0 (0, 206) | 0 (0, 238) | 0 (0, 805) |
| **Iraq** | 0 (0, 39) | 0 (0, 40) | 0 (0, 44) | 0 (0, 46) | 0 (0, 169) |
| **Jordan** | 4 (0, 9) | 5 (0, 10) | 6 (0, 10) | 7 (0, 10) | 22 (0, 39) |
| **Kuwait** | 2 (0, 20) | 2 (0, 20) | 3 (0, 22) | 4 (0, 25) | 11 (0, 87) |
| **Lebanon** | 4 (0, 19) | 0 (0, 5) | 0 (0, 11) | 0 (0, 13) | 4 (0, 47) |
| **Libya** | 333 (0, 679) | 258 (0, 609) | 176 (0, 575) | 146 (0, 579) | 912 (0, 2442) |
| **Morocco** | 548 (415, 681) | 350 (218, 492) | 0 (0, 0) | 0 (0, 0) | 898 (633, 1172) |
| **Oman** | 0 (0, 2) | 0 (0, 2) | 1 (0, 3) | 2 (0, 5) | 3 (0, 12) |
| **Qatar** | 0 (0, 0) | 0 (0, 0) | 0 (0, 1) | 0 (0, 1) | 0 (0, 3) |
| **Saudi Arabia** | 0 (0, 16) | 0 (0, 13) | 0 (0, 18) | 16 (0, 37) | 16 (0, 83) |
| **Syria** | 2 (0, 6) | 3 (0, 8) | 5 (1, 9) | 7 (3, 12) | 18 (4, 35) |
| **Tajikistan** | 44 (8, 75) | 103 (65, 137) | 121 (74, 159) | 86 (32, 128) | 354 (180, 500) |
| **Tunisia** | 7 (0, 15) | 11 (2, 20) | 14 (6, 24) | 18 (10, 29) | 50 (18, 87) |
| **Turkmenistan** | 82 (0, 234) | 114 (0, 281) | 144 (0, 324) | 179 (1, 361) | 518 (1, 1200) |
| **Turkey** | 24 (0, 122) | 34 (0, 138) | 43 (0, 146) | 54 (0, 160) | 156 (0, 566) |
| **United Arab Emirates** | 0 (0, 2) | 0 (0, 4) | 3 (0, 7) | 4 (1, 9) | 7 (1, 23) |
| **Uzbekistan** | 0 (0, 255) | 0 (0, 239) | 0 (0, 231) | 0 (0, 226) | 0 (0, 951) |
| **Yemen** | 1505 (397, 2676) | 1479 (282, 2730) | 1255 (0, 2605) | 1192 (0, 2742) | 5431 (679, 10753) |
| **Jerusalem** | 1 (0, 2) | 1 (0, 3) | 1 (0, 2) | 0 (0, 2) | 3 (0, 9) |
| **Mediterranean** | 11812 (7325, 16261) | 16488 (12018, 21290) | 10689 (6131, 15833) | 12713 (7909, 18227) | 51702 (33383, 71610) |

Table S11: Estimated excess TB mortality in the Eastern Mediterranean region for the years 2020 to 2023, presented both individually by year and as a total. The estimates include the median value along with the corresponding lower and upper bounds, calculated using an ensemble framework that models two distinct sub-epidemics.

## S6.2 Excess mortality rate

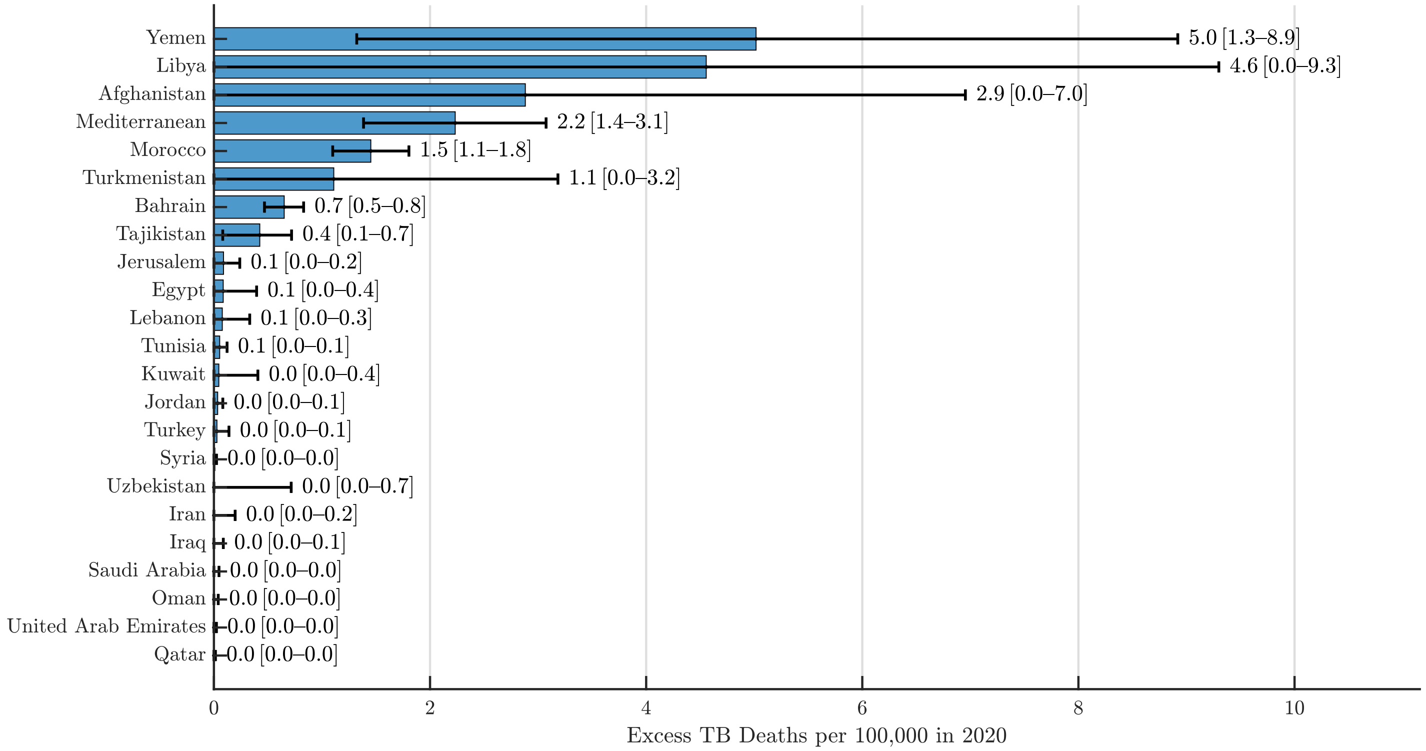


Figure S87: Excess TB mortality rate per 100,000 in the Eastern Mediterranean region in 2020, estimated using an ensemble framework with two sub-epidemics. The horizontal bar charts display the median excess mortality rate for each country, with horizontal lines extending from each bar representing the corresponding confidence intervals (lower and upper bounds). Exact mortality rate numbers are also provided for each country.

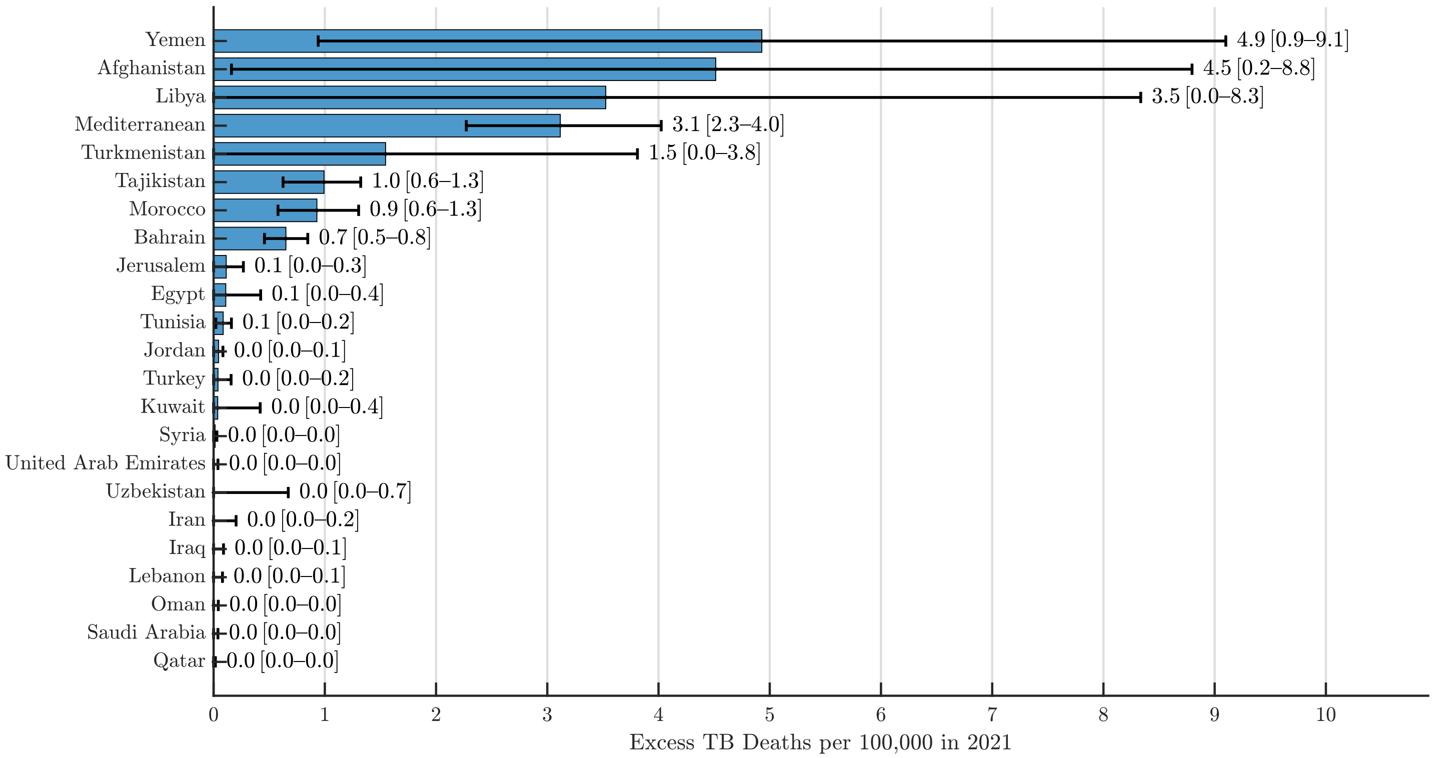


Figure S88: Excess TB mortality rate per 100,000 in the Eastern Mediterranean region in 2021, estimated using an ensemble framework with two sub-epidemics. The horizontal bar charts display the median excess mortality rate for each country, with horizontal lines extending from each bar representing the corresponding confidence intervals (lower and upper bounds). Exact mortality rate numbers are also provided for each country.

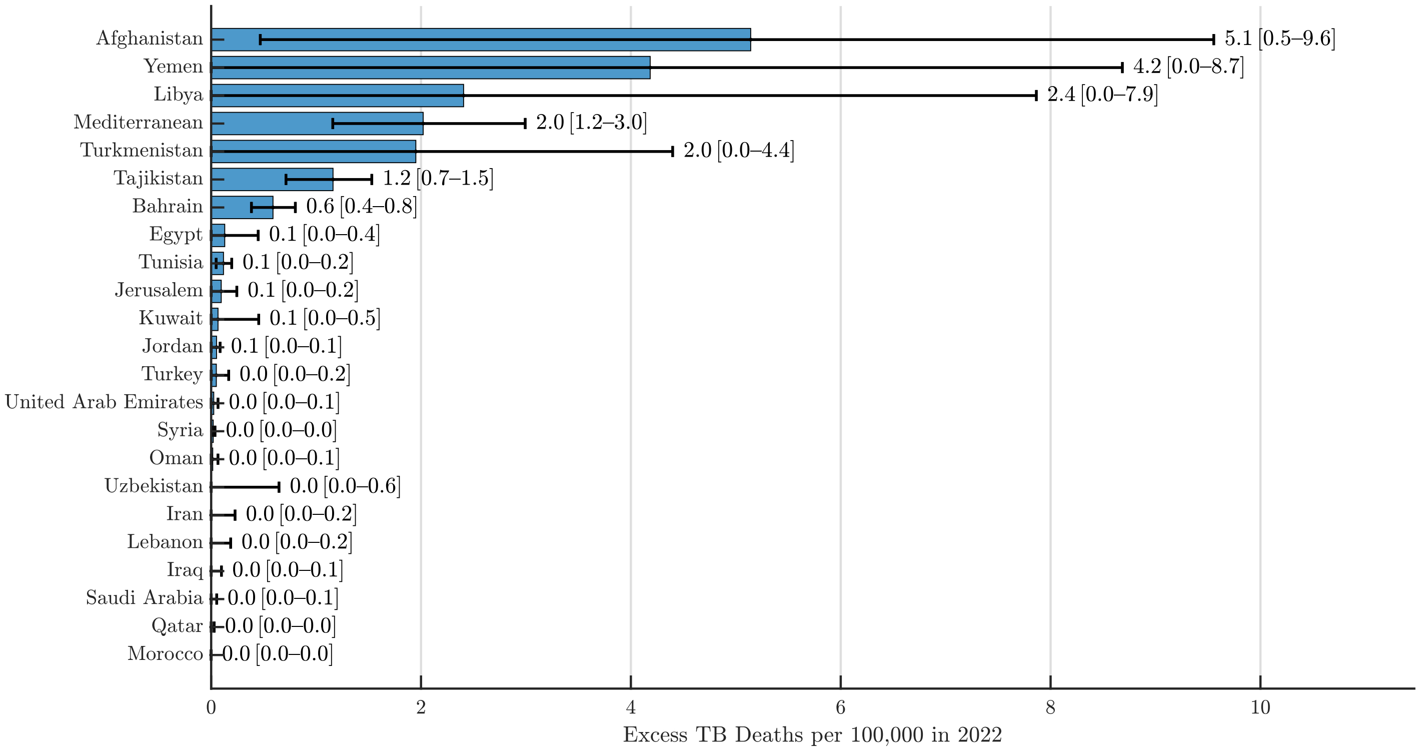


Figure S89: Excess TB mortality rate per 100,000 in the Eastern Mediterranean region in 2022, estimated using an ensemble framework with two sub-epidemics. The horizontal bar charts display the median excess mortality rate for each country, with horizontal lines extending from each bar representing the corresponding confidence intervals (lower and upper bounds). Exact mortality rate numbers are also provided for each country.

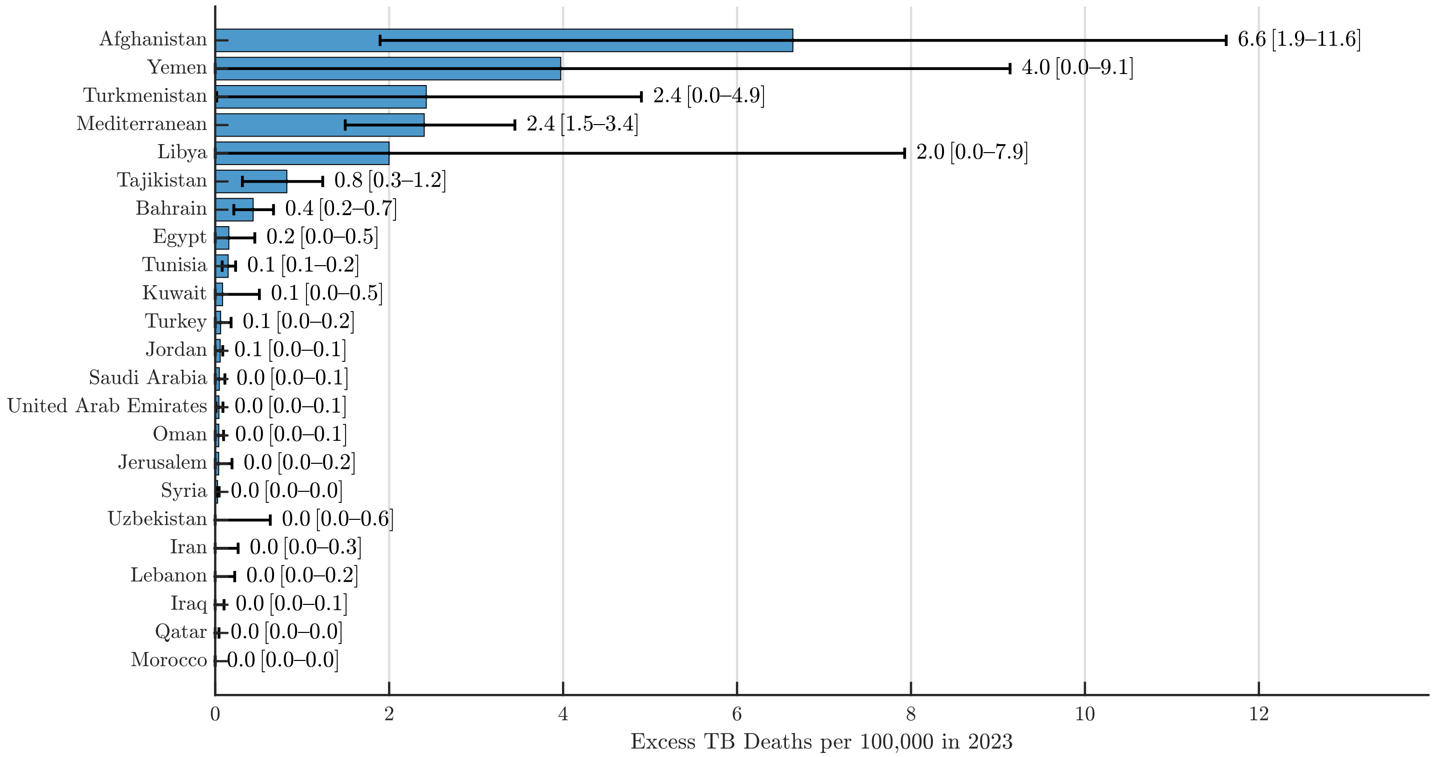


Figure S90: Excess TB mortality rate per 100,000 in the Eastern Mediterranean region in 2023, estimated using an ensemble framework with two sub-epidemics. The horizontal bar charts display the median excess mortality rate for each country, with horizontal lines extending from each bar representing the corresponding confidence intervals (lower and upper bounds). Exact mortality rate numbers are also provided for each country.

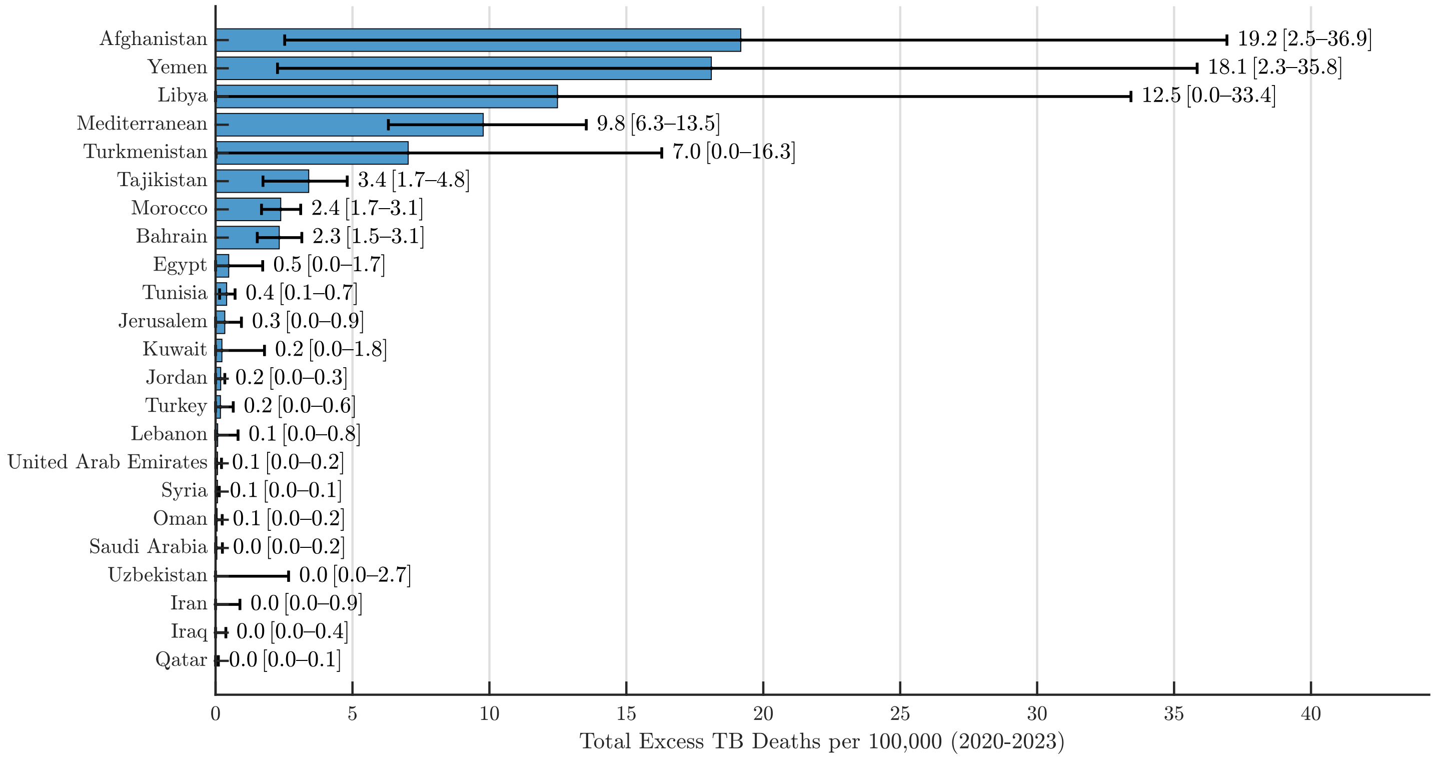


Figure S91: Total excess TB mortality rate per 100,000 in the Eastern Mediterranean region in 2020-2023, estimated using an ensemble framework with two sub-epidemics. The horizontal bar charts display the median excess mortality rate for each country, with horizontal lines extending from each bar representing the corresponding confidence intervals (lower and upper bounds). Exact mortality rate numbers are also provided for each country.

| | Excess TB mortality rate (LB,UB) | | | | |
|---|---|---|---|---|---|
| Country | **2020** | **2021** | **2022** | **2023** | **Total** |
| **Afghanistan** | 2.9 (0.0, 7.0) | 4.5 (0.2, 8.8) | 5.1 (0.5, 9.6) | 6.6 (1.9, 11.6) | 19.2 (2.5, 36.9) |
| **Bahrain** | 0.7 (0.5, 0.8) | 0.7 (0.5, 0.8) | 0.6 (0.4, 0.8) | 0.4 (0.2, 0.7) | 2.3 (1.5, 3.1) |
| **Egypt** | 0.1 (0.0, 0.4) | 0.1 (0.0, 0.4) | 0.1 (0.0, 0.4) | 0.2 (0.0, 0.5) | 0.5 (0.0, 1.7) |
| **Iran** | 0.0 (0.0, 0.2) | 0.0 (0.0, 0.2) | 0.0 (0.0, 0.2) | 0.0 (0.0, 0.3) | 0.0 (0.0, 0.9) |
| **Iraq** | 0.0 (0.0, 0.1) | 0.0 (0.0, 0.1) | 0.0 (0.0, 0.1) | 0.0 (0.0, 0.1) | 0.0 (0.0, 0.4) |
| **Jordan** | 0.0 (0.0, 0.1) | 0.0 (0.0, 0.1) | 0.1 (0.0, 0.1) | 0.1 (0.0, 0.1) | 0.2 (0.0, 0.3) |
| **Kuwait** | 0.0 (0.0, 0.4) | 0.0 (0.0, 0.4) | 0.1 (0.0, 0.5) | 0.1 (0.0, 0.5) | 0.2 (0.0, 1.8) |
| **Lebanon** | 0.1 (0.0, 0.3) | 0.0 (0.0, 0.1) | 0.0 (0.0, 0.2) | 0.0 (0.0, 0.2) | 0.1 (0.0, 0.8) |
| **Libya** | 4.6 (0.0, 9.3) | 3.5 (0.0, 8.3) | 2.4 (0.0, 7.9) | 2.0 (0.0, 7.9) | 12.5 (0.0, 33.4) |
| **Morocco** | 1.5 (1.1, 1.8) | 0.9 (0.6, 1.3) | 0.0 (0.0, 0.0) | 0.0 (0.0, 0.0) | 2.4 (1.7, 3.1) |
| **Oman** | 0.0 (0.0, 0.0) | 0.0 (0.0, 0.0) | 0.0 (0.0, 0.1) | 0.0 (0.0, 0.1) | 0.1 (0.0, 0.2) |
| **Qatar** | 0.0 (0.0, 0.0) | 0.0 (0.0, 0.0) | 0.0 (0.0, 0.0) | 0.0 (0.0, 0.0) | 0.0 (0.0, 0.1) |
| **Saudi Arabia** | 0.0 (0.0, 0.0) | 0.0 (0.0, 0.0) | 0.0 (0.0, 0.1) | 0.0 (0.0, 0.1) | 0.0 (0.0, 0.2) |
| **Syria** | 0.0 (0.0, 0.0) | 0.0 (0.0, 0.0) | 0.0 (0.0, 0.0) | 0.0 (0.0, 0.0) | 0.1 (0.0, 0.1) |
| **Tajikistan** | 0.4 (0.1, 0.7) | 1.0 (0.6, 1.3) | 1.2 (0.7, 1.5) | 0.8 (0.3, 1.2) | 3.4 (1.7, 4.8) |
| **Tunisia** | 0.1 (0.0, 0.1) | 0.1 (0.0, 0.2) | 0.1 (0.0, 0.2) | 0.1 (0.1, 0.2) | 0.4 (0.1, 0.7) |
| **Turkmenistan** | 1.1 (0.0, 3.2) | 1.5 (0.0, 3.8) | 2.0 (0.0, 4.4) | 2.4 (0.0, 4.9) | 7.0 (0.0, 16.3) |
| **Turkey** | 0.0 (0.0, 0.1) | 0.0 (0.0, 0.2) | 0.0 (0.0, 0.2) | 0.1 (0.0, 0.2) | 0.2 (0.0, 0.6) |
| **United Arab Emirates** | 0.0 (0.0, 0.0) | 0.0 (0.0, 0.0) | 0.0 (0.0, 0.1) | 0.0 (0.0, 0.1) | 0.1 (0.0, 0.2) |
| **Uzbekistan** | 0.0 (0.0, 0.7) | 0.0 (0.0, 0.7) | 0.0 (0.0, 0.6) | 0.0 (0.0, 0.6) | 0.0 (0.0, 2.7) |
| **Yemen** | 5.0 (1.3, 8.9) | 4.9 (0.9, 9.1) | 4.2 (0.0, 8.7) | 4.0 (0.0, 9.1) | 18.1 (2.3, 35.8) |
| **Jerusalem** | 0.1 (0.0, 0.2) | 0.1 (0.0, 0.3) | 0.1 (0.0, 0.2) | 0.0 (0.0, 0.2) | 0.3 (0.0, 0.9) |
| **Mediterranean** | 2.2 (1.4, 3.1) | 3.1 (2.3, 4.0) | 2.0 (1.2, 3.0) | 2.4 (1.5, 3.4) | 9.8 (6.3, 13.5) |

Table S12: Estimated excess TB mortality rate in the Eastern Mediterranean region for the years 2020 to 2023, presented both individually by year and as a total. The estimates include the median value along with the corresponding lower and upper bounds, calculated using an ensemble framework that models two distinct sub-epidemics.

## S6.3 SMR

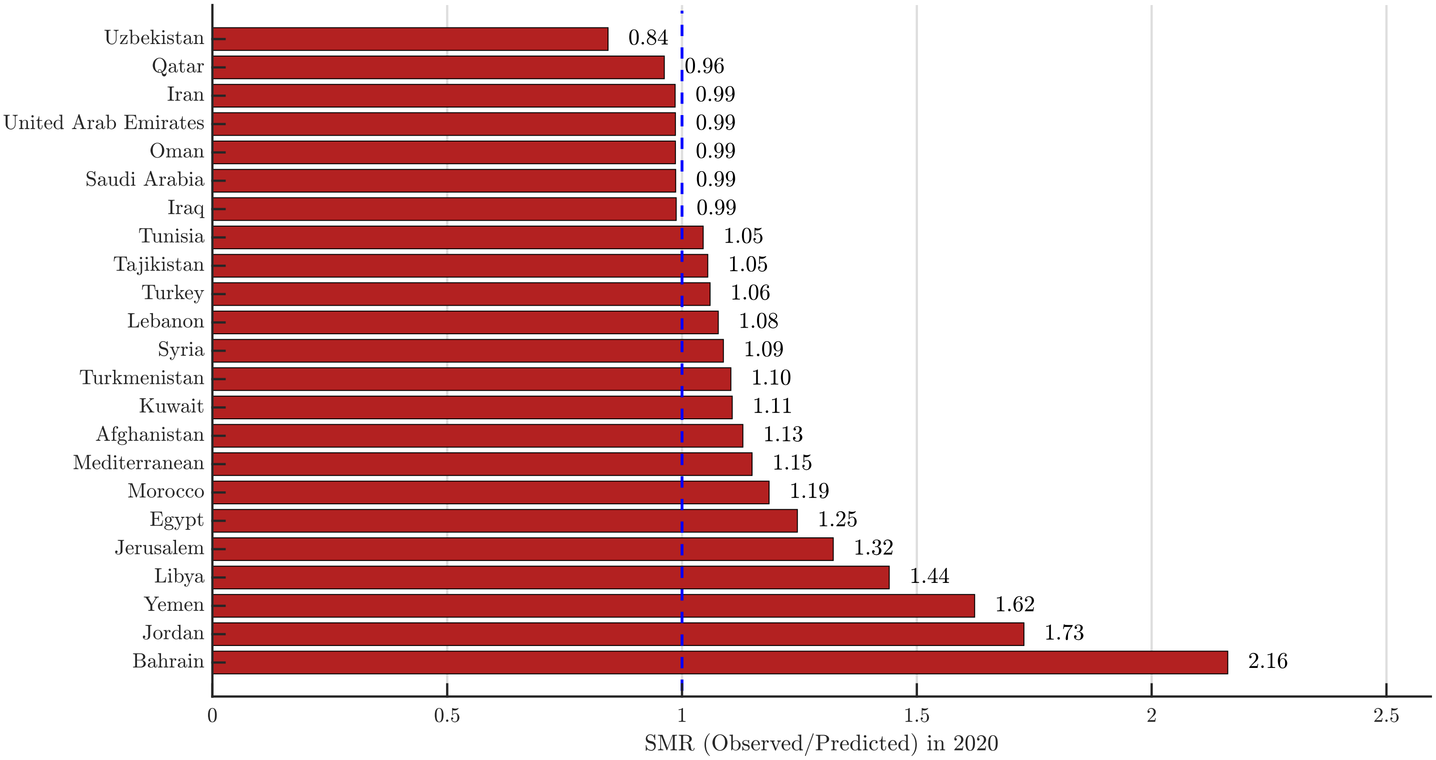


Figure S92: SMR in the Eastern Mediterranean region in 2020, estimated using an ensemble framework with two sub-epidemics. SMR is displayed horizontally in bar charts, with the exact number displayed in front of the bars.

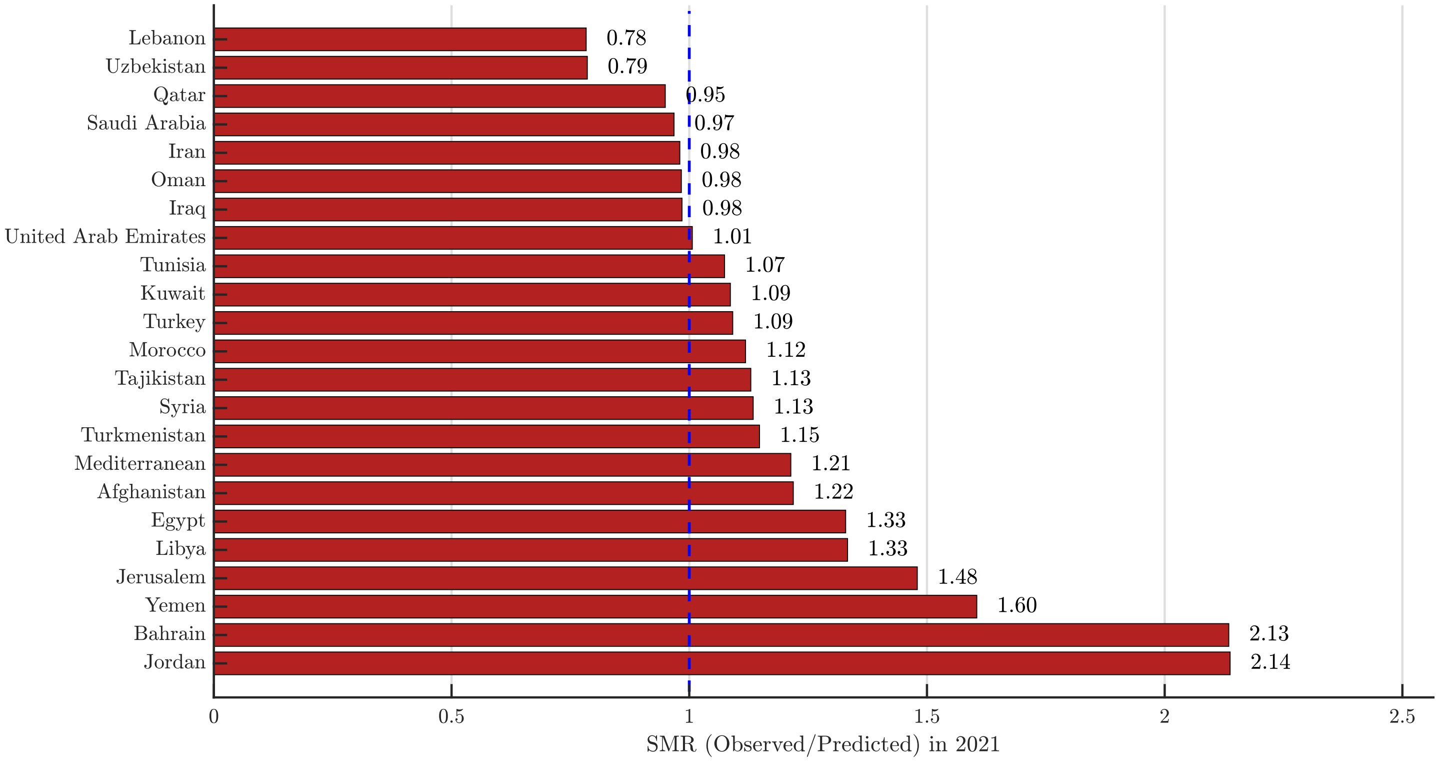


Figure S93: SMR in the Eastern Mediterranean region in 2021, estimated using an ensemble framework with two sub-epidemics. SMR is displayed horizontally in bar charts, with the exact number displayed in front of the bars.

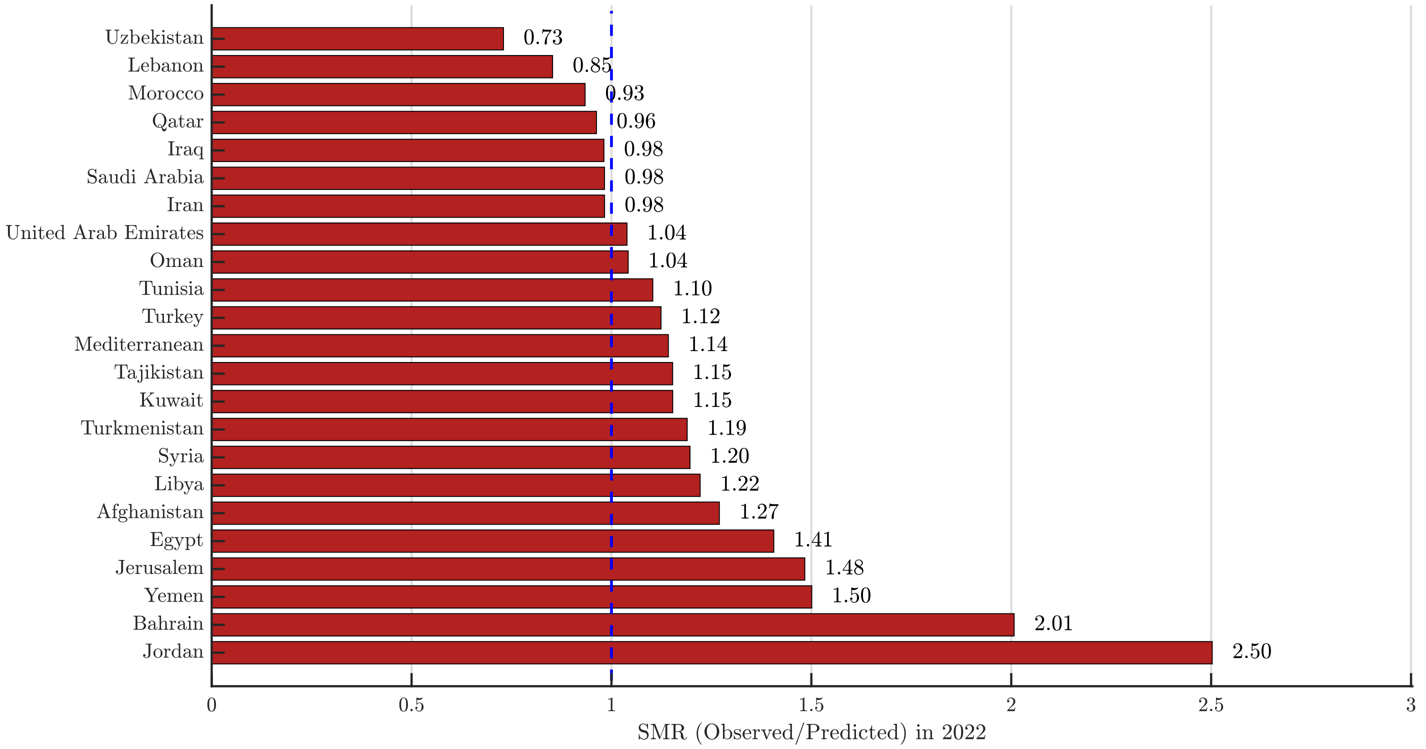


Figure S94: SMR in the Eastern Mediterranean region in 2022, estimated using an ensemble framework with two sub-epidemics. SMR is displayed horizontally in bar charts, with the exact number displayed in front of the bars.

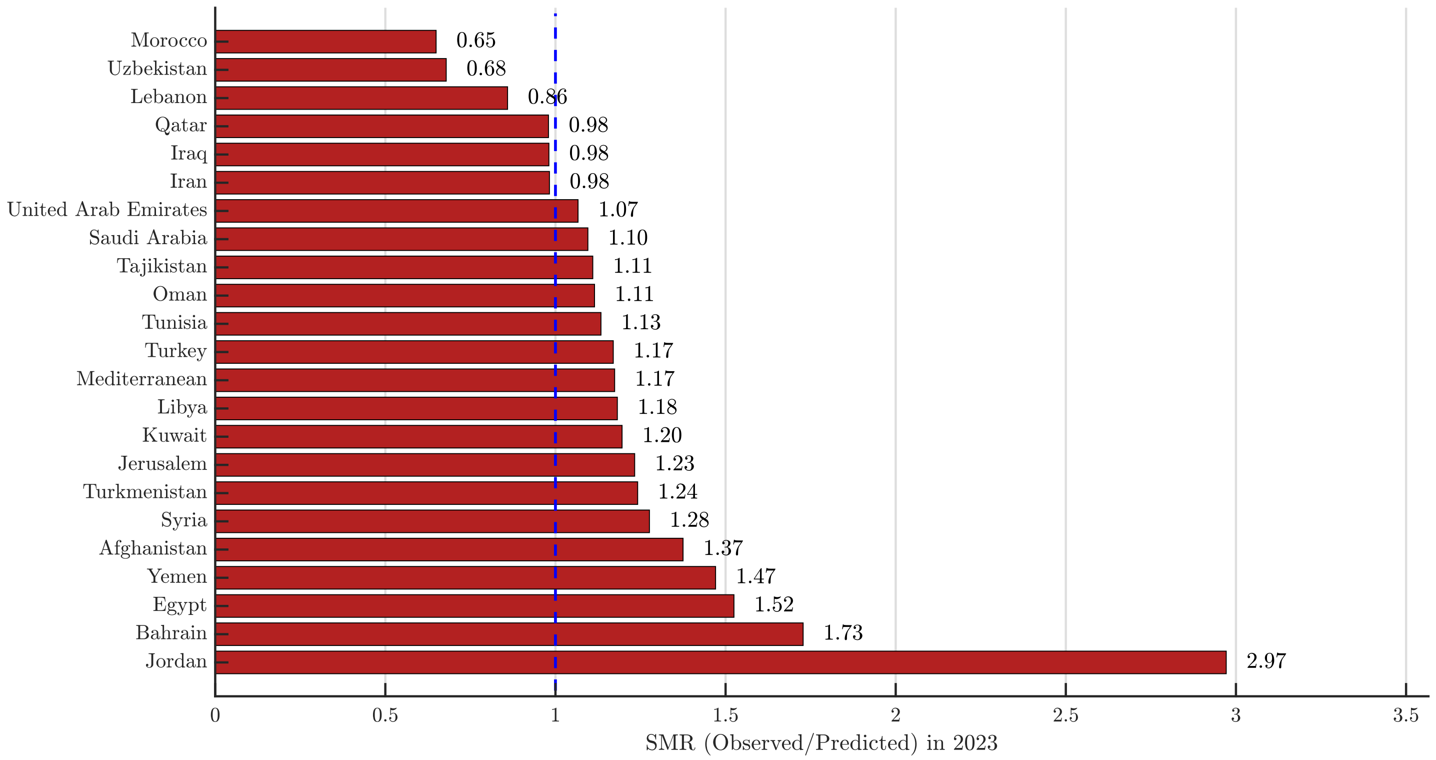


Figure S95: SMR in the Eastern Mediterranean region in 2023, estimated using an ensemble framework with two sub-epidemics. SMR is displayed horizontally in bar charts, with the exact number displayed in front of the bars.

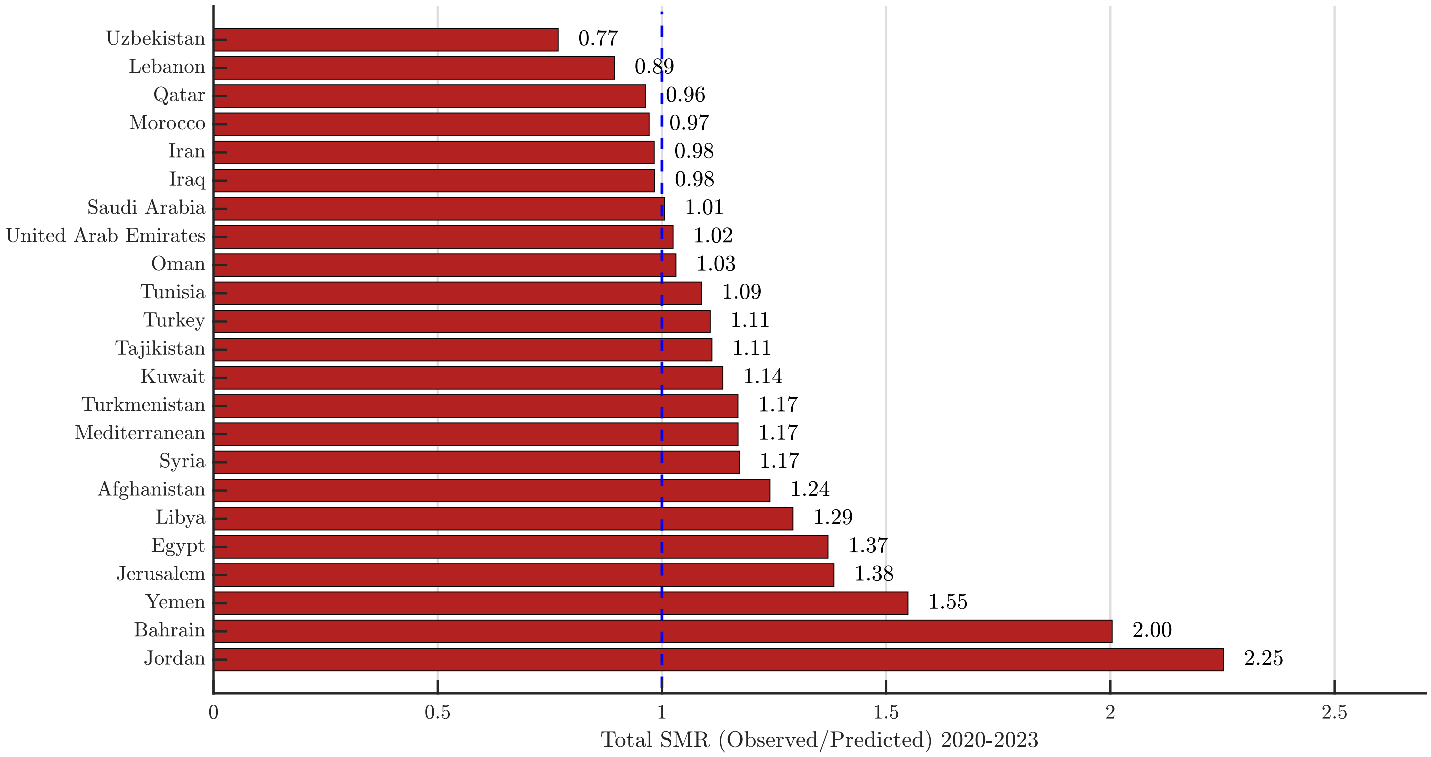


Figure S96: SMR in the Eastern Mediterranean region during 2020-2023, estimated using an ensemble framework with two sub-epidemics. SMR is displayed horizontally in bar charts, with the exact number displayed in front of the bars.

| Country | Year | Stringency Index | TB Rate per 100k |
|---|---|---|---|
| Bangladesh | 2020 | 66.0117 | 5.2 |
| Bangladesh | 2021 | 64.0338 | 7.5 |
| Bangladesh | 2022 | 19.8307 | 9.3 |
| China | 2020 | 68.2734 | 0.0 |
| China | 2021 | 74.1161 | 0.0 |
| China | 2022 | 77.5640 | 0.0 |
| DRC | 2020 | 44.3928 | 1.5 |
| DRC | 2021 | 37.1348 | 3.0 |
| DRC | 2022 | 30.7492 | 0.0 |
| India | 2020 | 64.7666 | 2.7 |
| India | 2021 | 67.1240 | 4.6 |
| India | 2022 | 41.6861 | 3.8 |
| Indonesia | 2020 | 55.0669 | 5.3 |
| Indonesia | 2021 | 68.6363 | 17.1 |
| Indonesia | 2022 | 39.6713 | 19.8 |
| Nigeria | 2020 | 55.1046 | 0.0 |
| Nigeria | 2021 | 46.0919 | 0.0 |
| Nigeria | 2022 | 21.1078 | 0.0 |
| Pakistan | 2020 | 54.5062 | 2.6 |
| Pakistan | 2021 | 57.8099 | 4.4 |
| Pakistan | 2022 | 44.4438 | 2.5 |
| Philippines | 2020 | 65.8290 | 5.2 |
| Philippines | 2021 | 72.8128 | 9.8 |
| Philippines | 2022 | 36.7836 | 7.9 |

Table S13: COVID-19 Stringency Index and excess TB deaths rate per 100,000 population across eight high-burden countries from 2020 to 2022.
